# Synthesis of Heparan Sulfate Hexadecasaccharides and Their Molecular Interaction with Mycobacterial Heparin-Binding Hemagglutinin for the Detection of *Mycobacterium tuberculosis*

**DOI:** 10.1021/jacs.5c14234

**Published:** 2025-12-22

**Authors:** Krishnagopal Maiti, Guan-Wen Huang, Yun-Hao Zhuang, Chih-Hung Wang, Jia-Ru Chang, Medel Manuel L. Zulueta, Jasper S. Dumalaog, Chiao-Chu Ku, Shih-Ching Wang, Cheng-Hsiu Chang, Chia-Lin Chyan, Horng-Yunn Dou, Gwo-Bin Lee, Shang-Cheng Hung

**Affiliations:** † Genomics Research Center, 38017 Academia Sinica, Nangang, Taipei 11529, Taiwan; ‡ Department of Chemistry, 63373 National Dong Hwa University, Hualien 97401, Taiwan; § Department of Power Mechanical Engineering, 34881 National Tsing Hua University, Hsinchu 30013, Taiwan; ∥ Institute of Infectious Disease and Vaccinology, National Health Research Institutes, Miaoli 35053, Taiwan; ⊥ Institute of Chemistry, College of Science, University of the Philippines, Diliman, Quezon City 1101, Philippines; # Department of Chemistry, 34881 National Tsing Hua University, Hsinchu 30013, Taiwan; ∇ Institute of Nanoengineering and Microsystems, 34881 National Tsing Hua University, Hsinchu 30013, Taiwan; ○ Institute of Biomedical Engineering, 34881 National Tsing Hua University, Hsinchu 30013, Taiwan; ◆ Department of Chemistry, 34912 National Cheng Kung University, Tainan 70101, Taiwan; ¶ Department of Applied Science, National Taitung University, Taitung 95092, Taiwan

## Abstract

Heparin-binding hemagglutinin (HBHA), located on the surface of *Mycobacterium tuberculosis*, binds to heparan sulfate (HS) on respiratory epithelial cells, initiating extrapulmonary dissemination and contributing to the development of latent tuberculosis. Previous characterization suggested that the lysine-rich domain of HBHA may accommodate an HS chain that is approximately twice the length of an octasaccharide. Herein, we prepared eight HS-based hexadecasaccharides through convergent assembly of a precursor, followed by divergent functional group modifications to generate varying sulfonation patterns. The hexadecasaccharide with repeating trisulfonated disaccharide units exhibited the highest binding affinity. The regions of HBHA affected by the interaction were identified by circular dichroism and multidimensional nuclear magnetic resonance analysis. Biotin functionalization of the hexadecasaccharide facilitated attachment to streptavidin-coated magnetic beads. These beads with bound HS displayed the ability to capture mycobacteria via HBHA on an integrated microfluidic chip. Aided by propidium monoazide as a DNA-binding agent and on-chip polymerase chain reaction, a means for diagnosis of tuberculosis is demonstrated.

## Graphical abstract

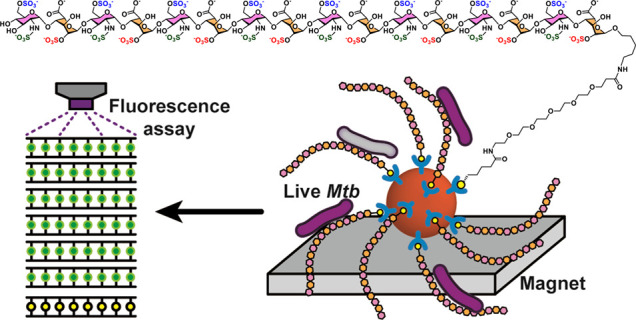

## Introduction

Nearly a quarter of the world’s population is estimated to be infected with *Mycobacterium tuberculosis* (*Mtb*).^1^ Pathogen invasion is typically initiated by inhalation of aerosols. Reproduction in alveolar macrophages in the lungs promotes the active form of tuberculosis, which led to 10.6 million new cases and 1.3 million deaths in 2022 alone.^2^ Management of this disease remains challenging despite decades of a concerted global eradication effort. Treatment involves a regimen of antibiotics for several months, and cases of multidrug resistance are prevalent. The only vaccine available—bacille Calmette-Guérin (BCG) prepared from attenuated *M. bovis*—displays variable protection rates around the world with no significant protection for adults. Following initial infection, slow replication ensues, taking about 6–8 weeks to reach quantities that trigger an immune response, and serious health issues manifest beyond 103 to 104 colony forming units (CFU).^3^ *Mtb* has manifold strategies to survive and evade the host immune systems.^4^ The infection may persist in a latent state for a long time without any clinical expression but with the risk of transition to the active form, especially when the immune system is weakened.^5^ Accordingly, research to decipher the mechanisms implicated in *Mtb* pathogenesis and to develop new therapeutic and diagnostic strategies is very important.^4,6^ A mycobacterial surface protein called heparin-binding hemagglutinin (HBHA) promotes the escape of *Mtb* from the lungs to other organs where it can lay dormant, permitting the latent form of the disease.^7^ HBHA is also a potent antigen that may be used as a diagnostic tool, for instance, in the detection of latently infected individuals.^8^ This protein has 199 amino acid residues forming a transmembrane domain (residues 12–29), a coiled-coil domain (residues 29–109), and a lysine-rich domain (residues 160–199) predisposed for interaction with the negatively charged sugar components of heparan sulfate (HS).^9^ The coiled-coil domain of HBHA serves as a critical trigger for protein dimerization, a key structural event that facilitates multivalent interactions between HBHA molecules. This dimerization ultimately promotes bacterial agglutination, a process important for *Mtb* adhesion and colonization.^10^

HS is a major proteoglycan component found on the surface of epithelial cells and in the extracellular matrix of many animal tissues. In the respiratory tract, HS is especially abundant along the epithelial lining, where it contributes to cell signaling, barrier integrity, and host–pathogen interactions.^11^ Its strategic presence on epithelial cells makes it readily accessible to microbial adhesins such as HBHA. This interaction promotes the initial attachment of *Mycobacterium tuberculosis* to the host epithelial cells, as depicted in Figure 1A.^7,12^ This linear polysaccharide consists of repeating disaccharide units composed of α-d-glucosamine (GlcN) and either α-l-iduronic acid (IdoA) or β-d-glucuronic acid (GlcA) linked in a 1 → 4 manner (Figure 1B). Its biosynthesis is not template-driven but rather is sensitive to cellular conditions that impact enzyme function. Thus, the cascade of modifications of the precursor chain leads to variable *O*- and *N*-sulfonation patterns that together with the differentially oriented carboxyl group of the uronic acid residue confer complex motifs of negative charges along the stretch of the polysaccharide. Proteins of various origins take advantage of this feature to promote physiological and pathological processes such as cell growth, angiogenesis, infection, and cancer among others.^13^ The promising diagnostic and therapeutic potentials of HS have driven research groups to synthetically access the diverse structural motifs present in this polysaccharide through either chemical or chemoenzymatic approaches.^14–46^

**Figure 1:**
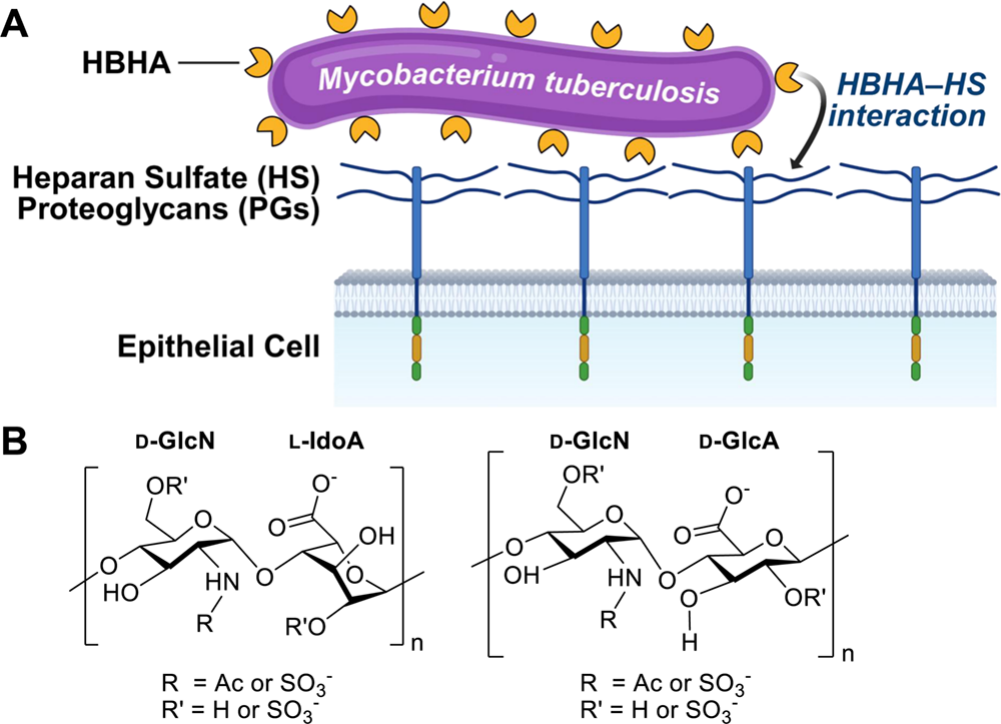
(A) Interaction of heparin-binding hemagglutinin (HBHA) at the envelope of *Mycobacterium tuberculosis* with heparan sulfate (HS) proteoglycans (PGs) on the surface of respiratory epithelial cells during the initial stage of bacterial infection; (B) the structure of the HS saccharides.

Our previous work with synthetic oligomers bearing 6-*O*- and 2-*N*-sulfonated GlcN and 2-*O*-sulfonated IdoA showed that the interaction of HBHA and HS involved electrostatic and entropic factors.^47^ Such a sulfonation pattern is common in the recurrent sulfated regions of HS as well as in heparin, a variant of HS found mainly in mastocytes.^48^ The hexasaccharide is the minimum length that could bind to HBHA,^44^ but examination of the extent of the lysine-rich domain suggests that it may accommodate a sugar length up to about twice an octasaccharide.^47^ Our preliminary evaluations on the capture of mycobacteria by magnetic beads with immobilized HS chains having 6, 8, 10, or 12 sugar units were unsuccessful (data not shown). Consequently, we envisioned the preparation of HS hexadecasaccharide analogues. An alternative procedure for preparing the l-idose monomer was explored, this time using the Mitsunobu reaction. With uniformly ^13^C- and ^15^N-labeled forms of HBHA made available, multidimensional NMR analyses were performed using the sugar with the best binding affinity. We also prepared the biotinylated form of the HS hexadecasaccharide to facilitate attachment to streptavidin-coated metallic beads. Driven by binding to HBHA, these beads with bound HS can capture live and dead mycobacteria on an integrated microfluidic chip. Together with the selectivity offered by propidium monoazide (PMA) as a DNA-binding agent and polymerase chain reaction (PCR) using a mycobacteria-specific primer,^49^ this platform may be useful for the early detection and in the treatment-response monitoring of tuberculosis.

## Results and Discussion

### Synthesis of HS Hexadecasaccharides and the ITC Evaluation of Their Binding with HBHA_110–199_

The long HS sugar backbone bears multiple and variable modifications that are challenging to capture by chemical synthesis.^50^ Typical HS binding proteins, like HBHA, have positively charged regions that complement the negative sulfate and sulfamate groups commonly found in clusters within HS domains wherein IdoA is also prevalent.^48^ We previously devised an orthogonal protecting group strategy addressing both the stereoselective and regioselective requirements of glycosidic bond formation and the functional group expected in HS.^18,44,46^ The rigor of this strategy was tested as we aimed to extend the sugar length to hexadecasaccharide by a convergent approach to form a fully protected backbone as well as enable divergent reagent applications leading to several compounds with differing functional group patterns.

The generation of the 1,6-anhydro-l-idopyranosyl derivative from diacetone-α-d-glucose is among the vital features of our strategy. Our original procedure^45^ involved an inversion of configuration through an intramolecular substitution of a mesylate group at C5 leading to epoxide formation, which upon heating in acid delivered the 1,6-anhydroidose derivative. While this transformation has been applied on various occasions,^42,45–47^ we considered the inversion at C5 by the Mitsunobu reaction as another means of access to our IdoA precursor. In this case, the partially protected glucofuranose **1**, readily prepared from diacetone d-glucose,^45,51^ was treated with triphenylphosphine (PPh_3_), *p*-nitrobenzoic acid, and diethyl azodicarboxylate (DEAD), as illustrated in Scheme 1. The result was the l-idose (l-Ido) derivative **2**, which formed diol **3** upon base hydrolysis. The anhydroidose **4** was ultimately achieved after heating in 4.5 M H_2_SO_4_ at 70 °C. Thus, an equally effective alternative access to this key building block was demonstrated. Regioselective benzoylation at the O2-position was then carried out to afford the desired l-idopyranosyl 4-alcohol **5** in 85% yield. In combination with a GlcN derivative **6** optimized for α-glycosylation using *N*-iodosuccinimide (NIS) and trifluoromethanesulfonic acid (TfOH) as a promoter, the desired disaccharide **7** was acquired in 83% yield. From compound **7**, deprotection of the 2-naphthylmethyl (2-NAP) group with 2,3-dichloro-5,6-dicyano-1,4-benzoquinone (DDQ) was then carried out to afford the glycosyl acceptor **8** in 92% yield, while a copper­(II) trifluoromethanesulfonate-catalyzed acetolysis using acetic anhydride (Ac_2_O), followed by thioglycosylation using trimethylsilyl *p*-toluenyl thioether (TMSSTol) and ZnI_2_ furnished the glycosyl donor **9** in 77% yield over two steps. A convergent synthetic route was employed, beginning with a [2 + 2] NIS/TfOH-promoted α-glycosylation between disaccharides **8** and **9** to afford tetrasaccharide **10** in 96% yield. This was followed by the preparation of the glycosyl acceptor **11** and donor **12** (78 and 93% yields, respectively), using conditions similar to those used for compounds **8** and **9**. A [4 + 4] α-glycosylation between **11** and **12**, also promoted by NIS/TfOH, furnished the desired octasaccharide **13** in 86% yield, as described in our previous reports.^44,47^

**Scheme 1:**
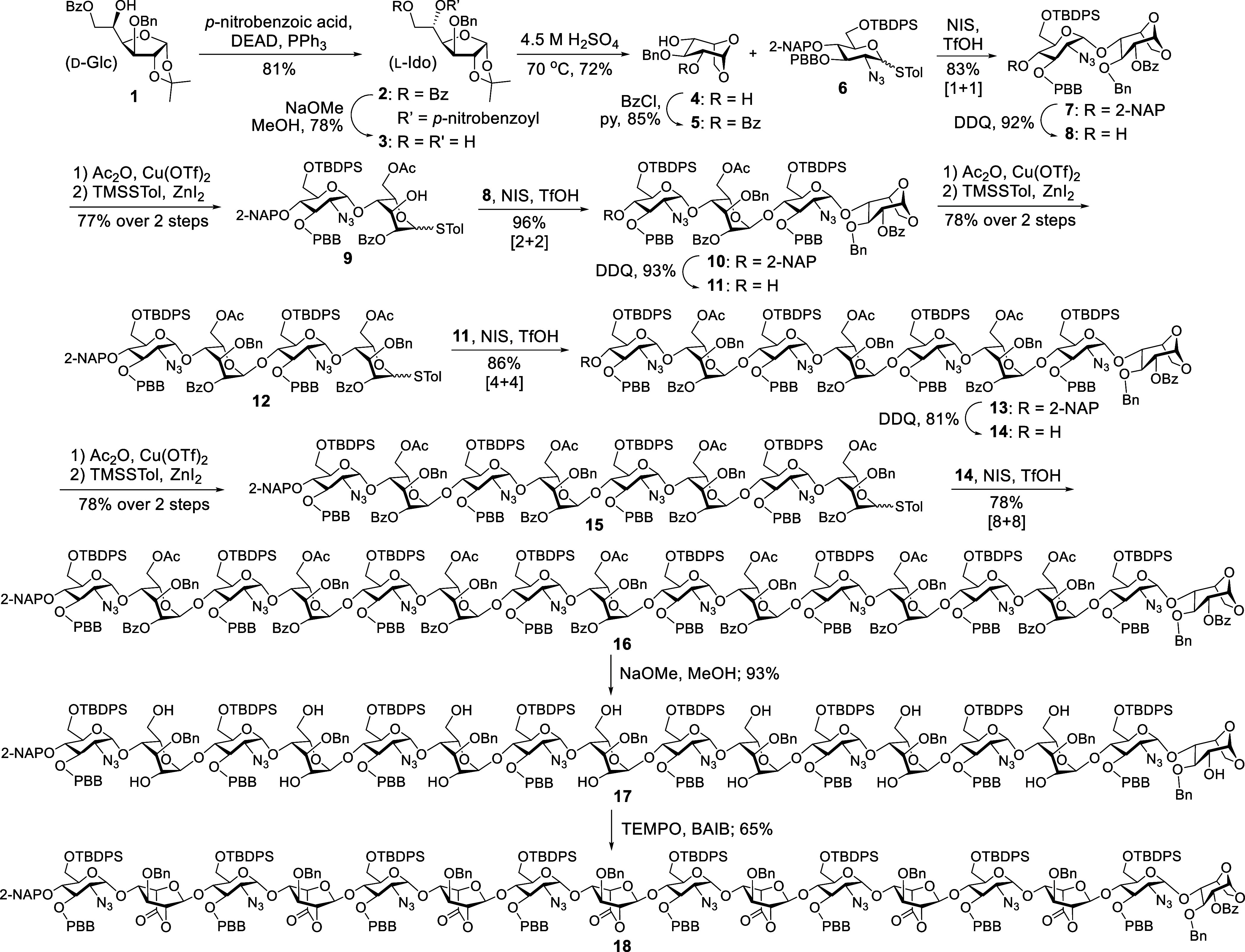
Synthesis of Heparan Sulfate Hexadecasaccharide Skeleton **16** and Its TEMPO-Mediated Lactonization

To achieve an even longer oligosaccharide chain, octasaccharide **13** was converted into glycosyl acceptor **14** (81%) through chemoselective 2-NAP ether deprotection with DDQ. In parallel, glycosyl donor **15** was prepared in 78% yield over two steps by introducing a *p*-toluenyl thioether (STol) group at the reducing end l-Ido unit via sequential acetolysis of **13** followed by thioglycosylation. The subsequent [8 + 8] glycosylation promoted by NIS/TfOH efficiently afforded the desired hexadecasaccharide skeleton **16** in 78% yield. The successful preparation of hexadecasaccharide **16** was confirmed via NMR and Matrix-Assisted Laser Desorption/Ionization Mass Spectrometry (MALDI MS) analyses (see Supporting Information).

The protons and carbons of hexadecasaccharide **16** were rigorously assigned by using ^1^H, ^13^C, 1D Total Correlation Spectroscopy (TOCSY), and 2D NMR analyses. From the 1D TOCSY analysis of hexadecasaccharide **16** (Figure 2), the complex ^1^H NMR spectrum was deconvoluted into multiple distinct patterns through the selective excitation of specific anomeric protons at defined frequencies. Using heteronuclear single quantum correlation (HSQC, Figure 3), all **16** anomeric proton and carbon signals corresponding to the individual sugar units were initially assigned with the help of their integrations from the NMR spectra. Extensive overlap between anomeric proton signals and other ring proton resonances in the 1D TOCSY spectrum impeded its resolution into **16** discrete monosaccharide patterns, thereby complicating unambiguous assignments. Nevertheless, the connectivity of sugar rings in skeleton **16** was elucidated by using a combination of 1D TOCSY, 2D TOCSY, COSY, HSQC, and HMBC NMR analyses. Non- decoupled HSQC allows direct measurement of one-bond ^1^ *J*_C–H_ at the anomeric position. A larger ^1^ *J*_C–H_ value (∼170–180 Hz) supports an α-configuration, while a smaller value (∼155–165 Hz) indicates a β-configuration.^52^

**Figure 2:**
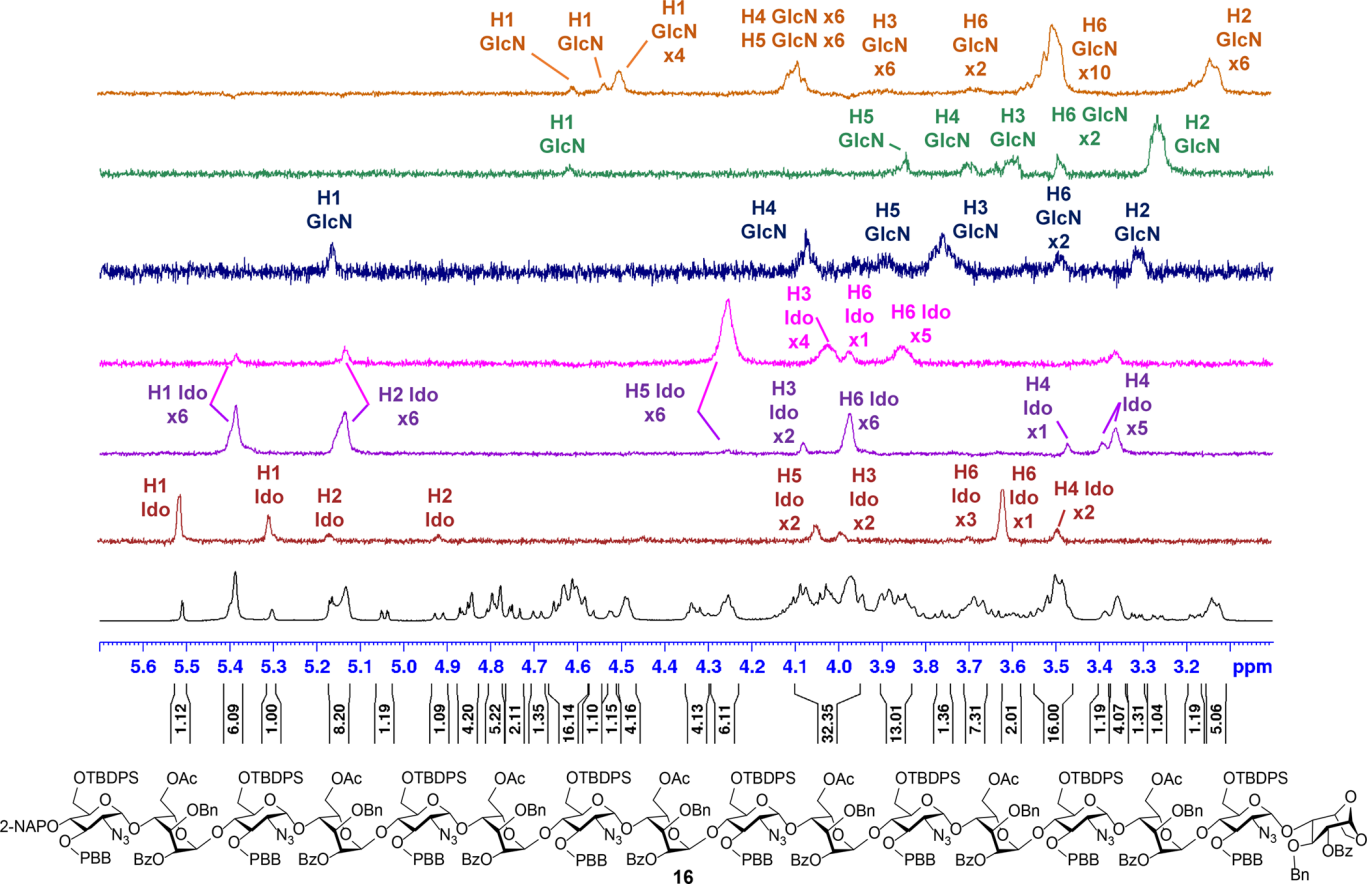
1D TOCSY analysis of hexadecasaccharide skeleton **16**.

**Figure 3:**
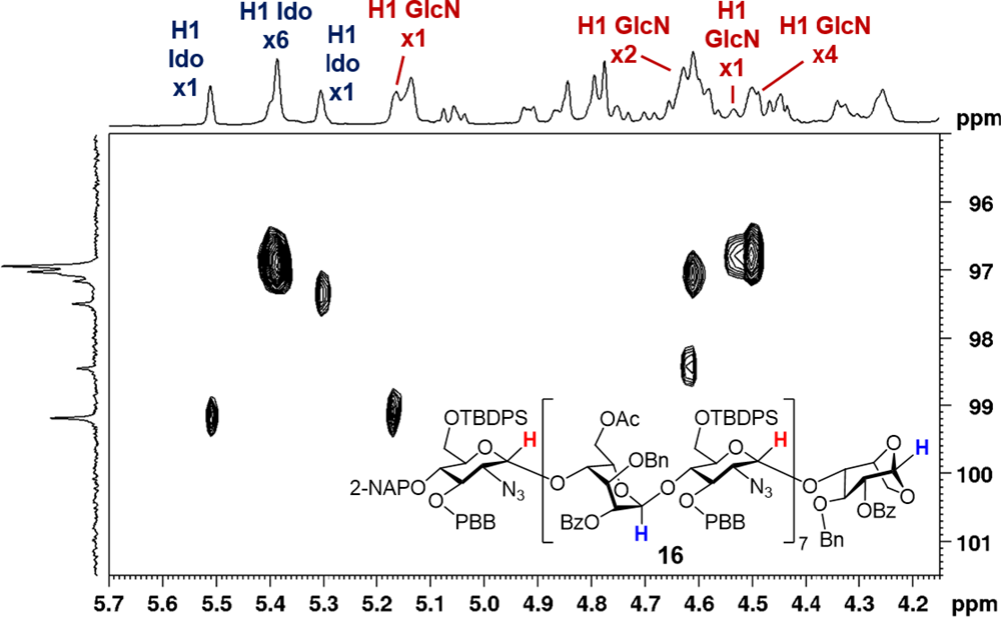
HSQC spectrum of hexadecasaccharide skeleton **16**.

From the nondecoupled HSQC analysis of all anomeric protons and carbons, all glycosidic linkages from the hexadecasaccharide skeleton **16** were confirmed to be in α-configuration (see Supporting Information). Furthermore, W-coupling correlations were observed in the 2D COSY spectrum of **16**, specifically between H1–H5 of the reducing l-Ido unit and between H1–H3 and H2–H4 of the internal l-Ido rings (see Supporting Information).

With the desired hexadecasaccharide skeleton **16** in hand, we employed a divergent strategy for functional group transformations to synthetically access the final HS compounds. A key challenge in this step was the selective and concurrent modification of the diverse functionalities distributed along the length of the hexadecasaccharide chain. Full deacylation using NaOMe in MeOH furnished **17** in 93% yield, which subsequently underwent (2,2,6,6-tetramethylpiperidin-1-yl)­oxyl (TEMPO)-mediated lactonization to afford heptalactone **18** in 65% yield. Structural confirmation of lactone ring formation was obtained from the HSQC cross-peak correlating the C6 carbonyl carbon of l-Ido with H2, providing direct spectroscopic evidence of successful lactonization. The infrared (IR) spectrum also shows a high IR ester C=O stretching band at 1794 cm^–1^.

From common heptalactone **18**, a series of divergent functional group transformations was employed to access a panel of sulfated hexadecasaccharides bearing distinct sulfonation patterns. This modular strategy enabled the generation of structurally related but functionally diverse glycosaminoglycan (GAG) mimetics from a single unified precursor. In these cases, the multiple cleavage and installation of functional groups were effectively accomplished, as monitored by one- and two-dimensional NMR techniques. As schematically depicted in Scheme 2, the first subset of these sulfonated hexadecasaccharide derivatives was synthesized through a sequence of reactions including desilylation using HF·pyridine, followed by lactone ring opening with LiOH, and global hydrogenolysis with H_2_ and Pd­(OH)_2_/C, affording compound **19** in 45% yield over three steps. Subsequently, the *N*-acetylation of **19** with acetic anhydride (Ac_2_O) yielded the nonsulfonated hexadecasaccharide **20** in 85%, while treatment of **19** with SO_3_·pyridine yielded the *N*-sulfonated hexadecasaccharide **21** in 83%.

**Scheme 2:**
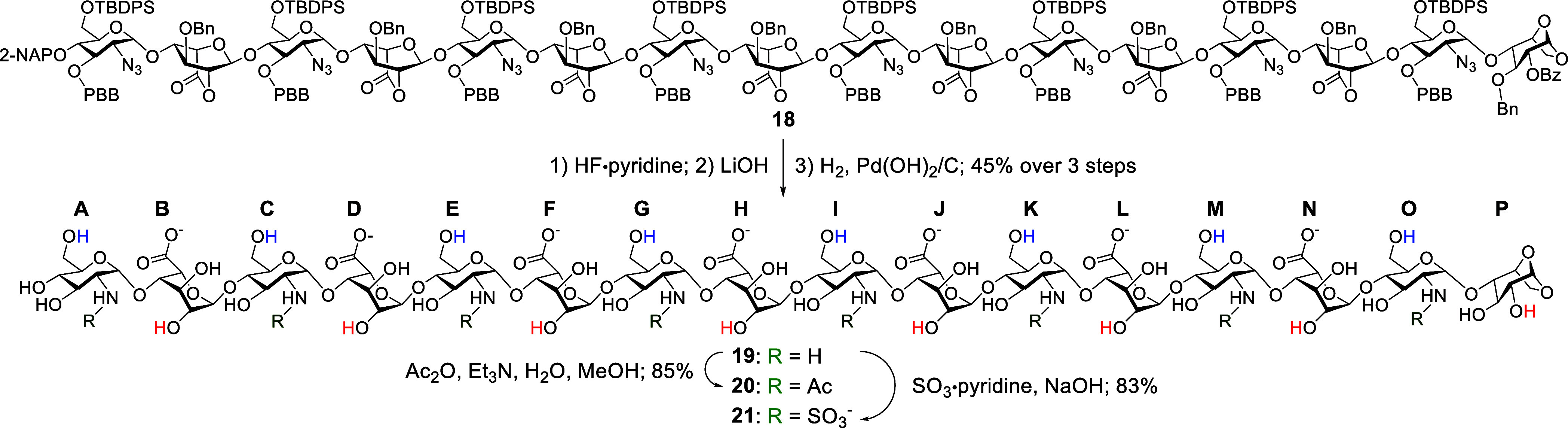
Synthesis of the Target HS Hexadecasaccharides **20** and **21**

Another subset of structurally distinct hexadecasaccharides was synthesized from **18** via an alternative sequence of functional group transformations. In the second set of reactions (Scheme 3), the synthesis began with desilylation using HF·pyridine, lactone ring opening with LiOH, regioselective *O*-sulfonation with SO_3_·Et_3_N, and then hydrogenolysis with H_2_ and Pd­(OH)_2_/C, which afforded **22** in 41% yield over four steps. This was then followed by either *N*-acetylation using Ac_2_O in aqueous MeOH to obtain the 6-*O*-sulfated GlcN and 2-*O*-sulfated IdoA hexadecasaccharide **23** in 81% yield or *N*-sulfonation via SO_3_·pyridine to get the 2,6-*O-* and *N*-sulfated hexadecasaccharide **24** in 77% yield.

**Scheme 3:**
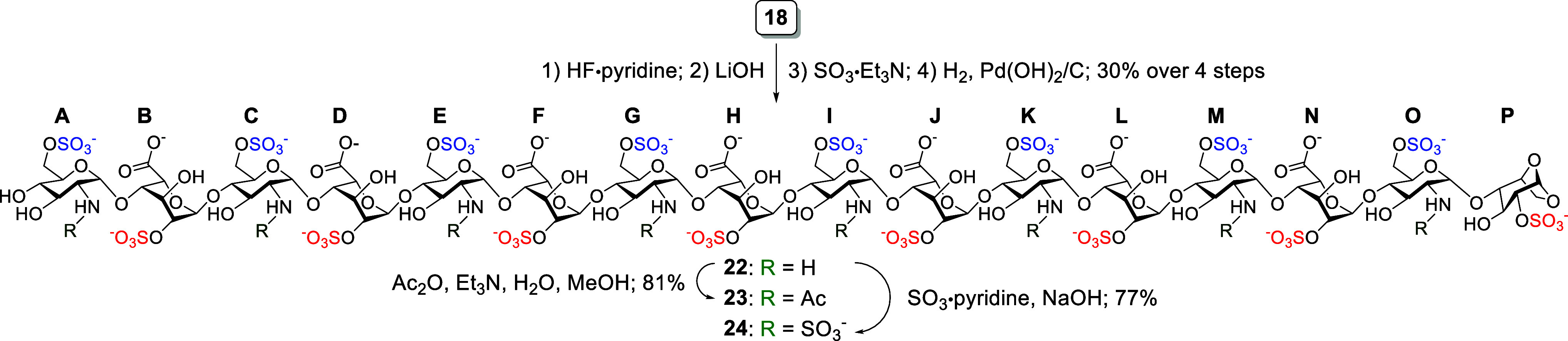
Synthesis of the Target HS Hexadecasaccharides **23** and **24**

Similarly, the third set of derivatives was obtained through a modified route from hexadecasaccharide **18** (Scheme 4). The sequence involved lactone ring opening with LiOH, regioselective *O*-sulfonation at the O2-position of l-IdoA using SO_3_·Et_3_N, desilylation using HF·pyridine, and global hydrogenolysis with H_2_ and Pd­(OH)_2_/C, culminating in the formation of hexadecasaccharide **25** in 30% yield over four steps. Subsequent *N*-acetylation of **25** afforded the 2-*O*-sulfonated hexadecasaccharide **26** in 76% yield, while *N*-sulfonation with SO_3_·pyridine furnished the 2-*O*- and *N*-sulfonated derivative **27** in 64% yield. This carefully orchestrated sequence of functional group transformations in the synthesis of the first six target hexadecasaccharides demonstrates the high level of regioselective and chemoselective control achievable in constructing complex sulfonation patterns across the hexadecasaccharide scaffold. The last subset of sulfated hexadecasaccharides was obtained from the chemoselective deacetylation of **16** using magnesium methoxide (Mg­(OMe)_2_), which allowed us to get the primary heptaol **28** in 82% yield (Scheme 5). This intermediate **16** with primary alcohol on the l-Ido units underwent a series of functional group transformations, including TEMPO-mediated oxidation, desilylation with HF·pyridine, and *O*-sulfonation with SO_3_·Et_3_N, followed by debenzoylation with LiOH, affording **29** in 32% yield over four steps. Hydrogenolysis using H_2_ and Pd­(OH)_2_/C was then carried out, which gave **30** in a 63% yield. Finally, *N*-acetylation of **30** with Ac_2_O yielded the target hexadecasaccharide **31** in 80% with the 6-*O*-sulfonated GlcN units, while treatment of **30** with SO_3_·pyridine furnished the hexadecasaccharide **32** featuring 6-*O*- and *N*-sulfonated GlcN residues in 74% yield.

**Scheme 4:**
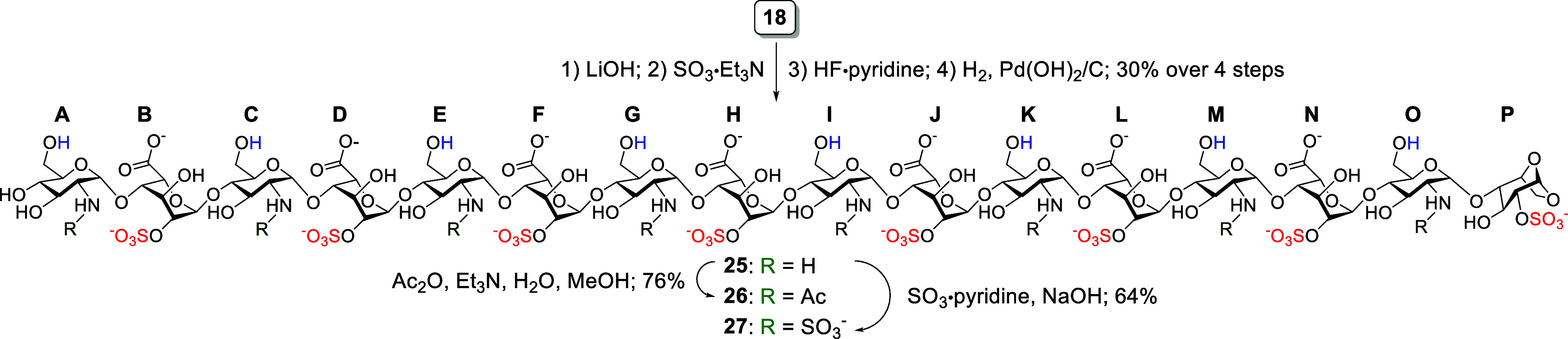
Synthesis of the Target HS Hexadecasaccharides **26** and **27**

**Scheme 5:**
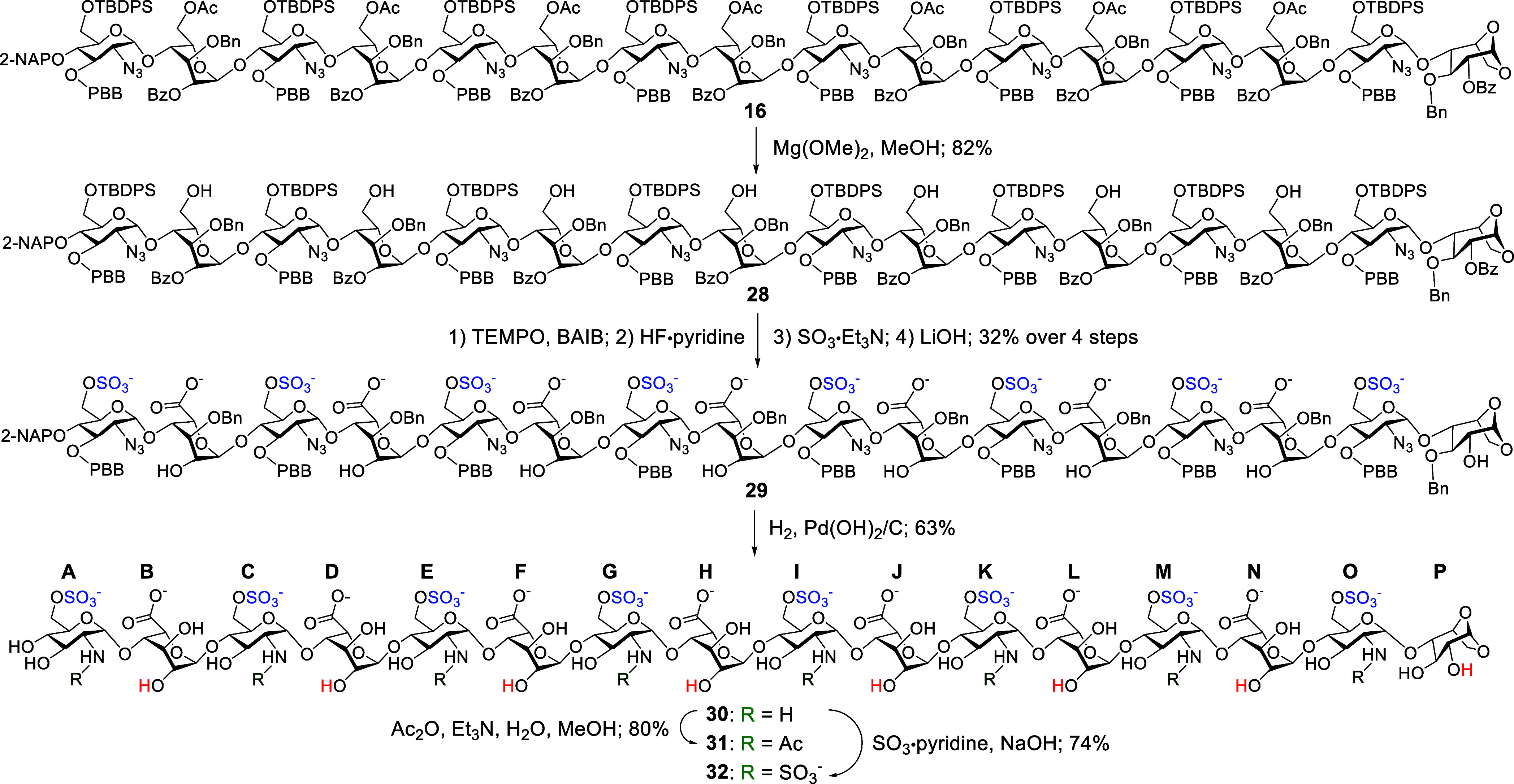
Synthesis of the Target HS Hexadecasaccharides **31** and **32**

Structural confirmation of all synthesized HS hexadecasaccharides was carried out through a combination of NMR spectroscopy and MALDI MS. At each key intermediate of the synthesis, these analytical methods verified that the correct intermediates were obtained. Gratifyingly, we found that all functionalizations could be clearly identified by the highly indicative downfield shifts (4–5 ppm) in the ^13^C NMR resonance of the nitrogen-bonded carbon from the GlcN units during the *N*-sulfonation steps (Figure 4, Table S1). We also figured out that *N*-acetylated and *N*-sulfonated GlcN residues can also be differentiated by the inspection of their ^13^C chemical shifts. Based on these comparisons, sulfonamido-linked carbons generally exhibit more downfield chemical shifts compared to acetamides, likely due to the stronger electron-withdrawing effect of the sulfonamide group. Despite the solvent differences between the azido-containing intermediates and the amino-, acetamido-, and sulfonamido-substituted hexadecasaccharides, pronounced variations in their ^13^C NMR chemical shifts remain clearly evident. All these analyses further confirmed the functional group transformations, complete removal of all protecting groups, as well as the precise and full incorporation of the intended sulfonation patterns, ensuring the structural integrity and purity of each compound.

**Figure 4:**
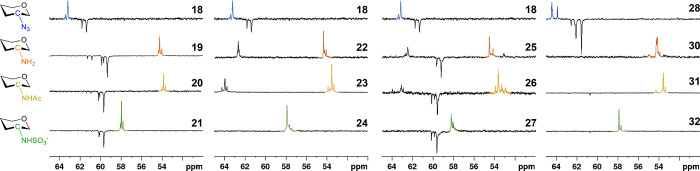
Comparisons of glucosamine carbons attached to azido (N_3_), amino (NH_2_), acetamido (NHAc), and sulfonamido (NHSO_3_^–^) groups in DEPT-135 spectra (in CDCl_3_ for **18** and **28**, in D_2_O for the other compounds).

The binding affinities of the synthesized hexadecasaccharides were assessed using isothermal titration calorimetry (ITC) against HBHA_110–199_, an easily handled surrogate for full-length HBHA^44,47^ that contains the lysine-rich domain (Figure S1, Supporting Information). The protein solution in Tris buffer (pH 7.6) was titrated with the sugar dissolved in the same buffer at 25 °C, with blank titration correcting for the heat of dilution. As summarized in Table 1, HS hexadecasaccharide **24** demonstrated the strongest binding to HBHA, with a dissociation constant (*K*_D_) of 1.87 μM, indicating the highest affinity among the tested hexadecasaccharides. In contrast, hexadecasaccharides **20**, **26**, **27**, and **31** showed no detectable binding, highlighting the importance of both *N*- and *O*-sulfonation in molecular recognition. Compound **21**, which lacks *O*-sulfonation, bound with significantly reduced affinity (*K*_D_ = 16.0 μM), while **23** (2,6-*O*-sulfonated) and **32** (6-*O*- and *N*-sulfonated) displayed comparatively weaker affinity to HBHA, with *K*_D_ values of 2.63 and 2.36 μM, respectively, relative to **24**. ITC binding curves (Figures S1 and S2) revealed that hexadecasaccharides interacting with HBHA exhibited endothermic binding behavior, indicating that the interactions are likely entropy-driven. In addition, **24** exhibited a better binding affinity with HBHA compared to a previously reported HS octasaccharide **HS8** (Figure S3), featuring the same sulfonation motif. Collectively, these results emphasize the role of precise sulfonation patterns, particularly the presence of both 6-*O*- and 2-*N*-sulfonation in GlcN and 2-*O*-sulfonation at IdoA, in enhancing the molecular recognition and interaction affinity.

**Table 1:** ITC Binding of HS Hexadecasaccharides with HBHA_110–199_

compound	dissociation constant, *K*_D_ (μM)^a^
**20**	no binding detected
**21**	16.0 ± 1.5
**23**	2.63 ± 0.11
**24**	1.87 ± 0.06
**26**	no binding detected
**27**	no binding detected
**31**	no binding detected
**32**	2.36 ± 0.30

a ITC measurements were performed in two trials, with results presented as the average ± standard deviation.

### Circular Dichroism (CD) Experiments with HBHA and Hexadecasaccharide **24**

CD was used to evaluate the secondary structural changes in full-length HBHA as compound **24** is added to the mix. When 1 equiv of **24** was added to the HBHA solution, a marked increase in negative molar ellipticity was observed, especially at 208 and 222 nm far-ultraviolet wavelengths (Figure 5). These spectral shifts indicate a strengthening or stabilization of α-helical domains, a characteristic structural response often observed when carbohydrate binding modulates protein conformation, particularly in the context of GAG–HBHA interactions.^53^ At the expense of some β-sheet residues, an approximately 8% increase in the α-helical content—roughly equivalent to **16** residues—in the bound HBHA was estimated by Dichroweb (Table 2).^54,55^ To confirm that the structural change of HBHA is specifically caused by **24**, a nonbinding hexadecasaccharide **20** was used as a negative control. Addition of varying equivalents of **20** to HBHA did not induce any significant conformational change in the protein (Figure S4). Conversely, there were no noteworthy changes observed in the CD spectra when **24** was added to HBHA_110–199_ (data not shown). These results indicate that the binding of hexadecasaccharide **24** to the C-terminal region induces a conformational change in the coiled-coil domain of the protein.

**Figure 5:**
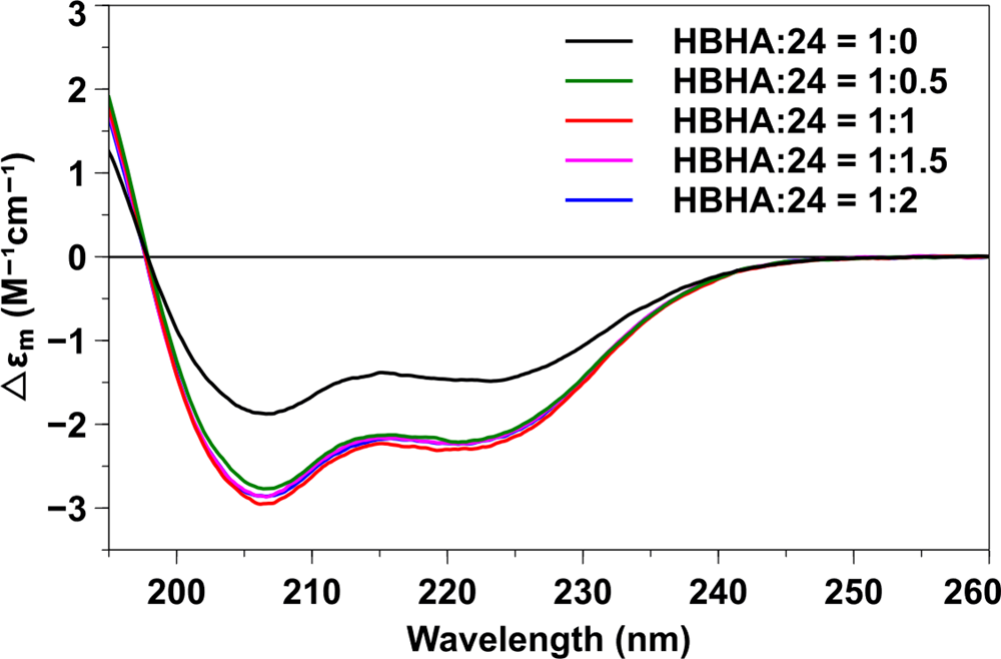
Far-ultraviolet CD spectra of full-length HBHA upon association with compound **24** in varying molar ratios.

**Table 2:** Prediction of Percentages of Protein Secondary Structure from CD Spectra of Full-Length HBHA with Compound **24** in Varying Molar Ratios^a^

	HBHA:compound 24			
secondary structure	1:0	1:0.5	1:1	1:1.5
α-helix (%)	16.5	23.6	24.9	24.4
β-sheet (%)	31.8	24.0	23.2	23.4
random coil (%)	51.7	52.4	51.9	52.2
total (%)	100.0	100.0	100.0	100.0

a Protein secondary structure percentages were estimated from the far-UV (195–260 nm) CD spectra using the CDPRO program with thirty-three basis sets.

### Chemical Shift Perturbation of HBHA_110–199_

Multidimensional NMR analyses were carried out using the ^13^C- and ^15^N-labeled HBHA_110–199_, afforded as described previously,^46^ and compound **24** to examine the affected components while the protein and sugar are interacting. Backbone and side-chain chemical shift values were established first with free HBHA_110–199_ by correlating intra- and inter-residue resonances from standard three-dimensional NMR spectra. Subsequently, changes in chemical shifts were noted when compound **24** was added in different protein-to-sugar molar ratios (1:0.5, 1:1, 1:1.5, and 1:2). Chemical shifts were confirmed by inspection of intraresidue and sequential nuclear Overhauser effects (NOE) in ^13^C, ^15^N-edited NOE spectroscopy (NOESY)–HSQC spectra.

The weighted chemical shift deviations of the backbone ^15^N, carbonyl ^13^C, and ^1^H bonded to amide nitrogen, Δδ_residue_, were plotted against residue number (Figure 6A). Residues 167–196 at the C-terminal region of HBHA_110–199_ were the most perturbed, with Δδ_residue_ being larger than the average value (0.035 ppm). These residues are likely in proximity to the interaction surface or underwent major structural readjustment and therefore are predicted to play pivotal roles in the binding event. In comparison, the average of Δδ_residue_ for all residues of HBHA_110–199_ with the octasaccharide variant **HS8**, as reported previously,^47^ is smaller (0.019 ppm). When the amount of **24** in the mixture was varied, the traced movement of the ^1^H–^15^N-HSQC cross-peak of labeled HBHA_110–199_ (Figure 6B) indicated a 1:1 binding stoichiometry between the protein and the sugar. The structures of both **HS8** and **24** are shown in Figure 6C. The average *K*_D_ value in the most perturbed region (residues 178–199) is 1.5 μM, which is close to the ITC results (Table S2). NMR chemical shift perturbation analysis was conducted using varying molar ratios of HBHA_110–199_ with compound **24**, alongside comparative studies with **HS8**.^46^ Focusing on the lysine-rich C-terminal domain of HBHA (Figure 7A), residue-specific chemical shift deviations across the repeat sequence (Figure 7B–F) were plotted as a function of the molar ratio between HBHA_110–199_ and either compound **24** or **HS8**. The results revealed an entropically driven interaction between HBHA and compound **24**, in line with the ITC results, underscoring the prominent role of hydrophobic forces in the binding mechanism. This hydrophobicity-driven interaction is likely facilitated by the key presence of several alanine and lysine residues within the HS-binding C-terminal region of HBHA, which together generate a favorable molecular environment for ligand binding.^48^

**Figure 6:**
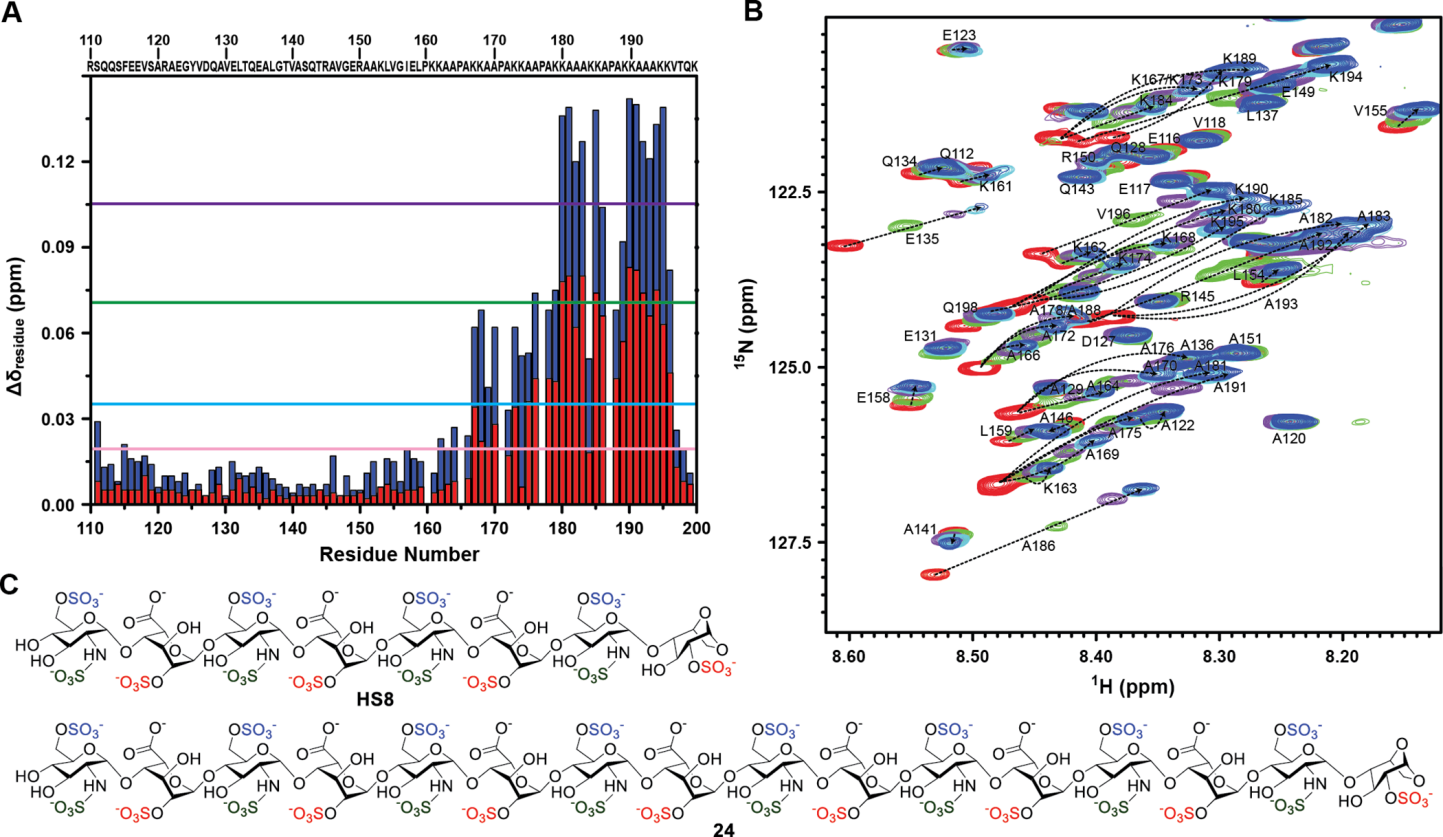
Modification of chemical shifts associated with HBHA_110–199_ upon binding to the synthetic HS analogue. (A) Overall chemical shift perturbation, Δδ_residue_, of HBHA_110–199_ upon association with compound **24** (in blue bars) plotted against residue number and compared to the chemical shift perturbation with the octasaccharide analogue **HS8** (in red bars) reported previously.^47^ The protein-to-sugar molar ratio is 1:2. The light pink line corresponds to the average Δδ_residue_ on binding with **HS8** (0.019 ppm). The light blue line corresponds to the average Δδ_residue_ in the case with 24 (0.035 ppm); two- and three-fold averaged Δδ_residue_ are indicated by the green and purple lines, respectively. (B) Overlaid ^1^H–^15^N HSQC spectra showing the movement of the cross-peaks of key residues of labeled HBHA_110–199_ upon mixing with different amounts of compound **24**. The spectrum of unbound HBHA_110–199_ is in red, in green for the complex of HBHA_110–199_ to **24** with a molar ratio of 1:0.5, in magenta for a ratio of 1:1, in cyan for a ratio of 1:1.5, and in blue for a ratio of 1:2. (C) Structure of octasaccharide **HS8** and hexadecasaccharide **24**.

**Figure 7:**
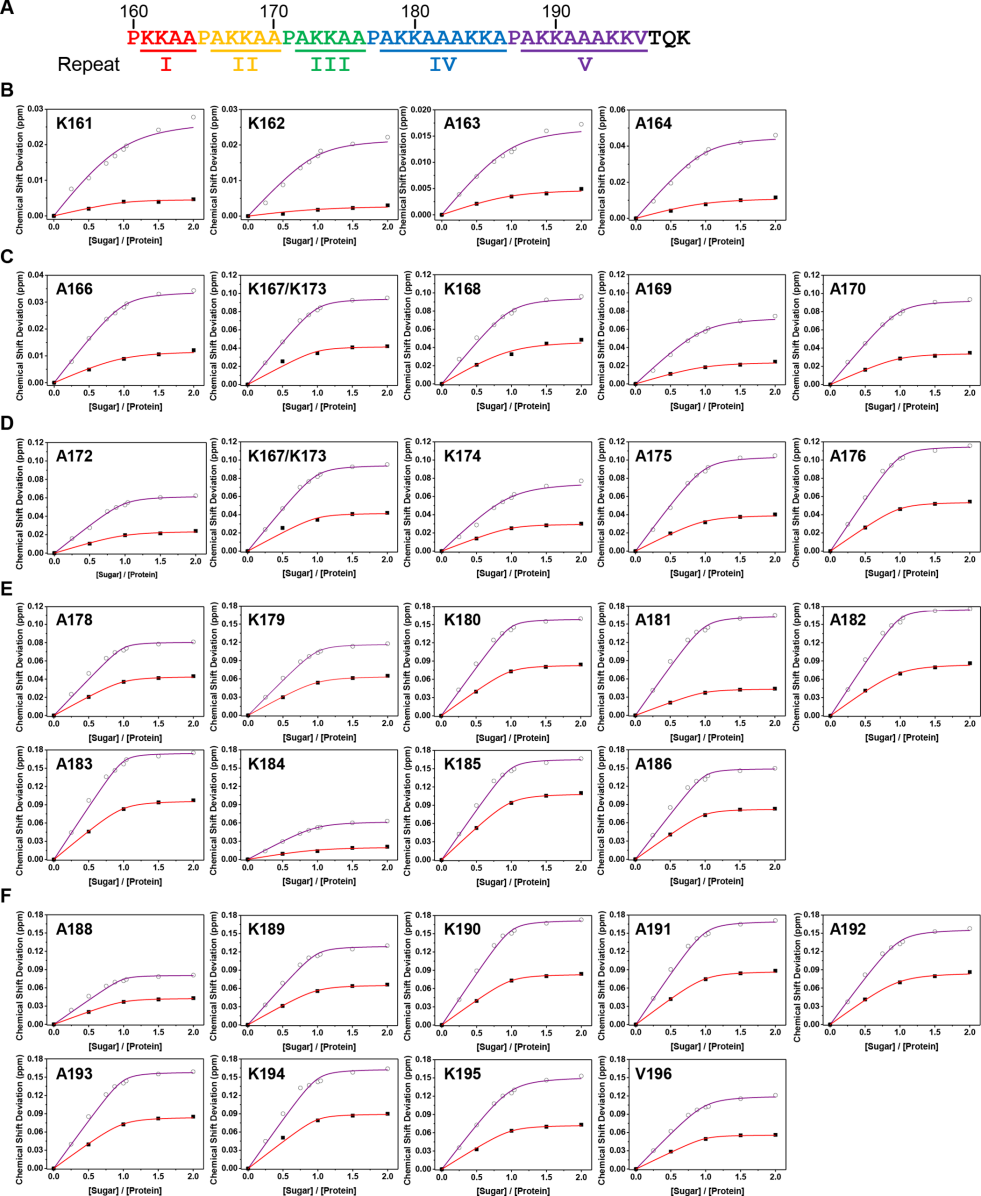
NMR chemical shift perturbations in different molar ratios of HBHA_110–199_ and compound **24** with comparison to the octasaccharide analogue (**HS8**).^46^ (A) Amino-acid sequence of the lysine-rich domain of HBHA. (B–F) Plots of the chemical shift deviation of each residue of the respective repeat sequence of the lysine-rich domain, labeled I–V in panel A, as a function of the molar ratio of HBHA_110–199_ to compounds **24** and **HS8**. The purple solid line showed the best fit for **24**; the red solid line, for **HS8**.

Concerning compound **24**, the opposite ends of the sugar (residues A, M, N, O, and P, see Scheme 3 for the residue labels) have well-dispersed chemical shifts, whereas all the residues in the middle of the sequence are highly overlapped. IdoA residues B, D, F, H, J, and L are degenerate, and GlcNS6S residues C, E, G, I, and K are also degenerate (Table S3). Coupling constants (Table S4) were used to elucidate the ring conformations and to derive the dihedral angles. While all GlcN residues are in the ^4^ *C*_1_ conformation, the IdoA residues assumed the ^2^ *S*_O_ conformer with some conformational population in ^1^ *C*_4_. Upon interaction with HBHA, hexadecasaccharide **24** exhibited an increased preference for the ^2^ *S*_O_ conformation of the IdoA residues. Consistent with restricted motion, more NOEs were found in bound than in free **24.**

### HS Hexadecasaccharide-Conjugated Magnetic Beads for *Mtb* Detection

The effectiveness of tuberculosis treatment is maximized when the disease is identified at an early stage, as this allows for timely intervention before significant lung damage or transmission occurs. This highlights the importance of continued exploration of disease detection strategies.^56^ Moreover, reliable protocols for monitoring viable mycobacterial counts during antibiotic treatments are essential for assessing treatment efficacy and forming clinical decisions. Accurate quantification of live bacterial load provides prognostic guidance, helping to distinguish between successful therapeutic response and potential relapse or treatment failure.^57^ Currently, the clinical diagnosis of tuberculosis relies on a combination of tools, including chest X-rays, tuberculin skin testing,^58^ examination of sputum smears,^59^ bronchoscopy for direct airway assessment,^60^ and sputum culture for bacterial growth confirmation. In recent years, nucleic acid amplification tests (NAATs), such as PCR-based assays,^61^ have enhanced the sensitivity and speed of *Mtb* detection. Despite these advancements, many of these methods cannot effectively differentiate between live and dead bacilli, which limits their prognostic value during treatment monitoring. We recently reported the use of α-HBHA antibody-conjugated magnetic beads to capture live and dead mycobacteria using an integrated microfluidic chip.^49^ With dead pathogens unable to maintain membrane integrity, their DNA becomes susceptible to modification by PMA,^62^ leaving only the DNA of a live *Mtb* being detectable by PCR. Thus, in this case, the interference from dead *Mycobacterium* in the analysis is effectively eliminated.

Given the strong affinity of hexadecasaccharide **24** for HBHA demonstrated by the ITC measurements, we envisioned using this sugar chain to capture mycobacteria upon conjugation with magnetic beads. The incorporation of a biotin tag should enable this conjugation with the hexadecasaccharide. Accordingly, hexadecasaccharide skeleton **16** was converted into the thioglycosyl donor **33** via anhydro-ring opening, followed by thioglycosylation (Figure 8). The coupling with alcohol **34** readily supplied linker-attached compound **35** in 86% yield. This intermediate was then subjected to selective deacylation with NaOMe in aqueous MeOH, yielding **36** in 90%, then primary alcohol oxidation with TEMPO, leading to the polylactone **37** in 60% yield. A series of functional group transformations involving desilylation with HF·pyridine, alkaline treatment with LiOH to hydrolyze the lactones, and then *O*-sulfonation with SO_3_·Et_3_N, then produced the 6-*O*-sulfonated hexadecasaccharide **38** in 48% yield over three steps. Azido reduction by PPh_3_, followed by *N*-sulfonation with SO_3_·pyridine, and global hydrogenolysis with H_2_ and Pd­(OH)_2_/C ultimately provided the HS-based hexadecasaccharide **39** in 38% yield over three steps. Hexadecasaccharide **39** contains an aminopentyl linker with 6-*O*- and *N*-sulfonation at the GlcN units and 2-*O*-sulfonation at the IdoA residues. The biotin component was introduced through the amine group using activated biotin ester **40** and Et_3_N to produce target biotinylated compound **41** in 81% yield. NMR and high-resolution mass analyses successfully confirmed the formation of hexadecasaccharide **41.**

**Figure 8:**
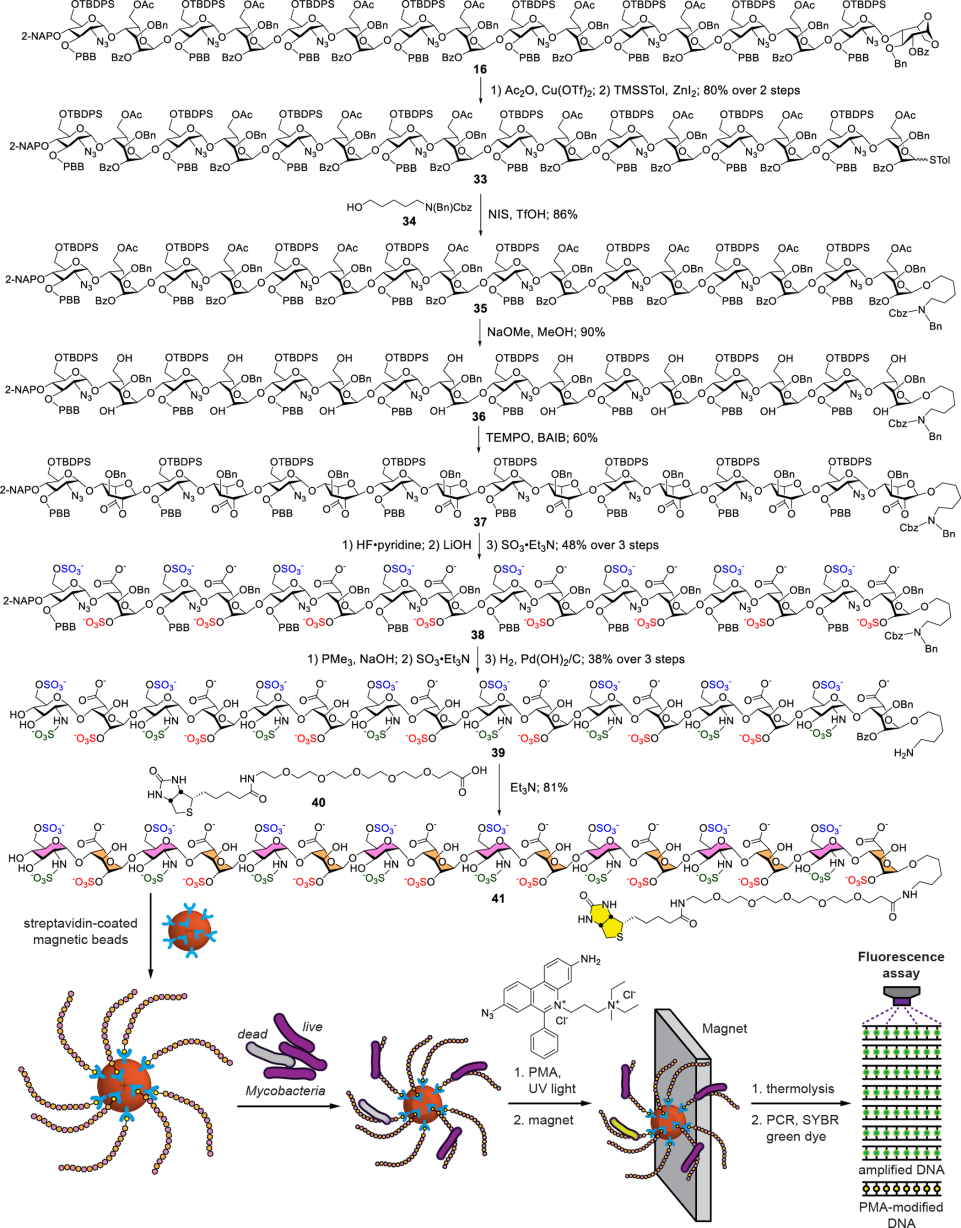
Preparation of biotinylated hexadecasaccharide **41** and the general concept of mycobacterial capture for analysis. The dead mycobacteria (gray color) lose membrane integrity that permits modification of their DNA by PMA treatment (yellow color). Cbz, benzyloxycarbonyl; PCR, polymerase chain reaction; PMA, propidium monoazide.

Compound **41** was conjugated onto MyOne beads through the streptavidin units coating its surface, for which biotin binds with high affinity. Following blocking with bovine serum albumin to prevent nonspecific binding, the hexadecasaccharide-conjugated magnetic beads were washed and then resuspended in water. HBHA at the cell surface of dead or live mycobacteria should attach to these beads upon incubation on account of its association with the HS chains. As a result, mycobacteria were captured by the beads (Figure 9A). These beads were then treated with PMA followed by irradiation with UV light at 254 nm. The live mycobacteria can have their DNA amplified after thermolysis, whereas the DNA of the dead mycobacterium modified by PMA is unaffected by PCR.^59^ In this case, the conserved 508-base pair region of the *rpo*B gene, encoding the β-subunit of bacterial RNA polymerase, was amplified using mycobacterium-specific primers (Figure 9B). Indeed, the dead mycobacteria were not detected by gel electrophoresis and the fluorescence assay.

**Figure 9:**
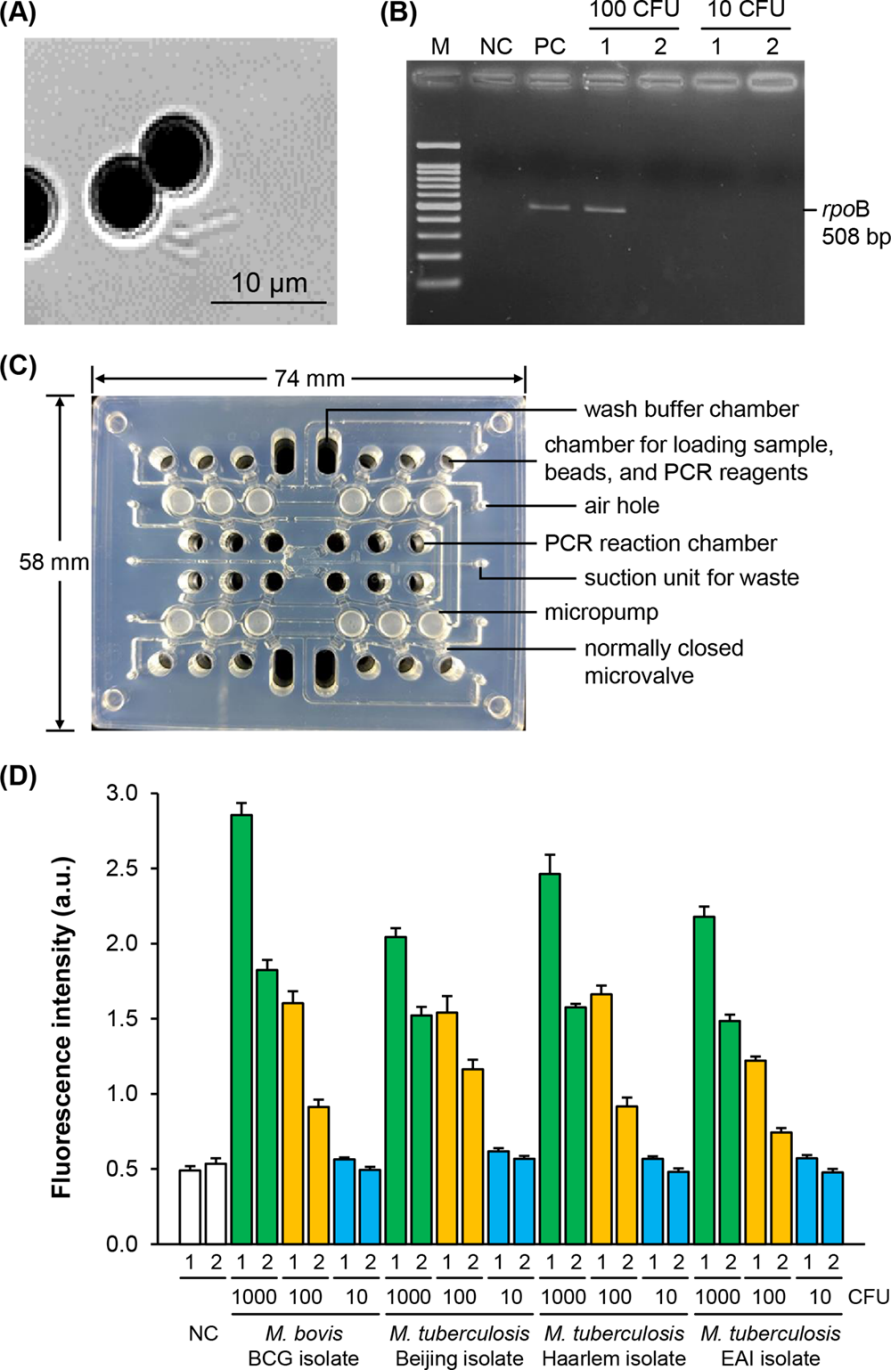
Use of the hexadecasaccharide **41**-conjugated magnetic beads in mycobacterial capture and detection. (A) **41**-Conjugated magnetic beads with captured *M. bovis* bacillus Calmette–Guérin (BCG) imaged under bright field microscopy (1000×). (B) Gel electrophoresis of live or dead *M. bovis* BCG captured by the **41**-conjugated beads treated with PMA followed by thermolysis and PCR of genomic DNA (gDNA) using *rpo*B primers. Lane M is a100 base-pair (bp) DNA ladder; NC is negative control using only double distilled water mixed with PCR reagents, PC is positive control with 100 colony-forming units (CFU) of BCG gDNA. Lanes 1 and 2 indicate live and dead captured BCG, respectively. (C) Integrated microfluidic chip used in the capture and detection experiments. (D) Fluorescence intensity measurements with *M. bovis* BCG and three isolates of *M. tuberculosis* on the integrated microfluidic system. Numbers 1 and 2 indicate fluorescence obtained when gDNA is amplified from the whole bacterial population and only from bacteria captured by **41**-conjugated beads, respectively. Error bars represent standard deviation (*n* = 10). a.u., arbitrary units; EAI, East African-Indian.

We next employed the integrated microfluidic chip with system design and operation as described previously.^49,63^ The chip has 24 reaction chambers with automated micropumps and micromixers that could carry out mycobacterial capture, genomic DNA release, and on-chip PCR (Figure 9C). It took 10 min for bacterial capture, and the whole process, including the laser-induced fluorescence measurement, was accomplished within 90 min on a single chip. Of the three bacterial counts employed for *M. bovis* BCG, the fluorescence for 10 CFU was comparable to that of the negative control (water), and the signals were marked higher for 100 CFU and 1000 CFU (Figure 9D). The data from samples with ≥100 CFU indicated the ability of hexadecasaccharide **41**-magnetic bead complexes to capture the mycobacteria while leaving some uncaptured portions in the supernatant. A comparable pattern occurred when three *Mtb* isolates were tested on this integrated microfluidic chip. These observations suggest a saturation effect, wherein the binding site of hexadecasaccharide **41** bound to the beads becomes fully occupied, preventing additional interactions with the remaining mycobacteria in the supernatant. This may be due, in part, to the multivalency of *Mtb*, which expresses multiple copies of HBHA on its surface, allowing a single bacterium to simultaneously engage with multiple HS chains. Such multivalent interactions could reduce the availability of free binding sites and exacerbate the saturation. Nevertheless, since the goal is to detect the presence of mycobacteria, not to exhaustively capture every bacterium, the ability to reliably detect as few as 100 colony-forming units (CFU) is considered sufficient for effective clinical monitoring.^64^

## Conclusions

The interaction between host HS and HBHA on the surface of *Mycobacterium tuberculosis* presents a compelling target for therapeutic intervention and diagnostic development, offering a promising strategy for the fight against tuberculosis. Working toward these possibilities, we successfully demonstrated the chemical preparation of eight HS-based hexadecasaccharides. A single 16-residue sugar precursor was rapidly achieved by convergent coupling of building blocks, which was then subjected to divergent transformations to generate compounds with varying patterns of sulfate, sulfamate, and acetamide groups found in the natural HS chains. Despite the extensive number of functionalities that need to be modified by each reagent application, the desired products were effectively acquired, as evidenced by NMR and mass analyses.

Our ITC binding assay with HBHA_110–199_ pointed to the synthetic HS hexadecasaccharide **24** with 6-*O*- and 2-*N*-sulfonated GlcN and 2-*O*-sulfonated IdoA as being the most suitable for further evaluation. CD and multidimensional NMR analysis indicated the affected sites in the binding between **24** and HBHA. In particular, the lysine-rich C-terminal region of HBHA showed its backbone atoms having notable shifts in the chemical environment when compound **24** is present in solution. The interaction also causes an increase in the α-helical content in the region of HBHA responsible for agglutination. Given such an understanding, we further manipulated the hexadecasaccharide precursor to include a biotin tag, allowing for attachment to a magnetic bead aimed at catching *Mtb* in samples via HBHA. PMA was used to separate the DNA of dead from live bacteria, and the conserved *rpo*B gene enabled selective DNA amplification by PCR. These processes were integrated into a microfluidic chip, and together with fluorescence detection of the amplified DNA bound to a green dye, detection of at least 100 CFU of mycobacteria was realized. Overall, the synthetic hexadecasaccharide offered a means to probe *Mtb* through HBHA and opened a possible diagnosis platform for the early detection of tuberculosis as well as the monitoring of patient responses to antibiotics and immunization.

## Supplementary Material



## Notes

Supplementary tables, supplementary figures, synthetic procedures, experimental procedures for binding assays and *Mtb* detection, NMR, mass, and IR spectra of key compounds (PDF)
