# Supplementary material for: Synthesis of Heparan Sulfate Hexadecasaccharides and Their Molecular Interaction with Mycobacterial Heparin-Binding Hemagglutinin for the Detection of Mycobacterium tuberculosis
Source: J Am Chem Soc. 2025 Dec 22;148(1):434–47. doi: 10.1021/jacs.5c14234 (PMC12814364; doi:10.1021/jacs.5c14234)
Supplement: Supplementary file 1 [file ja5c14234_si_001.pdf]

Supporting Information

Synthesis of Heparan Sulfate Hexadecasaccharides and their Molecular Interaction with Mycobacterial Heparin-Binding Hemagglutinin for the Detection of *Mycobacterium Tuberculosis*

Krishnagopal Maiti,[1] Guan-Wen Huang,[2] Yun-Hao Zhuang,[2] Chih-Hung Wang,[3] Jia-Ru Chang,[4] Medel Manuel L. Zulueta,[5] Jasper S. Dumalaog,[1,6] Chiao-Chu Ku,[1] Shih-Ching Wang,[1] Cheng-Hsiu Chang,[1] Chia-Lin Chyan,[2]* Horng-Yunn Dou,[4]* Gwo-Bin Lee,[3,7,8]* Shang-Cheng Hung[1,6,9,10]*

Affiliations:

[1] Genomics Research Center, Academia Sinica, Taipei 11529, Taiwan.

[2] Department of Chemistry, National Dong Hwa University, Hualien 97401, Taiwan.

[3] Department of Power Mechanical Engineering, National Tsing Hua University, Hsinchu 30013, Taiwan.

[4] Institute of Infectious Disease and Vaccinology, National Health Research Institutes, Miaoli 35053, Taiwan.

[5] Institute of Chemistry, College of Science, University of the Philippines, Diliman, Quezon City 1101, Philippines.

[6] Department of Chemistry, National Tsing Hua University, Hsinchu 30013, Taiwan.

[7] Institute of Nanoengineering and Microsystems, National Tsing Hua University, Hsinchu 30013, Taiwan.

[8] Institute of Biomedical Engineering, National Tsing Hua University, Hsinchu 30013, Taiwan.

[9] Department of Chemistry, National Cheng Kung University, Tainan 70101, Taiwan.

[10] Department of Applied Science, National Taitung University, Taitung 95092, Taiwan.

*E-mails:

Chia-Lin Chyan – chyan@gms.ndhu.edu.tw

Horng-Yunn Dou − 940719@nhri.edu.tw

Gwo-Bin Lee − gwobin@pme.nthu.edu.tw

Shang-Cheng Hung − schung@gate.sinica.edu.tw

Table of Contents

Supplementary Tables S3
Table S1 .... S3
Table S2 .... S4
Table S3 .... S5
Table S4 .... S6
Table S5 .... S7
Supplementary Figures S8
Figure S1 .... S8
Figure S2 .... S9
Figure S3 .... S10
Figure S4 .... S11
Synthetic Procedures S12
Experimental Procedures for Binding Assays and *Mtb* Detection S31
NMR, Mass, and IR Spectra of Key Compounds S35
References S170

Supplementary Tables

Table S1. Comparison of ^{13}C NMR chemical shifts of the nitrogen-bonded C2 from azido, amino, acetamido, and sulfonamido hexadecasaccharide derivatives.

***O*-Sulfonation**	**Compound**	**Functional Group**	**Chemical Shift (δ, in ppm)**	**Solvent**
Non-*O*-Sulfonated	**18**	Azido (N_3)	63.2–63.4	$CDCl_3$
	**19**	Amino (NH_2)	54.0–54.4	D_2O
	**20**	Acetamido (NHAc)	53.5–53.8	D_2O
	**21**	Sulfonamido ($NHSO_3^-$)	57.8–58.1	D_2O
2,6-Di-*O*-Sulfonated	**18**	Azido (N_3)	63.2–63.4	$CDCl_3$
	**22**	Amino (NH_2)	54.0–54.2	D_2O
	**23**	Acetamido (NHAc)	53.2–53.8	D_2O
	**24**	Sulfonamido ($NHSO_3^-$)	57.4–57.9	D_2O
2-*O*-Sulfonated	**18**	Azido (N_3)	63.2–63.4	$CDCl_3$
	**25**	Amino (NH_2)	54.2–54.5	D_2O
	**26**	Acetamido (NHAc)	53.3–54.0	D_2O
	**27**	Sulfonamido ($NHSO_3^-$)	58.0–58.3	D_2O
6-*O*-Sulfonated	**28**	Azido (N_3)	63.9–63.4	$CDCl_3$
	**30**	Amino (NH_2)	53.8–54.2	D_2O
	**31**	Acetamido (NHAc)	53.4–54.3	D_2O
	**32**	Sulfonamido ($NHSO_3^-$)	57.7–57.9	D_2O

Table S2. Dissociation constants obtained from tracking the movement of the NMR cross peaks of key residues of $HBHA_{110–199}$ upon titration with compound **24**.

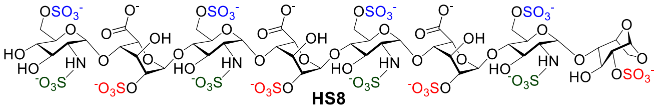

HS8

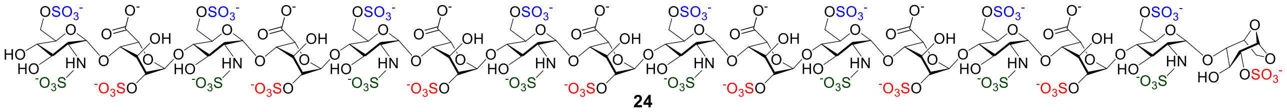

24

	Residue	HS8[1]	Compound 24
**Repeat I**	K161	9.8	12.9
**P(KKAA)**	K162	45.3	6.8
**Residue 160-164**	A163	20.7	9.5
	A164	22.2	5.4
	**Average**	**24.5**	**8.7**
**Repeat II**	A166	14.4	3.2
**P(AKKAA)**	K167	4.2	1.7
**Residue 165-170**	K168	17.2	3.2
	A169	14.6	5.5
	A170	8.3	2.5
	**Average**	**11.8**	**3.2**
**Repeat III**	A172	10.3	2.2
**P(AKKAA)**	K173	4.2	1.7
**Residue 171-176**	K174	6.6	6.7
	A175	8.5	2.3
	A176	5.0	1.4
	**Average**	**6.9**	**2.9**
**Repeat IV**	A178	4.8	0.8
**P(AKKAAAKKA)**	K179	6.6	1.3
**Residue 177-186**	K180	4.1	0.8
	A181	4.8	1.4
	A182	4.6	1.0
	A183	4.6	0.7
	K184	18.3	3.3
	K185	4.5	1.0
	A186	3.1	0.8
	**Average**	**6.2**	**1.4**
**Repeat V**	A188	4.8	0.8
**P(AKKAAAKKV)**	K189	5.9	1.2
**Residue 187-196**	K190	4.1	1.2
	A191	5.3	1.5
	A192	4.6	2.1
	A193	4.8	1.0
	K194	4.9	1.1
	K195	4.6	2.9
	V196	2.7	2.3
	**Average**	**4.6**	**1.6**

Table S3. ^{1}H and ^{13}C resonance assignments for compound **24**.

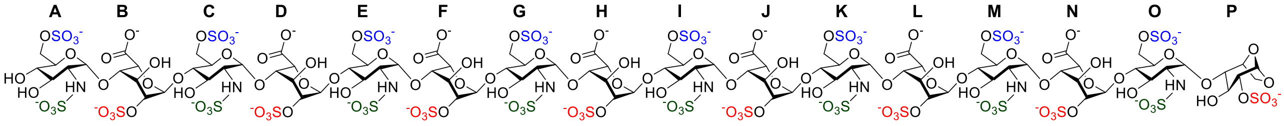

24

Residue	H1	C1	H2	C2	H3	C3	H4	C4	H5	C5	H6a	H6b	C6
**A**	5.408	99.405	3.197	60.733	3.613	73.736	3.533	71.824	3.963	72.552	4.338	4.179	69.062
**B**	5.184	102.07	4.303	78.711	4.172	71.882	4.070	78.619	4.810	72.111	-	-	-
**C**	5.407	99.324	3.242	60.737	3.619	72.292	3.752	78.361	3.985	72.020	4.399	4.240	69.050
**D**	5.184	102.07	4.303	78.711	4.172	71.882	4.070	78.619	4.810	72.111	-	-	-
**E**	5.407	99.324	3.242	60.737	3.619	72.292	3.752	78.361	3.985	72.020	4.399	4.240	69.050
**F**	5.184	102.07	4.303	78.711	4.172	71.882	4.070	78.619	4.810	72.111	-	-	-
**G**	5.407	99.324	3.242	60.737	3.619	72.292	3.752	78.361	3.985	72.020	4.399	4.240	69.050
**H**	5.184	102.07	4.303	78.711	4.172	71.882	4.070	78.619	4.810	72.111	-	-	-
**I**	5.407	99.324	3.242	60.737	3.619	72.292	3.752	78.361	3.985	72.020	4.399	4.240	69.050
**J**	5.184	102.07	4.303	78.711	4.172	71.882	4.070	78.619	4.810	72.111	-	-	-
**K**	5.407	99.324	3.242	60.737	3.619	72.292	3.752	78.361	3.985	72.020	4.399	4.240	69.050
**L**	5.184	102.07	4.303	78.711	4.172	71.882	4.070	78.619	4.810	72.111	-	-	-
**M**	5.376	99.253	3.244	60.730	3.620	72.287	3.752	78.361	3.985	72.020	4.408	4.229	69.043
**N**	5.165	102.15	4.263	78.529	4.170	71.902	4.057	78.584	4.761	71.972	-	-	-
**O**	5.386	101.970	3.278	60.694	3.631	72.283	3.683	79.927	3.905	72.319	4.334	4.259	69.516
**P**	5.662	101.74	4.132	82.827	3.839	73.290	3.847	82.984	4.854	76.794	4.144	3.811	68.170

Table S4. Coupling constants and conformation of sugar residues of compound **24** deduced from DQF–COSY.

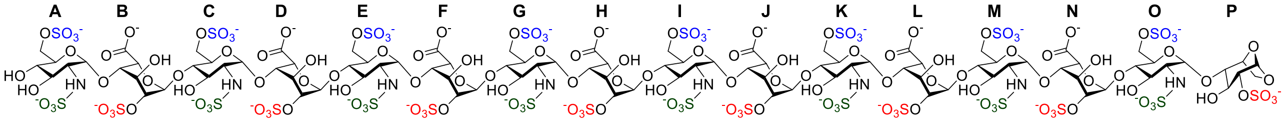

24

Residue	Monomer type	^3J coupling constant of proton pair (in Hz)							Conformation
		H1–H2	H2–H3	H3–H4	H4–H5	H5–H6a	H5–H6b	H6a–H6b	
**Free Form**									
**A**	GlcNS6S	3.8	10.7	8.3	10.3	3.5	3.4	10.9	$^4C_1$
**B**	IdoA2S	3.3	6.5	3.3	3.3	-	-	-	$^2S_O/^1C_4$ [a]
**C**	GlcNS6S	4.3	10.7	8.3	10.7	4.3	3.7	12.2	$^4C_1$
**D**	IdoA2S	3.3	6.5	3.3	3.3	-	-	-	$^2S_O/^1C_4$ [a]
**E**	GlcNS6S	4.3	10.7	8.3	10.7	4.3	3.7	12.2	$^4C_1$
**F**	IdoA2S	3.3	6.5	3.3	3.3	-	-	-	$^2S_O/^1C_4$ [a]
**G**	GlcNS6S	4.3	10.7	8.3	10.7	4.3	3.7	12.2	$^4C_1$
**H**	IdoA2S	3.3	6.5	3.3	3.3	-	-	-	$^2S_O/^1C_4$ [a]
**I**	GlcNS6S	4.3	10.7	8.3	10.7	4.3	3.7	12.2	$^4C_1$
**J**	IdoA2S	3.3	6.5	3.3	3.3	-	-	-	$^2S_O/^1C_4$ [a]
**K**	GlcNS6S	4.3	10.7	8.3	10.7	4.3	3.7	12.2	$^4C_1$
**L**	IdoA2S	3.3	6.5	3.3	3.3	-	-	-	$^2S_O/^1C_4$ [a]
**M**	GlcNS6S	4.1	10.8	8.3	10.7	4.3	3.7	12.2	$^4C_1$
**N**	IdoA2S	3.1	6.2	3.3	3.3	-	-	-	$^2S_O/^1C_4$ [a]
**O**	GlcNS6S	3.8	10.5	8.4	10.3	3.0	5.5	11.5	$^4C_1$
**P**	1,6 anhydro-IdoA2S	2.7	7.2	8.6	3.5	1.1	5.4	8.5	$^4C_1$
**Bound form ([24]:[HBHA] = 20:1)**									
**A**	GlcNS6S	3.7	10.9	8.4	10.5	3.5	3.5	10.8	$^4C_1$
**B**	IdoA2S	3.3	6.7	3.5	4.4	-	-	-	$^2S_O/^1C_4$ [b]
**C**	GlcNS6S	4.0	10.8	8.0	10.6	4.1	3.9	12.1	$^4C_1$
**D**	IdoA2S	3.3	6.7	3.5	4.4	-	-	-	$^2S_O/^1C_4$ [b]
**E**	GlcNS6S	4.0	10.8	8.0	10.6	4.1	3.9	12.1	$^4C_1$
**F**	IdoA2S	3.3	6.7	3.5	4.4	-	-	-	$^2S_O/^1C_4$ [b]
**G**	GlcNS6S	4.0	10.8	8.0	10.6	4.1	3.9	12.1	$^4C_1$
**H**	IdoA2S	3.3	6.7	3.5	4.4	-	-	-	$^2S_O/^1C_4$ [b]
**I**	GlcNS6S	4.0	10.8	8.0	10.6	4.1	3.9	12.1	$^4C_1$
**J**	IdoA2S	3.3	6.7	3.5	4.4	-	-	-	$^2S_O/^1C_4$ [b]
**K**	GlcNS6S	4.0	10.8	8.0	10.6	4.1	3.9	12.1	$^4C_1$
**L**	IdoA2S	3.3	6.7	3.5	4.4	-	-	-	$^2S_O/^1C_4$ [b]
**M**	GlcNS6S	4.0	10.8	8.0	10.6	4.1	3.9	12.1	$^4C_1$
**N**	IdoA2S	3.3	6.3	3.4	3.6	-	-	-	$^2S_O/^1C_4$ [b]
**O**	GlcNS6S	4.1	10.4	8.4	10.6	3.0	5.4	11.3	$^4C_1$
**P**	1,6 anhydro-IdoA2S	2.5	7.4	8.8	3.5	1.2	5.4	8.5	$^4C_1$

[a] Population ratio of ^2S_O to $^1C_4$ conformation of IdoA2S is estimated to around 2:1 according to previous studies.[2,3]

[b] Population of IdoA2S largely shifted to ^2S_O as suggested by an increase of ^3J upon addition of 5% $HBHA_{110–199}$.[4,5]

Table S5. Thermodynamic parameters of the binding of the synthesized HS hexadecasaccharides and **HS8** with HBHA obtained by ITC.

Sugar	K_D (μM)[a]	n[b]	ΔG (kcal/mol)	ΔH (kcal/mol)	–TΔS (kcal/mol)
**20**	–	–	–	–	–
**21**	16.0 ± 1.5	0.23 ± 0.10	–6.55 ± 0.06	7.76 ± 4.02	–14.30 ± 3.96
**23**	2.63 ± 0.11	0.27 ± 0.01	–7.62 ± 0.02	15.00 ± 0.00	–22.60 ± 0.00
**24**	1.87 ± 0.06	0.27 ± 0.01	–7.82 ± 0.02	16.00 ± 0.00	–23.85 ± 0.08
**26**	–	–	–	–	–
**27**	–	–	–	–	–
**31**	–	–	–	–	–
**32**	2.36 ± 0.30	0.30 ± 0.01	–7.69 ± 0.08	11.50 ± 0.28	–19.15 ± 0.21
**HS8**	2.40 ± 0.30	0.56 ± 0.01	–7.68 ± 0.08	5.38 ± 0.05	–13.05 ± 0.07

[a] ITC measurements were performed in two trials, with results presented as the average ± standard deviation.

[b] Sugar-to-protein binding ratio.

Supplementary Figures

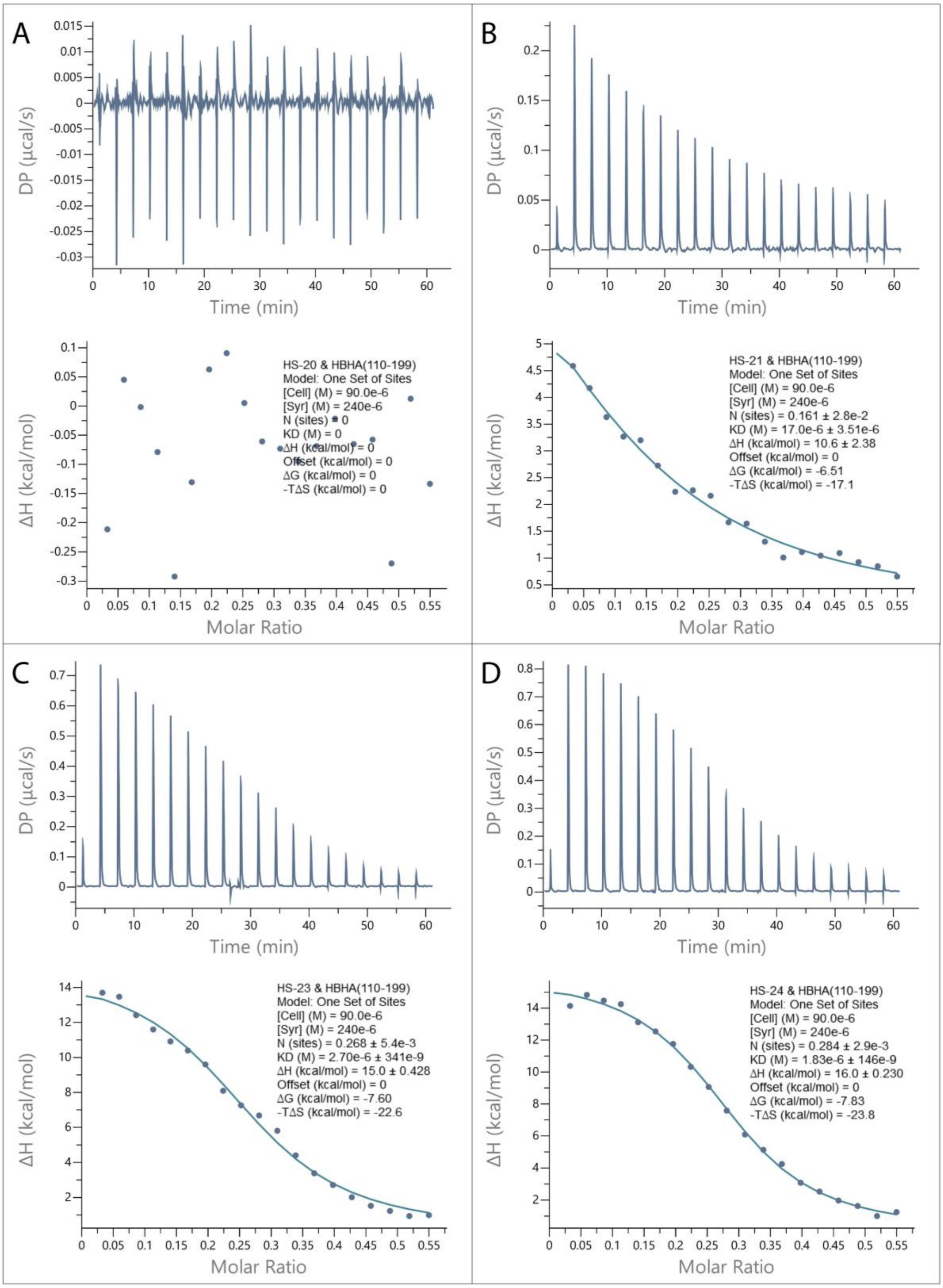

Figure S1. Representative titration isotherms of $HBHA_{110–199}$ by the synthesized heparan sulfate hexadecasaccharides. Panels **A–D** correspond to isotherms with compounds **20 (A)**, **21 (B)**, **23 (C)**, and **24 (D)**, respectively. The upper plots display the raw titration data (corrected for the heat of dilution), while the lower plots show the corresponding integrated heats of binding derived from the raw data.

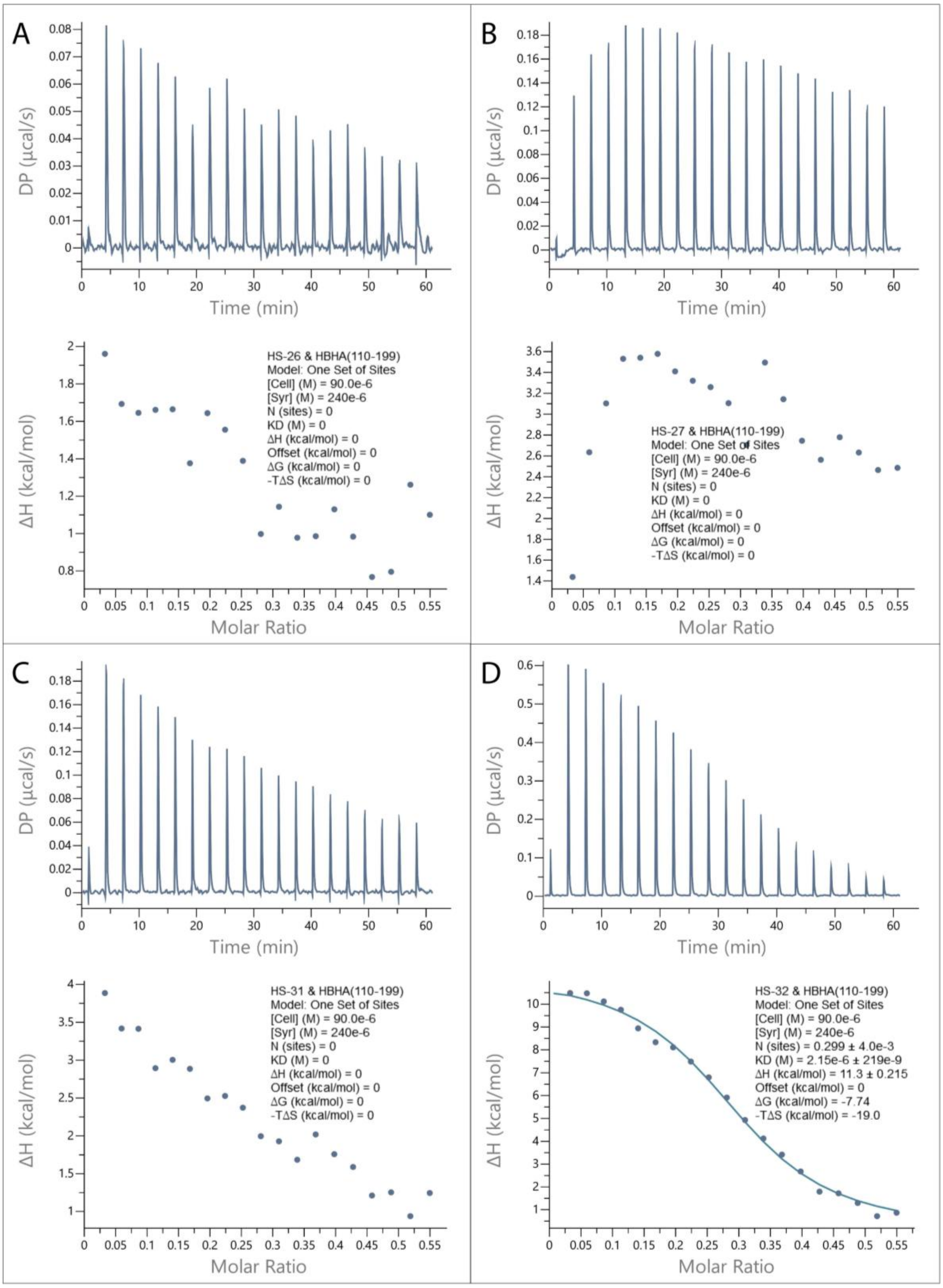

Figure S2. Representative titration isotherms of $HBHA_{110–199}$ by the synthesized heparan sulfate hexadecasaccharides. Panels **A–D** correspond to isotherms with compounds **26 (A)**, **27 (B)**, **31 (C)**, and **32 (D)**, respectively. The upper plots display the raw titration data (corrected for the heat of dilution), while the lower plots show the corresponding integrated heats of binding derived from the raw data.

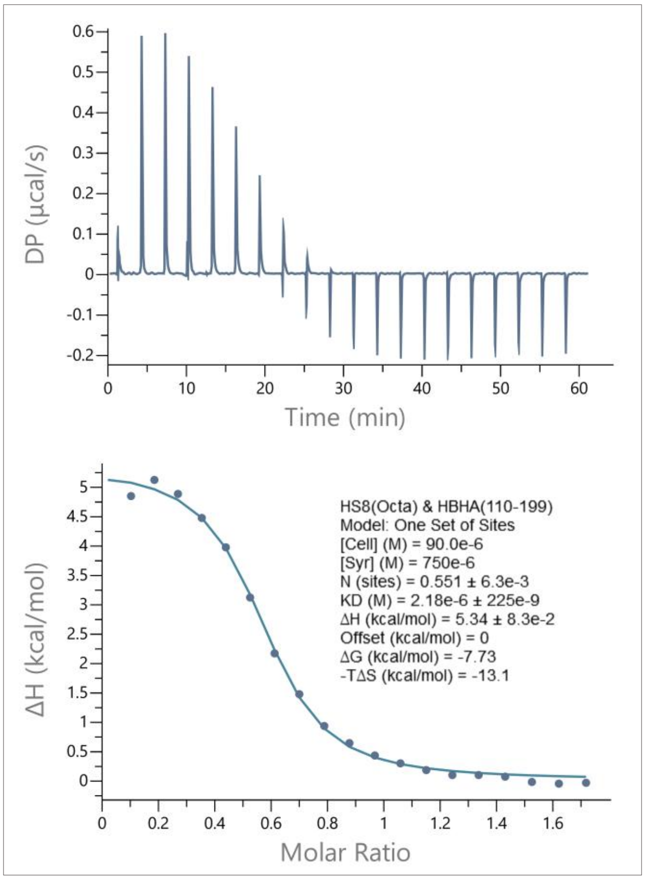

Figure S3. Representative titration isotherms of $HBHA_{110–199}$ by the synthetic heparan sulfate octasaccharide **HS8**. The upper plots display the raw titration data (corrected for the heat of dilution), while the lower plots show the corresponding integrated heats of binding derived from the raw data.

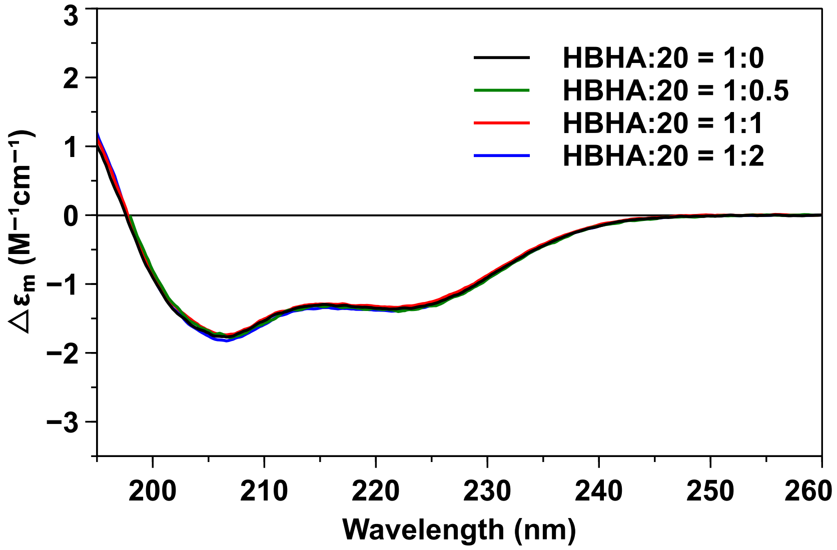

Figure S4. Far-ultraviolet CD spectra of full-length HBHA upon association with compound **20** in varying molar ratios (negative control).

Synthetic Procedures

General considerations. CH_2Cl_2 and THF were purified and dried from a safe purification system filled with anhydrous Al_2O_3. All other reagents were obtained from commercial sources and used without further purification. Water was either distilled or Milli-Q-purified. All the air or moisture sensitive reactions were carried out under a nitrogen atmosphere with anhydrous solvents. Prior to all the glycosylation and one-pot reactions, the starting materials were dried under high vacuum overnight in a desiccator. Flash column chromatography was carried out on Silica Gel 60 (230–400 mesh, 100–200 mesh, E. Merck). TLC was performed on glass plates pre-coated with Silica Gel 60 F_{254} (0.25 mm, E. Merck); detection was executed by spraying with a solution of $Ce(NH_4)_2(NO_3)_6$, $(NH_4)_6Mo_7O_{24}$, and H_2SO_4 in water and subsequent heating on a hot plate. Samples for IR analysis are deposited as thin films in KBr plates. Specific rotations were taken at ambient conditions by using 589 nm (sodium D line) and reported in 10^{-1} deg·cm^2·g^{-1}; the sample concentrations were in g·dL^{-1}. ^{1}H and ^{13}C NMR spectra were recorded on 600 MHz spectrometers. Chemical shifts are in ppm from Me_4Si calibrated using the resonances of the carbon and the residual proton of the deuterated solvent. Proton peaks were assigned with the aid of 2D NMR techniques (^{1}H–^{1}H COSY, ^{13}C–^{1}H HSQC, NOESY, and HMBC). The hydrogen multiplicities of carbon peaks were determined using DEPT-90 and DEPT-135 experiments, the spectra of which were herein provided together with the power-gated-decoupled ^{13}C NMR spectrum.

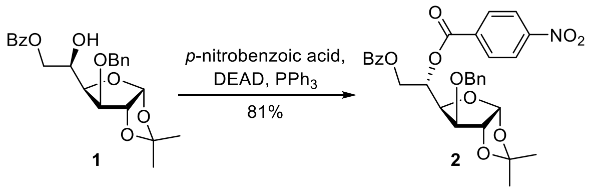

6-*O*-Benzoyl-3-*O*-benzyl-1,2-*O*-isopropylidene-5-*O*-(4-nitrobenzoyl)-*β*-L-idofuranose (2). *p*-Nitrobenzoic acid (2.06 g, 12.3 mmol) and triphenylphosphine (PPh_3, 3.23 g, 12.3 mmol) were added to a solution of the α-D-glucofuranose derivative **1** (510 mg, 1.23 mmol) in dry toluene (10 mL). Then diethyl azodicarboxylate (DEAD, 1.93 mL, 12.3 mmol) was added to the solution and the reaction mixture was heated at 80 °C for 16 h. The reaction mixture was cooled down, diluted with ethyl acetate (100 mL), washed with $NaHCO_{3(aq)}$ and water, dried over anhydrous $MgSO_4$, filtered, and concentrated *in vacuo*. The residue was purified by column chromatography (ethyl acetate/hexane = 1/5, v/v) on silica gel to provide compound **2** (561 mg, 81%). $[\alpha]_D^{26}$ –4.50 (*c* 0.1, $CHCl_3$); IR (thin film) *ν* 2917.6, 2850.5, 1810.5, 1727.7, 1602.5, 1527.1, 1453.4, 1374.8, 1346.7, 1282.0, 1261.8, 1098.2, 1074.4, 1024.6 cm^{-1}; ^{1}H NMR (600 MHz, $CDCl_3$) δ 8.22 (d, *J* = 8.5 Hz, 2H, Ph-H), 8.14 (d, *J* = 8.5 Hz, 2H, Ph-H), 7.97 (d, *J* = 7.8 Hz, 2H, Ph-H), 7.56 (t, *J* = 7.4 Hz, 1H, Ph-H), 7.42 (t, *J* = 7.6 Hz, 2H, Ph-H), 7.35 – 7.27 (m, 5H, Ph-H), 6.01 (d, *J* = 3.8 Hz, 1H, H-1), 5.87 (ddd, *J* = 8.2, 5.6, 2.6 Hz, 1H, H-5), 4.73 (d, *J* = 11.6 Hz, 1H, $PhCH_2$), 4.70 (d, *J* = 3.9 Hz, 1H, H-2), 4.60 (dd, *J* = 12.4, 2.7 Hz, 1H, H-6a), 4.57 (dd, *J* = 8.0, 3.7 Hz, 1H, H-4), 4.50 (d, *J* = 11.6 Hz, 1H, $PhCH_2$), 4.46 (dd, *J* = 12.4, 5.7 Hz, 1H, H-6b), 4.10 (d, *J* = 3.5 Hz, 1H, H-3), 1.52 (s, 3H, CH_3), 1.34 (s, 3H, CH_3); ^{13}C NMR (150 MHz, $CDCl_3$) δ 166.1, 164.2, 150.6, 136.6, 135.6, 133.5, 131.1, 129.8, 129.6, 128.8, 128.7, 128.5, 128.2, 123.5, 112.1, 105.2 (C-1), 82.0 (C-3), 81.9 (C-2), 78.4 (C-4), 72.0 (Ph*C*H_2), 71.9 (C-5), 63.4 (C-6), 26.9, 26.4; HRMS (ESI): *m/z* calcd for $C_{30}H_{30}O_{10}N$ $[M + H]^+$: 564.1864, found: 564.1839.

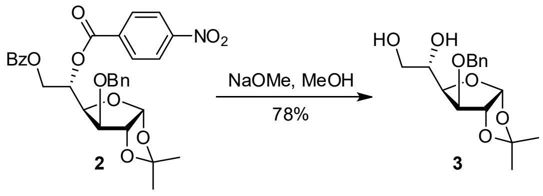

3-*O*-Benzyl-1,2-*O*-isopropylidene-*β*-L-idofuranose (3). Compound **2** (550 mg, 0.976 mmol) was dissolved in THF (2.20 mL) and NaOMe in MeOH (1 M, 1.10 mL) was added to it, and the resulting mixture was stirred for 1 h at room temperature. The reaction mixture was neutralized with Amberlite ion exchange resin (H^+), filtered, and concentrated in *vacuo.* The residue was purified by flash column chromatography (ethyl acetate/hexane = 1/2, v/v) on silica gel to provide the diol **3** (236 mg, 78%). $[\alpha]_D^{26}$ –51.5 (*c* 0.1, $CHCl_3$); IR (thin film) ν 3430, 2986, 2934, 1637, 1456, 1376, 1256, 1215, 1165, 1115, 1073, 1024 cm^{-1}; ^1H NMR (600 MHz, $CDCl_3$) δ 7.38–7.29 (m, 5H, Ph-H), 5.99 (d, *J* = 3.8 Hz, 1H, H-1), 4.72 (d, *J* = 11.7 Hz, 1H, $PhCH_2$), 4.65 (d, *J* = 3.9 Hz, 1H, H-2), 4.48 (d, *J* = 11.8 Hz, 1H, $PhCH_2$), 4.20 (dd, *J* = 5.3, 3.6 Hz, 1H, H-4), 4.09 – 4.05 (m, 1H, H-5), 4.02 (d, *J* = 3.5 Hz, 1H, H-3), 3.64 (dd, *J* = 11.4, 4.0 Hz, 1H, H-6a), 3.59 (dd, *J* = 11.5, 5.8 Hz, 1H, H-6b), 3.15 (br s, 1H, OH), 2.33 (br s, 1H, OH), 1.48 (s, 3H, CH_3), 1.33 (s, 3H, CH_3); ^{13}C NMR (150 MHz, $CDCl_3$) δ 136.6, 128.7, 128.4, 128.0, 112.0, 104.9 (C-1), 82.8 (C-3), 82.2 (C-2), 79.7 (C-4), 71.9 (Ph*C*H_2), 70.5 (C-5), 63.6 (C-6), 26.8, 26.3; HRMS (ESI): *m/z* calcd for $C_{16}H_{22}O_6Na$ $[M + Na]^+$: 333.1309, found: 333.1304.

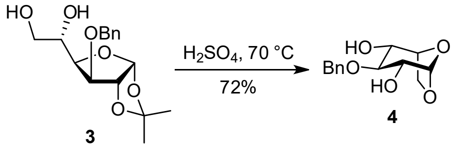

1,6-Anhydro-3-*O*-benzyl-*β*-L-idopyranose (4). Compound **3** (230 mg, 0.741 mmol) was dissolved in DMSO (2.30 mL) and 4.5 M H_2SO_4 (1.15 mL) was added to it, and the solution was heated to 70 °C for 16 h. After cooling the mixture down, the target material was carefully extracted with ethyl acetate (100 mL). The combined organic layer was washed with $NaHCO_{3(aq)}$ and water, dried over anhydrous $MgSO_4$, filtered, and concentrated *in vacuo*. The residue was purified by column chromatography (ethyl acetate/hexane = 1/2, v/v) on silica gel to provide anhydro-L-idose **4**[6] (135 mg, 72%). ^1H NMR (600 MHz, $CDCl_3$) δ 7.37 (d, *J* = 4.4 Hz, 4H, Ph-H), 7.34–7.30 (m, 1H, Ph-H), 5.29 (s, 1H, H-1), 4.95 (d, *J* = 11.7 Hz, 1H, $PhCH_2$), 4.74 (d, *J* = 11.8 Hz, 1H, $PhCH_2$), 4.43 (t, *J* = 4.7 Hz, 1H, H-5), 4.03 (d, *J* = 7.8 Hz, 1H, H-6a), 3.87 (dd, *J* = 8.2, 4.2 Hz, 1H, H-4), 3.72 (dd, *J* = 7.8, 5.1 Hz, 1H, H-6b), 3.65 (d, *J* = 7.6 Hz, 1H, H-2), 3.39 (t, *J* = 7.9 Hz, 1H, H-3), 2.19 (br s, 1H, OH), 1.99 (br s, 1H, OH); ^{13}C NMR (150 MHz, $CDCl_3$) δ 138.5, 128.8, 128.2, 128.1, 102.0 (C-1), 84.3 (C-3), 75.6 (C-2), 75.1 (C-5), 74.7 (Ph*C*H_2), 71.2 (C-4), 65.2 (C-6); HRMS (ESI): *m/z* calcd for $C_{13}H_{16}O_5Na$ $[M + Na]^+$: 275.0890, found: 275.0895.

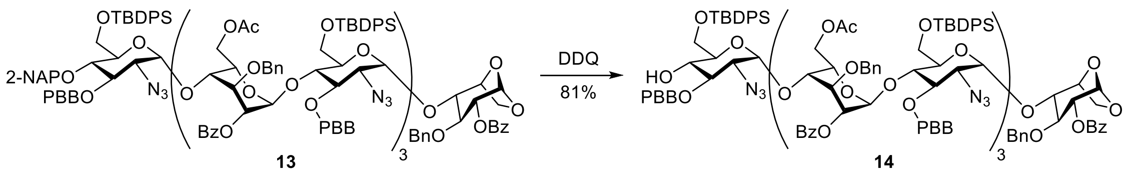

Compound 14. To a solution of the octasaccharide derivative **13**[7] (2.75 g, 0.676 mmol) in a mixed solvent of CH_2Cl_2 and water [18/1 (v/v), 138 mL], 2,3-dichloro-5,6-dicyano-1,4-benzoquinone (DDQ, 460 mg, 2.03 mmol) was added at 0 °C and stirred for 16 h at 4 °C. The reaction was filtered through a Celite pad and filtrate was extracted with CH_2Cl_2 (3 × 50 mL). The organic layers were consecutively washed with saturated $NaHCO_{3(aq)}$ and water, dried over anhydrous

$MgSO_4$, filtered, and concentrated *in vacuo*. The residue was purified by flash column chromatography (ethyl acetate/hexane = 1/3, v/v) on silica gel to provide the 4-alcohol **14** (2.15 g, 81%). $[\alpha]_D^{26}$ +23.0 (*c* 0.1, $CHCl_3$); IR (thin film) *ν* 3491, 3070, 2931, 2858, 2109, 1743, 1721, 1601, 1489, 1453, 1428, 1390, 1366, 1317, 1268, 1112, 1070, 1043, 1027 cm^{-1}; ^1H NMR (600 MHz, $CDCl_3$) δ 8.06–8.02 (m, 3H), 8.02–7.98 (m, 5H), 7.67–7.62 (m, 11H), 7.61–7.58 (m, 8H), 7.49–7.40 (m, 10H), 7.40–7.35 (m, 8H), 7.35–7.26 (m, 33H), 7.25–7.16 (m, 17H), 7.12–7.08 (m, 4H), 6.92 (dd, *J* = 10.6, 8.3 Hz, 4H), 5.50 (d, *J* = 1.8 Hz, 1H), 5.42 (s, 1H), 5.39 (d, *J* = 2.0 Hz, 1H), 5.29 (d, *J* = 2.5 Hz, 1H), 5.16 (d, *J* = 3.4 Hz, 4H), 5.03 (dd, *J* = 8.2, 1.9 Hz, 1H), 4.91 (d, *J* = 11.4 Hz, 1H), 4.85 (d, *J* = 10.9 Hz, 1H), 4.81 (dd, *J* = 14.0, 11.2 Hz, 2H), 4.74 (dd, *J* = 15.2, 11.0 Hz, 2H), 4.68 (d, *J* = 11.3 Hz, 1H), 4.65–4.61 (m, 4H), 4.59 (t, *J* = 4.4 Hz, 2H), 4.58–4.53 (m, 2H), 4.47 (d, *J* = 11.1 Hz, 1H), 4.34–4.31 (m, 3H), 4.27 (td, *J* = 6.6, 2.6 Hz, 1H), 4.17 (d, *J* = 11.1 Hz, 1H), 4.15–4.08 (m, 4H), 4.07–4.00 (m, 8H), 3.99–3.95 (m, 4H), 3.92–3.85 (m, 6H), 3.76 (d, *J* = 9.7 Hz, 1H), 3.74–3.73 (m, 1H), 3.72–3.68 (m, 4H), 3.63–3.57 (m, 2H), 3.54 (dd, *J* = 9.6, 2.3 Hz, 2H), 3.53–3.47 (m, 5H), 3.39 (t, *J* = 3.0 Hz, 1H), 3.30 (dd, *J* = 10.3, 3.9 Hz, 1H), 3.19–3.12 (m, 3H), 1.77 (s, 3H), 1.71 (s, 3H), 1.64 (s, 3H), 1.05 (s, 9H), 1.01 (s, 18H), 1.00 (s, 9H); ^{13}C NMR (150 MHz, $CDCl_3$) δ 170.2, 169.9, 169.9, 165.7, 165.7, 165.7, 165.6, 137.9, 137.3, 137.1, 137.0, 137.0, 136.0, 135.9, 135.7, 135.6, 135.6, 135.6, 133.4, 133.2, 133.2, 133.1, 132.9, 132.8, 131.5, 131.2, 131.0, 131.0, 130.0, 130.0, 129.9, 129.9, 129.8, 129.8, 129.7, 129.7, 129.6, 129.6, 129.4, 129.4, 129.3, 129.0, 129.0, 128.7, 128.5, 128.5, 128.5, 128.5, 128.4, 128.4, 128.4, 128.2, 128.2, 127.9, 127.8, 127.8, 127.7, 127.6, 127.6, 127.6, 127.5, 121.7, 121.4, 121.1, 121.0, 99.2, 98.2, 97.5, 97.2, 97.1, 97.0, 96.9, 80.0, 79.3, 78.9, 78.9, 78.5, 77.0, 75.0, 74.3, 74.1, 74.1, 73.9, 73.6, 73.1, 73.0, 73.0, 72.8, 72.7, 72.5, 72.3, 72.2, 71.9, 69.2, 68.8, 68.6, 65.9, 65.5, 65.0, 64.8, 64.2, 64.1, 63.8, 63.7, 63.1, 62.3, 62.2, 62.1, 26.9, 26.8, 26.8, 26.8, 20.7, 20.6, 20.6, 19.5, 19.4, 19.3, 19.3; HRMS (MALDI): *m/z* calcd for $C_{202}H_{214}O_{43}N_{12}Br_4Si_4Na$ $[M + Na]^+$: 3952.9326, found: 3952.9307.

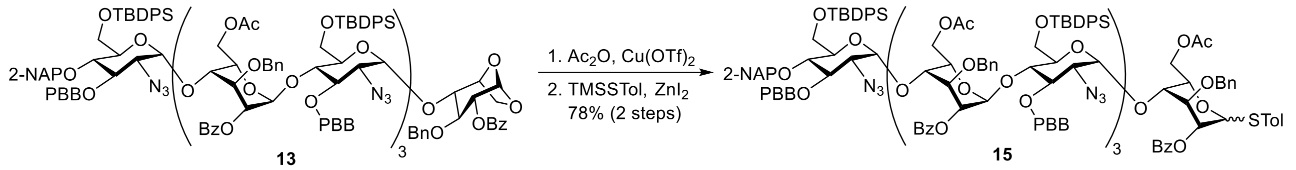

13 **15**

Compound 15. The octasaccharide derivative **13** (2.56 g, 0.629 mmol) was dissolved in acetic anhydride (Ac_2O, 7.68 mL) under a nitrogen atmosphere, the reaction flask was immersed into an ice bath, and copper(II) triflate ($Cu(OTf)_2$, 22.7 mg, 0.063 mol) was added to the solution. After stirring for 18 h, the reaction was quenched by saturated $NaHCO_{3(aq)}$ solution, and the mixture was extracted with CH_2Cl_2 (3 × 50 mL). The combined organic layers were washed with brine, dried over anhydrous $MgSO_4$, filtered, and concentrated *in vacuo*. The residue was co-evaporated with toluene (3 × 5 mL) and dried completely under high vacuum.

Trimethylsilyl *p*-toluenyl thioether (TMSSTol, 355 μL, density = 1.00 g/mL, 1.81 mmol) and ZnI_2 (578 mg, 1.81 mmol) were added to a solution of the crude compound (2.52 g, 0.604 mmol) in anhydrous CH_2Cl_2 (25 mL) at ambient temperature under a nitrogen atmosphere. After 6 h, the resulting mixture was filtered through a pad of Celite, and the filtrate was diluted with CH_2Cl_2 (100 mL) and washed with $Na_2S_2O_{3(aq)}$ solution and saturated solution of $NaHCO_{3(aq)}$, dried over anhydrous $MgSO_4$ and filtered. The resulting solution was concentrated *in vacuo* and purified by silica gel chromatography (ethyl acetate/hexane = 1/5) to afford the product **15** (2.07 g, 78%). $[\alpha]_D^{26}$ –57.0 (*c* 0.1, $CHCl_3$); IR (thin film) *ν* 3031, 2931, 2858, 2109, 1743, 1721, 1601, 1489, 1453, 1428, 1368, 1317, 1267, 1241, 1155, 1112, 1070, 1042 cm^{-1}; ^1H NMR (600 MHz, $CDCl_3$) δ 8.12–8.07 (m, 2H), 8.02–7.95 (m, 6H), 7.91–7.83 (m, 1H), 7.79–7.73 (m, 1H), 7.68–7.54 (m, 17H), 7.54–7.47 (m, 2H), 7.47–7.44 (m, 4H), 7.43–7.26 (m, 44H), 7.25–7.06 (m, 23H), 7.01–6.98 (m, 1H), 6.92–

6.88 (m, 4H), 6.86–6.82 (m, 3H), 5.52 (s, 1H), 5.43–5.36 (m, 2H), 5.36–5.31 (m, 2H), 5.16–5.10 (m, 3H), 4.95 (d, *J* = 11.6 Hz, 1H), 4.86–4.82 (m, 3H), 4.79 (dd, *J* = 11.2, 3.0 Hz, 2H), 4.72–4.68 (m, 2H), 4.66–4.57 (m, 5H), 4.55 (d, *J* = 3.8 Hz, 1H), 4.51–4.49 (m, 2H), 4.44 (d, *J* = 3.9 Hz, 1H), 4.36–4.28 (m, 3H), 4.28–4.20 (m, 2H), 4.16–3.92 (m, 17H), 3.92–3.78 (m, 7H), 3.78–3.74 (m, 2H), 3.72–3.61 (m, 4H), 3.59 (dd, *J* = 9.8, 2.6 Hz, 1H), 3.54–3.42 (m, 7H), 3.39–3.35 (m, 2H), 3.29–3.21 (m, 1H), 3.18 (dd, *J* = 10.2, 3.8 Hz, 1H), 3.14 (dd, *J* = 10.3, 3.7 Hz, 2H), 2.33 (s, 3H), 1.87 (s, 3H), 1.69 (s, 3H), 1.63 (s, 6H), 1.06 (s, 9H), 1.00 (s, 9H), 1.00 (s, 9H), 0.98 (s, 9H); ^{13}C NMR (150 MHz, $CDCl_3$) δ 170.3, 170.1, 170.0, 170.0, 165.9, 165.7, 165.7, 137.9, 137.6, 137.5, 137.4, 137.4, 137.4, 137.2, 137.1, 137.0, 136.9, 136.1, 136.1, 136.0, 135.7, 133.5, 133.5, 133.3, 133.3, 133.2, 133.1, 132.5, 131.7, 131.7, 131.6, 131.6, 131.6, 131.4, 131.1, 131.0, 131.0, 130.9, 130.3, 130.2, 130.0, 129.9, 129.8, 129.8, 129.7, 129.7, 129.6, 129.1, 129.1, 129.1, 128.9, 128.9, 128.7, 128.7, 128.6, 128.5, 128.5, 128.4, 128.3, 128.3, 128.2, 128.2, 128.0, 127.9, 127.9, 127.8, 127.7, 127.7, 127.7, 127.6, 127.5, 126.7, 126.5, 126.3, 126.2, 126.1, 125.9, 125.7, 121.8, 121.7, 121.1, 98.6, 98.2, 98.0, 97.2, 97.1, 97.1, 86.3, 80.5, 79.3, 79.0, 78.1, 77.9, 77.8, 75.3, 74.4, 74.3, 74.2, 74.0, 73.9, 73.2, 73.1, 73.0, 72.9, 72.8, 72.6, 72.5, 72.4, 72.2, 71.7, 69.9, 68.9, 68.8, 68.7, 68.6, 66.3, 65.2, 65.0, 64.6, 64.4, 64.3, 64.1, 63.0, 62.4, 62.3, 62.2, 62.1, 27.0, 27.0, 26.9, 26.9, 21.2, 21.2, 21.2, 20.8, 20.7, 20.7, 19.6, 19.5, 19.5; HRMS (MALDI): *m/z* calcd for $C_{222}H_{232}O_{44}N_{12}Br_4Si_4SNa$ $[M + Na]^+$: 4259.3608, found: 4259.3645.

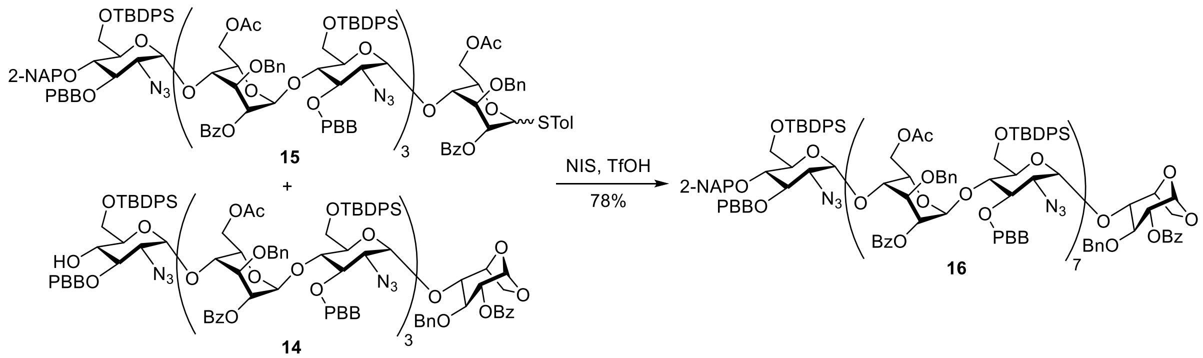

Fully protected hexadecasaccharide 16. A solution of the thioglycoside **15** (1.68 g, 0.398 mmol), the alcohol **14** (1.25 g, 0.318 mmol), and freshly dried 4 Å molecular sieves (1.46 g) in CH_2Cl_2 (29 mL) was stirred at room temperature for 1 hour under a nitrogen atmosphere. The reaction flask was cooled down to –40 °C, *N*-iodosuccinimide (NIS, 107 mg, 0.478 mmol) and triflic acid (TfOH, 7.02 µL, 0.080 mmol) was added to the solution, and the mixture was gradually warmed up to –20 °C. After stirring for 3 h, Et_3N was added to quench the reaction mixture, and the whole mixture was filtered through Celite. The crude compound was dissolved in CH_2Cl_2 and washed with 20% $Na_2S_2O_{3(aq)}$ and $NaHCO_{3(aq)}$ solution, water, and brine. The organic solution was dried over anhydrous $MgSO_4$, filtered, and concentrated under reduced pressure. The filtrate was concentrated *in vacuo* to furnish a residue, which was purified by column chromatography (ethyl acetate/hexane = 1/3.5, v/v) to provide the desired hexadecasaccharide **16** (2.01 g, 78%). $[\alpha]_D^{26}$ –20.0 (*c* 0.1, $CHCl_3$); IR (thin film) *ν* 3070, 3029, 2931, 2858, 2109, 1745, 1721, 1601, 1588, 1489, 1454, 1428, 1367, 1317, 1268, 1241, 1157, 1112, 1070, 1027, 875, 822, 804, 742, 702, 613, 505 cm^{-1}; ^{1}H NMR (600 MHz, $CDCl_3$) δ 8.04 (d, *J* = 8.3 Hz, 2H), 7.99–7.94 (m, 15H), 7.85–7.82 (m, 1H), 7.75 (d, *J* = 8.4 Hz, 1H), 7.73–7.70 (m, 1H), 7.65 (d, *J* = 7.2 Hz, 2H), 7.63–7.55 (m, 33H), 7.53 (d, *J* = 6.1 Hz, 2H), 7.49 (dd, *J* = 6.2, 3.2 Hz, 2H), 7.44 (q, *J* = 8.4 Hz, 4H), 7.40–7.27 (m, 59H), 7.25–7.11 (m, 60H), 7.09 (d, *J* = 8.8 Hz, 2H), 6.97 (d, *J* = 8.1 Hz, 2H), 6.91–6.84 (m, 13H), 5.48 (d, *J* = 1.7 Hz, 1H), 5.37 (d, *J* = 7.8 Hz, 6H), 5.28 (s, 1H), 5.12 (d, *J* = 19.0 Hz, 8H), 5.02 (d, *J* = 8.2 Hz, 1H), 4.89 (d, *J* = 11.3 Hz, 1H), 4.85–4.80 (m, 4H), 4.76 (d, *J* = 11.3 Hz, 5H), 4.74–4.70 (m, 2H), 4.67 (d, *J* = 11.2 Hz, 1H), 4.64–4.53 (m, 16H), 4.50 (d, *J* = 2.8 Hz, 1H), 4.46 (d, *J* = 4.6 Hz, 4H), 4.32–4.27 (m, 4H), 4.23 (s, 5H), 4.10–3.92 (m, 34H), 3.88–3.78 (m,

14H), 3.74 (t, J = 9.7 Hz, 1H), 3.69–3.61 (m, 8H), 3.59–3.54 (m, 2H), 3.53–3.46 (m, 16H), 3.36 (s, 1H), 3.33 (s, 4H), 3.29 (dd, J = 10.1, 3.8 Hz, 1H), 3.24 (dd, J = 10.0, 2.6 Hz, 1H), 3.15 (dd, J = 10.6, 2.7 Hz, 1H), 3.11 (d, J = 9.9 Hz, 5H), 1.68 (s, 3H), 1.66 (s, 3H), 1.61 (s, 3H), 1.60 – 1.58 (m, 12H), 1.04 (s, 9H), 1.00 (s, 9H), 0.98 (s, 45H), 0.95 (s, 9H); ^{13}C NMR (150 MHz, $CDCl_3$) δ 170.0, 169.9, 165.6, 137.9, 137.2, 137.0, 136.8, 135.9, 135.9, 135.8, 135.6, 135.6, 133.4, 133.2, 133.1, 132.9, 132.8, 131.4, 131.2, 130.9, 130.9, 129.9, 129.8, 129.6, 129.6, 129.5, 129.4, 129.4, 129.2, 129.0, 128.9, 128.8, 128.8, 128.7, 128.5, 128.4, 128.2, 128.1, 127.9, 127.8, 127.8, 127.7, 127.6, 127.6, 127.5, 127.4, 126.4, 126.2, 126.0, 125.8, 121.7, 121.4, 121.0, 99.2, 98.4, 97.5, 97.0, 96.9, 80.4, 79.2, 78.9, 78.5, 77.2, 77.0, 76.8, 75.1, 74.9, 74.3, 74.2, 74.0, 73.9, 73.8, 73.1, 72.9, 72.9, 72.8, 72.7, 72.5, 72.3, 72.1, 71.9, 69.2, 68.7, 68.4, 65.8, 65.5, 64.8, 64.5, 64.2, 64.0, 63.8, 62.3, 62.1, 26.8, 20.5, 19.4; HRMS (MALDI): *m/z* calcd for $C_{417}H_{438}O_{87}N_{24}Br_8Si_8Na$ [M + Na]$^+$: 8065.0981, found: 8065.0646.

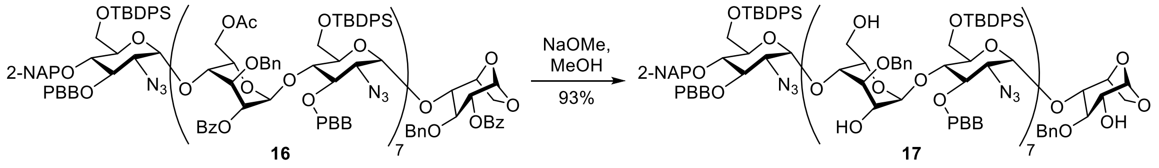

Compound 17. Sodium methoxide in MeOH (1 M, 3.33 mL) was added to a solution of compound **16** (1.25 g, 0.155 mmol) in THF (10 mL) at room temperature under a nitrogen atmosphere. After stirring for 1 h, the reaction solution was neutralized by DOWEX 50WX4-200 acidic resin. The resulting mixture was filtered through filter paper, and the filtrate was concentrated *in vacuo* to yield the crude product. Purification by precipitation in cold MeOH provided the polyol **17** (1.01 g, 93%). $[\alpha]_D^{25}$ +22.5 (*c* 0.1, $CHCl_3$); IR (thin film) ν 3585, 3571, 3069, 2932, 2885, 2858, 2112, 1590, 1488, 1472, 1428, 1390, 1322, 1258, 1157, 1104, 1071, 1043, 1002, 869, 823, 806, 744, 702, 615, 504 cm^{-1}; ^{1}H NMR (600 MHz, $CDCl_3$) δ 7.84–7.71 (m, 28H), 7.69 (dt, J = 8.1, 2.0 Hz, 4H), 7.67 (d, J = 7.4 Hz, 2H), 7.60 (s, 1H), 7.48 (dd, J = 6.2, 3.2 Hz, 2H), 7.46–7.31 (m, 82H), 7.31–7.27 (m, 5H), 7.25–7.19 (m, 7H), 7.16 (d, J = 7.7 Hz, 2H), 7.14–7.12 (d, J = 7.4 Hz, 4H), 7.11–7.06 (m, 9H), 7.04 (d, J = 8.2 Hz, 2H), 6.99–6.90 (m, 12H), 5.31–5.21 (m, 7H), 5.12 (s, 1H), 5.04 (d, J = 4.0 Hz, 1H), 5.00–4.97 (m, 5H), 4.90 (d, J = 5.2 Hz, 4H), 4.82 (d, J = 10.8 Hz, 1H), 4.76 (d, J = 10.9 Hz, 1H), 4.71 (dd, J = 10.3, 4.3 Hz, 6H), 4.61–4.51 (m, 9H), 4.47–4.34 (m, 12H), 4.19 (t, J = 6.8 Hz, 1H), 4.13–3.98 (m, 13H), 3.97–3.75 (m, 33H), 3.71 (t, J = 9.7 Hz, 2H), 3.65 (dd, J = 8.1, 6.0 Hz, 2H), 3.63–3.48 (m, 28H), 3.44–3.30 (m, 14H), 3.08–3.05 (m, 1H), 3.00 (s, 5H), 1.08 (s, 45H), 1.07 (s, 18H), 1.06 (s, 9H); ^{13}C NMR (150 MHz, $CDCl_3$) δ 138.3, 137.3, 137.2, 136.6, 136.4, 136.0, 135.9, 135.8, 135.8, 135.7, 135.6, 135.3, 133.3, 133.3, 133.2, 133.1, 133.0, 132.9, 132.8, 131.5, 131.3, 131.2, 129.8, 129.7, 129.7, 129.6, 129.5, 129.2, 129.2, 128.6, 128.6, 128.5, 128.5, 128.5, 128.2, 128.2, 127.9, 127.8, 127.7, 127.7, 127.7, 127.5, 127.5, 126.2, 126.1, 125.9, 125.5, 121.8, 121.5, 101.5, 100.3, 99.8, 99.1, 94.6, 94.4, 94.1, 83.2, 81.1, 80.1, 78.7, 78.6, 77.2, 77.0, 76.8, 75.7, 75.1, 74.9, 74.8, 74.3, 74.1, 74.0, 73.6, 72.9, 72.5, 72.4, 72.3, 72.2, 72.2, 71.8, 70.2, 69.9, 69.7, 66.5, 66.4, 66.0, 65.8, 65.2, 64.4, 63.8, 62.5, 62.0, 61.5, 26.8, 19.4, 19.3, 19.3; HRMS (MALDI): *m/z* calcd for $C_{347}H_{392}O_{72}N_{24}Br_8Si_8Na$ [M + Na]$^+$: 6937.9692, found: 6937.9827.

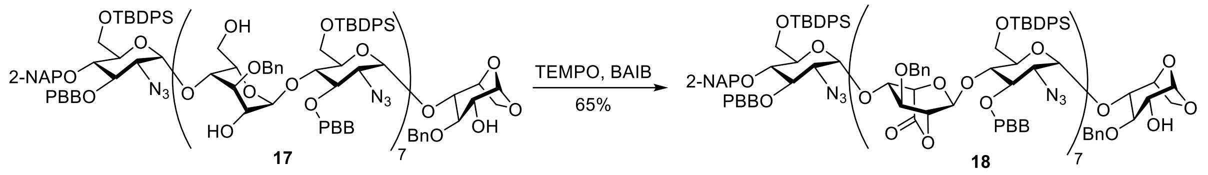

Compound 18. To a solution of the polyol **17** (1.01 g, 0.146 mmol) in a THF and CH_2Cl_2 [1/2 (v/v), 6 mL] mixed solvent, (2,2,6,6-tetramethylpiperidin-1-yl)oxyl free radical (TEMPO, 11.4 mg, 0.073 mmol) and bis(acetoxy)iodobenzene (BAIB, 958 mg, 2.93 mmol) were added at room temperature. After stirring for 48 h, ethyl acetate was added to the solution, and the mixture was sequentially washed with 10% $Na_2S_2O_{3(aq)}$, saturated $NaHCO_{3(aq)}$, and brine. The organic layer was dried over anhydrous $MgSO_4$, filtered, and concentrated *in vacuo* to get the crude product, which was purified by flash column chromatography (ethyl acetate/hexane = 1/3, v/v) on silica gel to furnish the heptalactone **18** (654 mg, 65%). $[\alpha]_D^{25}$ +17.5 (*c* 0.1, $CHCl_3$); IR (thin film) ν 3518, 3070, 2931, 2892, 2859, 2109, 1794, 1590, 1488, 1472, 1428, 1390, 1365, 1316, 1260, 1216, 1154, 1113, 1085, 1071, 1038, 874, 822, 806, 755, 702, 668, 614, 505 cm^{-1}; ^1H NMR (600 MHz, $CDCl_3$) δ 7.84–7.81 (m, 1H), 7.75 (dd, *J* = 8.6, 3.8 Hz, 2H), 7.72–7.68 (m, 13H), 7.66–7.59 (m, 20H), 7.49–7.28 (m, 110H), 7.23 (td, *J* = 8.2, 2.6 Hz, 12H), 7.18 (d, *J* = 8.1 Hz, 2H), 5.45 (d, *J* = 10.8 Hz, 5H), 5.40 (s, 1H), 5.29 (d, *J* = 1.9 Hz, 1H), 5.27 (d, *J* = 3.8 Hz, 1H), 4.99–4.88 (m, 10H), 4.81 (d, *J* = 11.0 Hz, 1H), 4.76 (d, *J* = 11.0 Hz, 1H), 4.71 (d, *J* = 11.5 Hz, 1H), 4.61 (td, *J* = 11.1, 9.8, 4.7 Hz, 12H), 4.54 (dd, *J* = 11.8, 6.2 Hz, 6H), 4.47 (d, *J* = 11.5 Hz, 1H), 4.37–4.23 (m, 12H), 4.19 (s, 6H), 4.15 (d, *J* = 3.6 Hz, 5H), 4.06 (dt, *J* = 11.3, 5.9 Hz, 7H), 3.98 (d, *J* = 11.2 Hz, 1H), 3.94 (d, *J* = 7.8 Hz, 1H) 3.88–3.51 (m, 52H), 3.47 (d, *J* = 9.9 Hz, 1H), 3.41–3.36 (m, 2H), 3.35–3.28 (m, 5H), 1.08 (s, 9H), 1.07 (s, 45H), 1.06 (s, 18H); ^{13}C NMR (150 MHz, $CDCl_3$) δ 167.2, 167.0, 138.2, 137.6, 136.8, 135.9, 135.8, 135.6, 135.5, 133.2, 132.9, 132.4, 132.2, 131.6, 131.5, 130.1, 130.0, 129.9, 129.7, 128.6, 128.6, 128.5, 128.5, 128.4, 128.3, 128.2, 128.1, 128.0, 127.9, 127.7, 127.7, 127.6, 127.5, 126.1, 125.4, 121.8, 121.4, 121.2, 101.5, 99.3, 99.2, 97.6, 97.0, 83.3, 80.5, 79.7, 78.2, 77.2, 77.0, 76.8, 76.3, 75.7, 75.0, 74.8, 74.7, 74.0, 73.5, 72.6, 72.3, 72.2, 72.0, 71.8, 69.1, 67.9, 65.1, 63.4, 63.1, 61.7, 61.3, 26.9; HRMS (MALDI): *m/z* calcd for $C_{347}H_{364}O_{72}N_{24}Br_8Si_8Na$ $[M + Na]^+$: 6909.7456, found: 6909.7424.

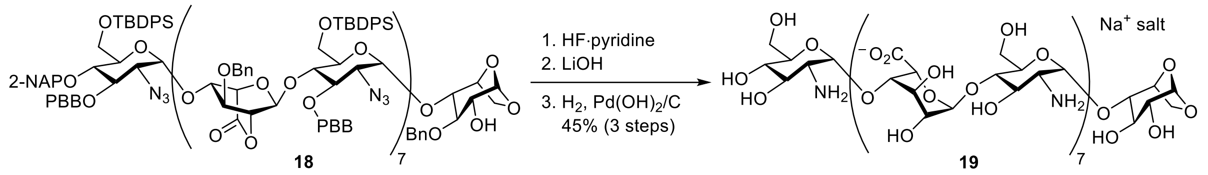

Compound S3. (1) *Desilylation.* HF·pyridine complex (2 mL) was added to a solution of the heptalactone **18** (560 mg, 0.081 mmol) in THF and pyridine (1/1 (v/v), 20 mL) mixed solvent at 0 °C. After stirring for 48 h at room temperature, the reaction mixture was quenched with silica gel and filtered through a Celite bed. The filtrate was concentrated *in vacuo* to afford the crude product, which was purified by flash column chromatography (methanol/chloroform = 1/50, v/v) on silica gel to furnish the desilylated hexadecasaccharide (332 mg).

(2) *Lactone ring opening.* An aqueous solution of LiOH (2.60 mL, 0.5 M) was added to a solution of the desilylated hexadecasaccharide (332 mg, 0.067 mmol) in THF/MeOH (3/1 (v/v), 8 mL) at room temperature. The mixture was kept stirring for 3 h, and then the reaction solution was neutralized by DOWEX 50WX4-200 acidic resin. The resulting mixture was filtered through filter paper, and the filtrate was concentrated *in vacuo* to yield the corresponding carboxylic acid derivative (289 mg).

(3) *Hydrogenolysis*. The carboxylic acid derivative (90 mg, 0.018 mmol) and 20% $Pd(OH)_2$ on carbon (270 mg) in phosphate buffer (20 mM, *p*H = 7.0) and MeOH mixed solvent (1/1 ratio, 3 mL) was equipped with a hydrogen balloon, and the mixture was stirred at room temperature for 48 h. The whole mixture was filtered through celite, and the filtrate was concentrated *in vacuo.* The residue was purified through a Sephadex G-10 column with H_2O as eluent. Lyophilization of the isolated material afforded the octaamine derivative **19** (32.5 mg, 45% over three steps) as a white solid. ^1H NMR (600 MHz, D_2O) δ 5.53–5.43 (m, 5H), 5.36 (s, 1H), 4.97–4.83 (m, 12H), 4.74–4.67 (m, 5H), 4.19–4.07 (m, 6H), 4.02–3.77 (m, 31H), 3.77–3.59 (m, 15H), 3.55 (d, *J* = 8.4 Hz, 1H), 3.45 (t, *J* = 9.6 Hz, 1H), 3.37–3.23 (m, 6H); ^{13}C NMR (150 MHz, D_2O) δ 175.9, 174.6, 174.6, 174.4, 101.9, 101.8, 101.4, 101.3, 101.2, 97.6, 92.5, 84.5, 82.5, 81.5, 79.4, 76.7, 76.5, 75.7, 75.5, 75.4, 74.6, 74.4, 74.4, 74.3, 73.9, 73.8, 72.8, 72.5, 72.2, 71.9, 71.5, 71.3, 71.2, 70.2, 70.1, 70.1, 70.0, 69.9, 69.7, 69.2, 69.1, 68.4, 65.1, 61.2, 60.8, 59.8, 59.7, 59.3, 54.3, 54.2, 54.0; HRMS (ESI): *m/z* calcd for $C_{96}H_{152}O_{79}N_8$ $[M + 5H]^{2-}$: 1340.9083, found: 1340.9054.

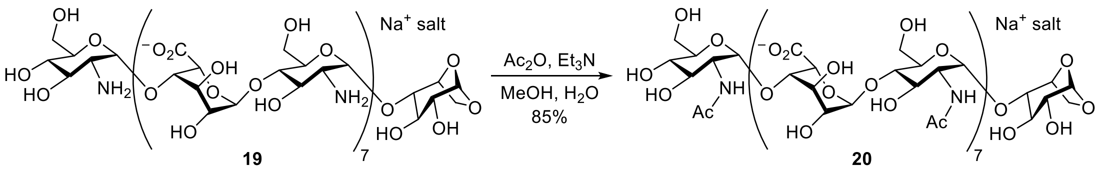

HS-based hexadecasaccharide 20. Compound **19** (12.0 mg, 4.23 µmol) was dissolved in H_2O/MeOH (1/1, 1 mL) and Et_3N (94.1 µL, 0.676 mmol) and Ac_2O (31.9 µL, 0.338 mmol) were added to it. The mixture was allowed to stir for 24 h at room temperature. The solvent was evaporated *in vacuo*, and the residue was purified through a Sephadex G-10 column with water as eluent, followed by Na^+ exchange through AG 50W-X8 (Na^+ form) cation exchange resin. Lyophilization of the isolated material was performed to afford compound **20** (11.4 mg, 85%) as a white solid. ^1H NMR (600 MHz, D_2O) δ 5.35 (d, *J* = 1.8 Hz, 1H), 5.19–5.14 (m, 7H), 5.10 (d, *J* = 3.7 Hz, 1H), 4.91 (d, *J* = 3.5 Hz, 1H), 4.89 (d, *J* = 3.7 Hz, 5H), 4.74 (d, *J* = 4.5 Hz, 1H), 4.72 (d, *J* = 3.2 Hz, 6H), 4.69 (d, *J* = 2.8 Hz, 1H), 4.10 (d, *J* = 8.4 Hz, 2H), 4.06 (t, *J* = 3.4 Hz, 2H), 4.03 (d, *J* = 4.2 Hz, 5H), 3.96–3.92 (m, 8H), 3.90 (d, *J* = 3.4 Hz, 1H), 3.88–3.79 (m, 32H), 3.76 (d, *J* = 5.4 Hz, 2H), 3.74–3.70 (m, 16H), 3.68–3.64 (m, 9H), 3.52 (dd, *J* = 8.1, 1.9 Hz, 1H), 3.46 (q, *J* = 10.0 Hz, 2H), 2.02 (s, 3H), 2.01 (s, 3H), 2.00 (s, 18H); ^{13}C NMR (150 MHz, D_2O) δ 174.9, 174.9, 174.7, 174.4, 174.4, 174.3, 101.7, 101.6, 101.2, 98.9, 94.5, 94.4, 84.4, 82.6, 81.6, 79.8, 77.7, 77.2, 76.6, 76.5, 75.5, 74.6, 74.3, 74.1, 74.0, 72.9, 72.4, 71.9, 71.7, 71.2, 71.1, 69.9, 69.8, 69.7, 69.7, 69.6, 69.6, 69.4, 69.2, 65.1, 61.3, 60.9, 60.1, 60.0, 59.6, 53.8, 53.7, 53.5, 21.8, 21.8; HRMS (ESI): *m/z* calcd for $C_{112}H_{167}O_{87}N_8$ $[M + 4H]^{3-}$: 1005.2969, found: 1005.2931.

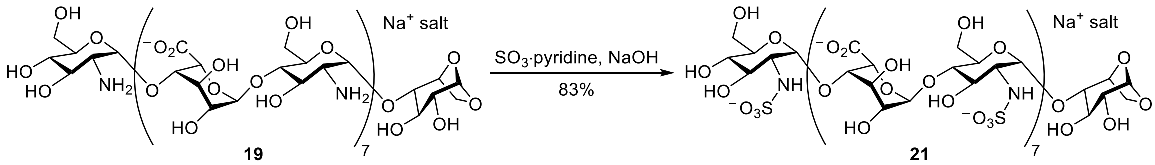

HS-based hexadecasaccharide 21. Compound **19** (16.4 mg, 5.78 µmol) was dissolved in H_2O (1.5 mL) and the pH of the solution was adjusted to within 9.5~10 by 1 M $NaOH_{(aq)}$. SO_3·Pyridine (184 mg, 1.16 mmol) was added in five equal portions in half-hour intervals while keeping the pH of the reaction solution to within 9.5~10 using 1 M $NaOH_{(aq)}$. The mixture was allowed to further stir for 48 h at room temperature. The solvent was evaporated in vacuo, and the residue was purified through a Sephadex G-10 column with water as eluent, followed by Na^+ exchange through AG 50W-X8 (Na^+ form) cation exchange resin. Lyophilization of the isolated material was performed to afford compound **21** (17.5

mg, 83%) as a white solid. ^1H NMR (600 MHz, D_2O) δ 5.42–5.33 (m, 6H), 4.94 (d, *J* = 3.7 Hz, 5H), 4.83 (d, *J* = 10.2 Hz, 7H), 4.16–4.00 (m, 12H), 3.80 (d, *J* = 11.6 Hz, 18H), 3.76–3.66 (m, 15H), 3.63 (t, *J* = 10.4 Hz, 5H), 3.55 (dd, *J* = 8.2, 2.0 Hz, 1H), 3.46 (t, *J* = 9.6 Hz, 1H), 3.31–3.25 (m, 1H), 3.23–3.18 (m, 5H); ^{13}C NMR (150 MHz, D_2O) δ 174.9, 101.6, 101.2, 99.0, 95.6, 95.4, 84.5, 82.5, 81.6, 79.8, 77.7, 77.0, 76.9, 75.4, 74.8, 74.7, 74.0, 73.8, 72.9, 72.3, 71.7, 71.4, 71.2, 70.9, 69.8, 69.7, 69.7, 69.5, 69.2, 69.1, 69.0, 68.9, 68.6, 68.3, 68.1, 65.1, 61.4, 60.9, 60.1, 60.1, 59.7, 58.1, 57.9, 57.8; HRMS (ESI): *m/z* calcd for $C_{96}H_{150}O_{103}N_8S_8$ [M + 11H]$^{4-}$: 830.1140, found: 830.1161.

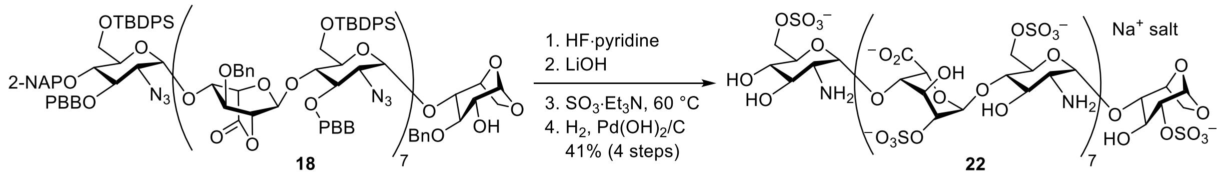

Compound 22. (1) *Desilylation.* HF·pyridine complex (2 mL) was added to a solution of the heptalactone **18** (650 mg, 0.094 mmol) in THF and pyridine (1/1 (v/v), 20 mL) mixed solvent at 0 °C. After stirring for 48 h at room temperature, the reaction mixture was quenched with silica gel and filtered through a Celite bed. The filtrate was concentrated *in vacuo* to afford the crude product, which was purified by flash column chromatography (MeOH/$CHCl_3$ = 1/50, v/v) on silica gel to furnish the desilylated hexadecasaccharide (376 mg, 80%); HRMS (MALDI): *m/z* calcd for $C_{219}H_{220}O_{72}N_{24}Br_8Na$ [M + Na]$^+$: 5002.5005, found: 5002.5182.

(2) *Lactone ring opening.* An aqueous solution of LiOH (2.80 mL, 0.5 M) was added to a solution of the desilylated hexadecasaccharide (376 mg, 0.076 mmol) in THF/MeOH (3/1 (v/v), 8 mL) at room temperature. The mixture was kept stirring for 3 h, and then the reaction solution was neutralized by DOWEX 50WX4-200 acidic resin. The resulting mixture was filtered through filter paper, and the filtrate was concentrated *in vacuo* to yield the corresponding hexadecasaccharide carboxylic acid derivative (282 mg).

(3) *O-Sulfonation.* A solution of the carboxylic acid derivative (282 mg, 0.055 mmol) and sulfur trioxide-triethylamine complex (801 mg, 4.42 mmol) in DMF (2.8 mL) was kept stirring at 60 °C for 48 h under a nitrogen atmosphere. The reaction flask was cooled down to room temperature, a solution of $NaHCO_{3(aq)}$ (1.2 mL, 0.5 M) was added to the mixture and the resulting solution was kept stirring for another 2 h. The solvent was co-evaporated with MeOH under reduced pressure and a mixed solvent of CH_2Cl_2/MeOH [1/1 (v/v), 10 mL] was added to the solid mass. The mixture was filtered, and the filtrate was concentrated *in vacuo*. The residue was layered on the top of a Sephadex LH-20 chromatography column, which was eluted with MeOH to get the *O*-sulfonated hexadecasaccharide (318 mg, 66% over two steps).

(4) *Hydrogenolysis*. The *O*-sulfonated hexadecasaccharide (110 mg, 0.017 mmol) and 20% $Pd(OH)_2$ on carbon (330 mg) in phosphate buffer (20 mM, *p*H = 7.0) and MeOH mixed solvent (1/1 ratio, 4 mL) was equipped with a hydrogen balloon, and the mixture was stirred at room temperature for 48 h. The whole mixture was filtered through Celite, and the filtrate was concentrated *in vacuo.* The residue was purified through a Sephadex G-10 column with H_2O as eluent. Lyophilization of the isolated material afforded compound **22** (53.5 mg, 75%) as a white solid; ^1H NMR (600 MHz, D_2O) δ 5.67 (d, *J* = 1.7 Hz, 1H), 5.52–5.36 (m, 7H), 5.23–5.18 (m, 5H), 4.92 (s, 1H), 4.91–4.88 (m, 2H), 4.88–4.83 (m, 5H), 4.74 (d, *J* = 3.8 Hz, 2H), 4.41–4.10 (m, 36H), 4.06 (t, *J* = 9.1 Hz, 2H), 3.99–3.92 (m, 15H), 3.87–3.67 (m, 11H), 3.54 (t, *J* = 9.7 Hz, 1H), 3.48–3.34 (m, 7H); ^{13}C NMR (150 MHz, D_2O) δ 175.4, 175.3, 99.25, 99.0, 98.7, 98.5, 97.1, 91.2, 90.9, 80.3, 79.5, 77.5, 75.4, 75.1, 73.9, 72.6, 70.6, 70.2, 70.0, 69.5, 69.2, 68.6, 68.4, 68.1, 67.2, 66.7, 66.0, 65.4, 62.7, 62.6, 54.2, 53.9; HRMS (ESI): *m/z* calcd for $C_{96}H_{149}O_{127}N_8S_{16}$ [M + 18H]$^{5-}$: 791.8204, found: 791.8189.

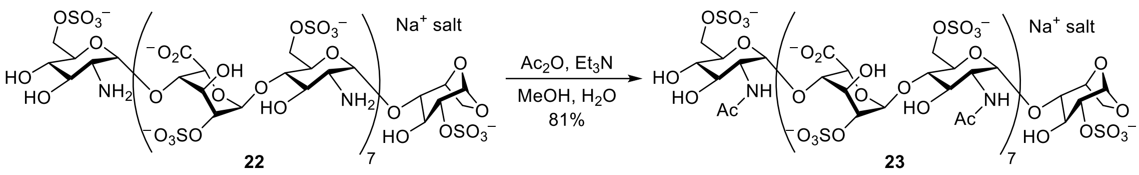

HS-based hexadecasaccharide 23. Compound **22** (15.8 mg, 3.53 μmol) was dissolved in H_2O/MeOH (1/1, 2 mL) and Et_3N (78.8 μL, 0.565 mmol) and Ac_2O (26.7 μL, 0.283 mmol) were added to it. The mixture was allowed to stir for 24 h at room temperature. The solvent was evaporated *in vacuo*, and the residue was purified through a Sephadex G-10 column with water as eluent, followed by Na^+ exchange through AG 50W-X8 (Na^+ form) cation exchange resin. Lyophilization of the isolated material was performed to afford compound **23** (13.8 mg, 81%) as a white solid. ^{1}H NMR (600 MHz, D_2O) δ 5.68 (s, 1H), 5.15–5.12 (m, 14H), 4.96 (s, 1H), 4.91–4.86 (m, 7H), 4.33–4.21 (m, 32H), 4.18–4.12 (m, 4H), 4.05–3.96 (m, 25H), 3.88 (s, 1H), 3.83–3.66 (m, 24H), 3.55 (t, J = 9.6 Hz, 1H), 2.04 (s, 15H), 2.02 (s, 6H), 2.00 (s, 3H); ^{13}C NMR (150 MHz, D_2O) δ 175.5, 175.3, 175.0, 174.7, 174.4, 102.4, 101.9, 99.6, 99.2, 99.1, 99.0, 98.9, 93.6, 93.3, 80.4, 79.7, 77.9, 76.2, 76.0, 74.0, 73.7, 71.2, 71.0, 70.9, 70.8, 69.9, 69.7, 69.4, 69.3, 69.1, 68.0, 67.8, 67.0, 66.4, 65.4, 64.3, 63.9, 63.8, 53.8, 53.6, 53.4, 53.2, 22.2, 21.9, 21.8; HRMS (ESI): *m/z* calcd for $C_{112}H_{156}O_{135}N_8S_{16}Na_8$ [M + 9H + 8Na]$^{6-}$: 745.0059, found: 745.0033.

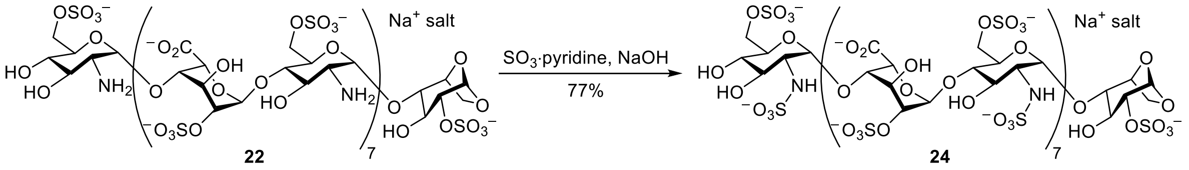

HS-based hexadecasaccharide 24. Compound **22** (21.2 mg, 4.74 μmol) was dissolved in H_2O (2 mL) and the pH of the solution was adjusted to within 9.5~10 by 1 M $NaOH_{(aq)}$. SO_3·pyridine (171 mg, 1.08 mmol) was added in five equal portions in half-hour intervals while keeping the pH of the reaction solution to within 9.5~10 using 1 M $NaOH_{(aq)}$. The mixture was allowed to further stir for 48 h at room temperature. The solvent was evaporated *in vacuo*, and the residue was purified through a Sephadex G-10 column with water as eluent, followed by Na^+ exchange through AG 50W-X8 (Na^+ form) cation exchange resin. Lyophilization of the isolated material was performed to afford compound **24** (21.9 mg, 77%) as a white solid. ^{1}H NMR (600 MHz, D_2O) δ 5.69 (d, J = 1.7 Hz, 1H), 5.50–5.35 (m, 7H), 5.25 (s, 1H), 5.23–5.16 (m, 4H), 5.13 (s, 1H), 4.89–4.83 (m, 7H), 4.76 (d, J = 2.6 Hz, 1H), 4.67 (s, 1H), 4.47–4.15 (m, 33H), 4.10 (s, 7H), 4.02–3.90 (m, 9H), 3.89–3.82 (m, 4H), 3.81–3.61 (m, 14H), 3.56 (t, J = 9.6 Hz, 1H), 3.37–3.20 (m, 7H); ^{13}C NMR (150 MHz, D_2O) δ 174.8, 174.62, 174.59, 99.5, 99.3, 99.1, 99.0, 98.7, 98.2, 96.8, 96.5, 80.09, 80.07, 77.5, 77.3, 76.7, 76.1, 75.9, 75.8, 74.0, 70.9, 70.6, 69.9, 69.7, 69.6, 69.5, 69.4, 69.2, 69.1, 67.0, 66.3, 65.4, 57.90, 57.85, 57.5, 57.4; HRMS (ESI): *m/z* calcd for $C_{96}H_{135}O_{151}N_8S_{24}Na_{13}$ [M + 12H + 13Na]$^{6-}$: 813.9190, found: 813.9227.

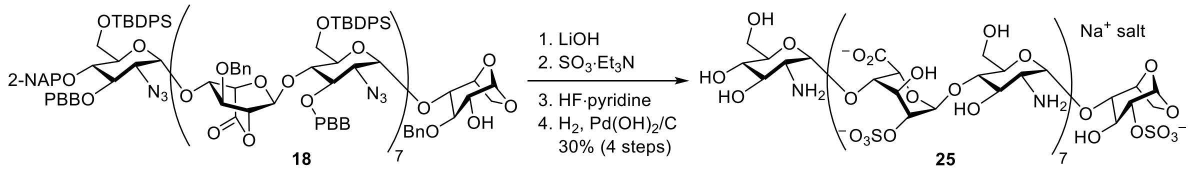

Compound 25. (1) *Lactone ring opening.* An aqueous solution of LiOH (5.68 mL, 0.5 M) was added to a solution of the heptalactone **18** (725 mg, 0.105 mmol) in THF/MeOH (3/1 (v/v), 9 mL) at room temperature. The mixture was

kept stirring for 3 h, and then the reaction solution was neutralized by DOWEX 50WX4-200 acidic resin. The resulting mixture was filtered through filter paper, and the filtrate was concentrated *in vacuo* to yield the corresponding hexadecasaccharide carboxylic acid derivative (664 mg).

(2) 2-*O-Sulfonation.* A solution of the carboxylic acid derivative (664 mg, 0.095 mmol) and sulfur trioxide-triethylamine complex (686 mg, 3.79 mmol) in DMF (2.60 mL) was kept stirring at 60 °C for 48 h under a nitrogen atmosphere. The reaction flask was cooled down to room temperature, a solution of $NaHCO_{3(aq)}$ (1.1 mL, 0.5 M) was added to the mixture and the resulting solution was kept stirring for another 2 h. The solvent was co-evaporated with MeOH under reduced pressure and a mixed solvent of CH_2Cl_2/MeOH [1/1 (v/v), 10 mL] was added to the solid mass. The mixture was filtered, and the filtrate was concentrated *in vacuo*. The residue was layered on the top of a Sephadex LH-20 chromatography column, which was eluted with MeOH to get the 2-*O*-sulfonated hexadecasaccharide (574 mg, 68% over two steps).

(3) *Desilylation.* HF·pyridine complex (2 mL) was added to a solution of the 2-*O*-sulfonated hexadecasaccharide (574 mg, 0.072 mmol) in THF and pyridine (1/1 (v/v), 18 mL) mixed solvent at 0 °C. After stirring for 48 h at room temperature, the reaction mixture was quenched with silica gel and filtered through Celite bed. The filtrate was concentrated *in vacuo* to afford the crude product. The residue was layered on the top of a Sephadex LH-20 chromatography column, which was eluted with MeOH to get the 2-*O*-sulfonated hexadecasaccharide (314 mg, 72%).

(4) *Hydrogenolysis*. The 2-*O*-sulfonated hexadecasaccharide (125 mg, 0.020 mmol) and 20% $Pd(OH)_2$ on carbon (375 mg) in phosphate buffer (20 mM, *p*H = 7.0) and MeOH mixed solvent (1/1 ratio, 4 mL) was equipped with a hydrogen balloon and the mixture was stirred at room temperature for 48 h. The whole mixture was filtered through Celite, and the filtrate was concentrated *in vacuo.* The residue was purified through a Sephadex G-10 column with H_2O as eluent. Lyophilization of the isolated material afforded the octaamine **25** (45.9 mg, 61%) as a white solid. ^{1}H NMR (600 MHz, D_2O) δ 5.59 (d, J = 1.7 Hz, 1H), 5.34–5.29 (m, 8H), 5.22 (s, 1H), 5.20 (s, 1H), 5.17 (s, 1H), 5.13 (s, 5H), 5.10 (s, 1H), 4.81 (d, J = 6.6 Hz, 5H), 4.29 (s, 2H), 4.25 (s, 10H), 4.17 (s, 2H), 4.09–4.04 (m, 10H), 3.87–3.82 (m, 8H), 3.79–3.71 (m, 31H), 3.67 (t, J = 9.6 Hz, 10H), 3.39 (t, J = 9.7 Hz, 1H), 3.26 (d, J = 10.9 Hz, 5H), 3.22 (d, J = 10.2 Hz, 2H); ^{13}C NMR (150 MHz, D_2O) δ 175.3, 99.0, 98.8, 91.3, 91.1, 80.3, 78.9, 76.1, 73.9, 72.4, 72.1, 71.4, 70.8, 70.0, 69.1, 68.1, 66.9, 65.3, 62.5, 59.7, 59.3, 54.5, 54.2; HRMS (ESI): *m/z* calcd for $C_{96}H_{151}O_{103}N_8S_8$ $[M + 12H]^{3-}$: 1106.8212, found: 1106.8252.

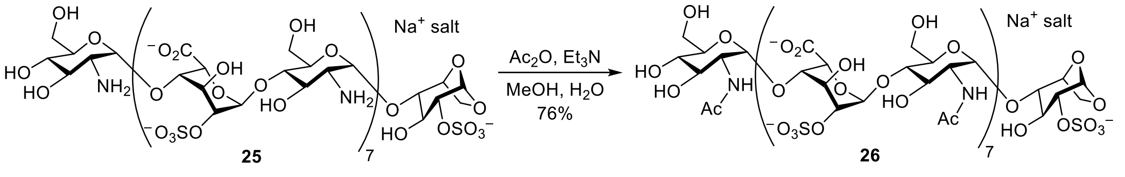

HS-based hexadecasaccharide 26. Compound **25** (10.2 mg, 2.79 μmol) was dissolved in H_2O/MeOH (1/1, 1 mL) and Et_3N (62.2 μL, 0.446 mmol) and Ac_2O (21.1 μL, 0.223 mmol) were added to it. The mixture was allowed to stir for 24 h at room temperature. The solvent was evaporated *in vacuo*, and the residue was purified through a Sephadex G-10 column with water as eluent, followed by Na^+ exchange through AG 50W-X8 (Na^+ form) cation exchange resin. Lyophilization of the isolated material was performed to afford compound **26** (8.46 mg, 76%) as a white solid; ^{1}H NMR (600 MHz, D_2O) δ 5.67 (s, 1H), 5.31 (s, 1H), 5.24 (s, 1H), 5.16 (d, J = 6.8 Hz, 6H), 5.12 (s, 4H), 5.08 (s, 6H), 4.90 (s, 1H), 4.88 (s, 4H), 4.31 (s, 8H), 4.28 (s, 2H), 4.25 (d, J = 11.7 Hz, 9H), 4.16 (d, J = 8.6 Hz, 2H), 4.12 (s, 2H), 4.00–3.97 (m, 18H), 3.87–3.79 (m, 42H), 3.77 (s, 2H), 3.72–3.66 (m, 16H), 3.46–3.43 (t, J = 9.9 Hz, 2H), 2.05 (s, 6H), 2.04 (s, 15H), 2.02 (s, 3H); ^{13}C NMR (150 MHz, D_2O) δ 175.6, 174.7, 99.4, 99.2, 99.0, 93.4, 93.3, 80.4, 79.1, 77.6, 77.2, 74.1, 73.1,

71.8, 71.2, 70.9, 70.2, 69.9, 69.2, 67.2, 65.3, 63.1, 60.2, 59.6, 53.9, 53.7, 53.4, 53.0, 22.2, 21.8; HRMS (ESI): *m/z* calcd for $C_{112}H_{165}O_{111}N_8S_8$ [M + 10H]$^{5-}$: 730.9067, found: 730.9020.

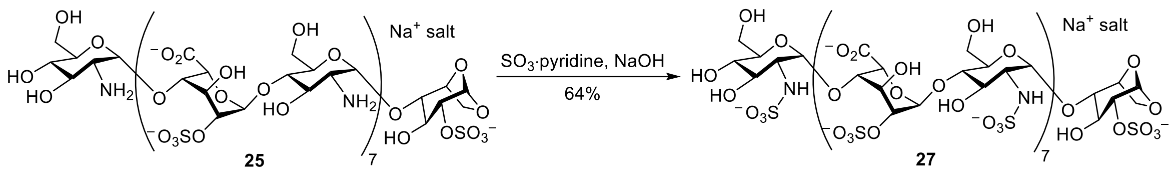

HS-based hexadecasaccharide 27. Compound **25** (14.0 mg, 3.83 μmol) was dissolved in H_2O (1 mL) and the pH of the solution was adjusted to within 9.5~10 by 1 M $NaOH_{(aq)}$. SO_3·pyridine (122 mg, 0.766 mmol) was added in five equal portions in half-hour intervals while keeping the pH of the reaction solution to within 9.5~10 using 1 M $NaOH_{(aq)}$. The mixture was allowed to further stir for 48 h at room temperature. The solvent was evaporated *in vacuo*, and the residue was purified through a Sephadex G-10 column with water as eluent, followed by Na^+ exchange through AG 50W-X8 (Na^+ form) cation exchange resin. Lyophilization of the isolated material was performed to afford compound **27** (10.9 mg, 64%) as a white solid. ^1H NMR (600 MHz, D_2O) δ 5.68 (d, *J* = 1.7 Hz, 1H), 5.41 (d, *J* = 3.6 Hz, 1H), 5.35 (d, *J* = 3.1 Hz, 1H), 5.29 (s, 6H), 5.24 (s, 6H), 5.17 (s, 1H), 4.85 (s, 5H), 4.33 (s, 7H), 4.28–4.26 (m, 1H), 4.23 (s, 7H), 4.18–4.15 (m, 2H), 4.05–4.01 (m, 8H), 3.88–3.78 (m, 34H), 3.70–3.64 (m, 19H), 3.45 (t, *J* = 9.7 Hz, 1H), 3.29–3.26 (m, 1H), 3.24–3.22 (m, 5H), 3.20 (d, *J* = 3.4 Hz, 1H); ^{13}C NMR (150 MHz, D_2O) δ 175.5, 175.3, 99.3, 99.1, 98.9, 97.3, 97.2, 80.1, 79.5, 77.3, 75.7, 75.5, 74.4, 74.0, 71.6, 71.0, 70.7, 69.9, 69.5, 68.5, 68.1, 67.6, 65.3, 60.2, 59.7, 58.3, 58.1; HRMS (ESI): *m/z* calcd for $C_{96}H_{143}O_{127}N_8S_{16}Na_5$ [M + 12H + 5Na]$^{6-}$: 678.0007, found: 678.0016.

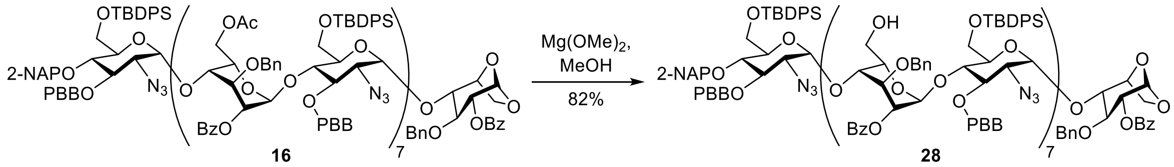

Compound 28. Magnesium methoxide in MeOH (7-8%, 4.50 mL) was added to a solution of compound **16** (750 mg, 0.093 mmol) in THF/CH_2Cl_2 (2/1, 9 mL) at room temperature under a nitrogen atmosphere. After stirring for 24 h, the reaction solution was neutralized by acetic acid, concentrated *in vacuo* and purified by column chromatography (ethyl acetate/hexane = 1/5, v/v) on silica gel to provide heptaol **28** (592 mg, 82%). IR (thin film) *ν* 3586, 3070, 2931, 2893, 2858, 2108, 178, 1590, 1472, 1489, 1453, 1428, 1391, 1362, 1317, 1269, 1216, 1152, 111, 1071, 1043, 1026, 1014, 870, 822, 805, 755, 702, 614, 505, 489 cm^{-1}; ^1H NMR (600 MHz, $CDCl_3$) δ 7.98 (d, *J* = 7.8 Hz, 14H), 7.83 (q, *J* = 4.4 Hz, 1H), 7.76–7.72 (m, 2H), 7.70 (d, *J* = 7.0 Hz, 3H), 7.66 (d, *J* = 7.3 Hz, 10H), 7.64–7.55 (m, 21H), 7.49 (dd, *J* = 6.3, 3.2 Hz, 2H), 7.45–7.32 (m, 55H), 7.32–7.26 (m, 27H), 7.26–7.19 (m, 25H), 7.17–7.14 (m, 22H), 7.11 (d, *J* = 8.1 Hz, 4H), 6.99 (d, *J* = 8.2 Hz, 2H), 6.94 (dd, *J* = 8.4, 2.2 Hz, 4H), 6.91 (d, *J* = 8.6 Hz, 7H), 5.38 (d, *J* = 6.7 Hz, 5H), 5.29 (s, 1H), 5.26 (d, *J* = 1.9 Hz, 1H), 5.18 (d, *J* = 3.9 Hz, 1H), 5.15 (d, *J* = 5.2 Hz, 6H), 4.92–4.81 (m, 11H), 4.76–4.67 (m, 9H), 4.64 (dd, *J* = 7.7, 3.5 Hz, 2H), 4.60–4.53 (m, 5H), 4.44–4.30 (m, 8H), 4.14 (s, 1H), 4.11–3.94 (m, 29H), 3.88–3.81 (m, 8H), 3.80–3.76 (m, 3H), 3.75–3.67 (m, 9H), 3.62 (t, *J* = 9.4 Hz, 2H), 3.59–3.46 (m, 20H), 3.43–3.33 (m, 11H), 3.28 (dd, *J* = 10.1, 3.5 Hz, 1H), 3.25–3.18 (m, 8H), 3.03 (s, 5H), 1.05 (s, 9H), 1.01 (s, 54H), 0.98 (s, 9H); ^{13}C NMR (150 MHz, $CDCl_3$) δ 165.6, 165.5, 138.3, 137.5, 137.4, 137.3, 136.7, 136.4, 135.9, 135.9, 135.8, 135.7, 135.5, 135.5, 135.4, 133.4, 133.3, 133.3, 133.2, 133.1, 133.1, 133.0, 132.9, 132.8, 132.7, 131.4, 131.3, 131.3, 131.2, 131.1, 130.8, 130.1, 130.1, 129.8, 129.7, 129.6, 129.6, 129.6, 129.5, 129.5, 129.4, 129.4, 128.6, 128.6, 128.5, 128.5, 128.4, 128.4, 128.4, 128.3, 128.2, 128.2, 128.1, 127.9,

127.8, 127.7, 127.7, 127.7, 127.6, 127.6, 127.5, 127.5, 127.5, 127.4, 127.4, 126.4, 126.1, 126.0, 125.7, 121.8, 121.6, 121.6, 101.4, 99.1, 98.2, 97.5, 97.1, 96.9, 83.1, 80.5, 79.3, 78.6, 78.6, 77.9, 77.2, 77.0, 76.8, 75.6, 75.2, 74.8, 74.4, 74.2, 73.9, 73.2, 72.9, 72.9, 72.8, 72.7, 72.6, 72.5, 72.5, 72.3, 69.1, 68.7, 68.6, 67.9, 67.7, 67.0, 66.9, 65.2, 64.0, 63.8, 63.7, 62.2, 61.5, 26.8, 19.4, 19.4, 19.3, 19.2; HRMS (MALDI): *m/z* calcd for $C_{403}H_{425}O_{80}N_{24}Br_8Si_8$ [M + H]$^+$: 7659.8328, found: 7659.3.

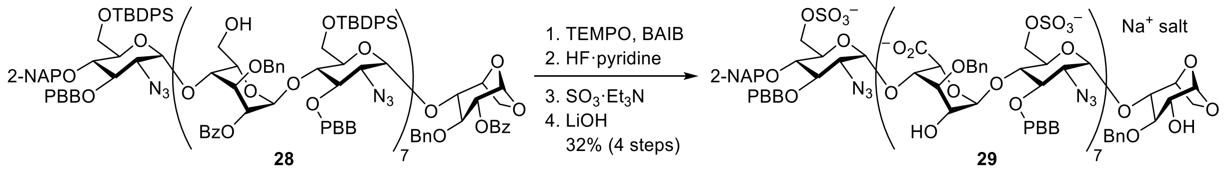

Compound 29. (1) *TEMPO oxidation.* To a solution of the heptaol **28** (560 mg, 0.072 mmol) in a THF and CH_2Cl_2 [1/2 (v/v), 3 mL] mixed solvent, TEMPO (5.65 mg, 0.036 mmol) and BAIB (466 mg, 1.44 mmol) were added at room temperature. After stirring for 48 h, ethyl acetate was added to the solution, and the mixture was sequentially washed with 10% $Na_2S_2O_{3(aq)}$, saturated $NaHCO_{3(aq)}$, and brine. The organic layer was dried over anhydrous $MgSO_4$, filtered, and concentrated *in vacuo* to get the crude product, which was purified by flash column chromatography (ethyl acetate/hexane = 1/4, v/v) on silica gel to furnish the hexadecasaccharide carboxylic acid derivative (454 mg, 80%).

(2) *Desilylation.* HF·pyridine complex (2 mL) was added to a solution of the hexadecasaccharide carboxylic acid derivative (450 mg, 0.057 mmol) in THF and pyridine (1/1 (v/v), 16 mL) mixed solvent at 0 °C. After stirring for 48 h at room temperature, the reaction mixture was quenched with silica gel and filtered through a Celite bed. The filtrate was concentrated *in vacuo* to afford the crude product, which was purified by flash column chromatography (methanol/chloroform = 1/40, v/v) on silica gel to furnish the desilylated hexadecasaccharide (242 mg, 71%).

(3) 6-*O*-*Sulfonation.* A solution of the desilylated carboxylic acid derivative (240 mg, 0.040 mmol) and sulfur trioxide-triethylamine complex (293 mg, 1.62 mmol) in DMF (2.4 mL) was kept stirring at 60 °C for 48 h under a nitrogen atmosphere. The reaction flask was cooled down to room temperature, an aqueous solution of $NaHCO_3$ (1.0 mL, 0.5 M) was added to the mixture and the resulting solution was kept stirring for another 2 h. The solvent was co-evaporated with MeOH under reduced pressure and a mixed solvent of CH_2Cl_2/MeOH [1/1 (v/v), 8 mL] was added to the solid mass. The mixture was filtered, and the filtrate was concentrated *in vacuo*. The residue was layered on the top of a Sephadex LH-20 chromatography column, which was eluted with MeOH to get the 6-*O*-sulfonated hexadecasaccharide (201 mg).

(4) *Lactone ring opening.* An aqueous solution of LiOH (2.80 mL, 0.5 M) was added to a solution of the 6-*O*-sulfonated hexadecasaccharide (201 mg, 0.029 mmol) in $CHCl_3$/MeOH/H_2O (2/7/1, v/v), 10 mL) at room temperature. The mixture was kept stirring for 24 h, and then the reaction solution was neutralized by DOWEX 50WX4-200 acidic resin. The resulting mixture was filtered through filter paper, and the filtrate was concentrated *in vacuo.* The residue was layered on the top of a Sephadex LH-20 chromatography column, which was eluted with MeOH, followed by Na^+ exchange through AG 50W-X8 (Na^+ form) cation exchange resin to get compound **29** (138 mg, 56% over two steps). ^{1}H NMR (600 MHz, MeOD) δ 7.81–7.79 (m, 1H), 7.78–7.76 (m, 1H), 7.75–7.71 (m, 2H), 7.45–7.44 (m, 5H), 7.42–7.39 (m, 21H), 7.37–7.34 (m, 13H), 7.33–7.29 (m, 18H), 7.28–7.26 (m, 3H), 7.25–7.21 (m, 23H), 7.18 (d, *J* = 8.5 Hz, 2H), 5.68 (d, *J* = 1.8 Hz, 1H), 5.29 (s, 8H), 5.22 (d, *J* = 3.9 Hz, 1H), 5.17 (s, 1H), 5.12 (d, *J* = 3.8 Hz, 1H), 5.05–5.02 (m, 10H), 4.97–4.93 (m, 5H), 4.80 (s, 6H), 4.74 (d, *J* = 6.8 Hz, 8H), 4.69 (t, *J* = 9.6 Hz, 5H), 4.64 (d, *J* = 11.5 Hz, 8H), 4.49 – 4.45 (m, 8H), 4.34 (d, *J* = 11.5 Hz, 5H), 4.30–4.26 (m, 11H), 4.21 (d, *J* = 6.5 Hz, 6H), 4.13 (s, 3H), 4.00 (t, *J* = 9.1 Hz, 9H), 3.93 (s, 3H), 3.89–3.83 (m, 18H), 3.79–3.72 (m, 11H), 3.64–3.61 (m, 2H), 3.51 (d, *J* = 10.7 Hz, 7H); ^{13}C NMR (150 MHz,

MeOD) δ 174.7, 139.8, 139.3, 139.1, 138.8, 138.5, 137.0, 135.9, 134.6, 134.4, 132.3, 131.3, 130.9, 130.4, 130.3, 129.5, 129.4, 129.3, 129.1, 128.9, 128.6, 128.5, 128.0, 127.5, 126.9, 126.8, 122.4, 122.1, 103.0, 101.1, 100.8, 96.3, 82.0, 81.9, 80.8, 80.5, 80.3, 79.5, 78.9, 77.0, 76.1, 75.8, 75.3, 75.1, 74.0, 73.8, 73.4, 73.3, 72.2, 71.1, 70.4, 69.3, 68.8, 68.0, 67.4, 66.9, 66.5, 65.5, 64.6, 57.4; HRMS (ESI): *m/z* calcd for $C_{219}H_{222}Br_8O_{103}N_{24}S_8Na_5$ $[M + 3H + 5Na]^{7-}$: 837.6260, found: 837.6238.

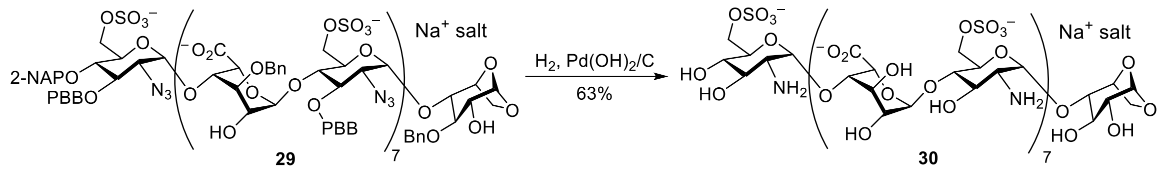

Compound 30. The 6-*O*-sulfonated hexadecasaccharide derivative **29** (120 mg, 0.020 mmol) and 20% $Pd(OH)_2$ on carbon (360 mg) in phosphate buffer (20 mM, *p*H = 7.0) and MeOH mixed solvent (1/1 ratio, 4 mL) was equipped with a hydrogen balloon and the mixture was stirred at room temperature for 48 h. The whole mixture was filtered through Celite, and the filtrate was concentrated *in vacuo.* The residue was purified through a Sephadex G-10 column with H_2O as eluent. Lyophilization of the isolated material afforded compound **30** (45.5 mg, 63%) as a white solid. ^1H NMR (600 MHz, D_2O) δ 5.69 (s, 1H), 5.49 (d, *J* = 4.4 Hz, 8H), 5.41 (d, *J* = 3.8 Hz, 1H), 4.94 (d, *J* = 3.5 Hz, 7H), 4.91 (d, *J* = 3.5 Hz, 1H), 4.76–4.74 (m, 7H), 4.72 (d, *J* = 3.1 Hz, 1H), 4.35 (d, *J* = 11.1 Hz, 8H), 4.31 (s, 1H), 4.25 (d, *J* = 11.0 Hz, 8H), 4.21 (s, 1H), 4.18–4.16 (m, 9H), 4.08 (d, *J* = 10.2 Hz, 6H), 3.99 – 3.93 (m, 9H), 3.87 (t, *J* = 9.8 Hz, 8H), 3.83 (s, 1H), 3.81–3-77 (m, 8H), 3.75–3.73 (m, 6H), 3.56 (t, *J* = 9.7 Hz, 1H), 3.37–3.31 (m, 8H); ^{13}C NMR (150 MHz, D_2O) δ 174.8, 174.7, 174.6,102.4, 102.0, 101.9, 101.8, 99.1, 98.9, 97.6, 92.2, 80.3, 80.2, 79.4, 77.1, 75.6, 74.4, 74.3, 74.2, 73.9, 73.8, 71.9, 71.6, 70.4, 70.3, 69.7, 69.6, 69.5, 69.3, 68.7, 68.5, 68.3, 66.1, 66.0, 65.8, 54.2, 54.1, 53.9, 53.8; HRMS (ESI): *m/z* calcd for $C_{96}H_{150}O_{103}N_8S_8$ $[M + 11H]^{4-}$: 830.1140, found: 830.1155.

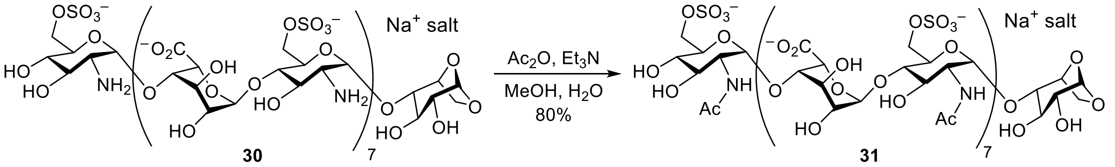

HS-based hexadecasaccharide 31. Compound **30** (12.4 mg, 3.39 μmol) was dissolved in H_2O/MeOH (1/1, 1 mL), and Et_3N (75.6 μL, 0.543 mmol) and Ac_2O (25.6 μL, 0.271 mmol) were added to it. The mixture was allowed to stir for 24 h at room temperature. The solvent was evaporated *in vacuo*, and the residue was purified through a Sephadex G-10 column with water as eluent, followed by Na^+ exchange through AG 50W-X8 (Na^+ form) cation exchange resin. Lyophilization of the isolated material was performed to afford compound **31** (10.8 mg, 80%) as a white solid. ^1H NMR (600 MHz, D_2O) δ 5.68 (d, *J* = 1.7 Hz, 1H), 5.45 (s, 1H), 5.17–5.12 (m, 8H), 4.97 (d, *J* = 3.0 Hz, 7H), 4.93 (s, 2H), 4.76 (d, *J* = 2.9 Hz, 4H), 4.35–4.30 (m, 10H), 4.25–4.19 (m, 11H), 4.17 (d, *J* = 6.9 Hz, 4H), 4.13 (d, *J* = 6.1 Hz, 1H), 4.08–4.03 (m, 15H), 3.98–3.95 (m, 10H), 3.93 (d, *J* = 3.5 Hz, 1H), 3.89 (d, *J* = 5.1 Hz, 8H), 3.84 – 3.80 (m, 5H), 3.78–3.70 (m, 26H), 3.56 (t, *J* = 9.6 Hz, 1H), 2.02 (s, 3H), 2.01 (s, 6H), 2.00 (s, 15H); ^{13}C NMR (150 MHz, D_2O) δ 175.0, 174.9, 174.8, 174.7, 174.4, 174.4, 102.2, 101.9, 101.7, 99.0, 98.9, 94.4, 94.4, 85.2, 82.9, 80.4, 80.2, 79.6, 79.3, 78.2, 76.5, 76.3, 75.7, 75.1, 74.4, 74.3, 74.2, 74.0, 71.0, 70.8, 70.1, 69.9, 69.7, 69.6, 69.4, 69.2, 69.2, 68.9, 68.8, 67.8, 66.7, 66.3, 66.1, 65.9, 65.4, 60.7, 54.3, 53.6, 53.6, 53.4, 23.2, 21.9, 21.8; HRMS (ESI): *m/z* calcd for $C_{112}H_{165}O_{111}N_8S_8$ $[M + 10H]^{5-}$: 730.9067, found: 730.9037.

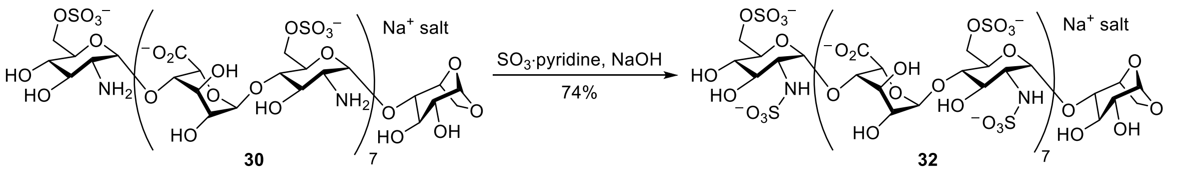

HS-based hexadecasaccharide 32. Compound **30** (12.8 mg, 3.50 μmol) was dissolved in H_2O (1 mL) and the pH of the solution was adjusted to within 9.5~10 by 1 M $NaOH_{(aq)}$. SO_3·pyridine (111 mg, 0.700 mmol) was added in five equal portions in half-hour intervals while keeping the pH of the reaction solution to within 9.5~10 using 1 M $NaOH_{(aq)}$. The mixture was allowed to further stir for 48 h at room temperature. The solvent was evaporated *in vacuo*, and the residue was purified through a Sephadex G-10 column with water as eluent, followed by Na^+ exchange through AG 50W-X8 (Na^+ form) cation exchange resin. Lyophilization of the isolated material was performed to afford compound **32** (11.6 mg, 74%) as a white solid. ^{1}H NMR (600 MHz, D_2O) δ 5.60 (d, *J* = 1.7 Hz, 1H), 5.32 (d, *J* = 3.8 Hz, 1H), 5.26–5.24 (m, 8H), 4.92 (s, 6H), 4.89 (s, 1H), 4.87 (s, 1H), 4.80 (s, 5H), 4.75 (s, 1H), 4.27–4.19 (m, 13H), 4.12–4.07 (m, 12H), 4.03 (m, 9H), 3.97 (s, 8H), 3.87 (d, *J* = 10.1 Hz, 8H), 3.80 (d, *J* = 10.8 Hz, 2H), 3.77 (s, 1H), 3.75 – 3.72 (m, 2H), 3.70 (s, 8H), 3.63 (d, *J* = 6.8 Hz, 9H), 3.59 – 3.53 (m, 11H), 3.46 (d, *J* = 9.4 Hz, 2H), 3.22 (dd, *J* = 9.1, 5.0 Hz, 1H), 3.15 (d, *J* = 10.9 Hz, 7H), 3.12 (d, *J* = 3.5 Hz, 1H); ^{13}C NMR (150 MHz, D_2O) δ 174.9, 102.1, 101.9, 101.8, 99.2, 98.9, 95.7, 95.6, 95.5, 95.5, 85.0, 82.9, 80.1, 80.0, 79.2, 78.4, 77.4, 77.3, 75.0, 74.5, 74.4, 73.9, 71.7, 71.1, 70.6, 69.9, 69.8, 69.8, 69.5, 69.1, 69.0, 68.6, 68.4, 68.3, 68.1, 67.6, 67.5, 67.4, 66.8, 66.4, 66.2, 65.3, 60.7, 57.9, 57.7; HRMS (ESI): *m/z* calcd for $C_{96}H_{139}O_{127}N_8S_{16}Na_8$ $[M + 8H + 8Na]^{7-}$: 590.1345, found: 590.1329.

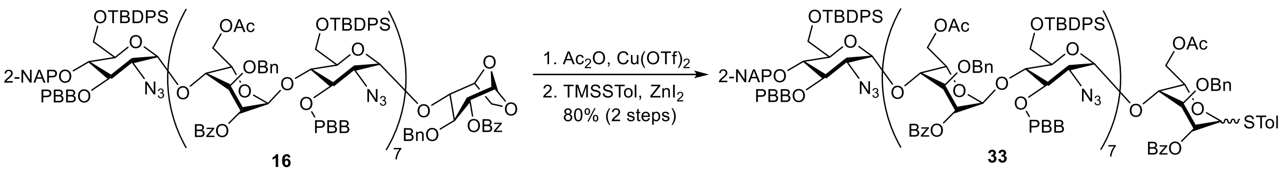

Compound 33. The hexadecasaccharide **16** (1.98 g, 0.246 mmol) was dissolved in Ac_2O (5.94 mL) under a nitrogen atmosphere, the reaction flask was immersed in an ice bath, and $Cu(OTf)_2$ (8.90 mg, 0.025 mmol) was added to the solution. After stirring for 18 h at 4 °C, the reaction was quenched by saturated $NaHCO_{3(aq)}$, and the mixture was extracted with ethyl acetate (3 × 50 mL). The combined organic layers were washed with brine, dried over anhydrous $MgSO_4$, filtered, and concentrated *in vacuo*. Purification of the residue via flash column chromatography (ethyl acetate/hexane = 1/4, v/v) provided the polyacetylated derivative.

To a solution of the polyacetylated derivative (1.95 g, 0.239 mmol) in dry CH_2Cl_2 (20 mL), TMSSTol (140 μL, 0.717 mmol) and ZnI_2 (229 mg, 0.717 mmol) were added. After 6 h of stirring at room temperature, the mixture was diluted with CH_2Cl_2 (100 mL). The organic layer was washed with saturated $NaHCO_{3(aq)}$, dried over anhydrous $MgSO_4$, filtered and concentrated *in vacuo*. Purification of the residue via flash column chromatography (ethyl acetate/hexane = 1/3, v/v) provided the thioglycoside **33** (1.62 g, 80% over two steps). $[\alpha]_D^{26}$ –30.5 (*c* 0.1, $CHCl_3$); IR (thin film) ν 3070, 3029, 2931, 285, 2109, 1746, 1721, 1601, 1588, 1489, 1471, 1453, 1428, 1391, 1367, 1317, 1268, 1241, 1157, 1112, 1070, 1042, 1028, 1007, 873, 822, 805, 754, 702, 613, 505, 489 cm^{-1}; ^{1}H NMR (600 MHz, $CDCl_3$) δ 8.07 (d, *J* = 7.9 Hz, 2 H), 7.97 (dd, *J* = 8.2, 2.9 Hz, 14 H), 7.85–7.81 (m, 1 H), 7.74 (d, *J* = 8.4 Hz, 1 H), 7.73–7.70 (m, 1 H), 7.64 (d, *J* = 7.9 Hz, 2 H), 7.61 (t, *J* = 8.6 Hz, 16 H), 7.57 (d, *J* = 8.0 Hz, 14 H), 7.53 (d, *J* = 6.0 Hz, 2 H), 7.49 (dd, *J* = 6.4, 3.1 Hz, 2 H), 7.44 (dd, *J* = 8.0, 3.4 Hz, 4 H), 7.41–7.27 (m, 64 H), 7.25–7.13 (m, 52 H), 7.12–7.08 (m, 4 H), 6.97 (d, *J* = 8.2 Hz, 2 H), 6.87 (t, *J* = 6.4 Hz, 12 H), 6.82 (d, *J* = 8.1 Hz, 2 H), 5.50 (s, 1 H), 5.37 (d, *J* = 8.8 Hz, 6 H), 5.31 (d, *J* = 7.6 Hz, 2 H), 5.11

(s, 7 H), 4.94 (d, J = 11.6 Hz, 1 H), 4.84–4.81 (m, 4 H), 4.76 (d, J = 11.3 Hz, 6 H), 4.68 (t, J = 12.4 Hz, 2 H), 4.63–4.56 (m, 13 H), 4.51 (d, J = 3.7 Hz, 1 H), 4.46 (d, J = 4.0 Hz, 6 H), 4.41 (d, J = 3.8 Hz, 1 H), 4.29 (t, J = 10.5 Hz, 3 H), 4.23–4.18 (m, 6 H), 4.10–4.02 (m, 10 H), 3.99 (d, J = 9.3 Hz, 8 H), 3.95–3.92 (m, 13 H), 3.87–3.78 (m, 15 H), 3.73 (t, J = 9.3 Hz, 2 H), 3.65 (q, J = 11.3 Hz, 7 H), 3.60 (d, J = 9.4 Hz, 1 H), 3.56 (d, J = 9.9 Hz, 1 H), 3.47 (t, J = 10.4 Hz, 14 H), 3.41 (d, J = 9.5 Hz, 1 H), 3.35 (s, 1 H), 3.33 (s, 4 H), 3.24 (dd, J = 10.0, 2.8 Hz, 1 H), 3.16 (dd, J = 10.2, 3.7 Hz, 1 H), 3.11 (d, J = 9.8 Hz, 6 H), 2.32 (s, 3 H), 1.84 (s, 3 H), 1.66 (s, 3 H), 1.59 (s, 18 H), 1.04 (s, 9 H), 0.98 (s, 54 H), 0.95 (s, 9 H); ^{13}C NMR (150 MHz, $CDCl_3$) δ 170.2, 170.0, 169.9, 165.8, 165.6, 165.5, 137.8, 137.4, 137.3, 137.3, 137.0, 136.9, 136.8, 135.9, 135.9, 135.9, 135.6, 135.5, 133.4, 133.4, 133.3, 133.2, 133.1, 132.9, 132.9, 132.4, 131.6, 131.5, 130.9, 130.9, 129.9, 129.8, 129.7, 129.7, 129.6, 129.6, 129.5, 129.0, 128.9, 128.8, 128.8, 128.6, 128.6, 128.4, 128.4, 128.4, 128.3, 128.2, 128.1, 128.1, 127.9, 127.8, 127.7, 127.6, 127.6, 127.6, 127.5, 127.4, 126.4, 126.2, 126.0, 125.8, 121.7, 121.0, 98.4, 97.9, 97.0, 96.9, 86.2, 80.4, 79.2, 78.9, 77.7, 77.2, 77.0, 76.8, 75.2, 74.3, 74.2, 74.0, 73.8, 73.1, 72.9, 72.9, 72.8, 72.7, 72.5, 72.3, 72.0, 71.5, 69.8, 68.8, 68.7, 68.4, 66.1, 65.1, 64.8, 64.5, 64.2, 64.0, 62.9, 62.3, 62.1, 26.9, 26.9, 26.8, 26.8, 22.7, 21.1, 20.6, 20.6, 20.5, 19.4, 19.4, 19.4; HRMS (MALDI): m/z calcd for $C_{426}H_{448}O_{88}N_{24}Br_8Si_8SNa$ $[M + Na]^+$: 8231.3408, found: 8231.3366.

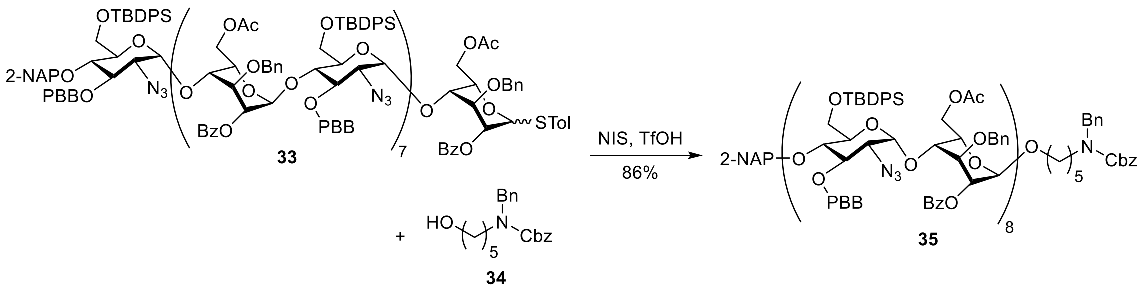

Compound 35. To a solution of thioglycoside **33** (1.60 g, 0.195 mmol) and alcohol **34** (95.8 mg, 0.292 mmol) in dry CH_2Cl_2 (16.1 mL), flame dried 3 Å MS (1.60 g) were added and stirred at room temperature for 1 h under a nitrogen atmosphere. The reaction mixture was cooled to –40 °C, NIS (54.8 mg, 0.244 mmol) and TfOH (3.44 µL, 0.039 mmol) was added dropwise to the reaction mixture and the solution was gradually warmed up to –20 °C. After stirring for 4 h, the reaction was quenched with Et_3N. The reaction mixture was filtered through a pad of Celite and dissolved in CH_2Cl_2 and washed with 20% $Na_2S_2O_{3(aq)}$ and saturated $NaHCO_{3(aq)}$ solution, water, and brine. The organic solution was dried over anhydrous $MgSO_4$, filtered, and concentrated under reduced pressure. The residue was purified by flash column chromatography (ethyl acetate/hexane = 1/3) on silica gel to get the linker attached hexadecasaccharide **35** (1.41 g, 86%) as white solid. $[\alpha]_D^{27}$ –43.5 (c 0.1, $CHCl_3$); IR (thin film) ν 3031, 2932, 2859, 2109, 1745, 1722, 1602, 1489, 1454, 1428, 1367, 1317, 1268, 1240, 1157, 1112, 1070, 1028 cm^{-1}; ^{1}H NMR (600 MHz, $CDCl_3$) δ 8.10–8.06 (m, 2H), 7.98–7.94 (m, 14H), 7.85–7.81 (m, 1H), 7.74 (d, J = 8.4 Hz, 1H), 7.73–7.71 (m, 1H), 7.65–7.53 (m, 44H), 7.50–7.48 (m, 2H), 7.39–7.29 (m, 72H), 7.24–7.14 (m, 58H), 6.97 (d, J = 8.2 Hz, 2H), 6.88–6.86 (m, 12H), 5.38–5.36 (m, 6H), 5.32 (s, 1H), 5.17 (s, 1H), 5.13–5.10 (m, 7H), 5.08 (s, 1H), 4.92 (d, J = 12.8 Hz, 1H), 4.84–4.82 (m, 3H), 4.79 (s, 1H), 4.76 (d, J = 10.8 Hz, 6H), 4.67 (d, J = 11.3 Hz, 2H), 4.63–4.56 (m, 17H), 4.47–4.45 (m, 7H), 4.33–4.22 (m, 9H), 4.08–3.91 (m, 38H), 3.88–3.80 (m, 14H), 3.73–3.61 (m, 10H), 3.59–3.54 (m, 2H), 3.53–3.45 (m, 14H), 3.37–3.33 (m, 6H), 3.25–3.22 (m, 1H), 3.21–3.15 (m, 2H), 3.14–3.09 (m, 7H), 1.82–1.80 (m, 4H), 1.68–1.65 (m, 2H), 1.66 (s, 3H), 1.61 (s, 6H), 1.59 (s, 15H), 1.04 (s, 9H), 0.98 (s, 54H), 0.95 (s, 9H); ^{13}C NMR (150 MHz, $CDCl_3$) δ 170.4, 170.2, 170.0, 165.9, 165.8, 165.7, 156.8, 156.3, 138.1, 137.9, 137.5, 137.4, 137.4, 137.2, 136.9, 136.1, 136.1, 136.0, 135.7, 135.6, 133.6, 133.5, 133.4, 133.2, 133.1, 133.1, 131.6, 131.4, 131.1, 130.4, 130.0, 129.9, 129.9, 129.8, 129.7, 129.7, 129.2, 129.2, 129.1, 128.9, 128.9, 128.8, 128.7, 128.7,

128.6, 128.5, 128.4, 128.4, 128.3, 128.2, 128.0, 128.0, 128.0, 127.9, 127.8, 127.7, 127.7, 127.6, 126.5, 126.3, 126.2, 125.9, 125.4, 121.8, 121.2, 98.6, 98.3, 97.6, 97.2, 97.1, 96.3, 80.5, 79.2, 79.0, 75.3, 74.3, 74.2, 73.9, 73.8, 73.1, 73.0, 72.9, 72.8, 72.6, 72.4, 72.1, 70.4, 69.3, 68.8, 68.6, 68.2, 67.3, 65.7, 65.3, 65.2, 65.0, 64.6, 64.3, 64.1, 63.5, 62.8, 62.3, 50.6, 50.3, 47.3, 46.3, 32.1, 29.9, 28.1, 27.7, 27.0, 27.0, 26.9, 23.6, 22.8, 20.8, 20.7, 19.6, 19.5, 19.5, 19.3; HRMS (MALDI): *m/z* calcd for $C_{439}H_{465}O_{91}N_{25}Br_8Si_8Na$ [M + Na]$^+$: 8434.5605, found: 8434.5327.

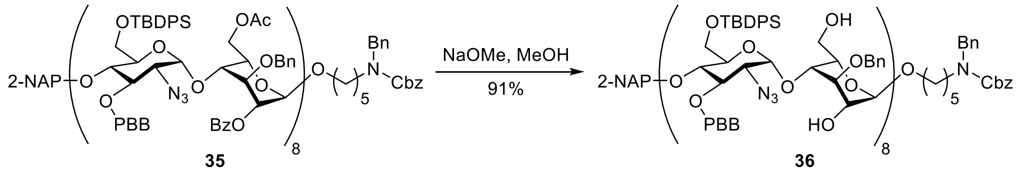

35 **36**

Compound 36. Sodium methoxide in MeOH (1 M, 2.50 mL) was added to a solution of the fully protected hexadecasaccharide **35** (1.10 g, 0.131 mmol) in THF (5 mL) at room temperature under a nitrogen atmosphere. After stirring for 1 h, the reaction solution was neutralized by DOWEX 50WX4-200 acidic resin. The resulting mixture was filtered through paper, and the filtrate was concentrated *in vacuo* to yield the crude product. Purification by precipitation in cold MeOH provided the polyol **36** (862 mg, 91%). $[\alpha]_D^{28}$ +30.0 (*c* 0.1, THF); IR (thin film) *ν* 3516, 2931, 2857, 2112, 1471, 1428, 1258, 1157, 1104, 1034, 1002 cm^{-1}; ^{1}H NMR (600 MHz, $CDCl_3$) δ 7.80–7.74 (m, 28H), 7.73–7.69 (m, 7H), 7.68–7.66 (m, 2H), 7.61–7.59 (m, 1H), 7.51–7.47 (m, 2H), 7.45–7.39 (m, 61H), 7.36–7.32 (m, 23H), 7.31–7.28 (m, 10H), 7.23–7.20 (m, 7H), 7.15–7.12 (m, 6H), 7.10–7.08 (m, 10H), 6.98–6.94 (m, 14H), 5.28–5.25 (m, 5H), 5.24 (s, 1H), 5.20–5.15 (m, 2H), 5.09–4.96 (m, 11H), 4.92–4.87 (m, 2H), 4.83 (d, *J* = 11.0 Hz, 1H), 4.79–4.71 (m, 10H), 4.67–4.64 (m, 1H), 4.60–4.52 (m, 10H), 4.49–4.45 (m, 4H), 4.43–4.37 (m, 12H), 4.33–4.21 (m, 1H), 4.13–4.10 (m, 7H), 4.08–4.04 (m, 6H), 4.00 (d, *J* = 11.7 Hz, 1H), 3.95–3.92 (m, 7H), 3.85–3.81 (m, 31H), 3.68–3.66 (m, 2H), 3.60–3.52 (m, 32H), 3.47–3.42 (m, 3H), 3.37–3.32 (m, 6H), 3.28–3.13 (m, 1H), 3.04–2.96 (m, 7H), 1.65–1.42 (m, 4H), 1.33–1.30 (m, 2H), 1.09 (s, 45H), 1.08 (s, 9H), 1.08–1.07 (m, 18H); ^{13}C NMR (150 MHz, $CDCl_3$) δ 156.5, 137.9, 137.4, 136.7, 136.2, 136.1, 136.0, 135.9, 135.8, 135.5, 133.5, 133.4, 133.1, 131.9, 131.7, 131.5, 131.4, 130.3, 129.9, 129.9, 129.8, 129.7, 129.4, 129.2, 128.8, 128.7, 128.7, 128.6, 128.5, 128.4, 128.1, 128.0, 127.9, 127.7, 127.7, 127.5, 127.3, 126.4, 126.3, 126.2, 126.1, 125.7, 122.0, 121.7, 101.0, 100.0, 94.8, 94.3, 81.3, 80.2, 78.1, 75.3, 75.1, 74.5, 73.0, 72.7, 72.5, 72.5, 72.3, 72.0, 71.7, 70.3, 69.9, 68.1, 67.4, 67.0, 66.3, 66.2, 65.9, 64.6, 64.0, 62.6, 62.2, 61.5, 58.5, 50.6, 50.4, 47.5, 46.2, 29.8, 29.1, 27.0, 25.7, 23.3, 19.6, 18.5; HRMS (MALDI): *m/z* calcd for $C_{367}H_{417}O_{75}N_{25}Br_8Si_8Na$ [M + Na]$^+$: 7265.3940, found: 7265.3960.

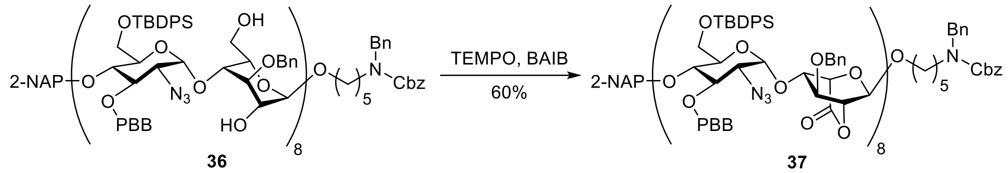

36 **37**

Compound 37. To a solution of the polyol **36** (850 mg, 0.117 mmol) in a THF and CH_2Cl_2 [1/2 (v/v), 3 mL] mixed solvent, TEMPO (9.14 mg, 0.058 mmol) and BAIB (756 mg, 2.35 mmol) were added at room temperature. After stirring for 48 h, ethyl acetate was added to the solution, and the mixture was sequentially washed with 10% $Na_2S_2O_{3(aq)}$, saturated $NaHCO_{3(aq)}$, and brine. The organic layer was dried over anhydrous $MgSO_4$, filtered and concentrated *in vacuo* to get the crude product, which was purified by flash column chromatography (ethyl acetate/hexane = 1/4, v/v) on silica gel to furnish the octalactone **37** (506 mg, 60%). $[\alpha]_D^{27}$ –4.50 (*c* 0.1, $CHCl_3$); IR (thin film) *ν* 3070, 2930, 2858, 2109, 1791, 1698, 1590, 1488, 1471, 1428, 1367, 1316, 1259, 1154, 1113, 1084, 1071, 1037 cm^{-1}; ^{1}H NMR (600 MHz, $CDCl_3$) δ

7.85–7.80 (m, 1H), 7.80–7.55 (m, 34H), 7.52–7.26 (m, 116H), 7.25–7.10 (m, 18H), 5.46 (s, 6H), 5.44 (s, 2 H), 5.20–5.14 (m, 2H), 5.10–5.02 (m, 2H), 5.01–4.86 (m, 8H), 4.81 (d, J = 10.9 Hz, 1H), 4.78–4.66 (m, 4H), 4.66–4.41 (m, 23H), 4.38–4.24 (m, 9H), 4.23–4.11 (m, 13H), 4.08–4.04 (m, 7H), 3.98 (d, J = 10.8 Hz, 2H), 3.95–3.78 (m, 13H), 3.77–3.58 (m, 30H), 3.55–3.50 (m, 7H), 3.42–3.12 (m, 11H), 1.68–1.59 (m, 4H), 1.31–1.28 (m, 2H), 1.10–1.07 (m, 54H), 1.05 (s, 9H), 1.04 (s, 9H); ^{13}C NMR (150 MHz, $CDCl_3$) δ 167.4, 137.8, 137.4, 137.0, 136.1, 136.0, 135.7, 135.6, 133.7, 133.4, 133.1, 132.6, 131.7, 131.7, 131.7, 131.4, 130.1, 129.9, 128.7, 128.7, 128.6, 128.6, 128.4, 128.3, 128.3, 128.2, 128.1, 128.0, 127.9, 127.8, 127.7, 127.3, 126.3, 126.1, 125.6, 122.0, 121.3, 99.5, 97.2, 80.8, 80.6, 79.9, 79.5, 78.5, 78.4, 78.2, 78.0, 76.5, 75.2, 74.9, 73.7, 72.8, 72.5, 72.4, 72.2, 70.4, 69.8, 69.6, 69.3, 68.1, 67.3, 65.3, 65.2, 63.5, 63.3, 61.9, 61.5, 50.4, 47.3, 32.1, 29.8, 27.0, 23.3, 22.8, 19.5, 19.4, 19.4; HRMS (MALDI): *m/z* calcd for $C_{367}H_{385}O_{75}N_{25}Br_8Si_8Na$ [M + Na]$^+$: 7233.1387, found: 7233.1350.

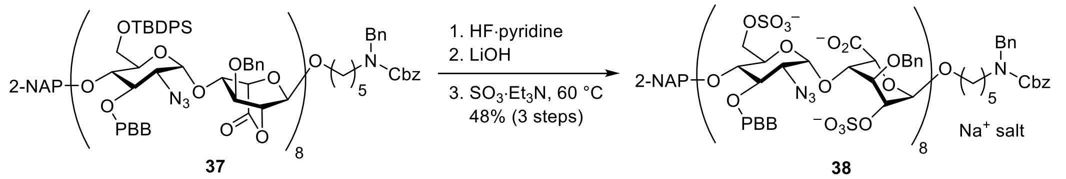

Compound 38. (1) *Desilylation.* HF·pyridine complex (2 mL) was added to a solution of the octalactone **37** (490 mg, 0.068 mmol) in THF and pyridine (1/1 (v/v), 8 mL) mixed solvent at 0 °C. After stirring for 48 h at room temperature, the reaction mixture was quenched with silica gel and filtered through a Celite bed. The filtrate was concentrated *in vacuo* to afford the desilylated crude product. HRMS (MALDI): *m/z* calcd for $C_{239}H_{241}O_{75}N_{25}Br_8Na$ [M + Na]$^+$: 5325.8936, found: 5325.8781.

(2) *Lactone ring opening.* An aqueous solution of LiOH (2.70 mL, 0.5 M) was added to a solution of the desilylated compound (350 mg, 0.066 mmol) in THF/MeOH (3/1 (v/v), 2 mL) at room temperature. The mixture was kept stirring for 3 h, and the reaction solution was neutralized by DOWEX 50WX4-200 acidic resin. The resulting mixture was filtered through paper, and the filtrate was concentrated *in vacuo* to yield the corresponding carboxylic acid derivative. HRMS (MALDI): *m/z* calcd for $C_{239}H_{257}O_{83}N_{25}Br_8Na$ [M + Na]$^+$: 5461.9517, found: 5461.9457.

(3) *O-Sulfonation.* A solution of the carboxylic acid derivative (340 mg, 0.062 mmol) and sulfur trioxide-triethylamine complex (905 mg, 4.99 mmol) in DMF (2.8 mL) was kept stirring at 60 °C for 48 h under a nitrogen atmosphere. The reaction flask was cooled down to room temperature, a solution of $NaHCO_{3(aq)}$ (0.9 mL, 0.5 M) was added to the mixture and the resulting solution was kept stirring for another 2 h. The solvent was co-evaporated with MeOH under reduced pressure and a mixed solvent of CH_2Cl_2/MeOH [1/1 (v/v), 10 mL] was added to the solid mass. The mixture was filtered and the filtrate was concentrated *in vacuo*. The residue was layered on the top of a Sephadex LH-20 chromatography column, which was eluted with MeOH, followed by Na^+ exchange through AG 50W-X8 (Na^+ form) cation exchange resin to get compound **38** (219 mg, 48% over three steps). ^{1}H NMR (600 MHz, MeOD) δ 7.81–7.73 (m, 3H), 7.46–7.43 (m, 4H), 7.38–7.23 (m, 82H), 5.98 (s, 1H), 5.92 (s, 1H), 5.84–5.68 (m, 4H), 5.23–5.00 (m, 26H), 4.81–4.74 (m, 3H), 4.68–4.40 (m, 45H), 4.38–4.16 (m, 24H), 4.14–4.06 (m, 13H), 3.98–3.96 (m, 1H), 3.92 (t, J = 9.4 Hz, 1H), 3.86–3.73 (m, 6H), 3.67–3.45 (m, 12H), 3.17 (app s, 1H), 1.66–1.44 (m, 6H); ^{13}C NMR (150 MHz, CD_3OD) δ 175.0, 174.8, 174.1, 173.8, 160.1, 157.1, 156.6, 138.3, 138.0, 137.9, 137.7, 137.6, 137.5, 136.7, 135.8, 133.3, 133.1, 131.0, 130.8, 130.8, 130.7, 129.9, 129.5, 129.4, 129.3, 129.2, 129.2, 128.2, 128.0, 127.9, 127.9, 127.6, 127.5, 127.5, 127.4, 127.3, 127.2, 126.9, 126.7, 126.2, 125.6, 125.5, 120.6, 99.2, 94.4, 94.4, 93.5, 92.7, 92.6, 81.1, 77.9, 77.6, 76.2, 76.1, 75.4, 74.7, 73.8, 73.0, 72.5, 71.5, 71.4, 71.3, 71.1, 70.0, 69.7, 69.3, 69.2, 68.3, 68.3, 67.9, 67.6, 67.1, 65.5, 65.1, 64.8, 64.8, 64.6, 64.4,

63.5, 62.6, 50.1, 49.7, 46.2, 29.4, 27.8, 27.1, 22.3; ESI-MS: *m/z* calcd for $C_{239}H_{233}O_{131}N_{25}Br_8S_{16}Na_{20}$ [M - 4Na]$^{4-}$: 1787.7328, found: 1787.6953.

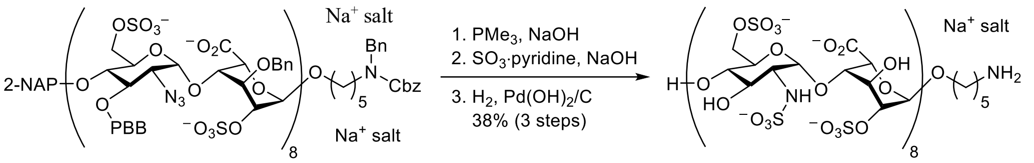

38 **39**

Compound 39. (1) *Azido reduction*. Compound **38** (210 mg, 0.031 mmol) was dissolved in THF (2 mL) and aqueous NaOH (0.2 mL 0.1 M) was added to it to make the final concentration 0.01 M. PMe_3 (1.0 M solution in THF, 1.30 mL) was added and the solution was stirred for 12 h at room temperature. The reaction mixture was neutralized with 0.1 M $HCl_{(aq)}$ and the solvent was evaporated under reduced pressure and the residue was purified by Sephadex LH-20 with MeOH as the eluent to obtain the polyamine. The amine compound was directly used for the next step.

(2) *N-Sulfonation*. To the solution of the amine derivative (201 mg, 0.031 mmol) in DMF (2 mL), SO_3·Pyridine (985 mg, 6.19 mmol), triethylamine (1.72 mL, 12.4 mmol) and 0.1 M $NaOH_{(aq)}$ (1.72 mL, 1 mL per mL of Et_3N) were added and stirred at room temperature for 48 h. After the full consumption of the starting material, the solvent was removed under reduced pressure and the residue was purified by Sephadex LH-20 using MeOH as an eluent to yield the *N*-sulfonated hexadecasaccharide (124 mg).

(3) *Hydrogenolysis*. A mixture of the *N*-sulfonated hexadecasaccharide (90 mg, 0.012 mmol) and 20% $Pd(OH)_2$ on carbon (540 mg) in phosphate buffer (20 mM, *p*H = 7.0) and MeOH mixed solvent (1/1 ratio, 4 mL) was equipped with a hydrogen balloon and the mixture was stirred at room temperature for 48 h. The whole mixture was filtered through Celite and the filtrate was concentrated *in vacuo.* The residue was purified through a Sephadex G-10 column with H_2O as eluent and passed through Na^+ exchange resin (AG 50W-X8, Na^+ form). Lyophilization of the isolated material afforded compound **39** (43.6 mg, 38% over three steps) as a white solid. ^{1}H NMR (600 MHz, D_2O) δ 5.48–5.36 (m, 8H), 5.24–5.18 (m, 7H), 5.09 (d, *J* = 3.0 Hz, 1H), 4.49 (d, *J* = 3.0 Hz, 1H), 4.43–4.37 (m, 7H), 4.36–4.32 (m, 8H), 4.30–4.24 (m, 8H), 4.23–4.17 (m, 11H), 4.12–4.06 (m, 10H), 4.05–3.97 (m, 8H), 3.80–3.74 (m, 9H), 3.70–3.63 (m, 10H), 3.60–3.53 (m, 2H), 3.30–3.25 (m, 7H), 3.23 (dd, *J* = 10.4, 3.5 Hz, 1H), 2.99 (t, *J* = 7.5 Hz, 2H), 1.69–1.62 (m, 4H), 1.48–1.44 (m, 2H).^{13}C NMR (150 MHz, D_2O) δ 174.8, 174.6, 174.5, 99.3, 99.2, 96.8, 96.5, 76.9, 76.0, 75.8, 73.4, 71.0, 69.9, 69.5, 69.5, 69.3, 69.2, 69.2, 69.1, 68.0, 66.4, 66.3, 64.1, 57.9, 39.4, 27.9, 26.2, 22.2; HRMS (ESI): *m/z* calcd for $C_{101}H_{152}O_{153}N_9S_{24}Na_8$ [M + 19H - 24Na]$^{5-}$: 977.9379, found: 977.9358.

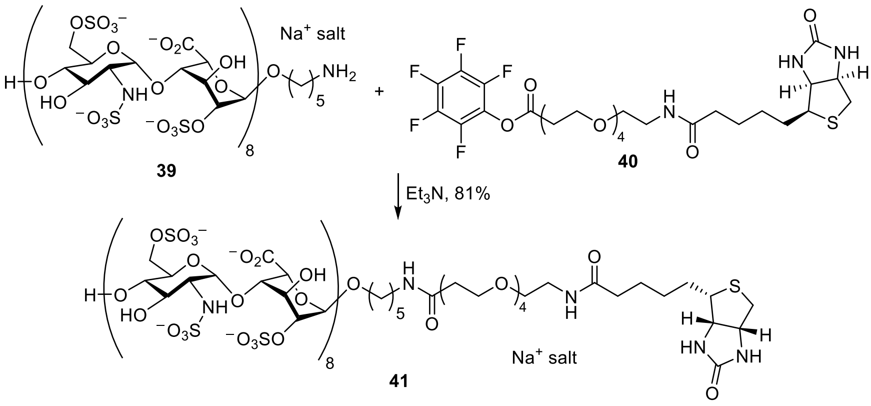

Biotinylated HS hexadecasaccharide 41. To a solution of compound **39** (5.12 mg, 944 μmol) in DMF/H_2O (1:1, 1.20 mL), triethylamine (25.6 μL, 5 μL/mg of **39**) and freshly prepared activated biotin ester **40** (76.8 μL, 15 μL/mg of **39**) were added and stirred for 48 h at room temperature. The solvent was evaporated under reduced pressure and the residue was purified through a Sephadex G-10 column with H_2O as eluent and passed through Na^+ exchange resin (AG 50W-X8, Na^+ form). Lyophilization of the isolated material was carried out to afford biotinylated hexadecasaccharide **41** (4.58 mg, 81%) as a white solid. ^{1}H NMR (600 MHz, D_2O) δ 5.41–5.37 (m, 8H), 5.28–5.18 (m, 7H), 5.11 (s, 1H), 4.62–4.59 (m, 2H), 4.54–4.51 (m, 1H), 4.48 (s, 1H), 4.42–4.39 (m, 4H), 4.35–4.32 (m, 6H), 4.28–4.25 (m, 4H), 4.20–4.18 (m, 6H), 4.11–3.98 (m, 11H), 3.78–3.73 (m, 10H), 3.70–3.67 (m, 30H), 3.64–3.62 (m, 6H), 3.59–3.56 (m, 3H), 3.40–3.38 (m, 5H), 3.35–3.32 (m, 2H), 3.28–3.21 (m, 5H), 3.19 (t, *J* = 6.2 Hz, 1H), 3.12–3.10 (m, 1H), 2.99 (d, *J* = 11.8 Hz, 2H), 2.92 (d, *J* = 11.6 Hz, 1H), 2.79–2.77 (m, 2H), 2.51 (t, *J* = 5.9 Hz, 2H), 2.46 (t, *J* = 8.0 Hz, 3H), 2.28 (t, *J* = 7.2 Hz,, 4H), 1.75–1.71 (m, 2H), 1.67–1.59 (m, 10H), 1.55–1.51 (m, 2H), 1.43–1.39 (m, 6H); ^{13}C NMR (150 MHz, D_2O) δ 180.2, 176.9, 174.6, 165.3, 163.1, 99.3, 96.5, 96.5, 75.9, 75.9, 69.6, 69.4, 69.2, 68.8, 67.9, 66.8, 66.3, 64.3, 63.1, 62.0, 60.2, 59.5, 57.9, 55.3, 55.1, 39.6, 39.3, 38.9, 37.6, 37.1, 36.1, 35.4, 27.9, 27.7, 25.1, 22.6, 22.5; HRMS (ESI): *m/z* calcd for $C_{126}H_{189}O_{162}N_{12}S_{25}Na_{11}$ [M + 13H - 21Na]$^{8-}$: 689.2357, found: 689.2367.

Experimental Procedures for Binding Assays and *Mtb* Detection

Preparation of the heparin-binding hemagglutinin full length and truncated form (full length HBHA and $HBHA_{110–199}$). The full length HBHA gene and truncated HBHA gene, which codes for residue 110–199, were amplified by PCR. The amplified HBHA fragments were then subcloned into a modified pET-based expression vector and transformed in *Escherichia coli* BL21 (DE3) cell. Protein expression was induced by isopropyl 1-thio-*β*-D-galactopyranoside at 37 °C for 3 h. Purification of the target protein from the supernatant following cell lysis and centrifugation was carried out through nickel-affinity and heparin-affinity chromatography. Cleavage of the N-terminal hexahistidine tag was accomplished by AcTEV or thrombin digestion.

ITC measurements. ITC experiments were carried out at 25 °C using Tris buffer (20 mM Tris, 100 mM NaCl, pH 7.6) as solvent. The $HBHA_{110–199}$ solution was placed in the calorimeter cell and subsequently titrated with the sugar solution (2 μL injections with 3 min spacing). ITC experiments were conducted using 90 μM concentrations of HS hexadecasaccharides, and **HS8**, with $HBHA_{110–199}$ concentrations of 240 μM for HS hexadecasaccharides and 750 μM for **HS8**. To account for the contribution of the heat of the dilution, the buffer without the protein was also titrated with the sugar solution and the generated data was subtracted from the results of the protein titration. Titration isotherms were fitted to the One Site model using the Malvern PEAQ ITC software, which assumes a 1:1 binding interaction. The fitting process estimated the dissociation constant (K_D), stoichiometry (n), binding enthalpy change (ΔH) binding entropy change (–TΔS), and binding free energy change (ΔG), and by minimizing the difference between experimental data and the model. K_D values were derived to characterize the binding affinity, with lower values indicating stronger interactions. Duplicate measurements were performed for each synthetic HS-based hexadecasaccharide, and the means are provided in Figures S1 to S3.

CD measurements. CD spectra were recorded on a Jasco J-715 CD spectropolarimeter, and the CD spectra were collected using a cylindrical quartz cuvette with a 1 mm path length. The step resolution was 0.2 nm with 1.0 nm bandwidth at a scan speed of 50 nm/min. Each CD spectrum was averaged over 16 measurements and corrected for the appropriate buffer baseline. The titration experiment involved the addition of various amounts of compound **24** to a fixed amount of $HBHA_{110–199}$. All spectra are presented as the molar CD absorption coefficient ($\Delta\varepsilon_M$) using the molar concentration of the protein to facilitate direct comparison of its free and bound form. The contents of secondary structures were analyzed by the Dichroweb program.[8,9] The concentration of $HBHA_{110–199}$ is 10 μM, and compound **24** ranged from 1 to 20 μM.

NMR spectroscopy and resonance assignments of free and bound $HBHA_{110-199}$. The ^{13}C, ^{15}N-labeled $HBHA_{110–199}$ protein[1] was dissolved in a solution containing 150 mM NaCl, 90% H_2O/10% D_2O and the pH was adjusted to 6.5. The final concentration of $HBHA_{110–199}$ was 0.4 mM for structure determination. $HBHA_{110–199}$-compound **24** complex was prepared as follows: To make as stock solution of compound **24**, compound **24** (1 mg) was dissolved into a 0.5-mL solution containing 90% H_2O/10% D_2O and 150 mM NaCl at pH 6.5. An appropriate amount of compound **24** stock solution was added dropwise into the $HBHA_{110–199}$ solution with gentle mixing to ensure complex formation. The final NMR sample solutions were transferred into 5 mm Shigemi NMR tubes. All NMR data were recorded at 280 K on Bruker AVANCE-600 spectrometer equipped with *xyz*-gradient TXI probe. All the spectra were processed by TOPSPIN and

analyzed by AURELIA. Proton chemical shifts were referenced to sodium trimethylsilylpropanesulfonate, and ^{13}C and ^{15}N chemical shifts were calibrated indirectly using the absolute frequency ratios.

Backbone resonance assignments of free $HBHA_{110–199}$ were obtained by correlating intra- and inter-residue through-bond connectivity of $^{13}C^{\alpha}$, $^{13}C^{\beta}$, and $^{13}C'$ in a series of standard two-dimensional and three-dimensional NMR spectra including $^{15}N–^{1}H$ HSQC, HNCO, HN(CA)CO, HNCA, HN(CO)CA, CBCANH, and CBCA(CO)NH. Assignments of H^{α} and H^{β} were obtained by HBHA(CACBCO)NH, HCCH–COSY and HCCH–TOCSY spectra. Resonance assignments of ^{15}N- and ^{13}C-labeled $HBHA_{110–199}$ when associated with compound **24** were first performed by comparing chemical shifts with those in free $HBHA_{110–199}$. Ambiguous chemical shifts were then obtained by correlating intra- and inter-residue resonances in standard three-dimensional NMR spectra using similar procedures as in the assignments for free $HBHA_{110–199}$. Chemical shifts were confirmed by inspection of intra-residue and sequential NOEs in ^{13}C, ^{15}N-edited NOESY–HSQC spectra.

Chemical shift deviation experiments. The binding site of compound **24** in $HBHA_{110–199}$ was identified using a chemical shift perturbation map. A single quantity used as chemical shift perturbation of each residue ($\Delta\delta_{residue}$) was expressed as equation 1.

$$\Delta\delta_{\text{residue}} = \left\{\frac{1}{3}\left[\left(\Delta\delta_{H^N}\right)^2 + \left(\frac{\Delta\delta_N}{10}\right)^2 + \left(\frac{\Delta\delta_{C'}}{4}\right)^2\right]\right\}^{\frac{1}{2}} \qquad \text{(equation 1)}$$

$\Delta\delta_{H^N}$, and $\Delta\delta_N$, and $\Delta\delta_{C'}$ represent the chemical shift difference of $HBHA_{110–199}$ upon association with compound **24** for the proton bound to backbone amide N, backbone amide N, and backbone carbonyl carbon, respectively. The binding interface of $HBHA_{110–199}$ was defined as those residues with significant chemical shift perturbations upon complex formation.

Titration and affinity measurements. ^{15}N-labeled $HBHA_{110–199}$ (200 μM) was titrated with aliquots of a stock solution of compound **24**. Overall, five two-dimensional $^{15}N–^{1}H$ HSQC titration spectra were recorded with molar ratio 1:0, 1:0.5, 1:1, 1:1.5, and 1:2, respectively. The chemical shift deviation ($\Delta\delta$) was normalized to the maximum chemical shift deviation ($\Delta\delta_{max}$). The normalized chemical shift deviation was found as a function of the concentration ratio of **24** to $HBHA_{110–199}$. The titration results were fitted to equation 2 to obtain the dissociation constant (K_D) using a regression algorithm in the program Origin 6.0, where $[P]_T$ was the total concentration of protein $HBHA_{110–199}$, $[L]_T$ was the total concentration of ligand **24**, [PL] was the concentration of $HBHA_{110–199}$-**24** complex, and K_D was the dissociation constant of $HBHA_{110–199}$-**24** complex.

$$\frac{\Delta\delta}{\Delta\delta_{\text{max}}} = \frac{[\text{PL}]}{[\text{P}]_\text{T}} = \frac{\left(1+\frac{[\text{L}]_\text{T}}{[\text{P}]_\text{T}}+\frac{K_\text{D}}{[\text{P}]_\text{T}}\right) - \sqrt{\left(1+\frac{[\text{L}]_\text{T}}{[\text{P}]_\text{T}}+\frac{K_\text{D}}{[\text{P}]_\text{T}}\right)^2 - 4\left(\frac{[\text{L}]_\text{T}}{[\text{P}]_\text{T}}\right)}}{2} \qquad \text{(equation 2)}$$

NMR spectroscopy and resonance assignments of free and bound compound 24. Resonance assignments of free and bound compound **24** were obtained by correlating intra- and inter-residue through-bond and through-space connectivity in a series of standard two-dimensional NMR spectra. The intra-residue spin systems of **24** were primarily identified from double quantum field (DQF)–COSY and $^{13}C–^{1}H$ HSQC spectra. The inter-residue sequential assignments were obtained by through-space connectivities in NOESY. DQF-COSY spectrum was used to extract three-bond coupling constants, $^{3}J_{HH}$. Dihedral angles within sugar residue were derived from $^{3}J_{HH}$ using the Karplus equation.[10]

Preparation of mycobacteria. Three *Mtb* isolates—Beijing, Haarlem, East African-Indian (EAI) (handled in biosafety level-2+ [BSL-2+] laboratories at Taiwan's National Health Research Institutes [NHRI])—and *M. bovis* BCG (handled in a BSL-2 laboratory at National Tsing Hua University [NTHU], Taiwan) were provided by NHRI under NHRI guidelines and upon NHRI ethics committee approval (IRB no. EC1060303-E & EC1090902-E). The isolates were collected and identified from clinical patients from Chest Hospital, Ministry of Health and Welfare, Tainan, Taiwan. Both species were used to ensure the wide applicability of the assay. The bacteria were incubated in Middlebrook (7H10) solid media broth (Difco, USA) and Middlebrook (7H9) broth.[11] The former was supplemented with 0.2% glycerol and autoclaved. Then, 10% oleic acid enriched with albumin, dextrose, and catalase (Becton-Dickinson, USA) was added prior to solidification in culture plates. 7H9 broth was prepared similarly, though with a different albumin+dextrose+catalase enrichment cocktail (Becton-Dickinson). The *Mtb* isolates were inoculated from a single colony into 10 mL of 7H9 broth under 37 °C for 2 weeks. The tested dead *M. bovis* BCG were cultured first in 7H9 broth and heated at 100 °C for 30 min as reported previously.[12]

PCR protocol. Thermo-Fisher Scientific's (TFS, USA) ProFlex^{TM} system was used for benchtop amplification of a conserved, 508-base pair region of the *rpo*B genes with the *M. bovis* BCG-specific *rpo*B primers (designed from NCBI accession AP010918 and synthesized by Protech, Taiwan): *rpo*BF (5'-GCTGGACATCTACCGCAAGCTGC-3') and *rpo*BR (5'-CAGCGGGTTGTTCTGGTCCATG-3'). The 20-μL reactions contained 10 μL of 2× KAPA SYBR® Fast qPCR mastermix (Roche, USA), 0.4 μL of DMSO, 0.3 μL of each primer, and bead+bacteria complexes (described below). Thermocycling was performed at 95 °C for 5 min followed by 35 cycles of 95 °C for 20 s, 58 °C for 20 s, and 72 °C for 20 s and a final extension at 72 °C for 10 min. On-chip PCRs (10 μL) featured half of the respective volumes. PCR products were electrophoresed at 100 V for 30 min on 2% agarose gels (in 0.5× TBE, Sigma-Aldrich), stained with 0.5 μg/mL ethidium bromide for 10 min, and imaged under a 254-nm ultraviolet transilluminator (Analytik Jena GmbH, Germany). Thermocycling for on-chip PCR was carried out using thermoelectric coolers placed underneath the microfluidic chip equipped with a thermocouple as feedback control.[12] The same thermoelectric cooler was also used for bacterial lysis.

Preparation of hexadecasaccharide 41-magnetic beads and mycobacterial capture. The biotin-labeled hexadecasaccharide **41** (100 μL of 10 μM solution in double distilled water) was incubated with streptavidin (C1)-coated MyOne^{TM} beads (900 μL of 4×10^8 beads/mL stock in double distilled water, 1 μm bead diameter, Invitrogen) overnight at room temperature on an ELMI RM-2L Intelli Mixer (Latvia) at 20 rpm (C2 mode). The bead-HS complexes were washed with water twice, concentrated by a magnet (DynaMag^{TM}-2 magnet rack, 3500-3700 Gauss, TFS), blocked with 1000 μL of 1% bovine serum albumin (Sigma-Aldrich, Inc., USA) in double distilled water at room temperature for 1 hour, washed twice with 1000 μL of double distilled water, collected, and concentrated via magnet, and resuspended in 1000 μL of double distilled water. The 10^4 bead-HS complexes were incubated with 10 CFU, 100 CFU, and 1,000 CFU of each bacterial strain for 30 min at room temperature. Then, propidium monoazide (Biotium, USA; stored in the dark at –20 °C) was mixed with bacteria-bead complexes under ultraviolet (254 nm) exposure (max power = 67 mW, SATIO-08mini/HV5-B, Saito Precision, Japan) for 30 min at room temperature. The bacteria-bead complexes were washed twice with water, suspended in double distilled water, and collected via magnet. Then, 10 μL of bacteria-bead complexes were lysed at 90 °C for 10–30 min to free the gDNA.

On-chip bacteria diagnosis. The entire process for diagnosis of *M. bovis* BCG and *Mtb* clinical isolates was automated using an integrated microfluidic system described previously.[13] The microfluidic chip was composed of polydimethylsiloxane liquid and air control layers and one glass substrate, all of which were bonded by oxygen plasma treatment. The 74 × 58 mm (length × width) chips were mass-produced by Jabil Circuit9 (Taiwan) via hot-embossing fabrication, and a home-made control system automated the entire process. Ten μL of bead-HS complex stock, 50 μL of wash buffer, and 20 μL of PCR reagents were first pre-loaded into their corresponding chambers. Then, 20 μL of the mycobacterial sample of the indicated CFU incubated with propidium monoazide was loaded onto the reaction chambers. The chip was exposed to ultraviolet light (254 nm) to trigger the propidium monoazide reaction. After incubation for 10 minutes, a magnet was placed underneath the microfluidic chip to collect bacteria-bead complexes. The wash buffer was pumped into the reaction chambers by the activation of transportation micropumps and normally closed micro-valves to wash out PCR inhibitors. Subsequently, the PCR reagents were pumped into the reaction chambers and the temperature of the microfluidic chip was regulated by the temperature control module of the integrated system to perform PCR. The bacteria were then disrupted at a high temperature of 95 °C during PCR, and the DNA released from the live bacteria was amplified. After 25 PCR cycles, the fluorescent signals emitted from the PCR products were detected by a laser-induced fluorescent module.

Statistical analysis. The fluorescence intensity of serially diluted tested samples was compared with the negative control among ten experiments in this study. Therefore, the two-tailed student t-test analysis was used to calculate whether there was a significant difference ($p < 0.05$) for limit-of-detection determination.

NMR, Mass, and IR Spectra of Key Compounds

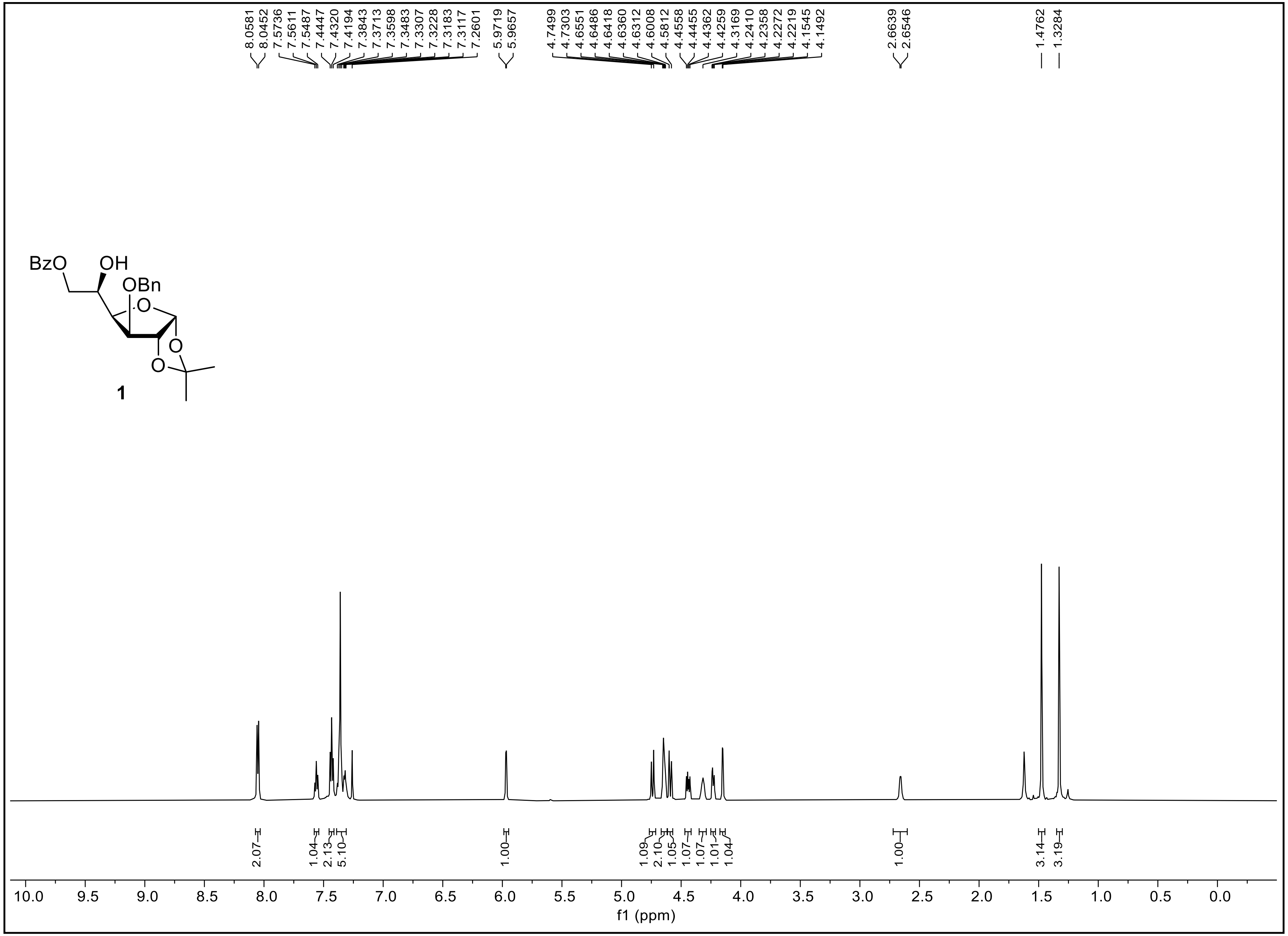
8.0581
8.0452
7.5736
7.5611
7.5487
7.4447
7.4320
7.4194
7.3843
7.3713
7.3598
7.3483
7.3307
7.3228
7.3183
7.3117
7.2601
5.9719
5.9657
4.7499
4.7303
4.6551
4.6486
4.6418
4.6360
4.6312
4.6008
4.5812
4.4558
4.4455
4.4362
4.4259
4.3169
4.2410
4.2358
4.2272
4.2219
4.1545
4.1492
2.6639
2.6546
1.4762
1.3284
BzO
OH
OBn
O
O
O
2.07
1.04
2.13
5.10
1.00
1.09
2.10
1.05
1.07
1.07
1.01
1.04
1.00
3.14
3.19
10.0
9.5
9.0
8.5
8.0
7.5
7.0
6.5
6.0
5.5
5.0
4.5
4.0
3.5
3.0
2.5
2.0
1.5
1.0
0.5
0.0
f1 (ppm)

1

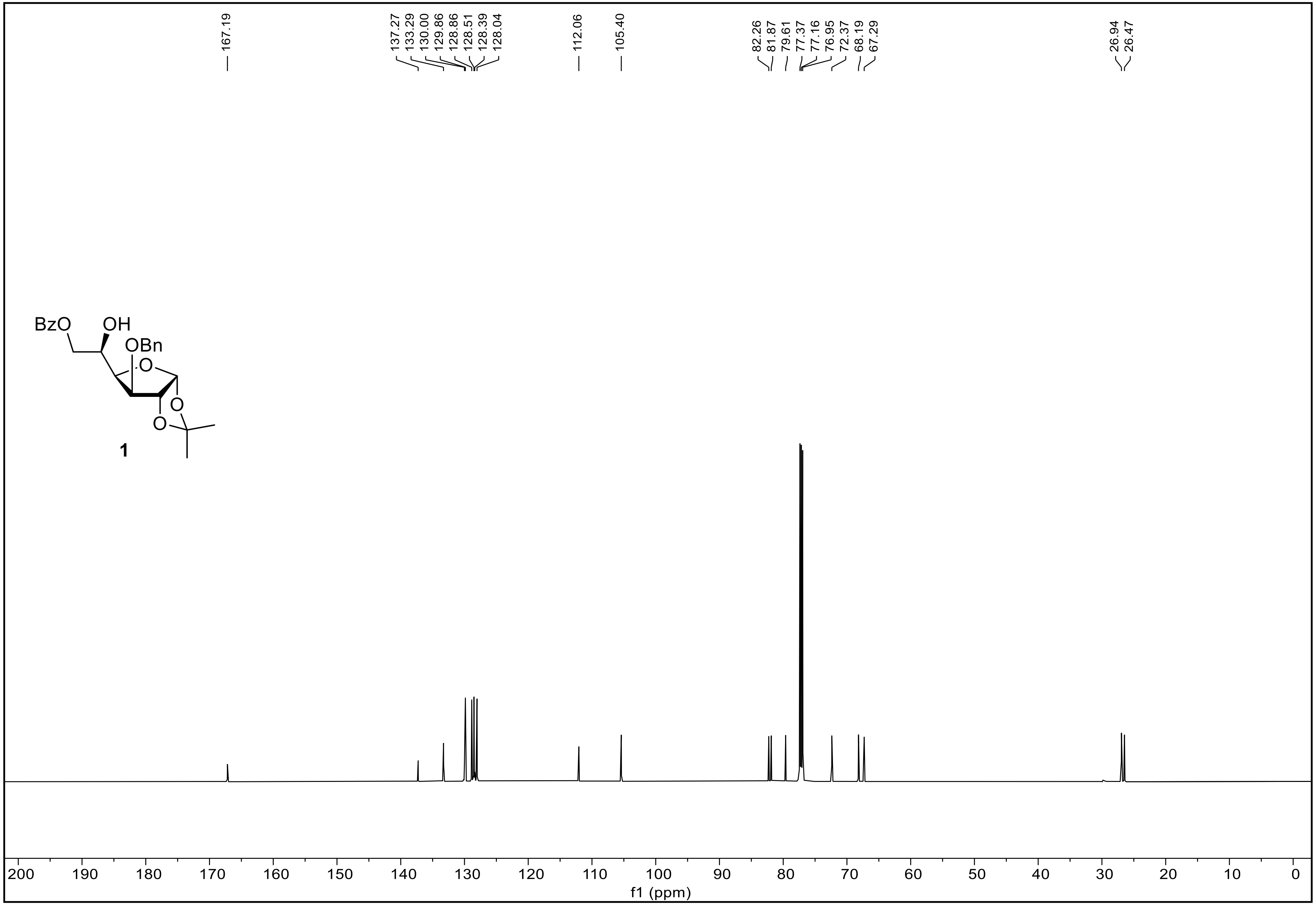
167.19
137.27
133.29
130.00
129.86
128.86
128.51
128.39
128.04
112.06
105.40
82.26
81.87
79.61
77.37
77.16
76.95
72.37
68.19
67.29
26.94
26.47
BzO
OH
OBn
O
O
O
1
200 190 180 170 160 150 140 130 120 110 100 90 80 70 60 50 40 30 20 10 0
f1 (ppm)

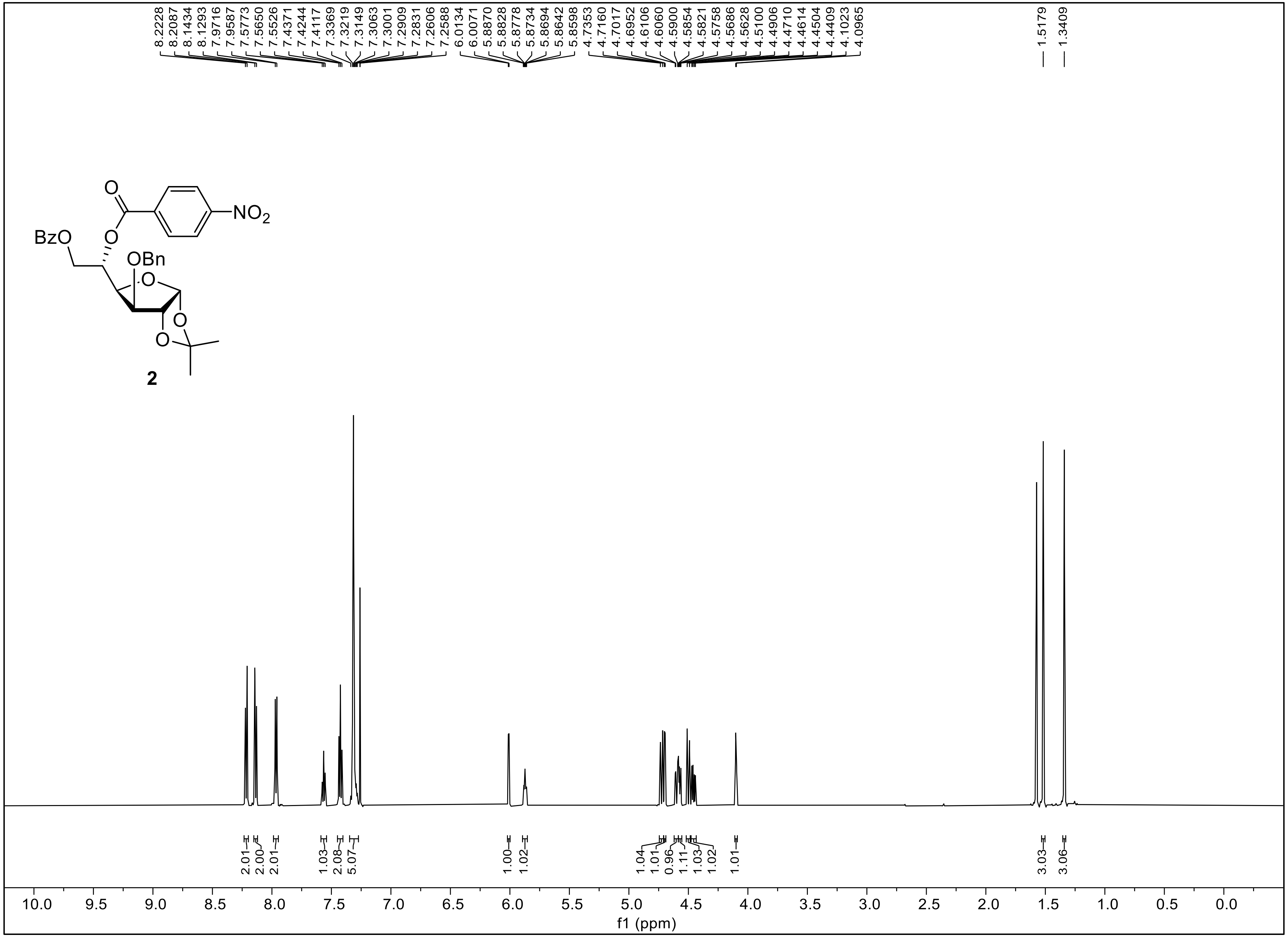
8.2228
8.2087
8.1434
8.1293
7.9716
7.9587
7.5773
7.5650
7.5526
7.4371
7.4244
7.4117
7.3369
7.3219
7.3149
7.3063
7.3001
7.2909
7.2831
7.2606
7.2588
6.0134
6.0071
5.8870
5.8828
5.8778
5.8734
5.8694
5.8642
5.8598
4.7353
4.7160
4.7017
4.6952
4.6106
4.6060
4.5900
4.5854
4.5821
4.5758
4.5686
4.5628
4.5100
4.4906
4.4710
4.4614
4.4504
4.4409
4.1023
4.0965
1.5179
1.3409
BzO
OBn
NO_2
2
2.01
2.00
2.01
1.03
2.08
5.07
1.00
1.02
1.04
1.01
0.96
1.11
1.03
1.02
1.01
3.03
3.06
10.0 9.5 9.0 8.5 8.0 7.5 7.0 6.5 6.0 5.5 5.0 4.5 4.0 3.5 3.0 2.5 2.0 1.5 1.0 0.5 0.0
f1 (ppm)

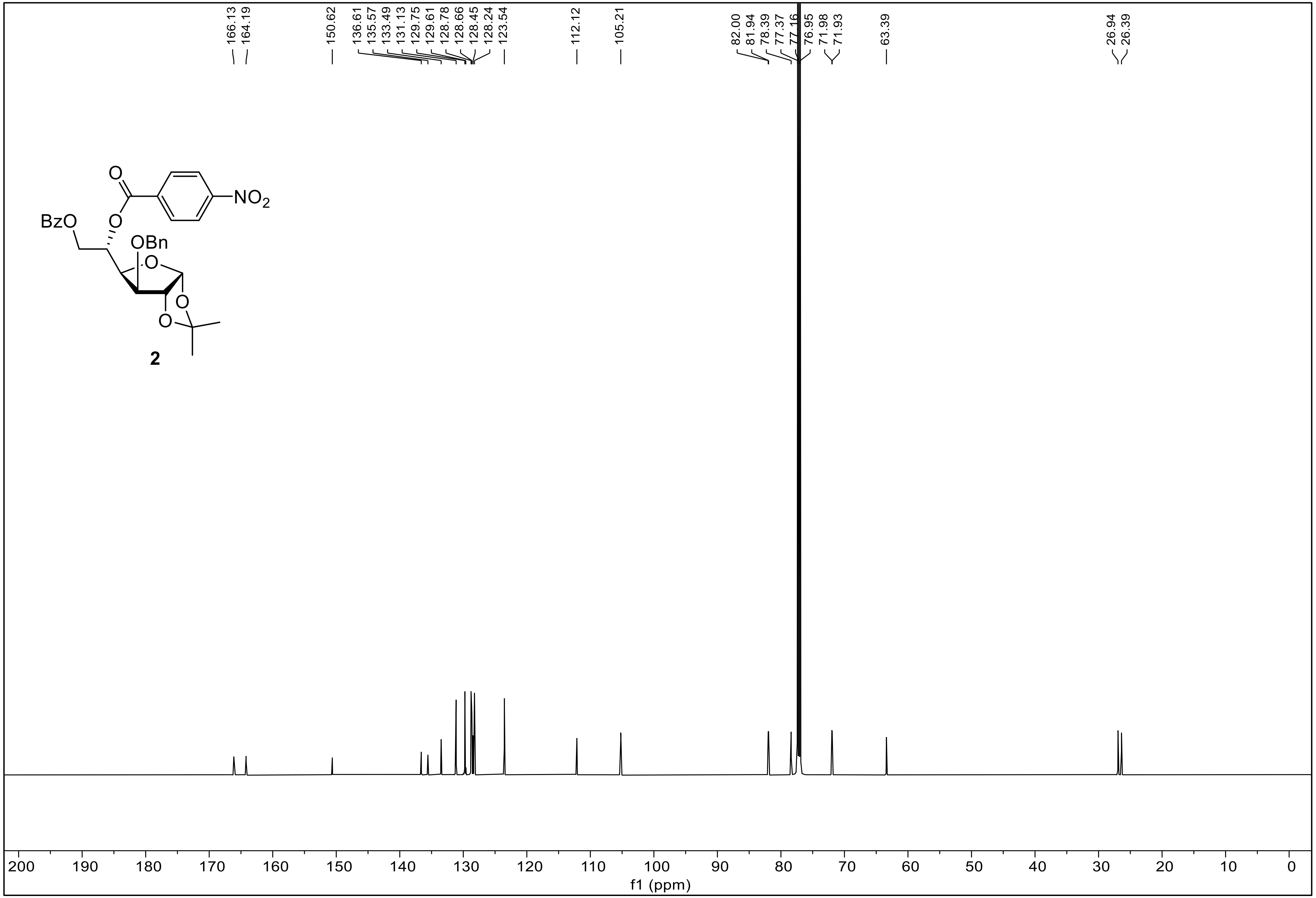
166.13
164.19
150.62
136.61
135.57
133.49
131.13
129.75
129.61
128.78
128.66
128.45
128.24
123.54
112.12
105.21
82.00
81.94
78.39
77.37
77.16
76.95
71.98
71.93
63.39
26.94
26.39
O
NO_2
BzO
O
OBn
O
O
O
2
200
190
180
170
160
150
140
130
120
110
100
90
80
70
60
50
40
30
20
10
0
f1 (ppm)

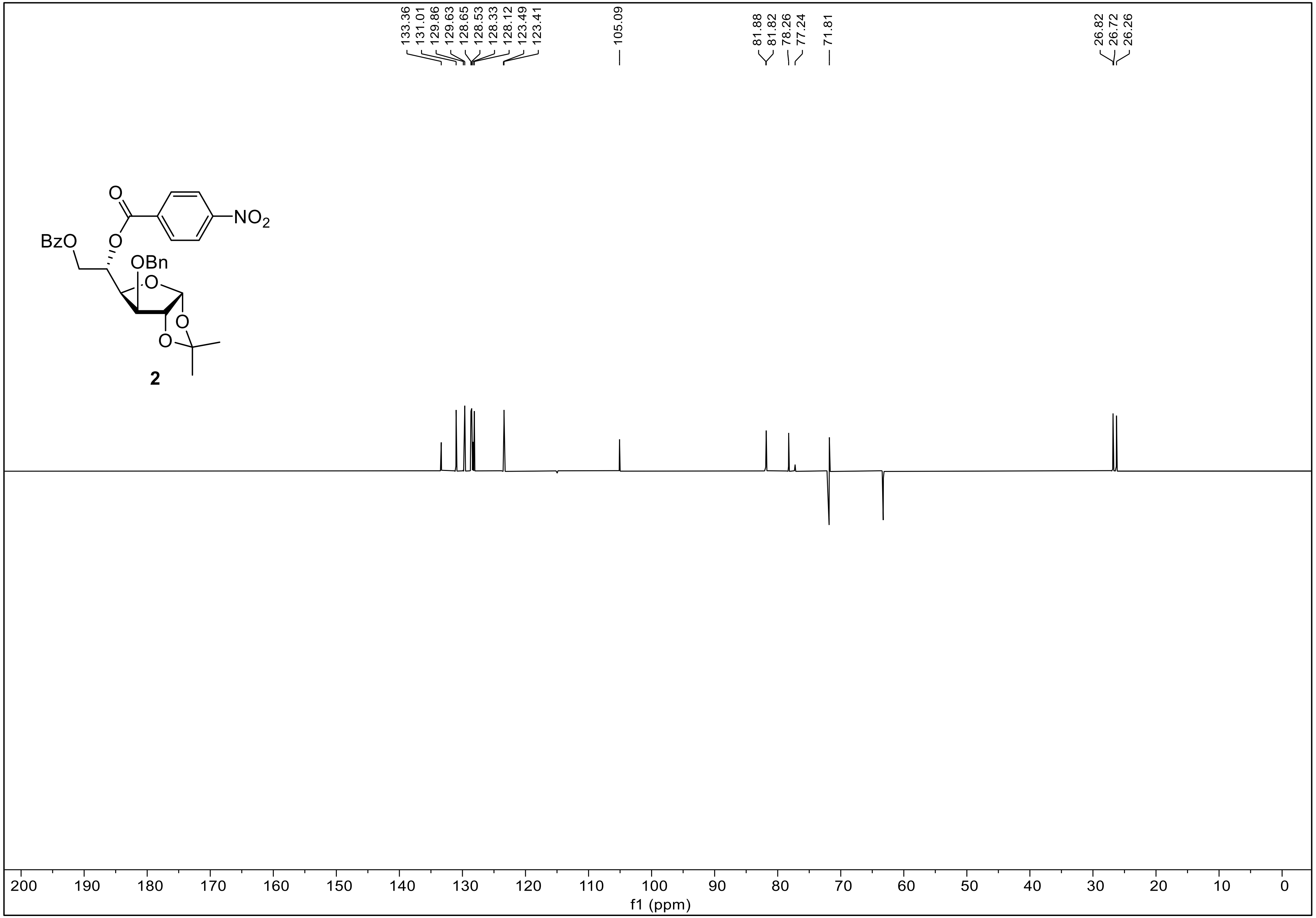
133.36
131.01
129.86
129.63
128.65
128.53
128.33
128.12
123.49
123.41
105.09
81.88
81.82
78.26
77.24
71.81
26.82
26.72
26.26
BzO
OBn
NO2
2
200 190 180 170 160 150 140 130 120 110 100 90 80 70 60 50 40 30 20 10 0
f1 (ppm)

^{1}H-^{1}H COSY

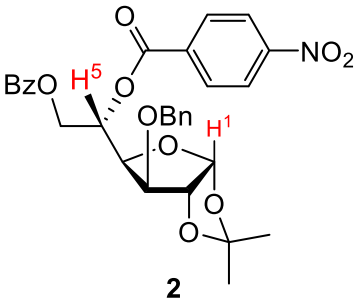

2

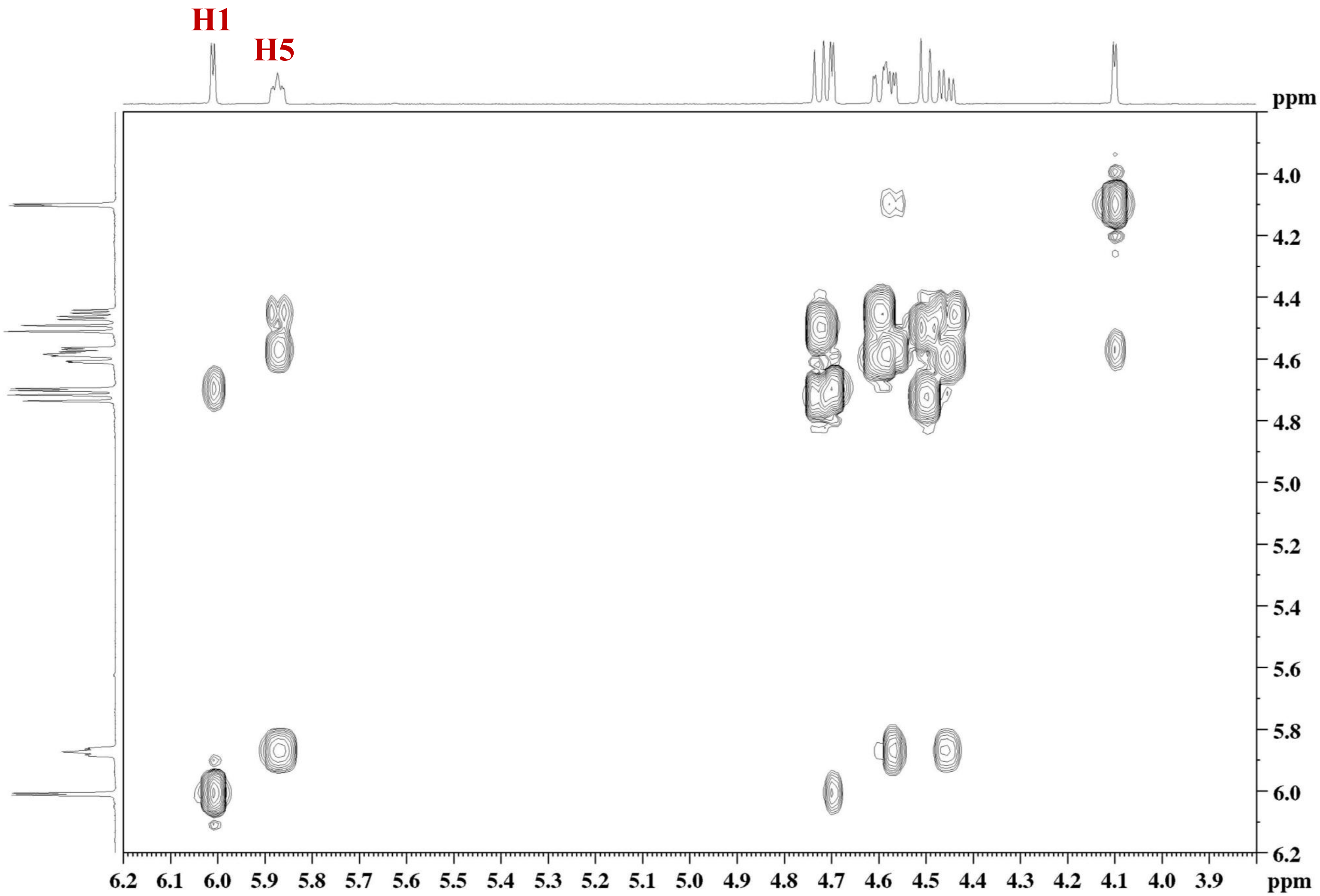

^1H-^{13}C HSQC

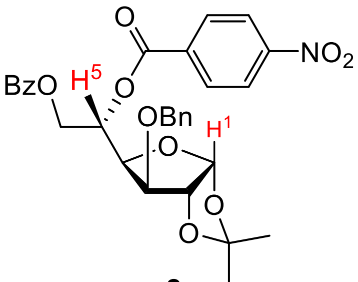
BzO
H5
O
NO2
OBn
H1
O
O
O

2

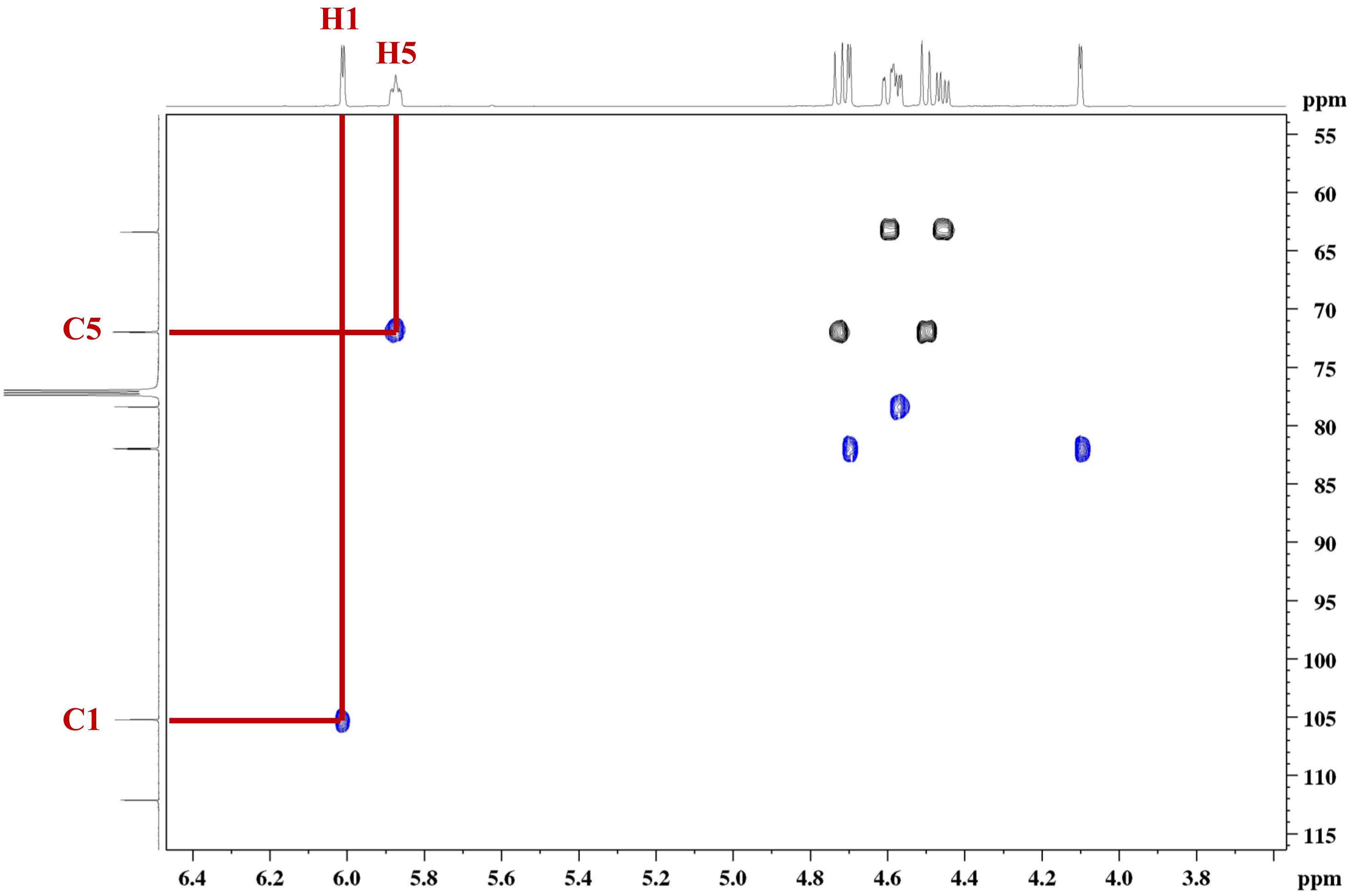
H1
H5
C5
C1
ppm
55
60
65
70
75
80
85
90
95
100
105
110
115
6.4
6.2
6.0
5.8
5.6
5.4
5.2
5.0
4.8
4.6
4.4
4.2
4.0
3.8
ppm

HRMS-ESI

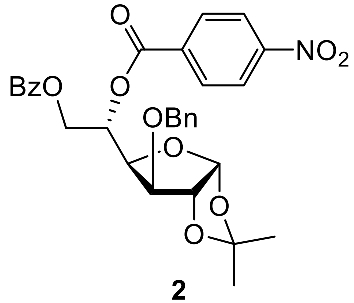

2

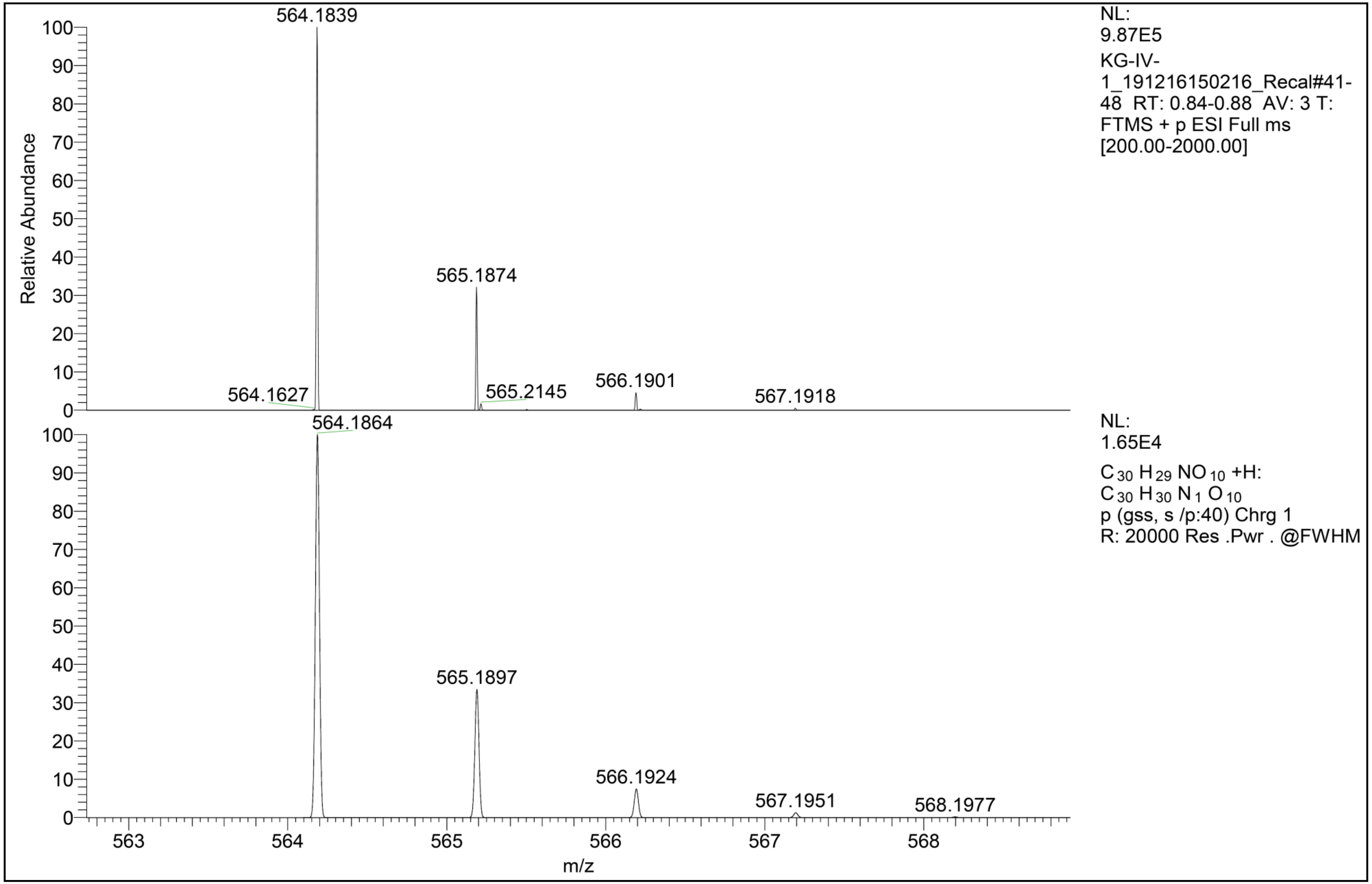

564.1839
565.1874
564.1627
565.2145
566.1901
567.1918
564.1864
565.1897
566.1924
567.1951
568.1977
Relative Abundance
m/z
NL:
9.87E5
KG-IV-1_191216150216_Recal#41-48 RT: 0.84-0.88 AV: 3 T: FTMS + p ESI Full ms [200.00-2000.00]
NL:
1.65E4
C 30 H 29 NO 10 +H:
C 30 H 30 N 1 O 10
p (gss, s /p:40) Chrg 1
R: 20000 Res .Pwr . @FWHM

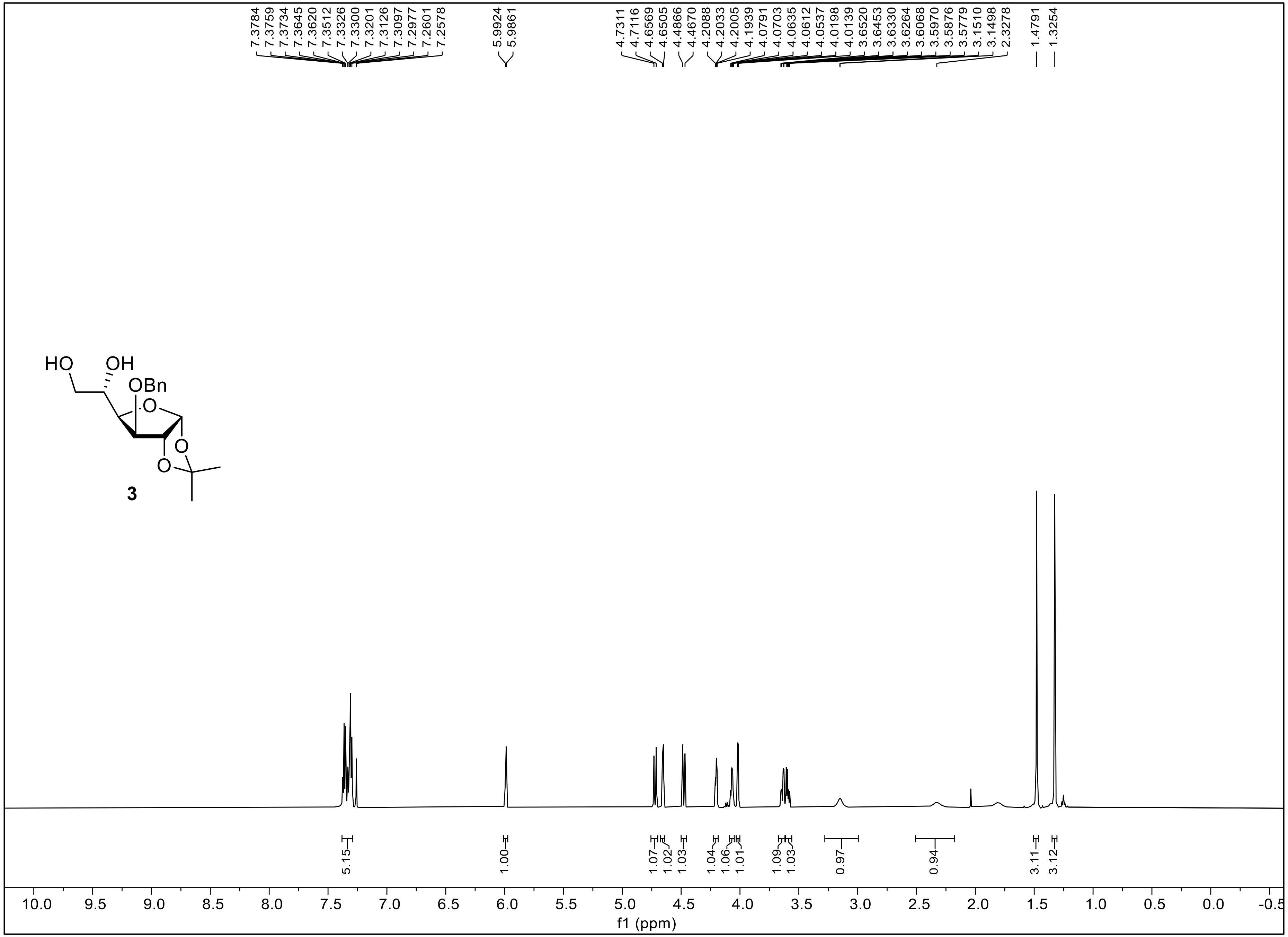
7.3784
7.3759
7.3734
7.3645
7.3620
7.3512
7.3326
7.3300
7.3201
7.3126
7.3097
7.2977
7.2601
7.2578
5.9924
5.9861
4.7311
4.7116
4.6569
4.6505
4.4866
4.4670
4.2088
4.2033
4.2005
4.1939
4.0791
4.0703
4.0635
4.0612
4.0537
4.0198
4.0139
3.6520
3.6453
3.6330
3.6264
3.6068
3.5970
3.5876
3.5779
3.1510
3.1498
2.3278
1.4791
1.3254
HO
OH
OBn
O
O
O
3
5.15
1.00
1.07
1.02
1.03
1.04
1.06
1.01
1.09
1.03
0.97
0.94
3.11
3.12
10.0
9.5
9.0
8.5
8.0
7.5
7.0
6.5
6.0
5.5
5.0
4.5
4.0
3.5
3.0
2.5
2.0
1.5
1.0
0.5
0.0
-0.5
f1 (ppm)

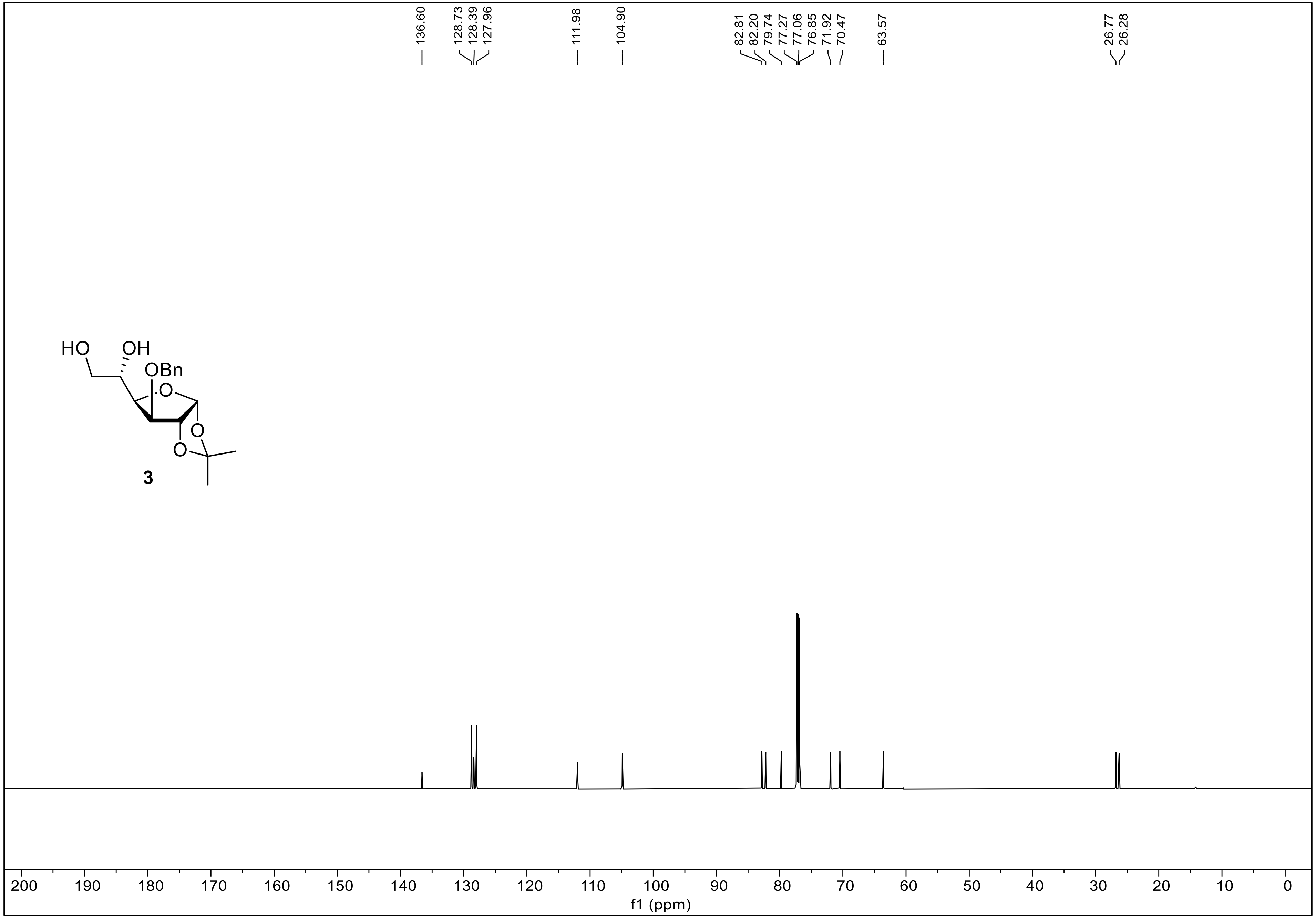
136.60
128.73
128.39
127.96
111.98
104.90
82.81
82.20
79.74
77.27
77.06
76.85
71.92
70.47
63.57
26.77
26.28
HO
OH
OBn
O
O
O
3
200 190 180 170 160 150 140 130 120 110 100 90 80 70 60 50 40 30 20 10 0
f1 (ppm)

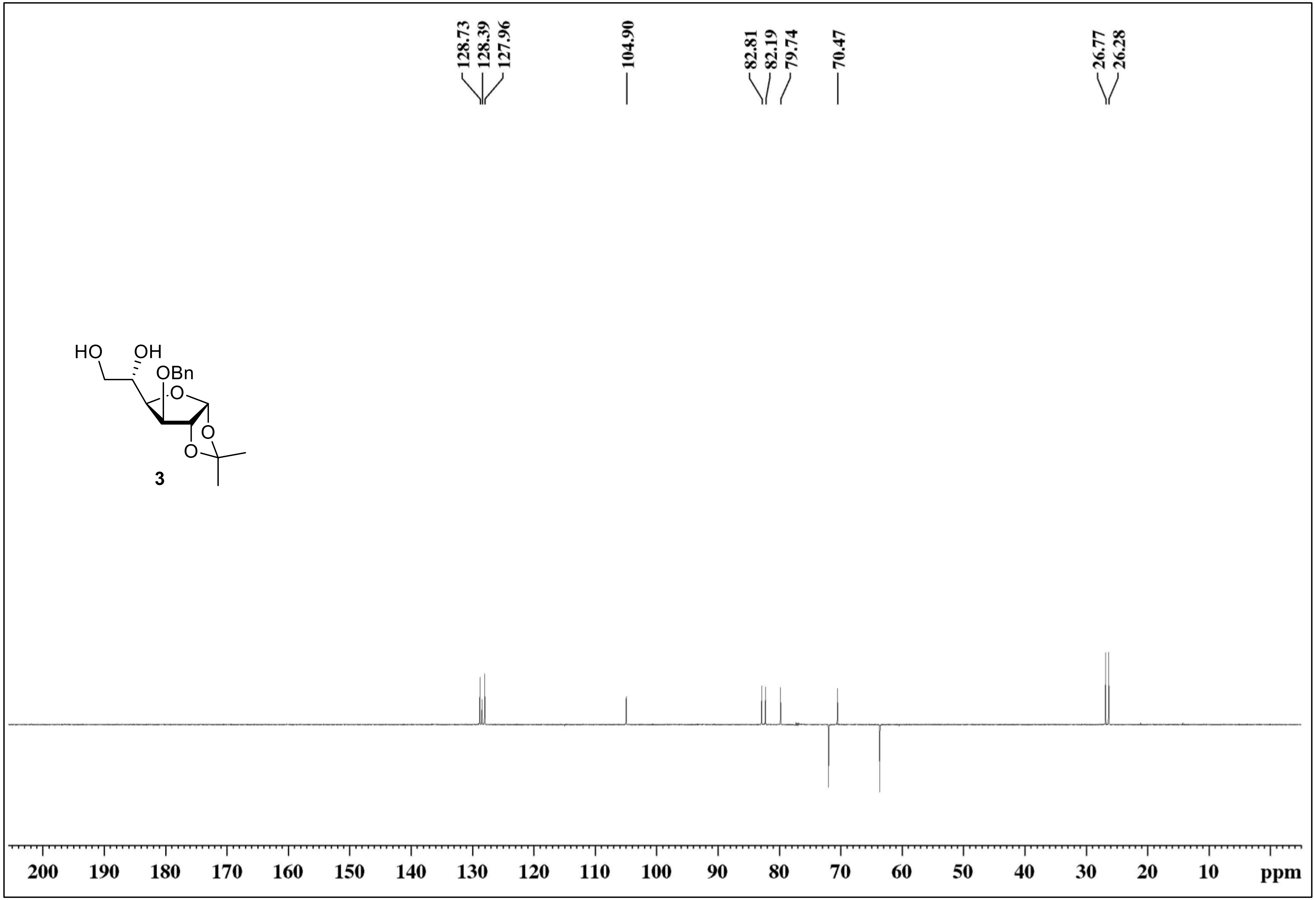
128.73
128.39
127.96
104.90
82.81
82.19
79.74
70.47
26.77
26.28
HO
OH
OBn
O
O
O
3
200 190 180 170 160 150 140 130 120 110 100 90 80 70 60 50 40 30 20 10 ppm

^1H-^1H COSY

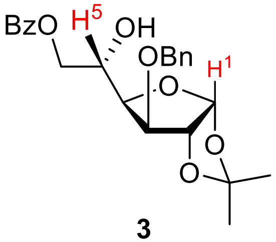
BzO
H5
OH
OBn
H1
O
O
O

3

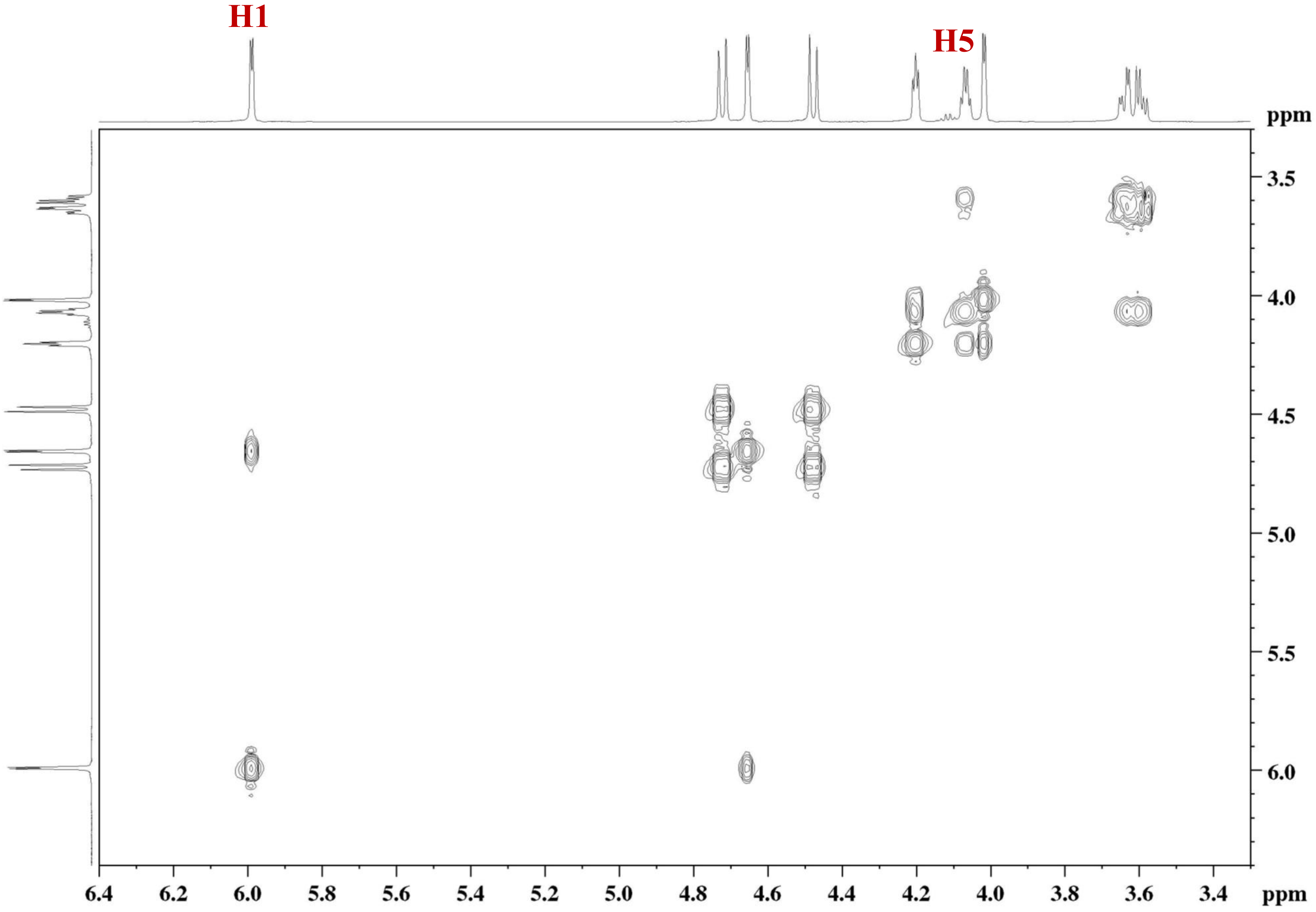
H1
H5
ppm
3.5
4.0
4.5
5.0
5.5
6.0
6.4
6.2
6.0
5.8
5.6
5.4
5.2
5.0
4.8
4.6
4.4
4.2
4.0
3.8
3.6
3.4
ppm

^{13}C-^{1}H HSQC

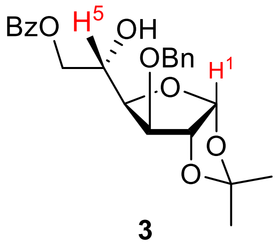
BzO
H5
OH
OBn
O
H1
O
O

3

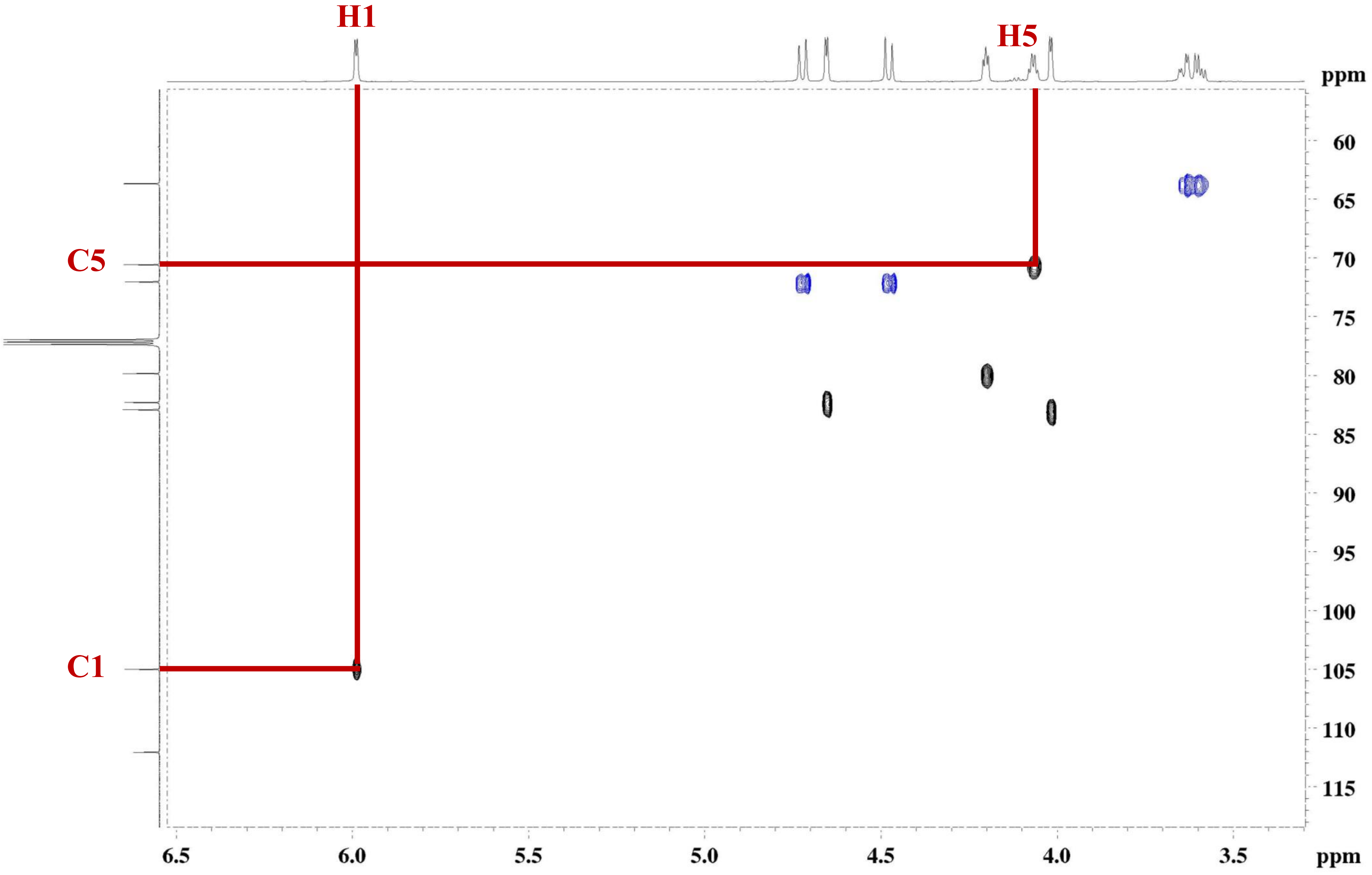
H1
H5
C5
C1
ppm
60
65
70
75
80
85
90
95
100
105
110
115
6.5
6.0
5.5
5.0
4.5
4.0
3.5
ppm

HRMS-ESI

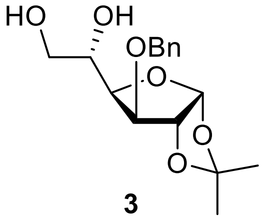
HO
OH
OBn
O
O
O

3

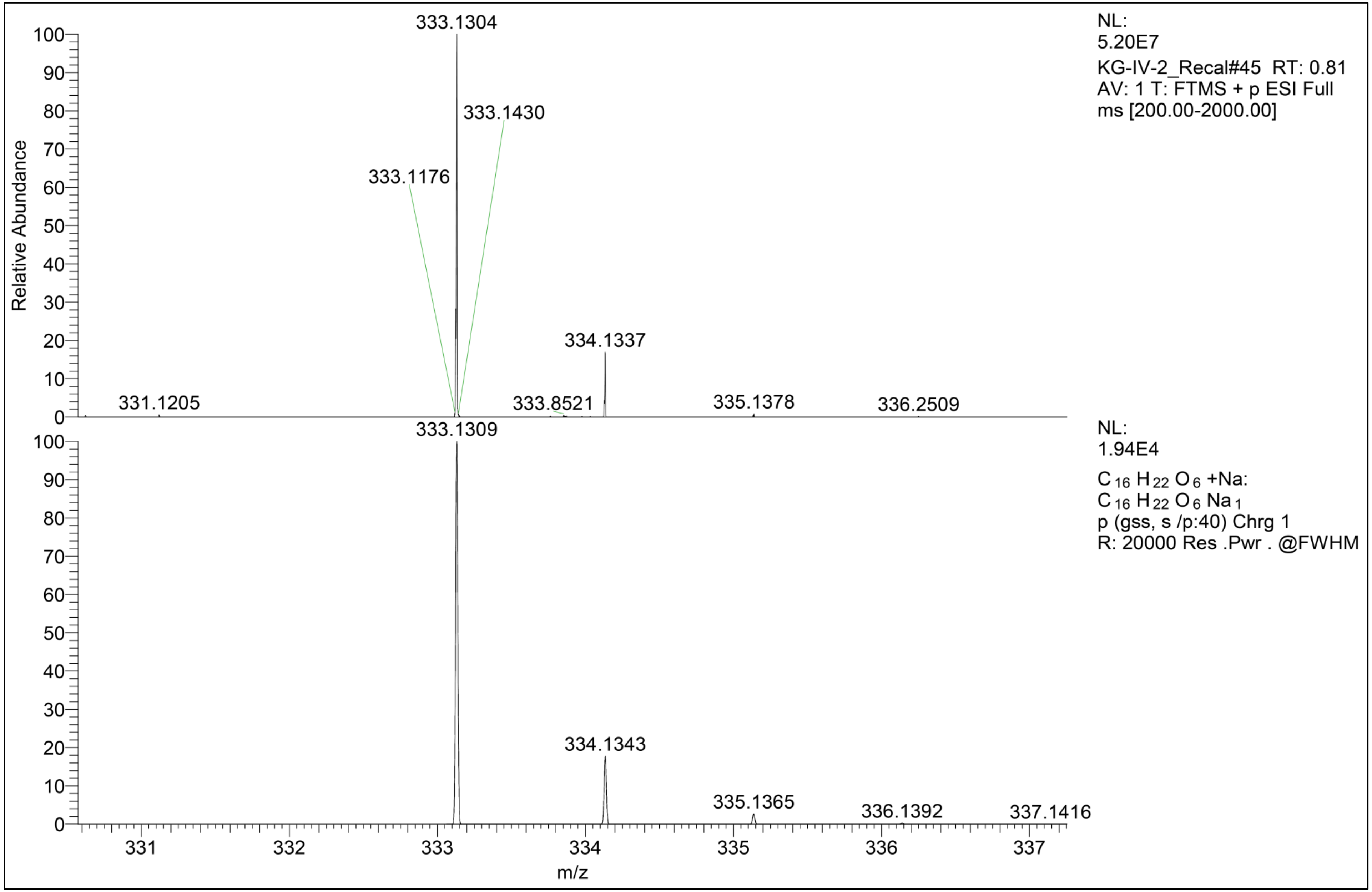
NL:
5.20E7
KG-IV-2_Recal#45 RT: 0.81
AV: 1 T: FTMS + p ESI Full
ms [200.00-2000.00]
333.1304
333.1430
333.1176
334.1337
331.1205
333.8521
335.1378
336.2509
Relative Abundance
NL:
1.94E4
C 16 H 22 O 6 +Na:
C 16 H 22 O 6 Na 1
p (gss, s /p:40) Chrg 1
R: 20000 Res .Pwr . @FWHM
333.1309
334.1343
335.1365
336.1392
337.1416
m/z

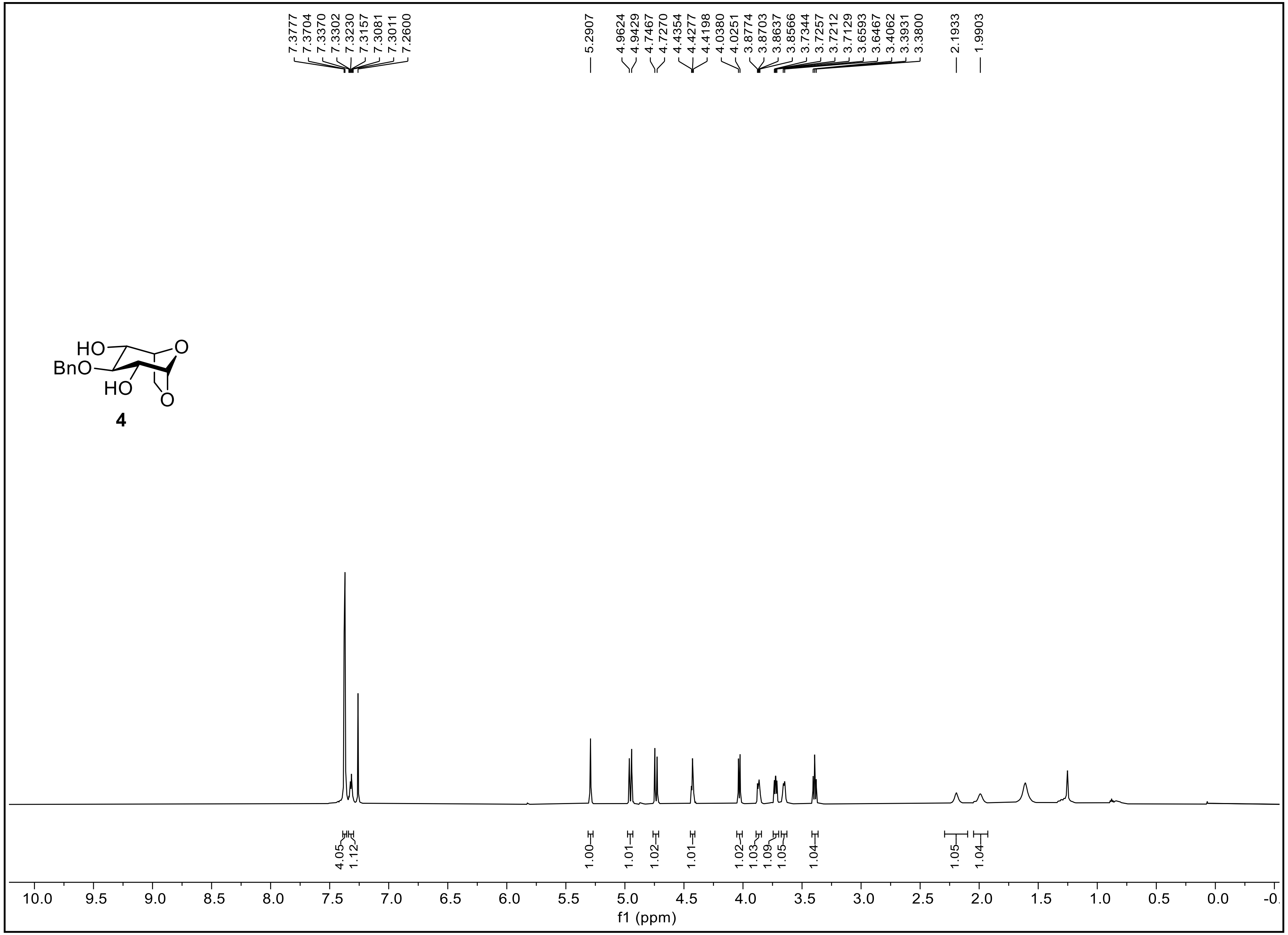
7.3777
7.3704
7.3370
7.3302
7.3230
7.3157
7.3081
7.3011
7.2600
5.2907
4.9624
4.9429
4.7467
4.7270
4.4354
4.4277
4.4198
4.0380
4.0251
3.8774
3.8703
3.8637
3.8566
3.7344
3.7257
3.7212
3.7129
3.6593
3.6467
3.4062
3.3931
3.3800
2.1933
1.9903
HO
BnO
HO
O
O
4
4.05
1.12
1.00
1.01
1.02
1.01
1.02
1.03
1.09
1.05
1.04
1.05
1.04
10.0
9.5
9.0
8.5
8.0
7.5
7.0
6.5
6.0
5.5
5.0
4.5
4.0
3.5
3.0
2.5
2.0
1.5
1.0
0.5
0.0
f1 (ppm)

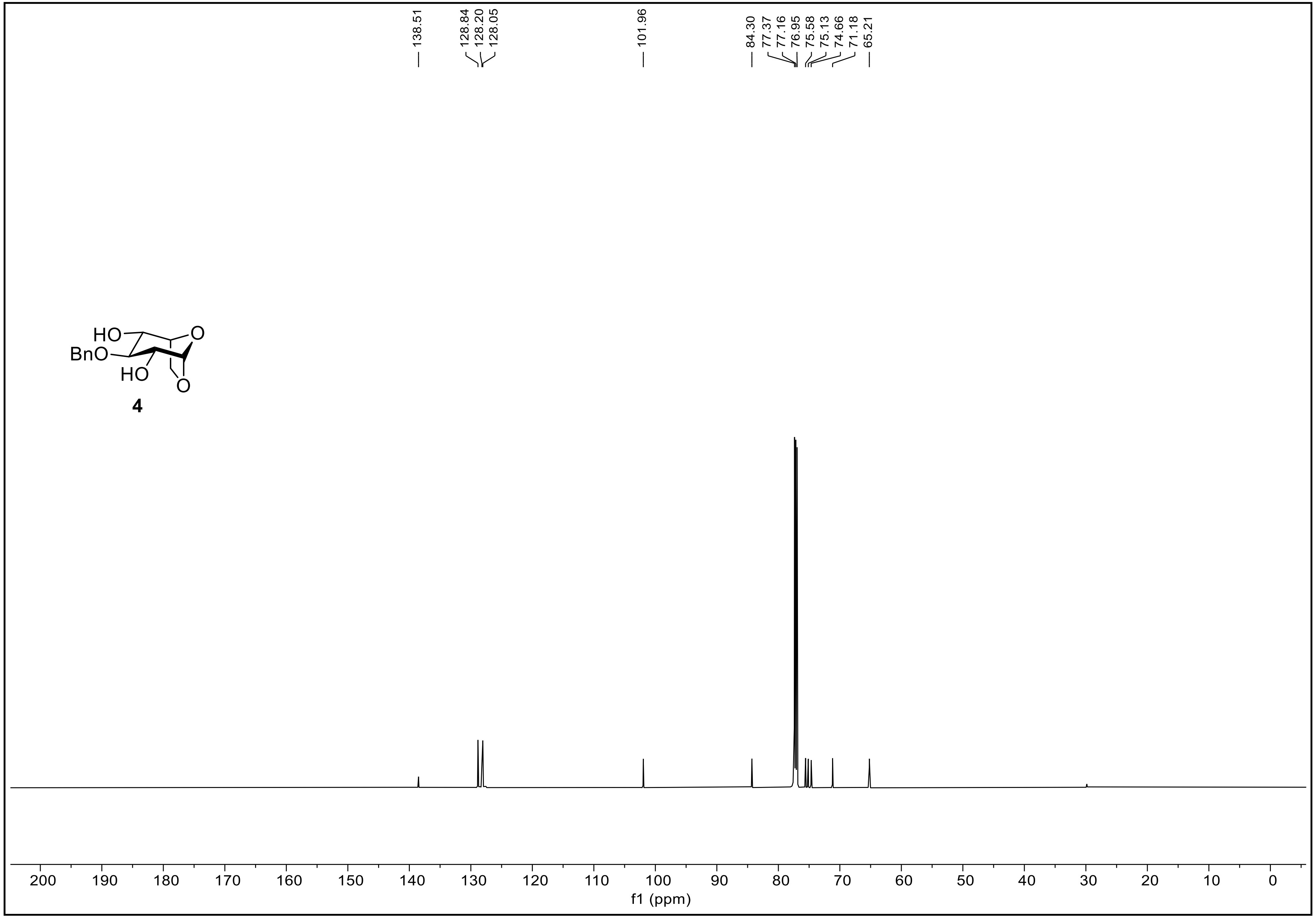
138.51
128.84
128.20
128.05
101.96
84.30
77.37
77.16
76.95
75.58
75.13
74.66
71.18
65.21
HO
BnO
HO
O
O
4
200 190 180 170 160 150 140 130 120 110 100 90 80 70 60 50 40 30 20 10 0
f1 (ppm)

^{1}H-^{1}H COSY

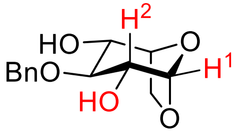

HO
BnO
HO
O
O
H2
H1

4

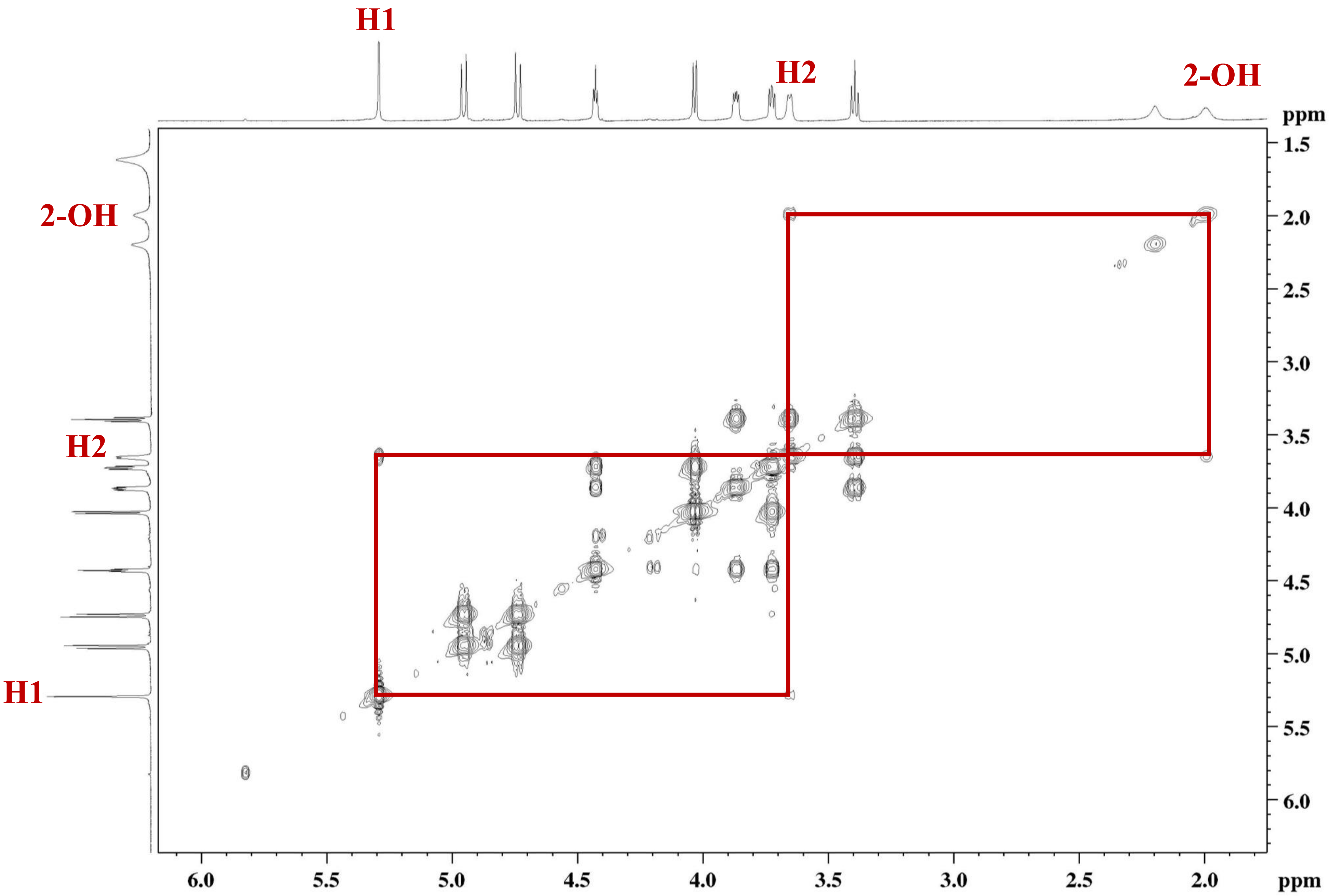

H1
H2
2-OH
ppm
2-OH
H2
H1
1.5
2.0
2.5
3.0
3.5
4.0
4.5
5.0
5.5
6.0
ppm
6.0
5.5
5.0
4.5
4.0
3.5
3.0
2.5
2.0
ppm

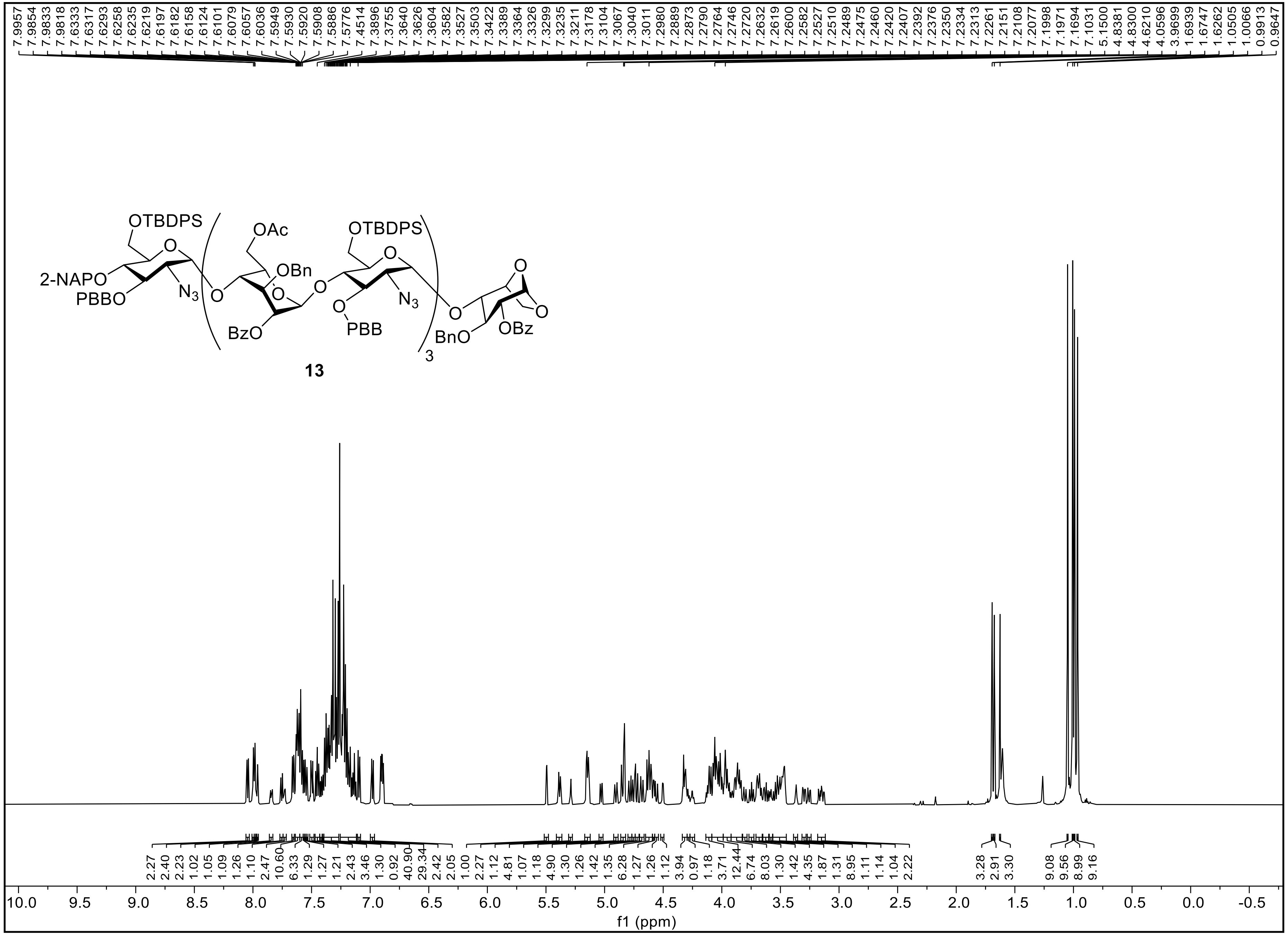

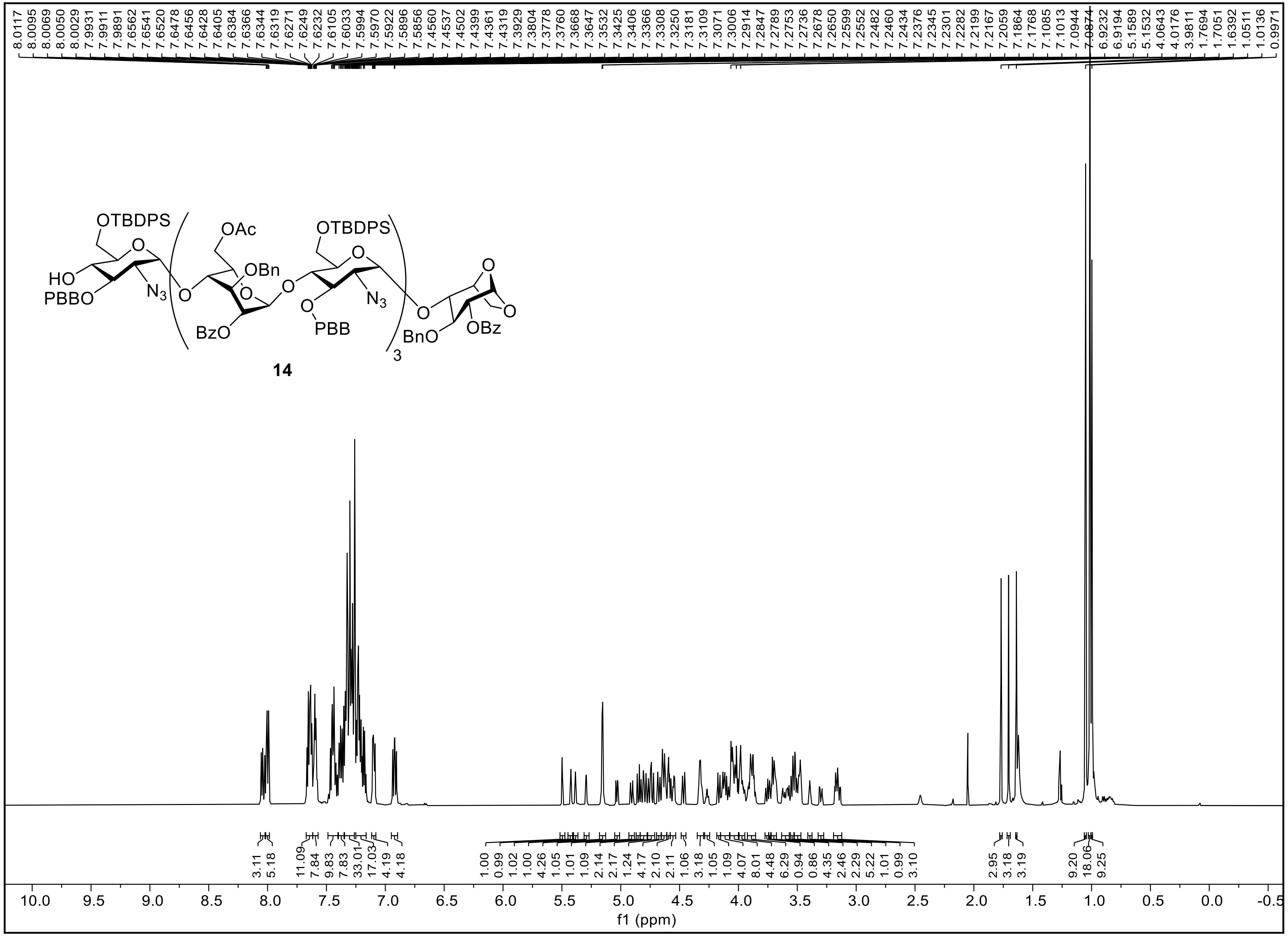
OTBDPS
OAc
OTBDPS
HO
PBBO
N3
O
OBn
BzO
PBB
N3
BnO
OBz
3
14
f1 (ppm)

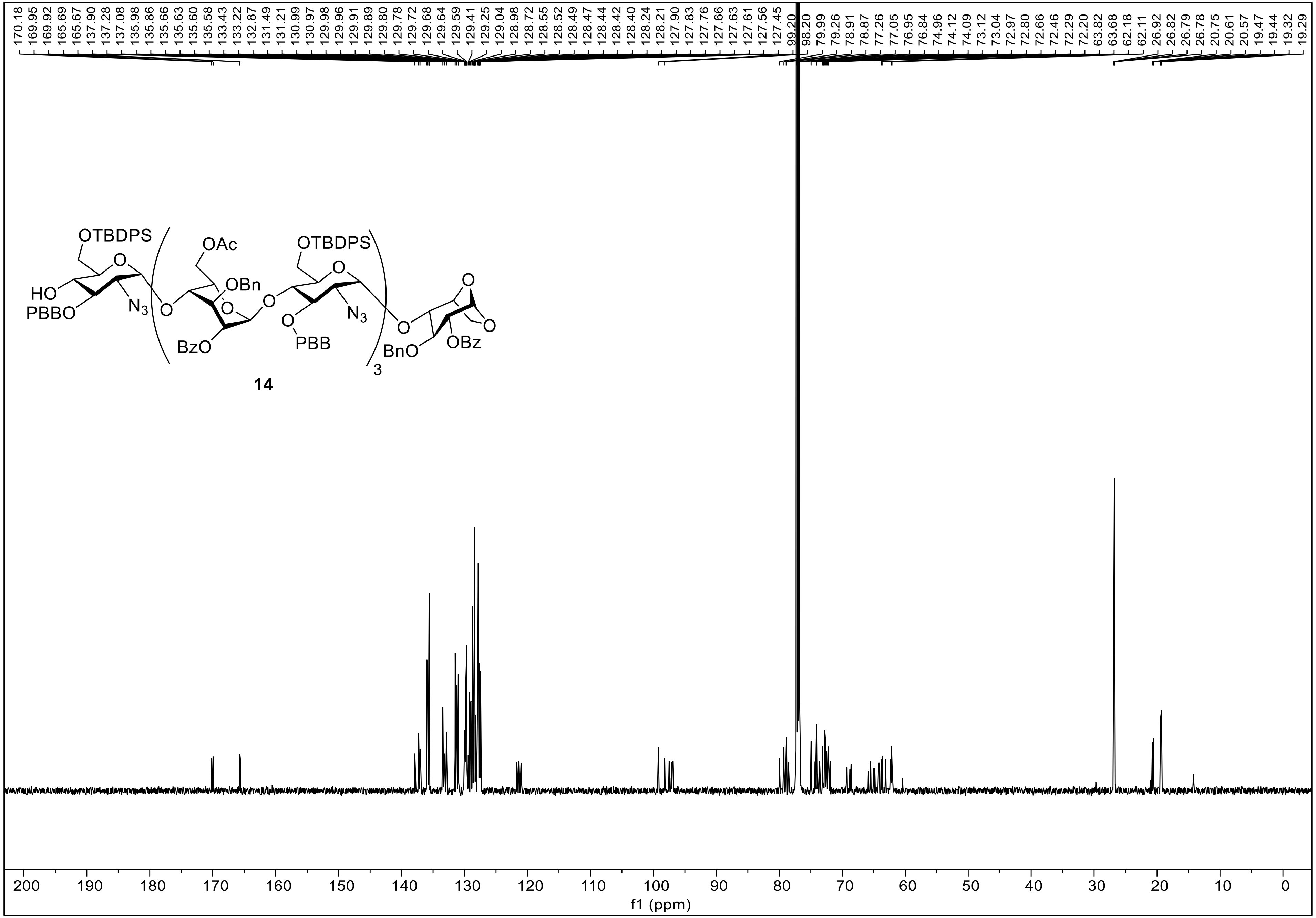

170.18
169.95
169.92
165.69
165.67
137.90
137.28
137.08
135.98
135.86
135.66
135.63
135.60
135.58
133.43
133.22
132.87
131.49
131.21
130.99
130.97
129.98
129.96
129.91
129.89
129.80
129.78
129.72
129.68
129.64
129.59
129.41
129.25
129.04
128.98
128.72
128.55
128.52
128.49
128.47
128.44
128.42
128.40
128.24
128.21
127.90
127.83
127.76
127.66
127.63
127.61
127.56
127.45
99.20
98.20
79.99
79.26
78.91
78.87
77.26
77.05
76.95
76.84
74.96
74.12
74.09
73.12
73.04
72.97
72.80
72.66
72.46
72.29
72.20
63.82
63.68
62.18
62.11
26.92
26.82
26.79
26.78
20.75
20.61
20.57
19.47
19.44
19.32
19.29
OTBDPS
OAc
OTBDPS
HO
PBBO
N_3
O
OBn
BzO
O
PBB
N_3
3
BnO
OBz
14
f1 (ppm)

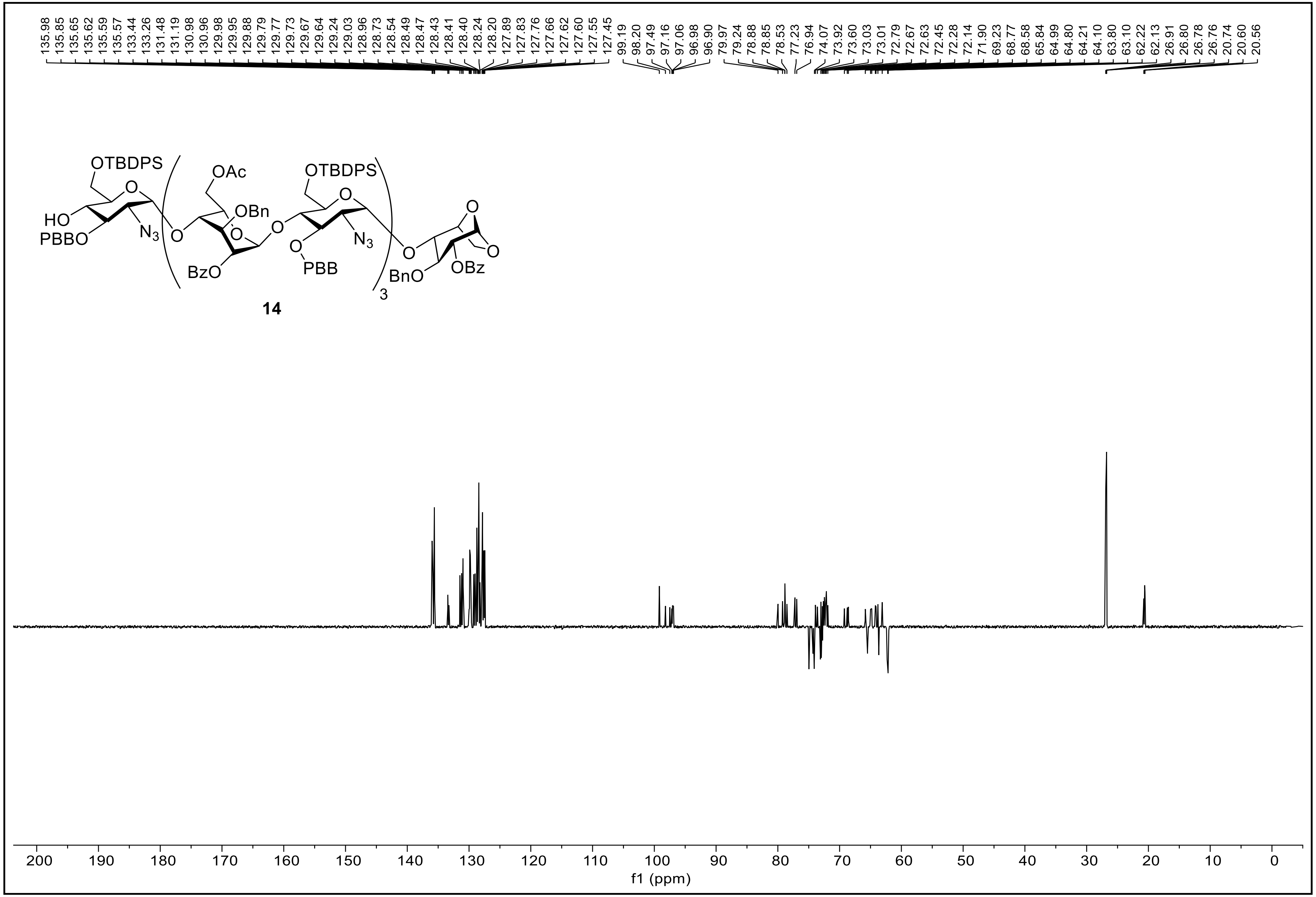

135.98
135.85
135.65
135.62
135.59
135.57
133.44
133.26
131.48
131.19
130.98
130.96
129.98
129.95
129.88
129.79
129.77
129.73
129.67
129.64
129.24
129.03
128.96
128.73
128.54
128.49
128.47
128.43
128.41
128.40
128.24
128.20
127.89
127.83
127.76
127.66
127.62
127.60
127.55
127.45
99.19
98.20
97.49
97.16
97.06
96.98
96.90
79.97
79.24
78.88
78.85
78.53
77.23
76.94
74.07
73.92
73.60
73.03
73.01
72.79
72.67
72.63
72.45
72.28
72.14
71.90
69.23
68.77
68.58
65.84
64.99
64.80
64.21
64.10
63.80
63.10
62.22
62.13
26.91
26.80
26.78
26.76
20.74
20.60
20.56
OTBDPS
HO
PBBO
N3
O
OAc
OBn
BzO
OTBDPS
PBB
N3
3
BnO
OBz
14
200
190
180
170
160
150
140
130
120
110
100
90
80
70
60
50
40
30
20
10
0
f1 (ppm)

HRMS-MALDI

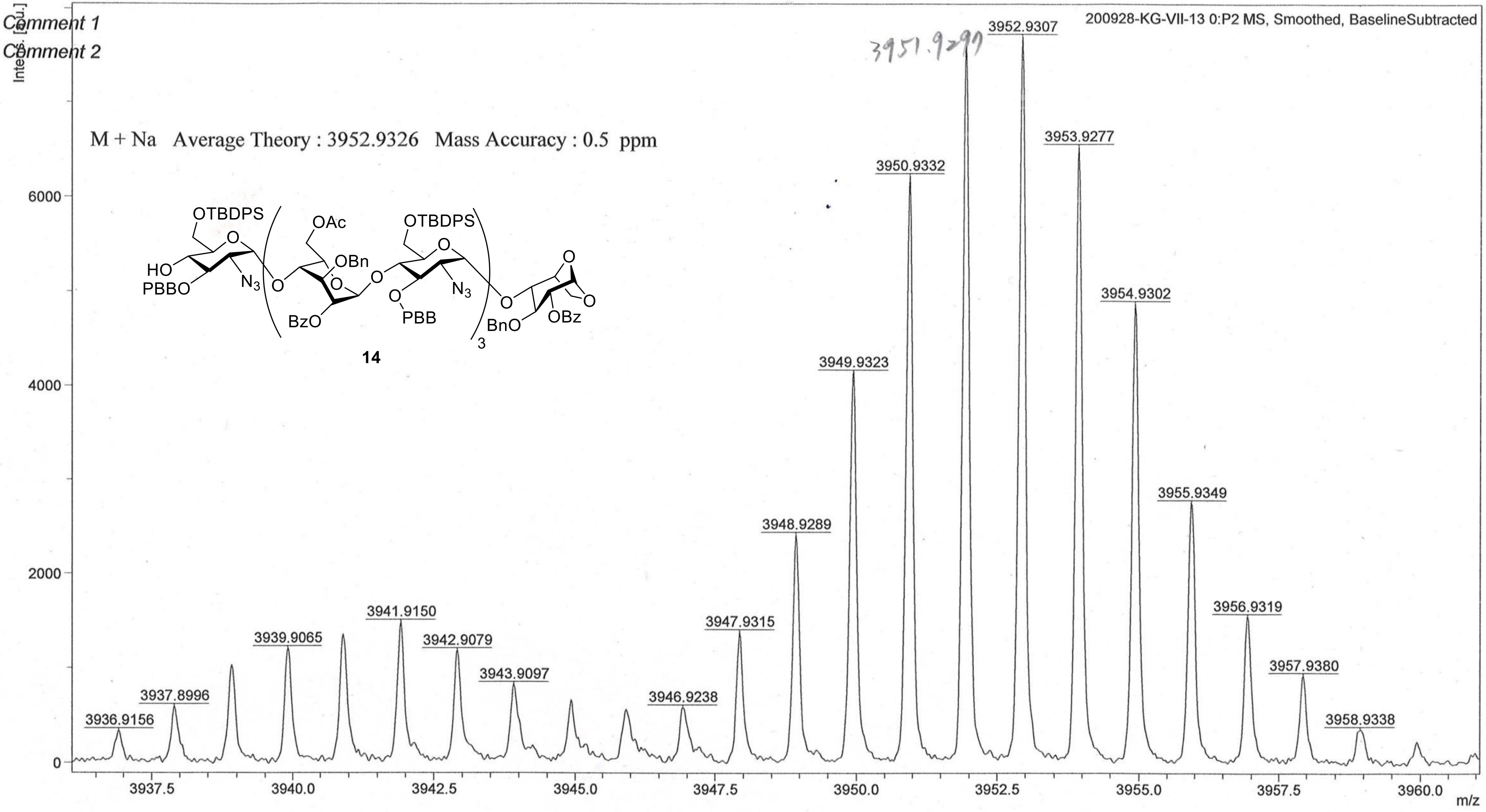
Comment 1
Comment 2
Intens. [a.u.]
200928-KG-VII-13 0:P2 MS, Smoothed, BaselineSubtracted
M + Na Average Theory : 3952.9326 Mass Accuracy : 0.5 ppm
OTBDPS
OAc
OBn
OTBDPS
HO
PBBO
N_3
BzO
PBB
N_3
BnO
OBz
3
14
3952.9307
3951.9297
3953.9277
3950.9332
3954.9302
3949.9323
3955.9349
3948.9289
3956.9319
3947.9315
3941.9150
3939.9065
3942.9079
3943.9097
3946.9238
3957.9380
3937.8996
3936.9156
3958.9338
6000
4000
2000
0
3937.5
3940.0
3942.5
3945.0
3947.5
3950.0
3952.5
3955.0
3957.5
3960.0
m/z

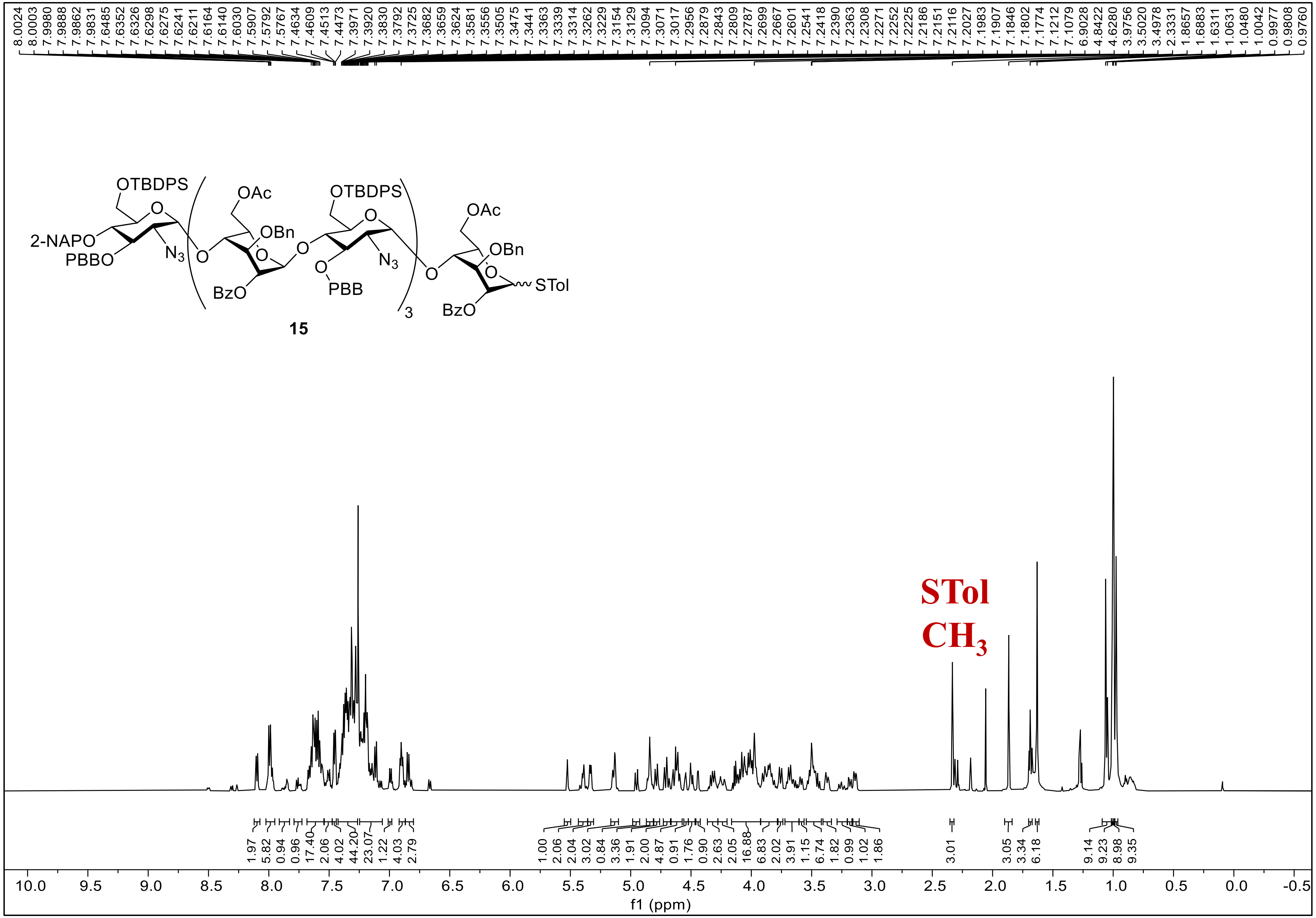

OTBDPS
OAc
OTBDPS
OAc
2-NAPO
PBBO
N3
OBn
O
PBB
BzO
N3
OBn
STol
BzO
3
15
STol
CH3
f1 (ppm)

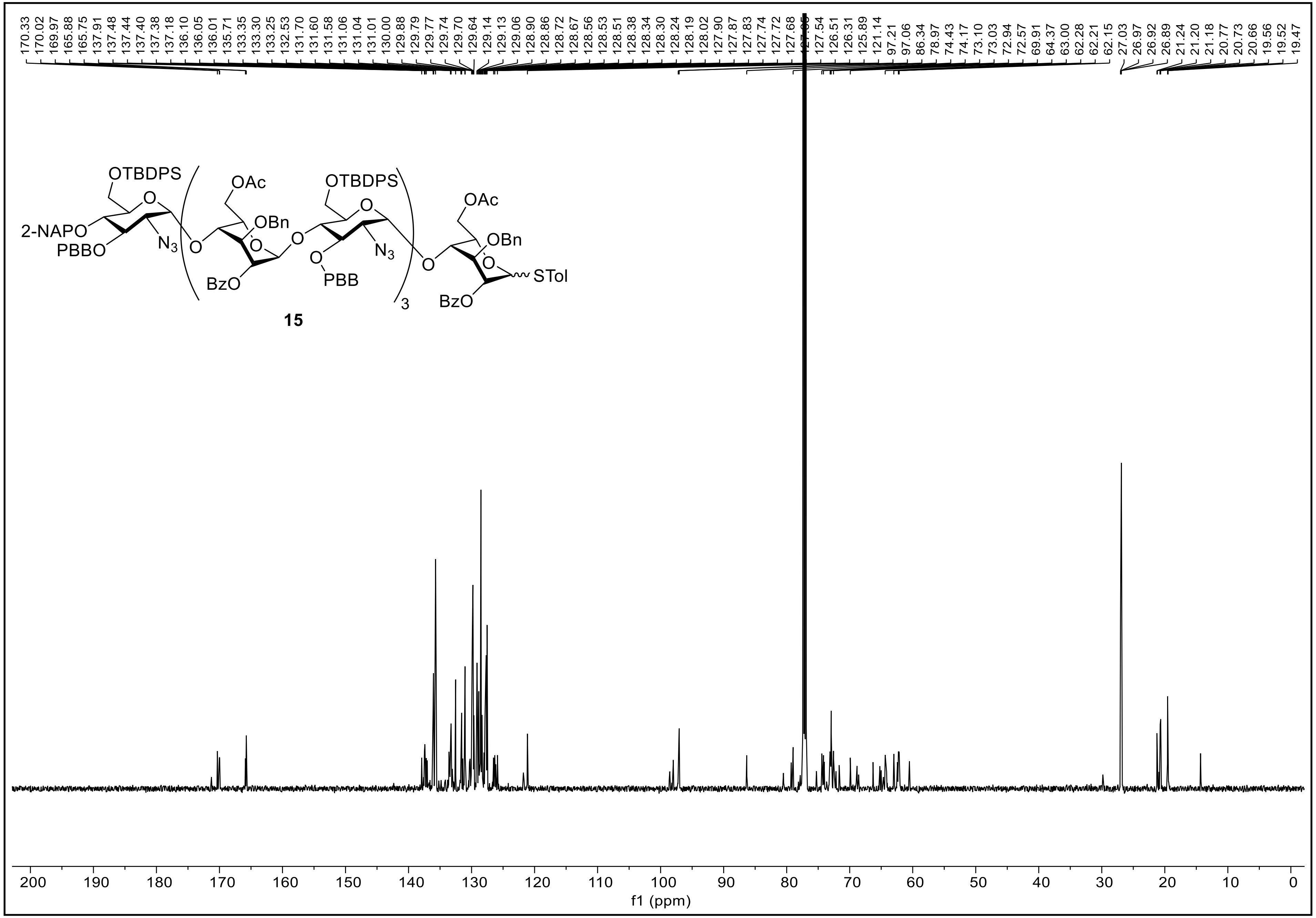

OTBDPS
OAc
OBn
2-NAPO
PBBO
N3
O
BzO
PBB
3
STol
15
f1 (ppm)

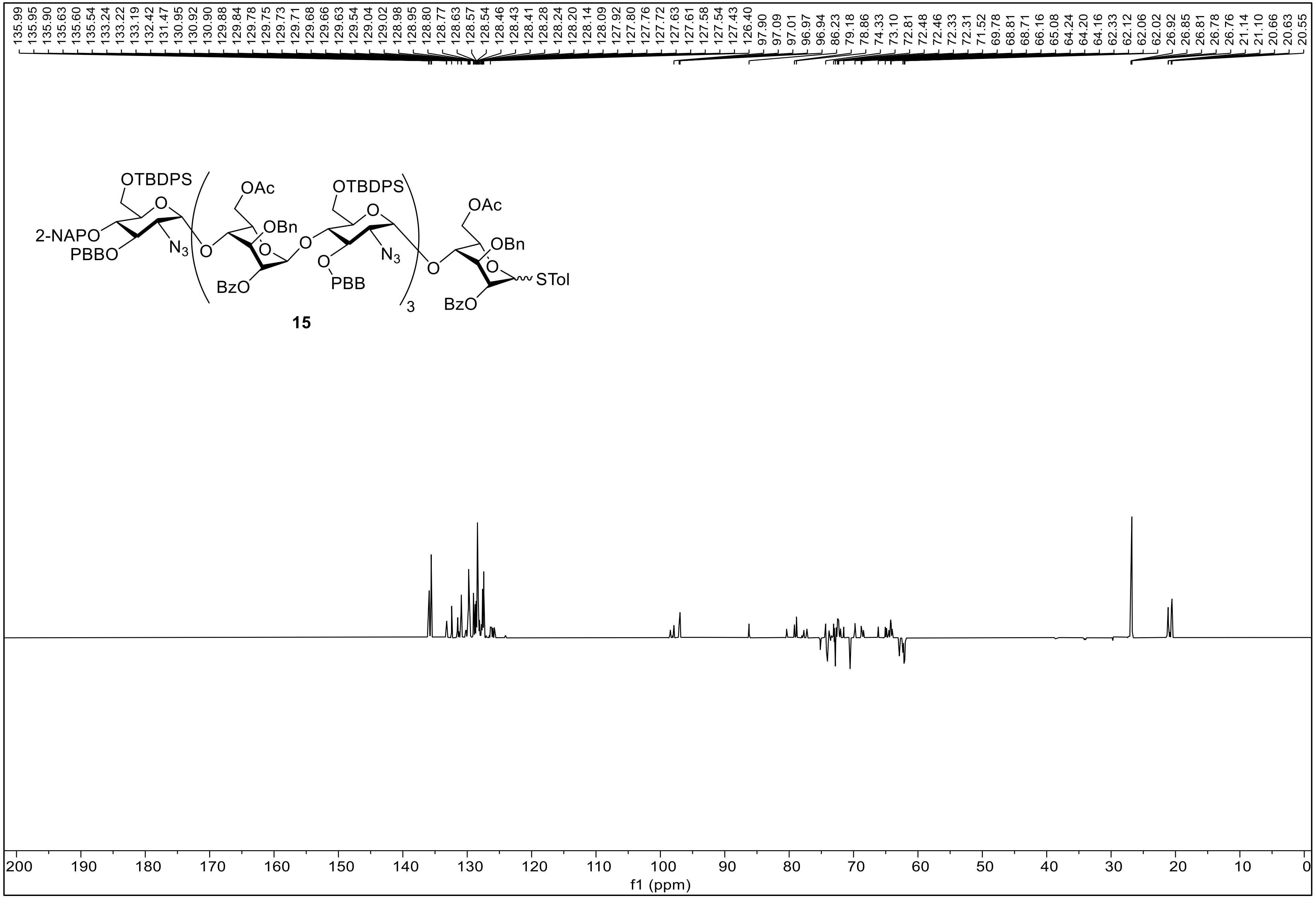
135.99
135.95
135.90
135.63
135.60
135.54
133.24
133.22
133.19
132.42
131.47
130.95
130.92
130.90
129.88
129.84
129.78
129.75
129.73
129.71
129.68
129.66
129.63
129.54
129.04
129.02
128.98
128.95
128.80
128.77
128.63
128.57
128.54
128.46
128.43
128.41
128.28
128.24
128.20
128.14
128.09
127.92
127.80
127.76
127.72
127.63
127.61
127.58
127.54
127.43
126.40
97.90
97.09
97.01
96.97
96.94
86.23
79.18
78.86
74.33
73.10
72.81
72.48
72.46
72.33
72.31
71.52
69.78
68.81
68.71
66.16
65.08
64.24
64.20
64.16
62.33
62.12
62.06
62.02
26.92
26.85
26.81
26.78
26.76
21.14
21.10
20.66
20.63
20.55
OTBDPS
OAc
OBn
2-NAPO
PBBO
N3
O
BzO
PBB
STol
3
15
f1 (ppm)

HRMS-MALDI

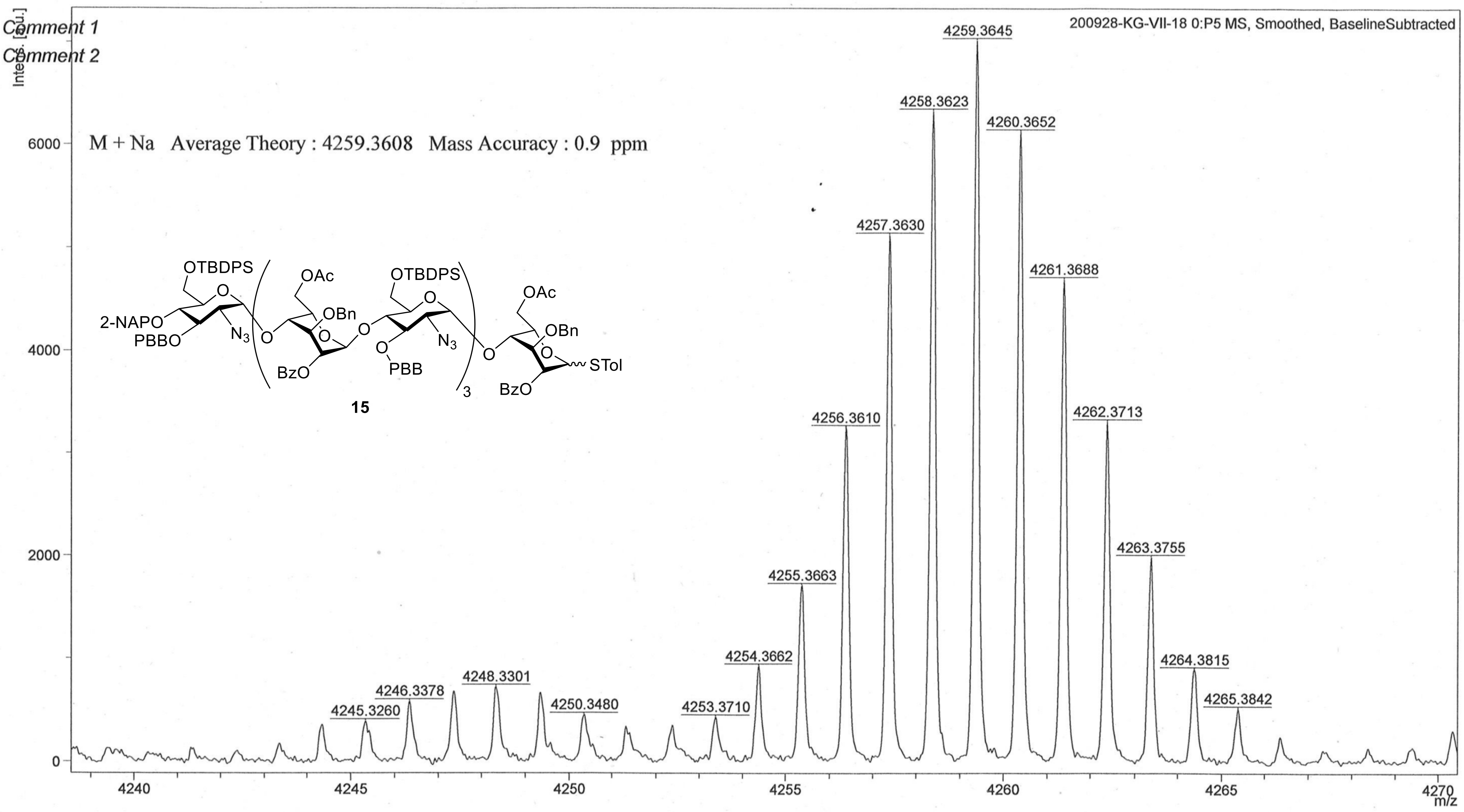

Comment 1
Comment 2
Intens. [a.u.]
200928-KG-VII-18 0:P5 MS, Smoothed, BaselineSubtracted
M + Na Average Theory : 4259.3608 Mass Accuracy : 0.9 ppm
OTBDPS
OAc
OTBDPS
OAc
2-NAPO
PBBO
N3
OBn
O
BzO
O
PBB
N3
3
OBn
STol
BzO
15
4259.3645
4258.3623
4260.3652
4257.3630
4261.3688
4256.3610
4262.3713
4263.3755
4255.3663
4254.3662
4264.3815
4265.3842
4253.3710
4250.3480
4248.3301
4246.3378
4245.3260
6000
4000
2000
0
4240
4245
4250
4255
4260
4265
4270
m/z

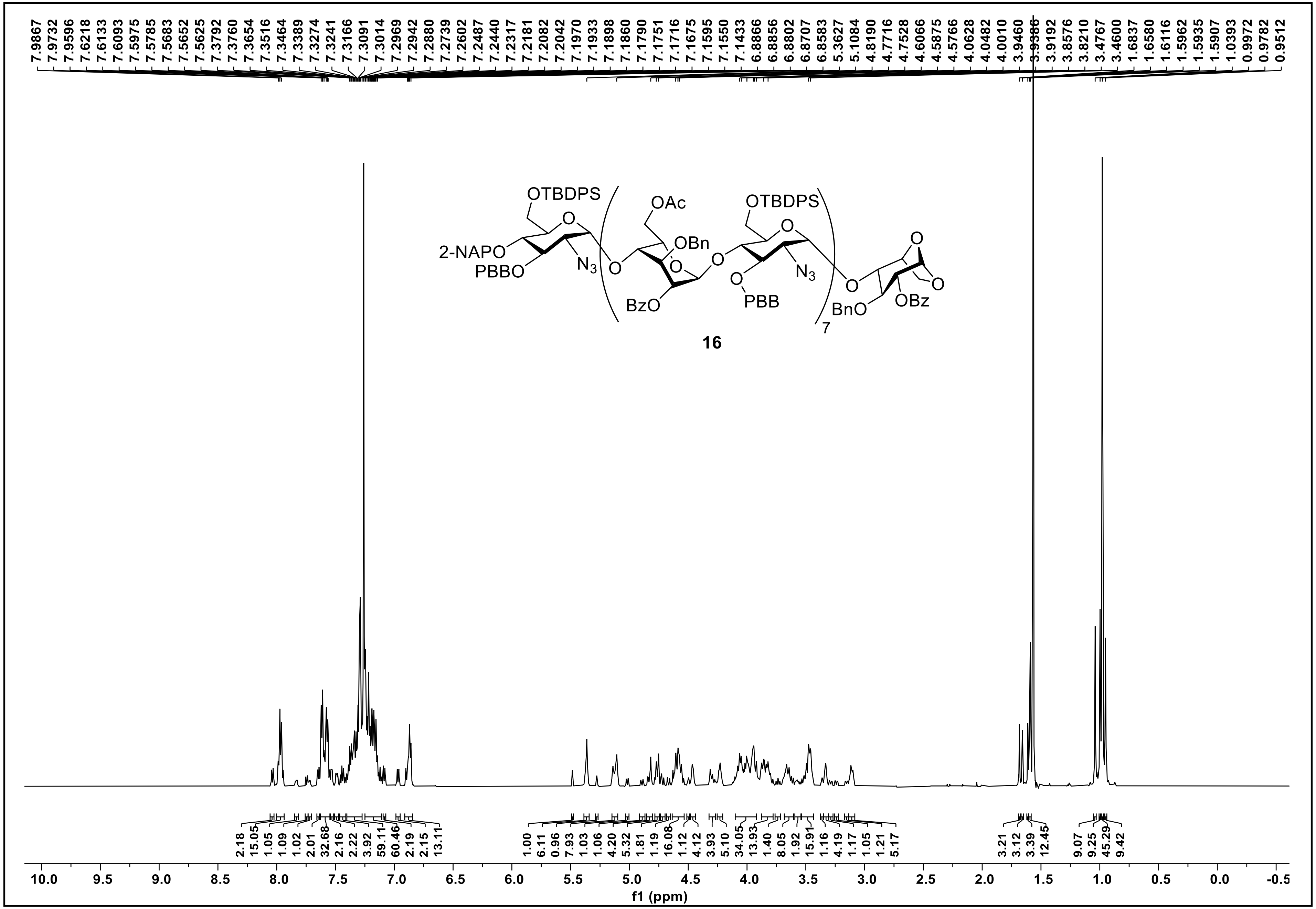

OTBDPS
OAc
OTBDPS
2-NAPO
PBBO
N3
OBn
O
BzO
PBB
N3
BnO
OBz
7
16
f1 (ppm)

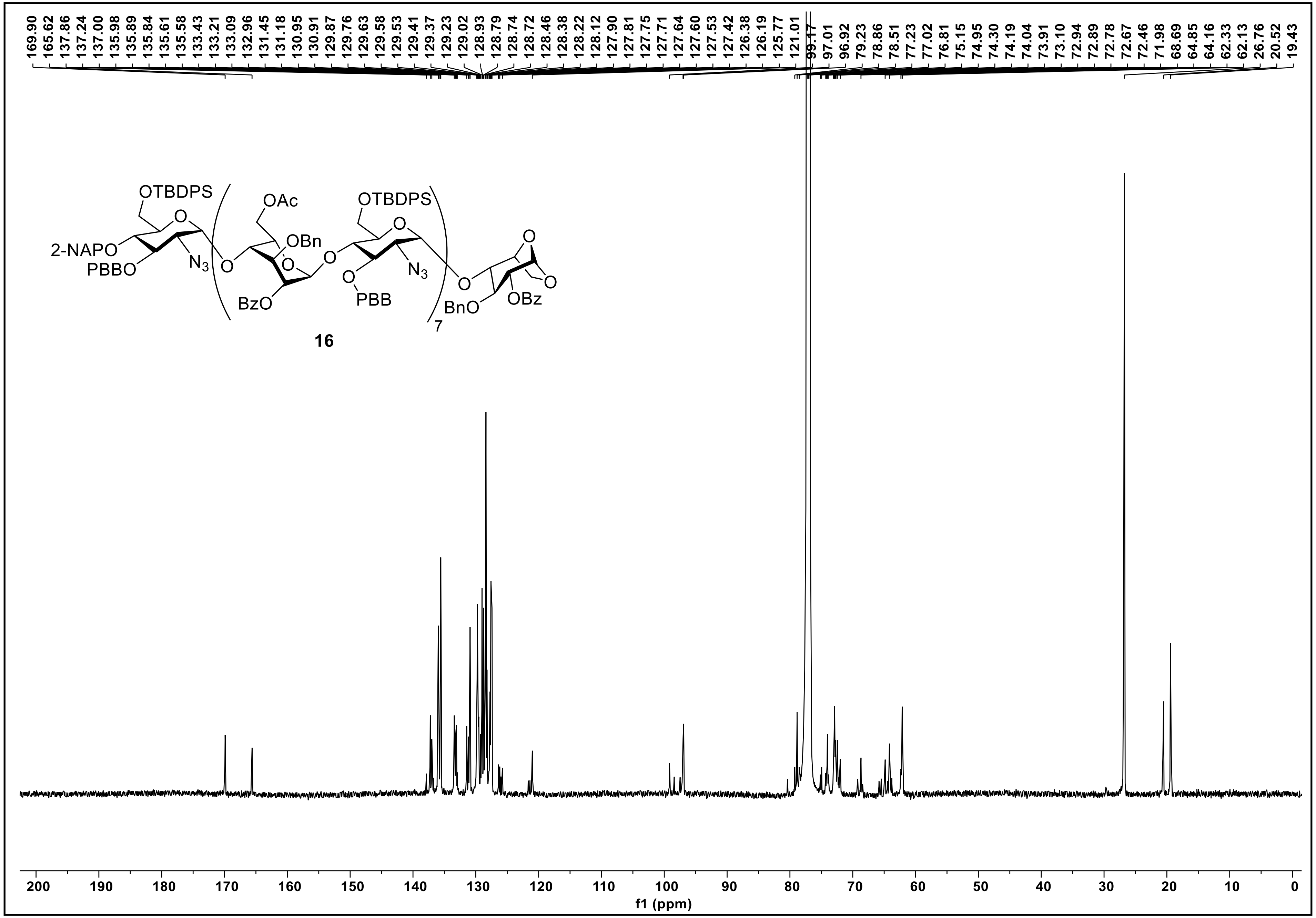
16
f1 (ppm)

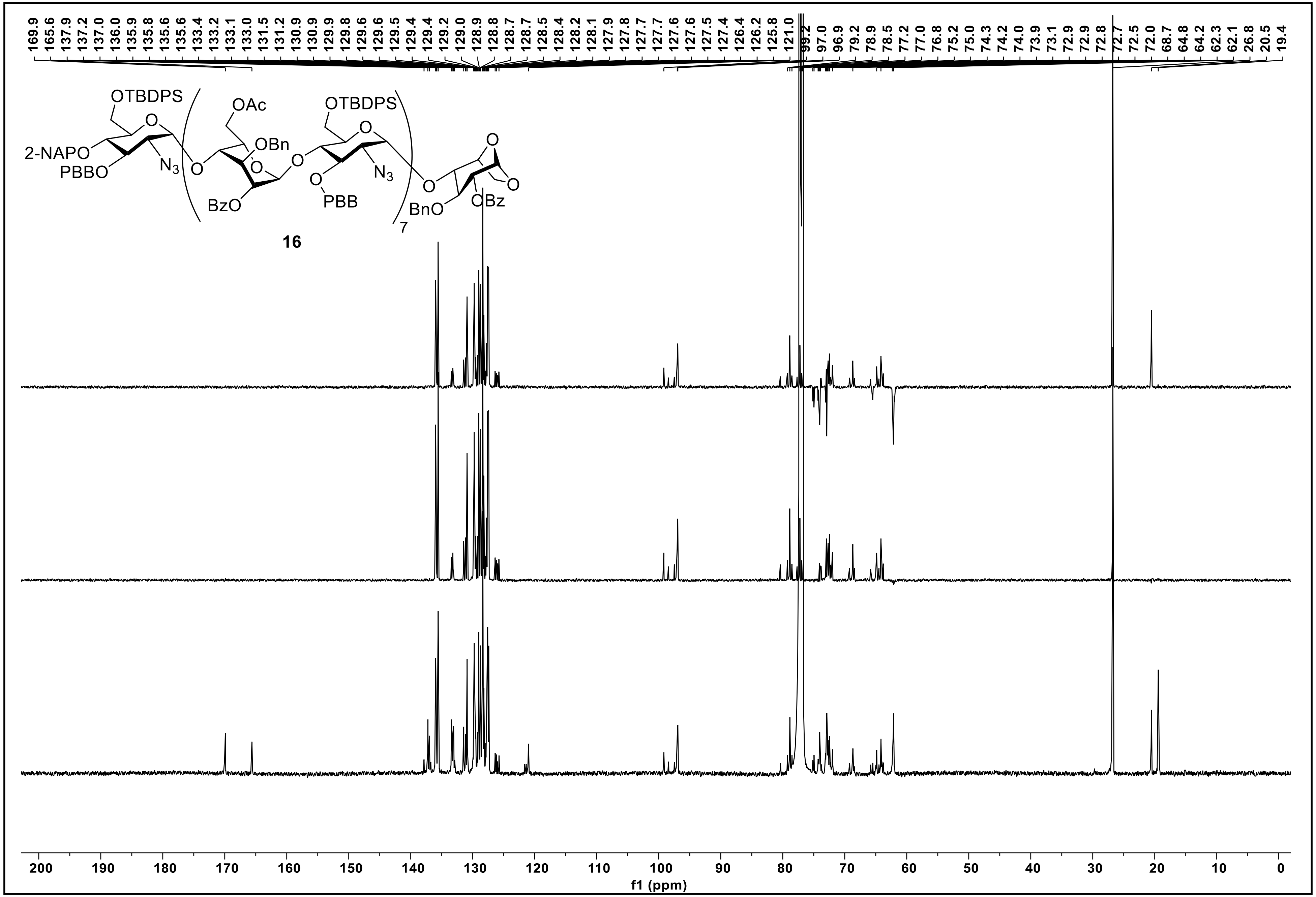
OTBDPS
OAc
OTBDPS
2-NAPO
PBBO
N_3
OBn
O
BzO
PBB
N_3
7
BnO
OBz
16
f1 (ppm)

HRMS-MALDI

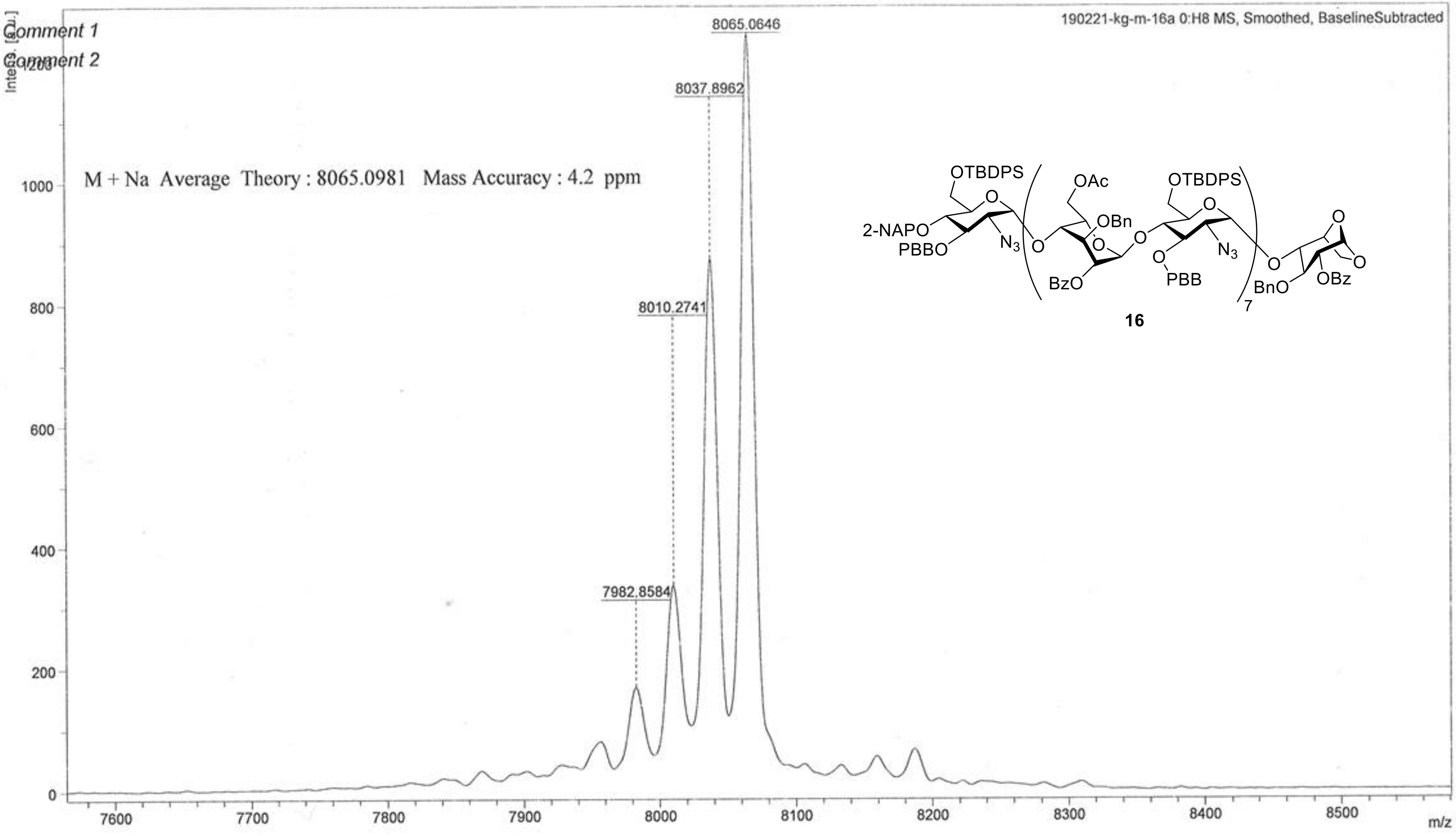

190221-kg-m-16a 0:H8 MS, Smoothed, BaselineSubtracted
Comment 1
Comment 2
Intens. [a.u.]
8065.0646
8037.8962
8010.2741
7982.8584
M + Na Average Theory : 8065.0981 Mass Accuracy : 4.2 ppm
OTBDPS
OAc
OTBDPS
2-NAPO
PBBO
N_3
OBn
BzO
PBB
BnO
OBz
7
16
m/z

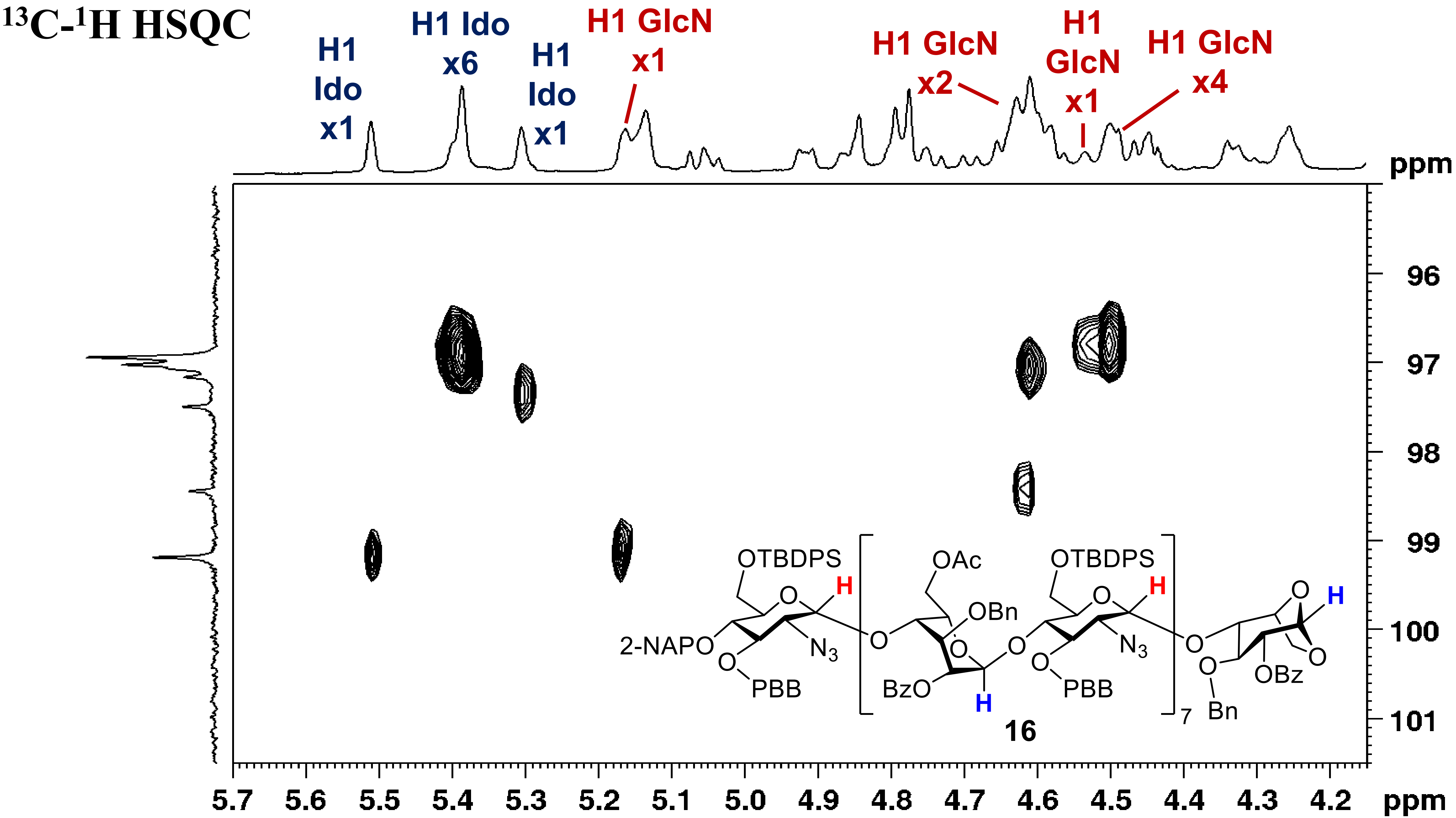

13C-1H HSQC
H1 Ido x1
H1 Ido x6
H1 Ido x1
H1 GlcN x1
H1 GlcN x2
H1 GlcN x1
H1 GlcN x4
ppm
96
97
98
99
100
101
5.7 5.6 5.5 5.4 5.3 5.2 5.1 5.0 4.9 4.8 4.7 4.6 4.5 4.4 4.3 4.2
OTBDPS
OAc
OBn
2-NAPO
N3
PBB
BzO
OBz
Bn
7
16

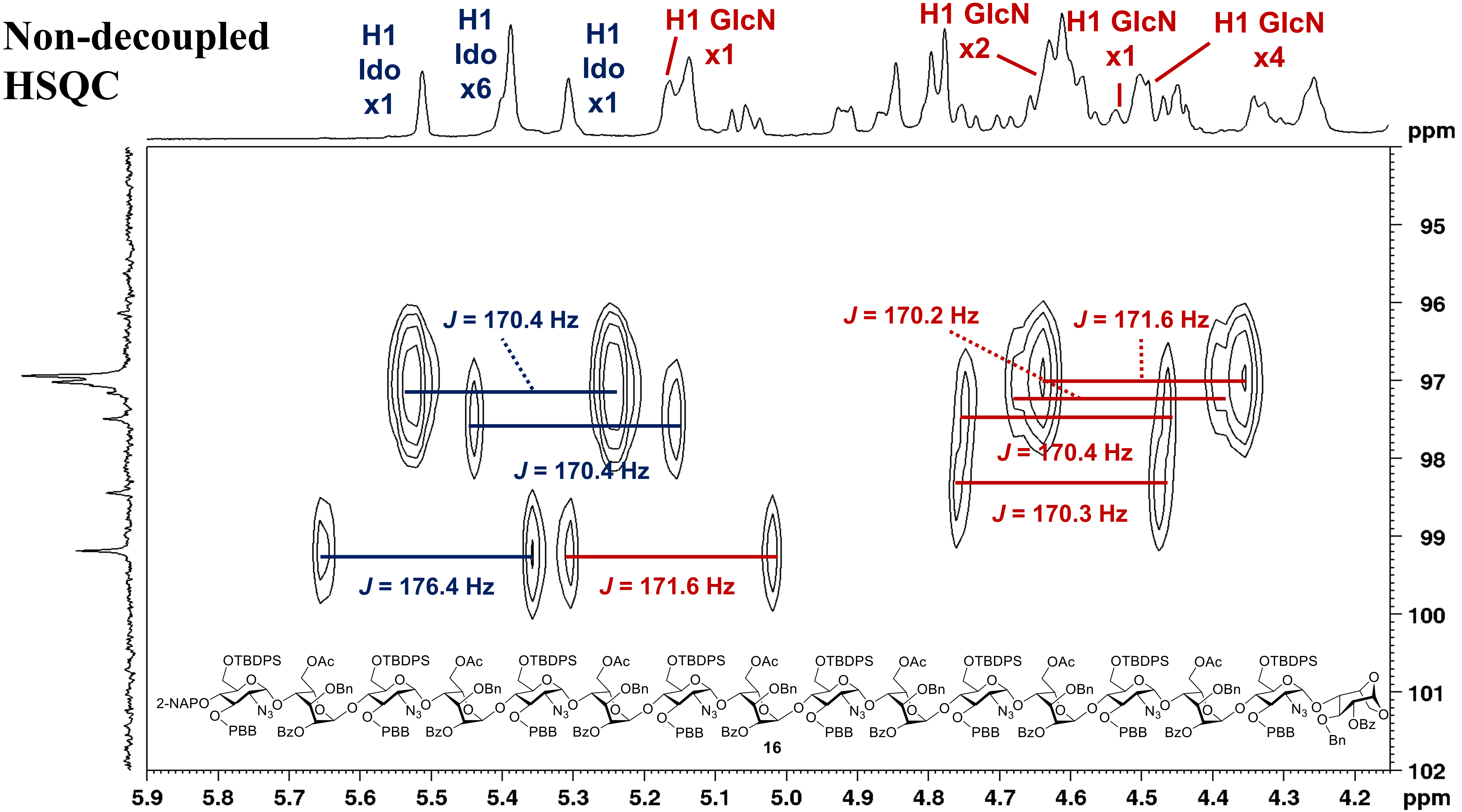
Non-decoupled HSQC
H1 Ido x1
H1 Ido x6
H1 Ido x1
H1 GlcN x1
H1 GlcN x2
H1 GlcN x1
H1 GlcN x4
ppm
J = 170.4 Hz
J = 170.4 Hz
J = 176.4 Hz
J = 171.6 Hz
J = 170.2 Hz
J = 171.6 Hz
J = 170.4 Hz
J = 170.3 Hz
2-NAPO
OTBDPS
OAc
OBn
N3
PBB
BzO
OBz
Bn
16
5.9 5.8 5.7 5.6 5.5 5.4 5.3 5.2 5.1 5.0 4.9 4.8 4.7 4.6 4.5 4.4 4.3 4.2 ppm
95 96 97 98 99 100 101 102 ppm

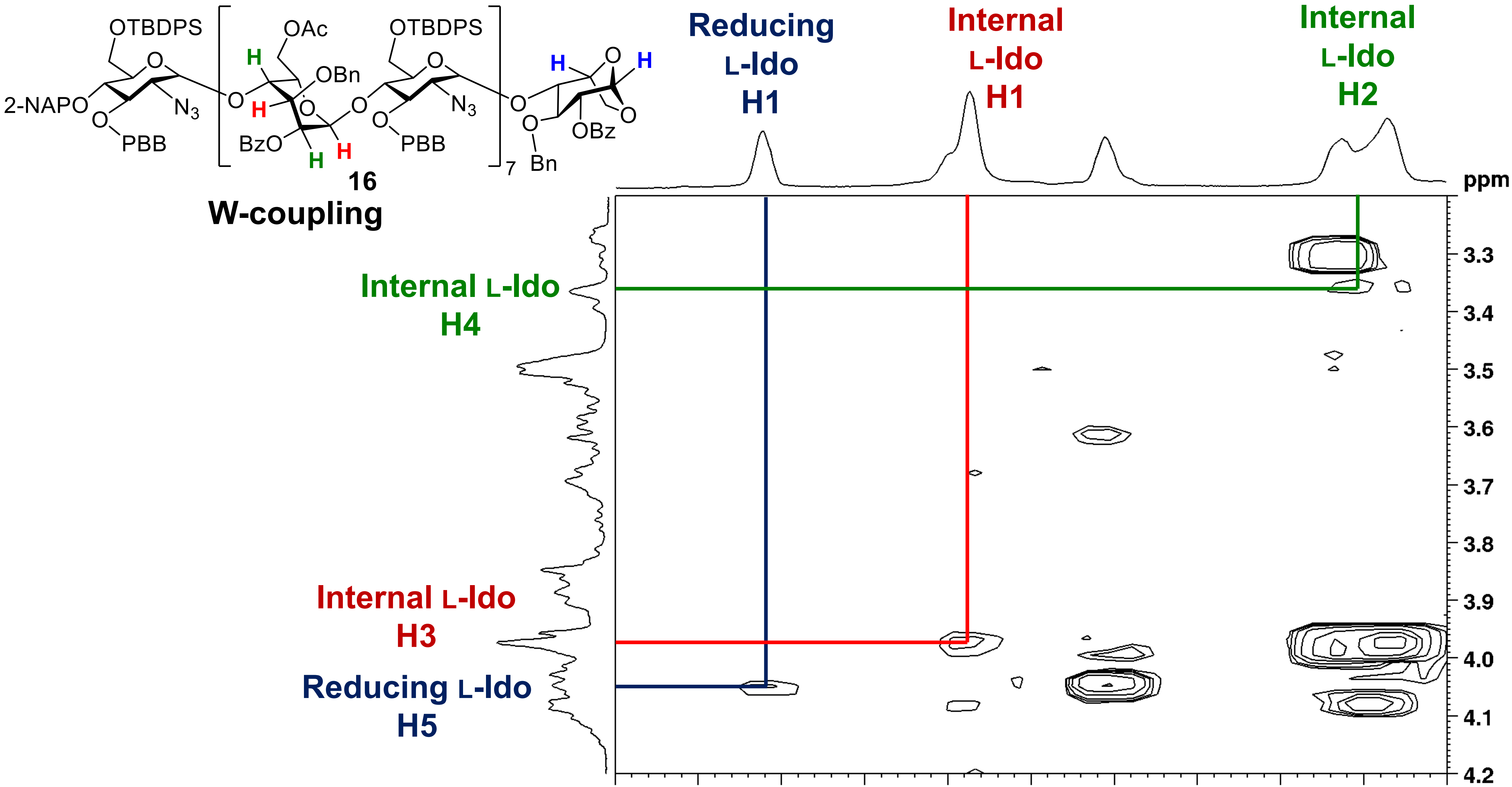
OTBDPS
OAc
OTBDPS
OBn
2-NAPO
N3
H
O
BzO
PBB
N3
PBB
OBz
Bn
7
16
W-coupling
Reducing L-Ido H1
Internal L-Ido H1
Internal L-Ido H2
Internal L-Ido H4
Internal L-Ido H3
Reducing L-Ido H5
ppm
5.60
5.55
5.50
5.45
5.40
5.35
5.30
5.25
5.20
5.15
3.3
3.4
3.5
3.6
3.7
3.8
3.9
4.0
4.1
4.2

^{1}H-^{1}H COSY

1D TOCSY

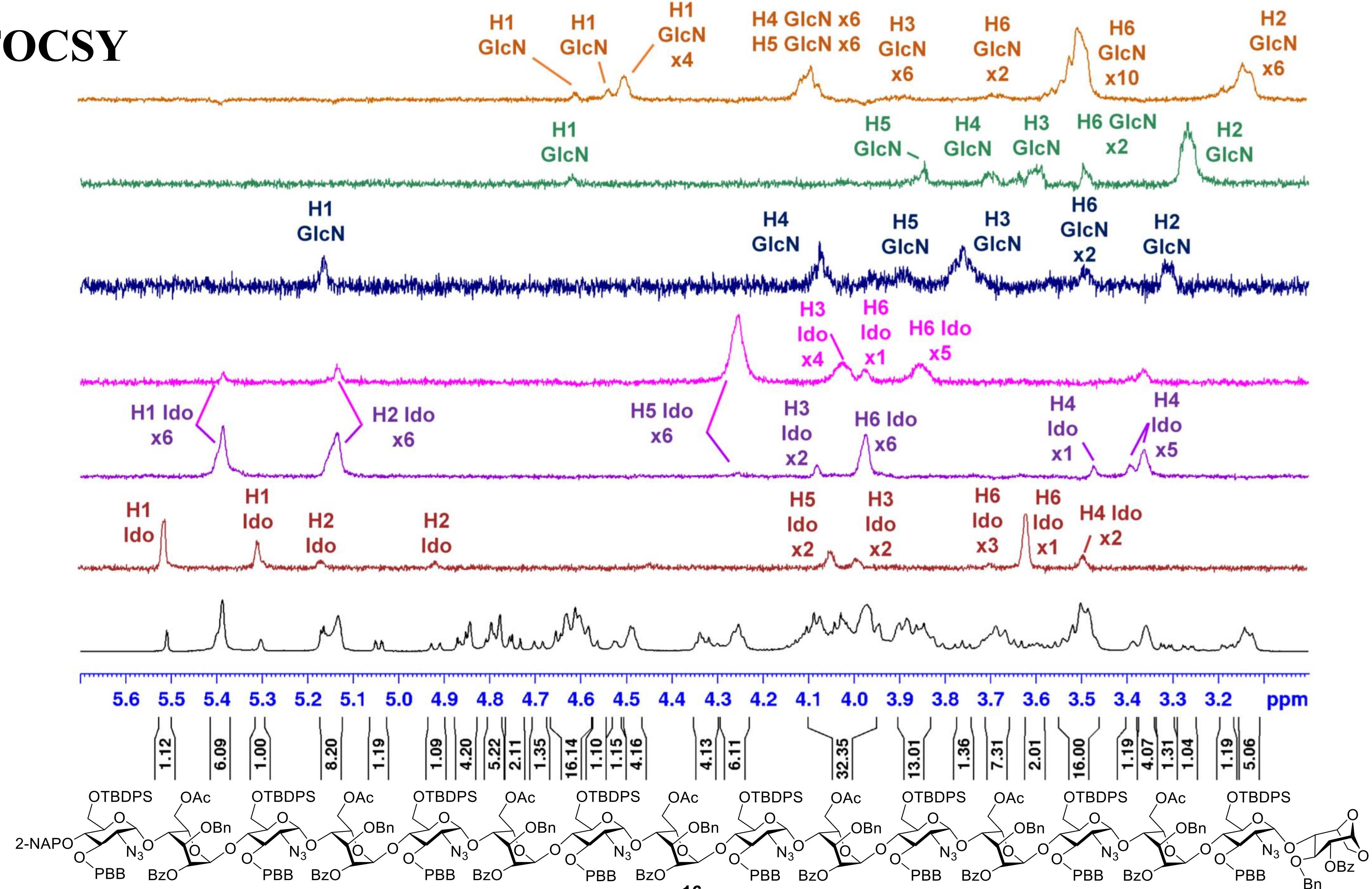

H1 GlcN
H1 GlcN
H1 GlcN x4
H4 GlcN x6
H5 GlcN x6
H3 GlcN x6
H6 GlcN x2
H6 GlcN x10
H2 GlcN x6
H1 GlcN
H5 GlcN
H4 GlcN
H3 GlcN
H6 GlcN x2
H2 GlcN
H1 GlcN
H4 GlcN
H5 GlcN
H3 GlcN
H6 GlcN x2
H2 GlcN
H3 Ido x4
H6 Ido x1
H6 Ido x5
H1 Ido x6
H2 Ido x6
H5 Ido x6
H3 Ido x2
H6 Ido x6
H4 Ido x1
H4 Ido x5
H1 Ido
H1 Ido
H2 Ido
H2 Ido
H5 Ido x2
H3 Ido x2
H6 Ido x3
H6 Ido x1
H4 Ido x2
5.6 5.5 5.4 5.3 5.2 5.1 5.0 4.9 4.8 4.7 4.6 4.5 4.4 4.3 4.2 4.1 4.0 3.9 3.8 3.7 3.6 3.5 3.4 3.3 3.2 ppm
1.12 6.09 1.00 8.20 1.19 1.09 4.20 5.22 2.11 1.35 16.14 1.10 1.15 4.16 4.13 6.11 32.35 13.01 1.36 7.31 2.01 16.00 1.19 4.07 1.31 1.04 1.19 5.06
OTBDPS
OAc
OBn
2-NAPO
N_3
PBB
BzO
OBz
Bn

16

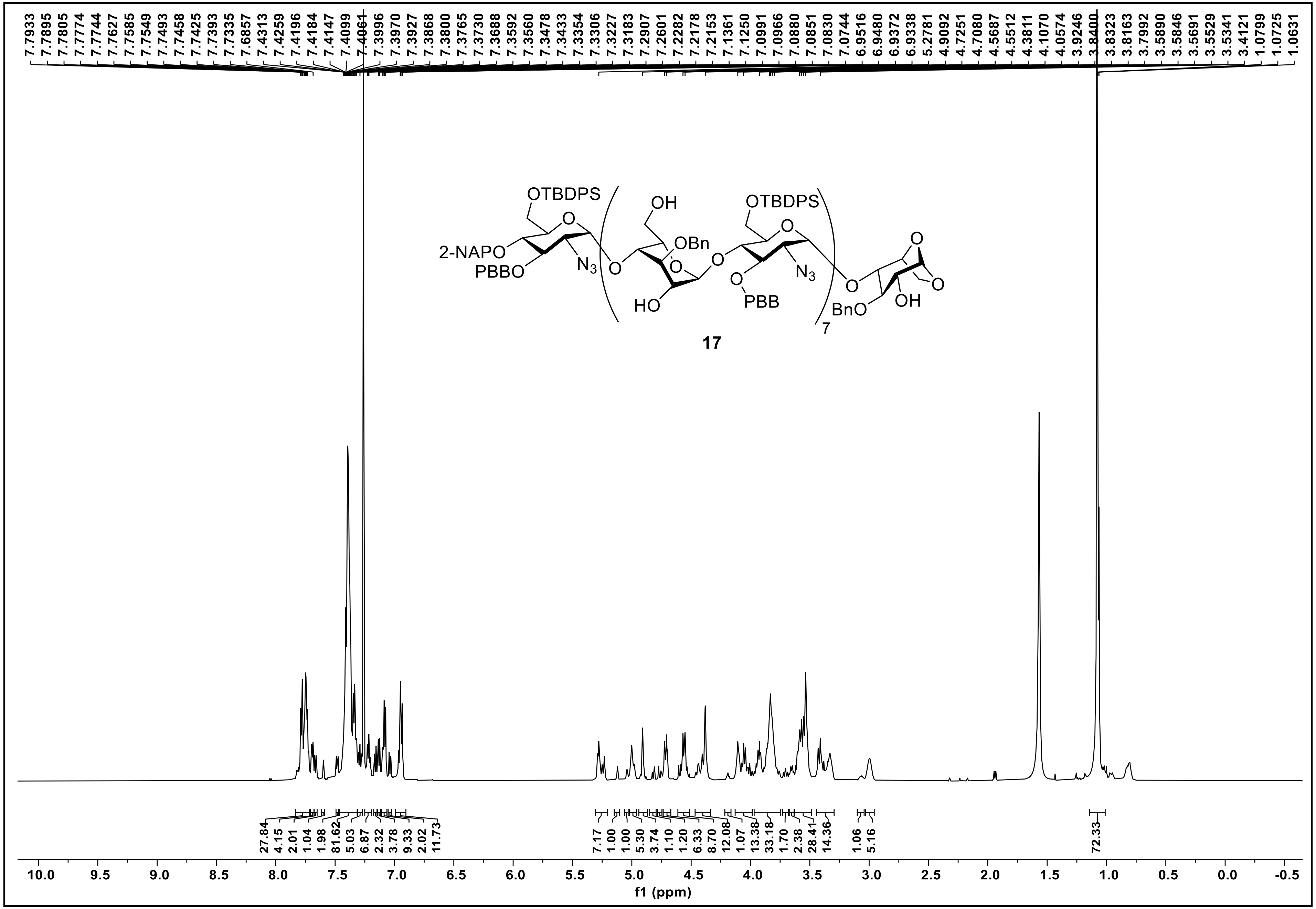
OTBDPS
2-NAPO
PBBO
N3
OH
OBn
HO
O
PBB
7
BnO
OH
17
f1 (ppm)

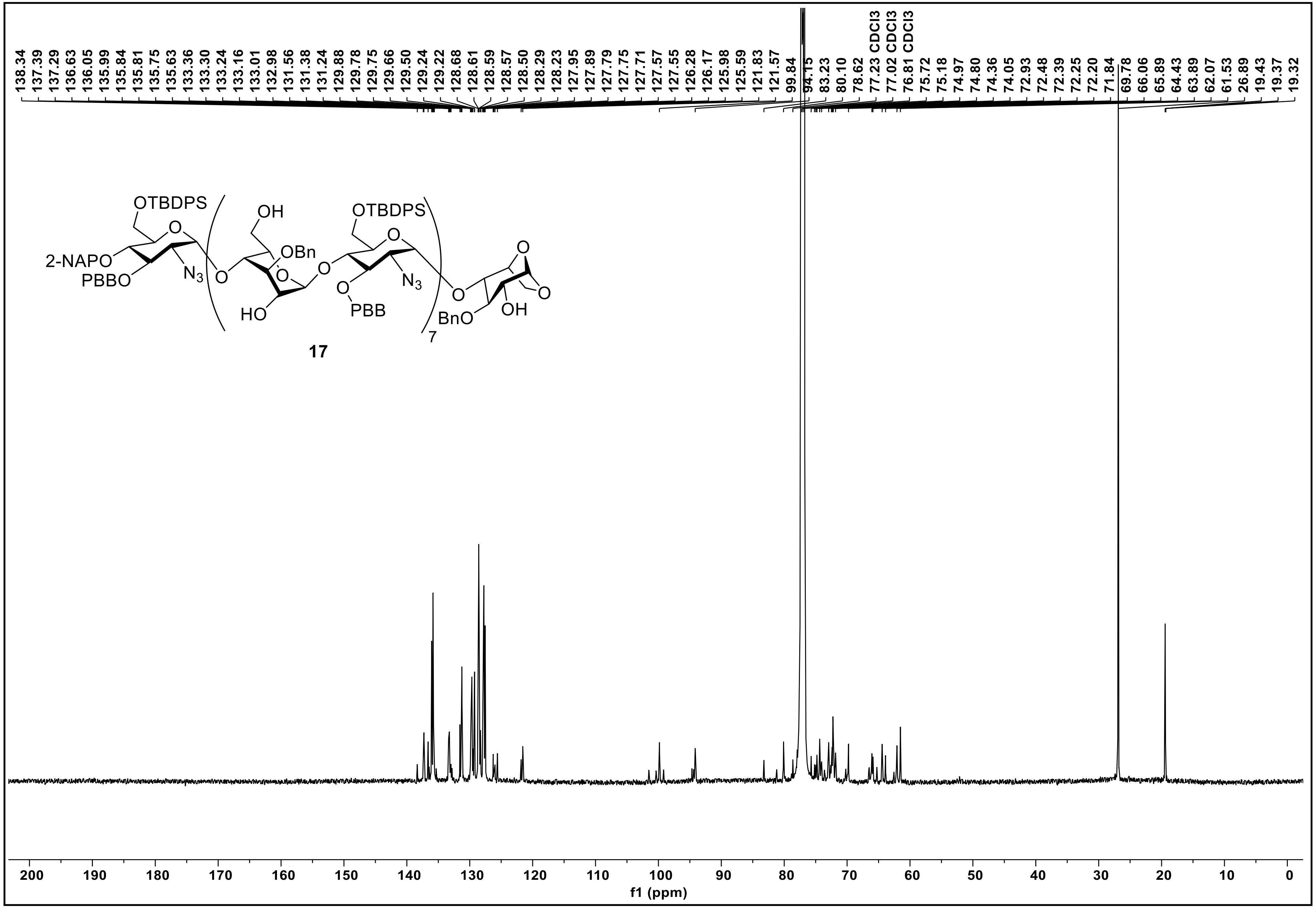
138.34
137.39
137.29
136.63
136.05
135.99
135.84
135.81
135.75
135.63
133.36
133.30
133.24
133.16
133.01
132.98
131.56
131.38
131.24
129.88
129.78
129.75
129.66
129.50
129.24
129.22
128.68
128.61
128.59
128.57
128.50
128.29
128.23
127.95
127.89
127.79
127.75
127.71
127.57
127.55
126.28
126.17
125.98
125.59
121.83
121.57
99.84
94.15
83.23
80.10
78.62
77.23 CDCl3
77.02 CDCl3
76.81 CDCl3
75.72
75.18
74.97
74.80
74.36
74.05
72.93
72.48
72.39
72.25
72.20
71.84
69.78
66.06
65.89
64.43
63.89
62.07
61.53
26.89
19.43
19.37
19.32
OTBDPS
2-NAPO
PBBO
N_3
O
OH
OBn
HO
PBB
7
BnO
OH
17
f1 (ppm)

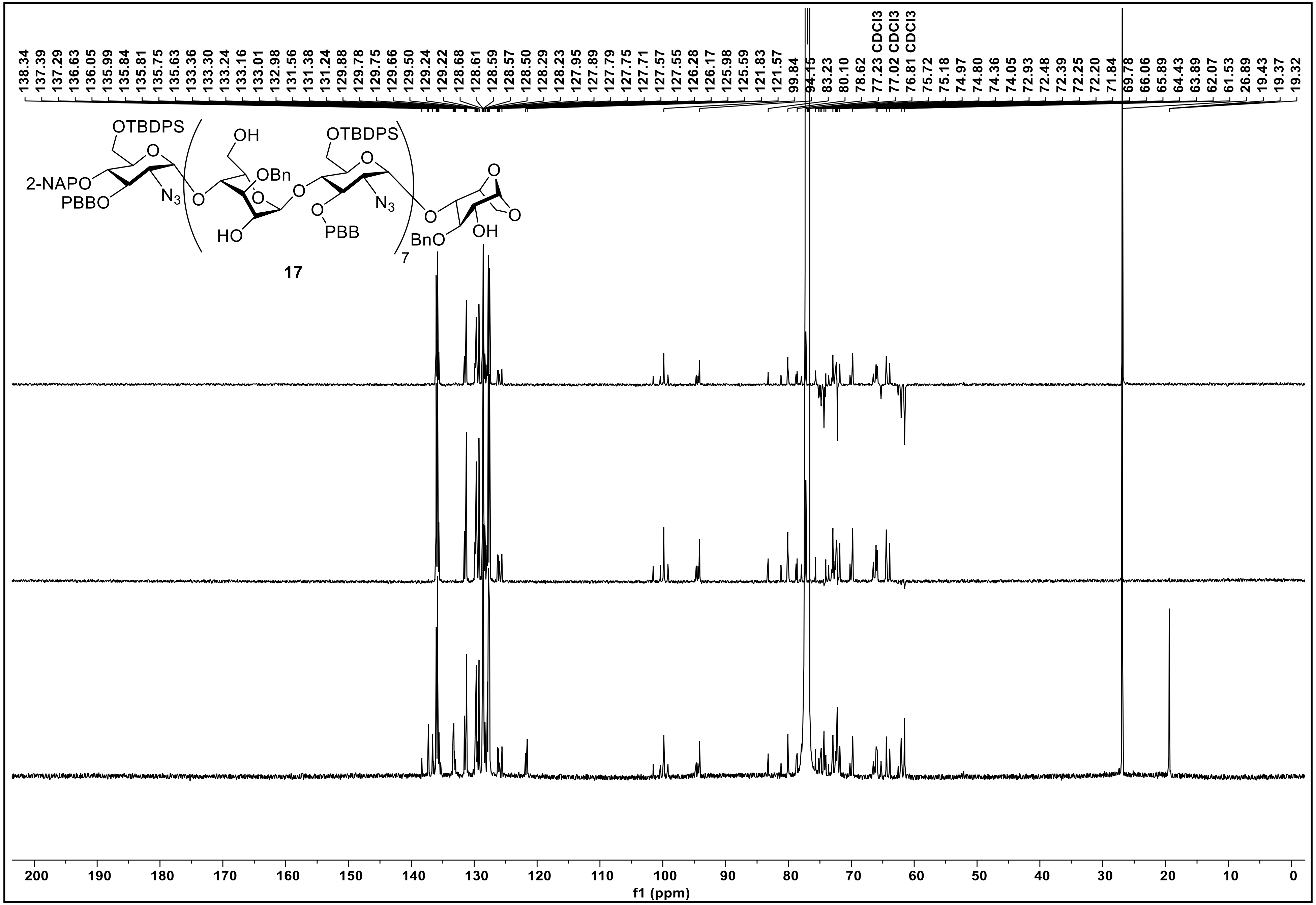

HRMS-MALDI

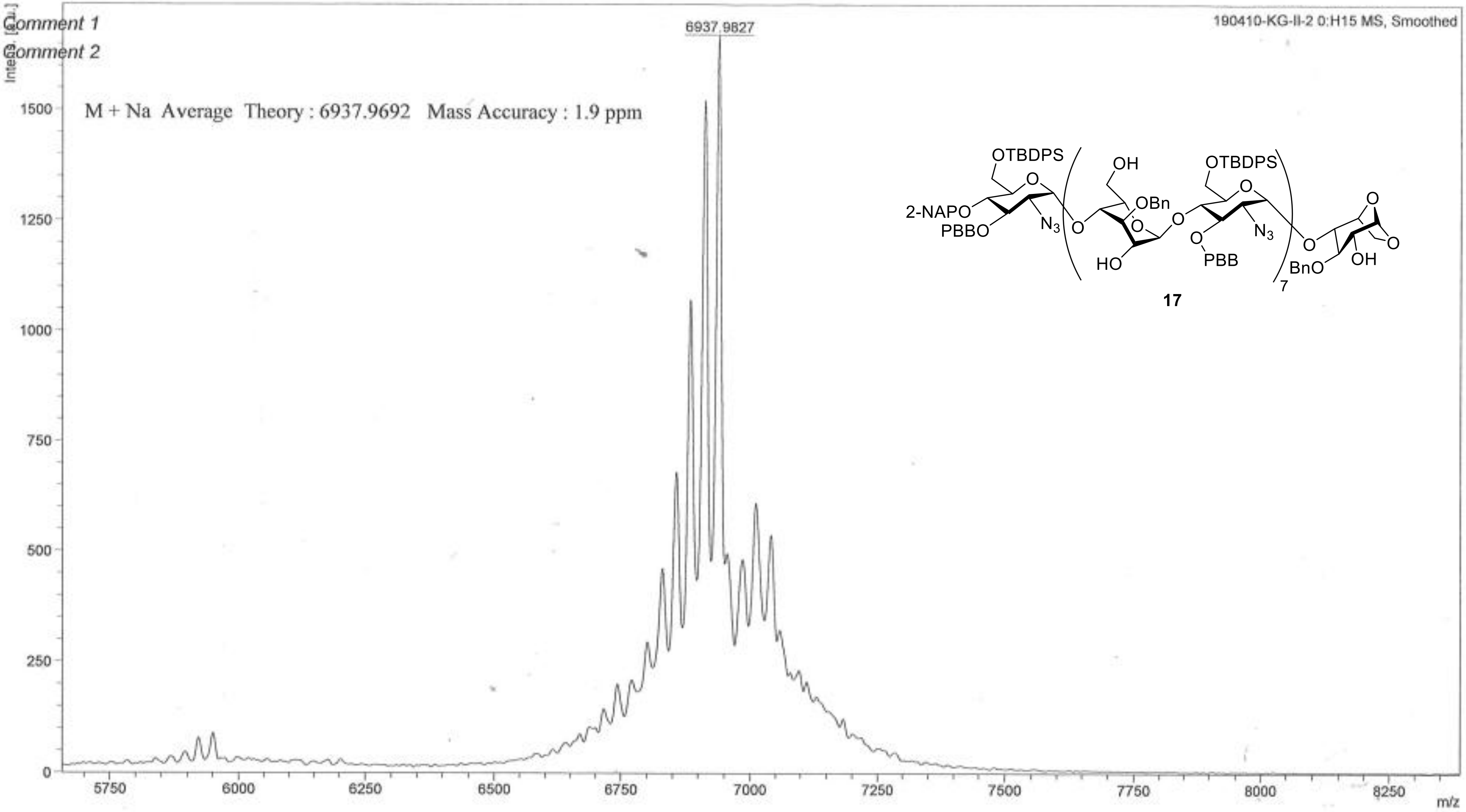
Comment 1
Comment 2
Intens. [a.u.]
6937.9827
190410-KG-II-2 0:H15 MS, Smoothed
M + Na Average Theory : 6937.9692 Mass Accuracy : 1.9 ppm
1500
1250
1000
750
500
250
0
5750
6000
6250
6500
6750
7000
7250
7500
7750
8000
8250
m/z
OTBDPS
2-NAPO
PBBO
N_3
OH
OBn
HO
O
PBB
7
BnO
OH
17

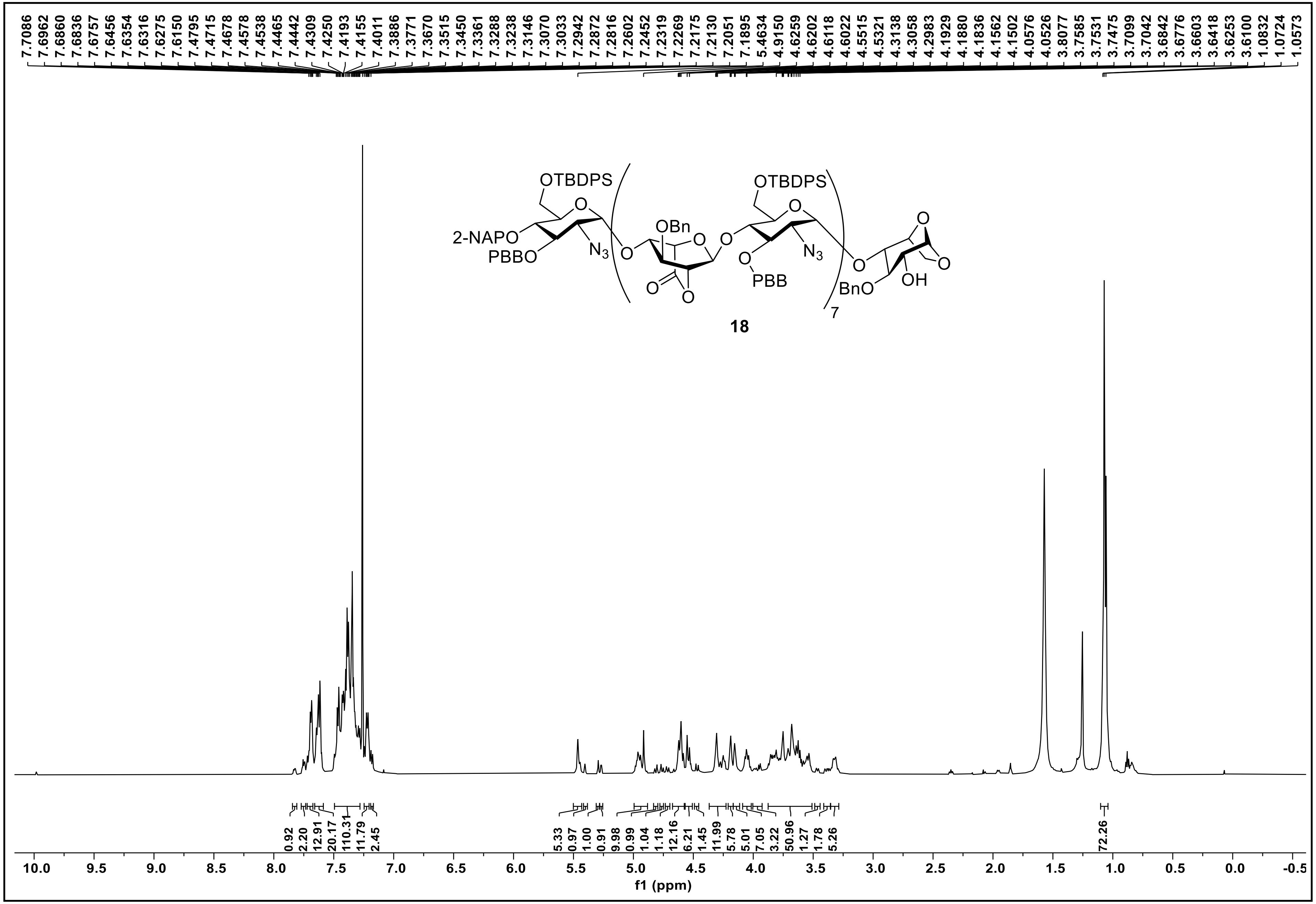

OTBDPS
2-NAPO
PBBO
N3
OBn
O
PBB
7
BnO
OH
18
f1 (ppm)

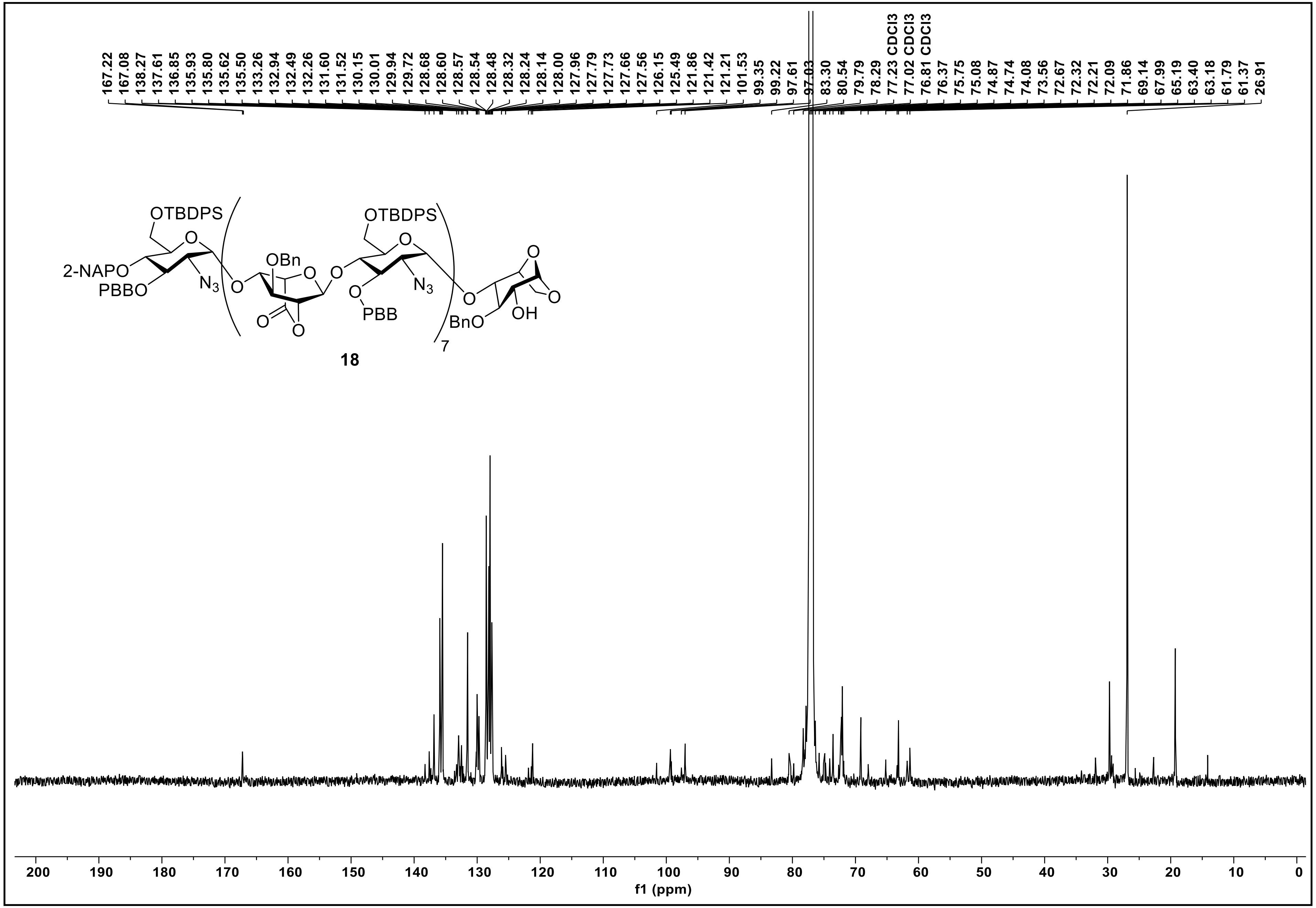
167.22
167.08
138.27
137.61
136.85
135.93
135.80
135.62
135.50
133.26
132.94
132.49
132.26
131.60
131.52
130.15
130.01
129.94
129.72
128.68
128.60
128.57
128.54
128.48
128.32
128.24
128.14
128.00
127.96
127.79
127.73
127.66
127.56
126.15
125.49
121.86
121.42
121.21
101.53
99.35
99.22
97.61
97.03
83.30
80.54
79.79
78.29
77.23 CDCl3
77.02 CDCl3
76.81 CDCl3
76.37
75.75
75.08
74.87
74.74
74.08
73.56
72.67
72.32
72.21
72.09
71.86
69.14
67.99
65.19
63.40
63.18
61.79
61.37
26.91
OTBDPS
2-NAPO
PBBO
N3
OBn
O
PBB
BnO
OH
7
18
f1 (ppm)

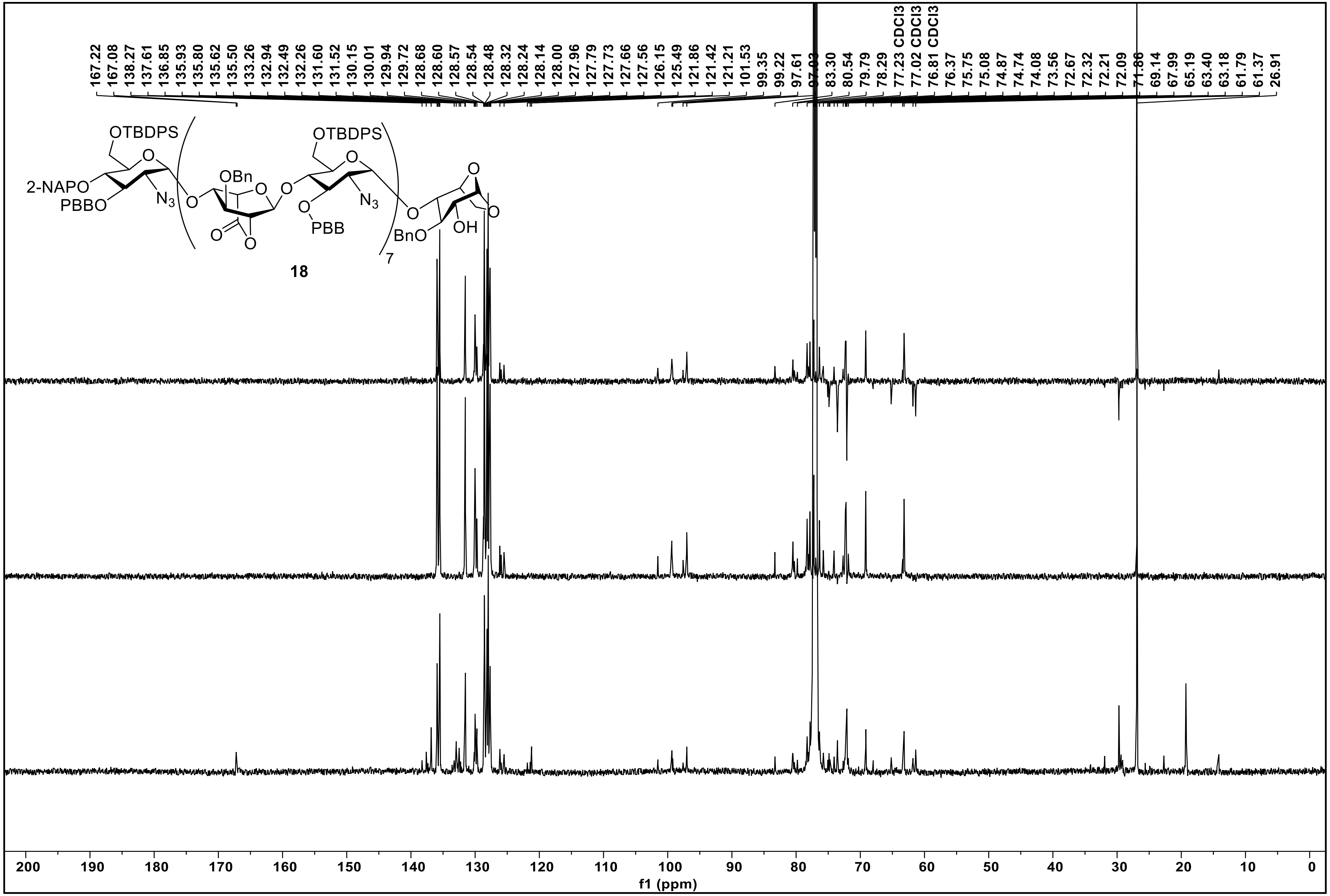
167.22 167.08 138.27 137.61 136.85 135.93 135.80 135.62 135.50 133.26 132.94 132.49 132.26 131.60 131.52 130.15 130.01 129.94 129.72 128.68 128.60 128.57 128.54 128.48 128.32 128.24 128.14 128.00 127.96 127.79 127.73 127.66 127.56 126.15 125.49 121.86 121.42 121.21 101.53 99.35 99.22 97.61 83.30 80.54 79.79 78.29 77.23 CDCl3 77.02 CDCl3 76.81 CDCl3 76.37 75.75 75.08 74.87 74.74 74.08 73.56 72.67 72.32 72.21 72.09 71.86 69.14 67.99 65.19 63.40 63.18 61.79 61.37 26.91
OTBDPS
2-NAPO
PBBO
N3
OBn
PBB
BnO
OH
7
18
200 190 180 170 160 150 140 130 120 110 100 90 80 70 60 50 40 30 20 10 0
f1 (ppm)

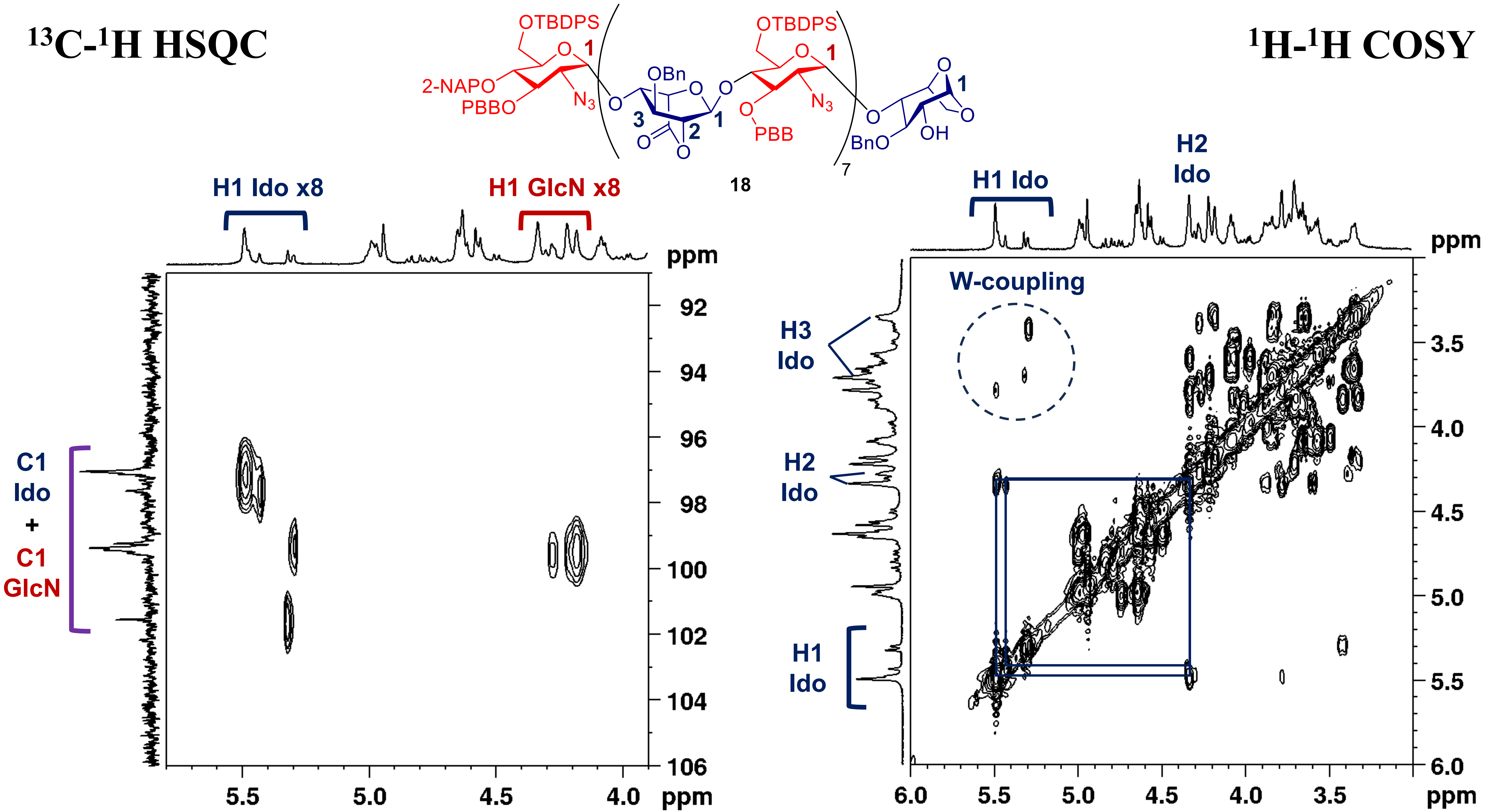

^{13}C-^{1}H HSQC
^{1}H-^{1}H COSY
OTBDPS
2-NAPO
PBBO
N_3
OBn
O
3
2
1
OTBDPS
PBB
BnO
OH
7
18
H1 Ido x8
H1 GlcN x8
ppm
92
94
96
98
100
102
104
106
5.5
5.0
4.5
4.0
C1
Ido
+
C1
GlcN
H1 Ido
H2
Ido
W-coupling
H3
Ido
H2
Ido
H1
Ido
3.5
4.0
4.5
5.0
5.5
6.0

^{13}C-^{1}H HMBC

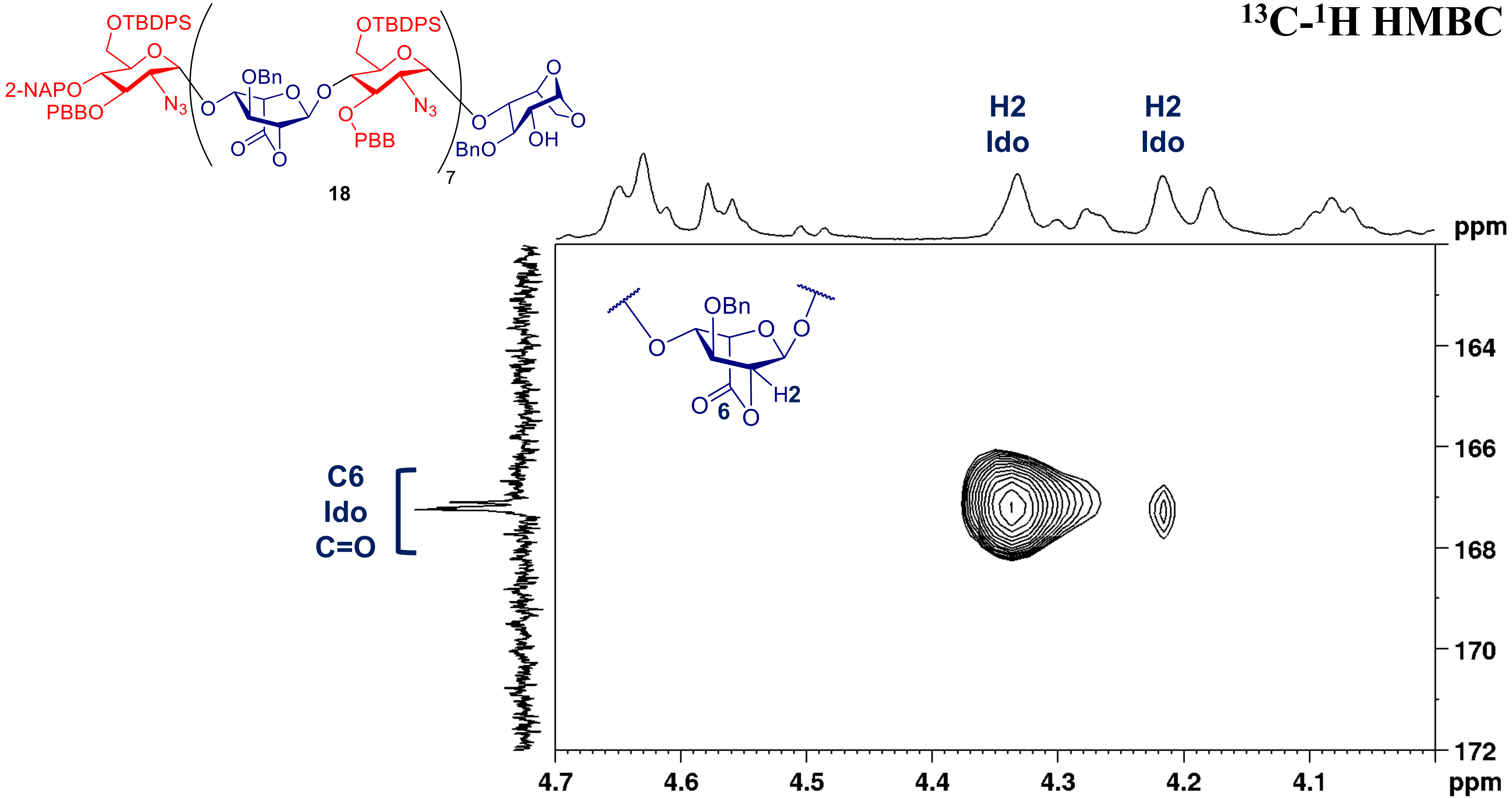
OTBDPS
2-NAPO
PBBO
N3
OBn
O
PBB
7
BnO
OH
18
H2
Ido
H2
Ido
ppm
H2
6
C6
Ido
C=O
164
166
168
170
172
4.7
4.6
4.5
4.4
4.3
4.2
4.1
ppm

HRMS-MALDI

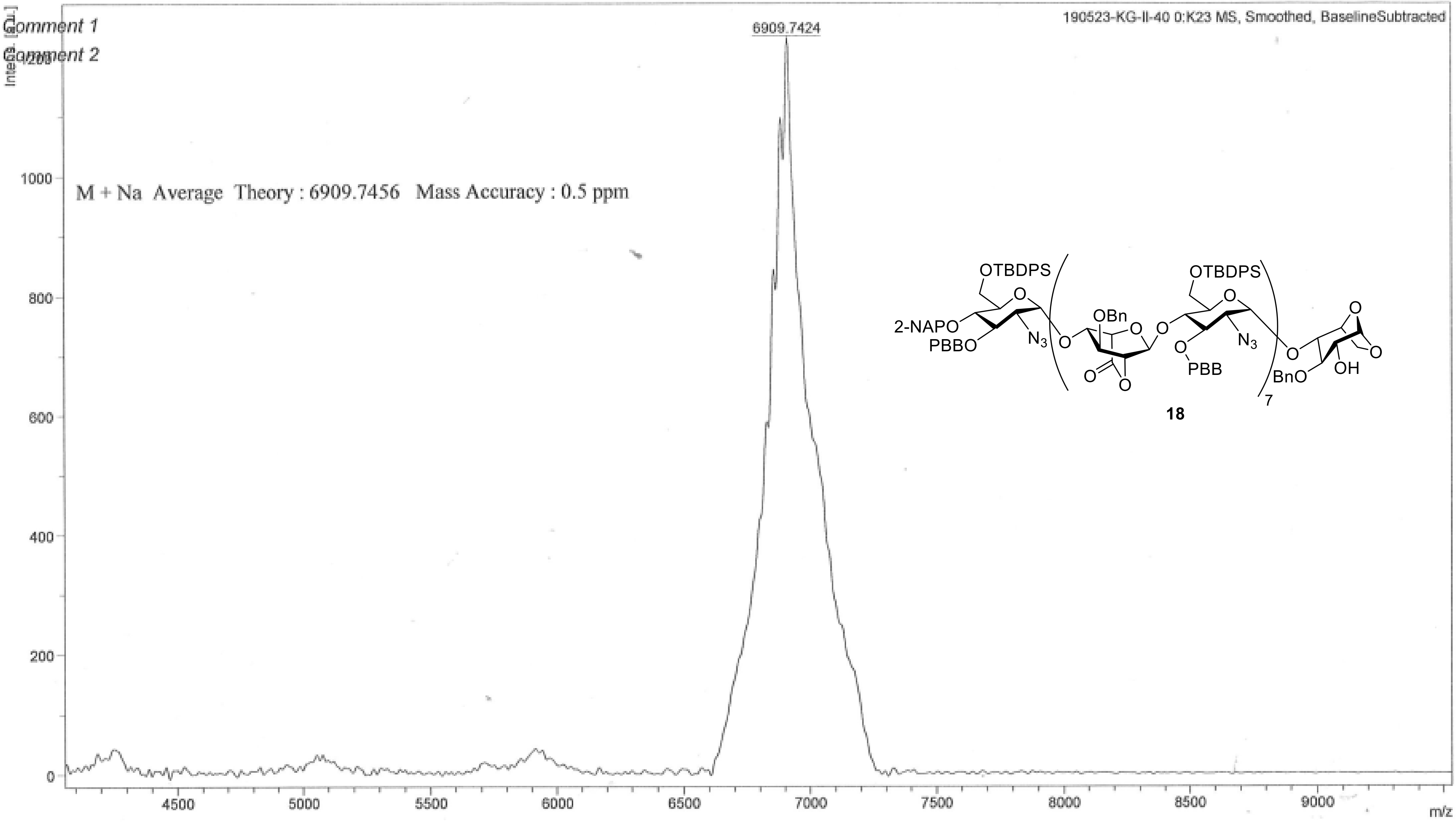
Comment 1
Comment 2
Intens. [a.u.]
190523-KG-II-40 0:K23 MS, Smoothed, BaselineSubtracted
6909.7424
M + Na Average Theory : 6909.7456 Mass Accuracy : 0.5 ppm
1000
800
600
400
200
0
4500
5000
5500
6000
6500
7000
7500
8000
8500
9000
m/z
OTBDPS
2-NAPO
PBBO
N_3
OBn
O
PBB
BnO
OH
7
18

FTIR

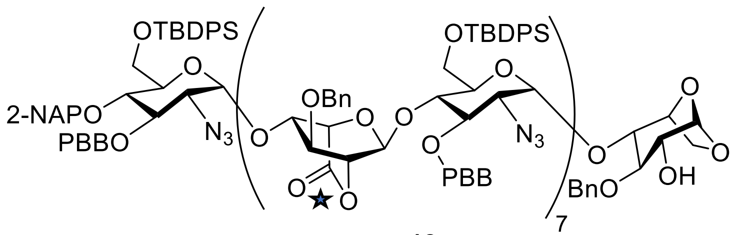

OTBDPS
2-NAPO
PBBO
N3
OBn
OTBDPS
PBB
N3
BnO
OH
7

18

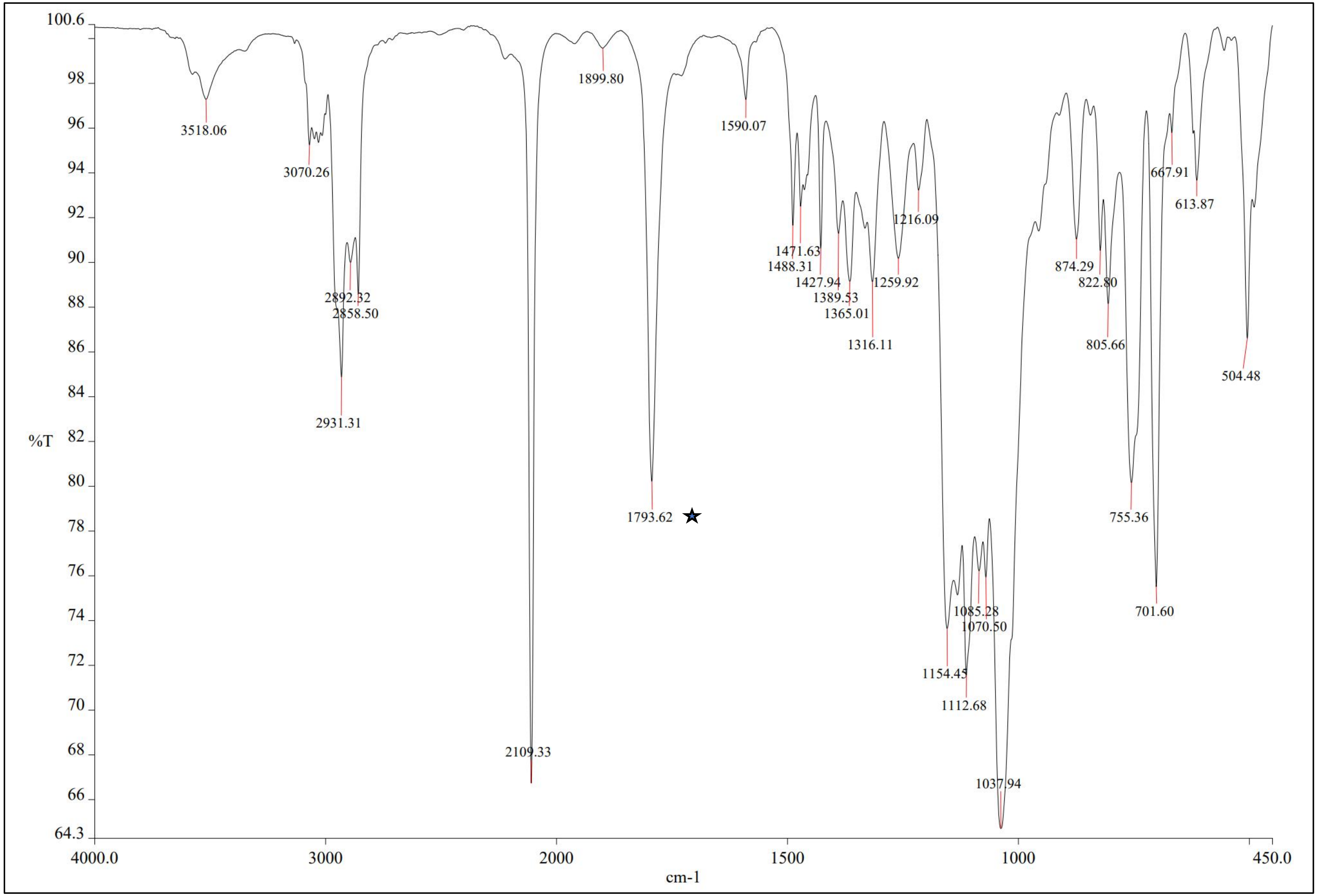

100.6
%T
64.3
4000.0
3000
2000
1500
1000
450.0
cm-1
3518.06
3070.26
2892.32
2858.50
2931.31
2109.33
1899.80
1793.62
1590.07
1471.63
1488.31
1427.94
1389.53
1365.01
1316.11
1259.92
1216.09
1154.45
1112.68
1085.28
1070.50
1037.94
874.29
822.80
805.66
755.36
701.60
667.91
613.87
504.48

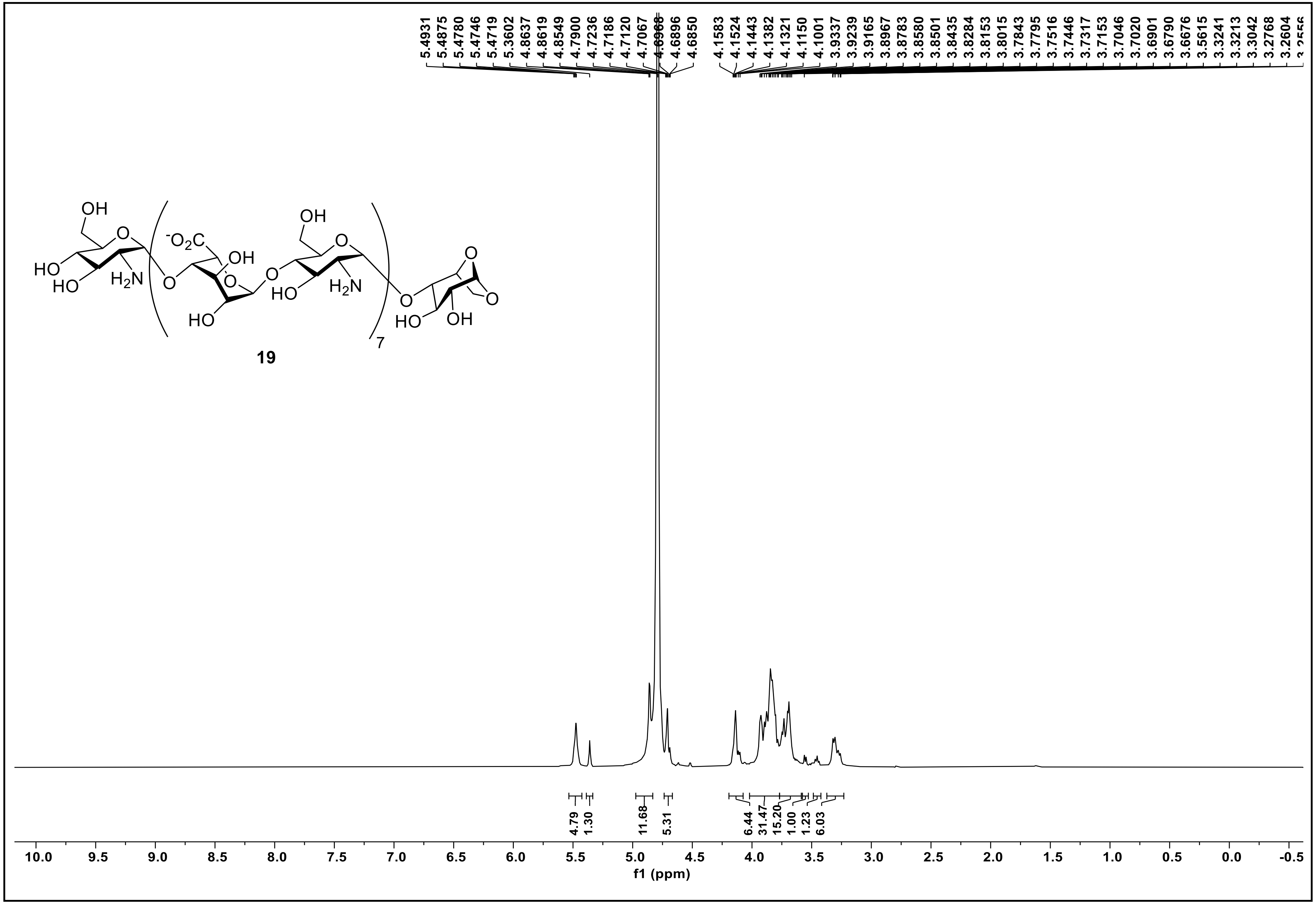
5.4931
5.4875
5.4780
5.4746
5.4719
5.3602
4.8637
4.8619
4.8549
4.7900
4.7236
4.7186
4.7120
4.7067
4.6896
4.6850
4.1583
4.1524
4.1443
4.1382
4.1321
4.1150
4.1001
3.9337
3.9239
3.9165
3.8967
3.8783
3.8580
3.8501
3.8435
3.8284
3.8153
3.8015
3.7843
3.7795
3.7516
3.7446
3.7317
3.7153
3.7046
3.7020
3.6901
3.6790
3.6676
3.5615
3.3241
3.3213
3.3042
3.2768
3.2604
OH
O
HO
HO
H2N
O
-O2C
OH
O
HO
O
OH
O
HO
H2N
7
O
O
O
HO
OH
19
4.79
1.30
11.68
5.31
6.44
31.47
15.20
1.00
1.23
6.03
10.0
9.5
9.0
8.5
8.0
7.5
7.0
6.5
6.0
5.5
5.0
4.5
4.0
3.5
3.0
2.5
2.0
1.5
1.0
0.5
0.0
-0.5
f1 (ppm)

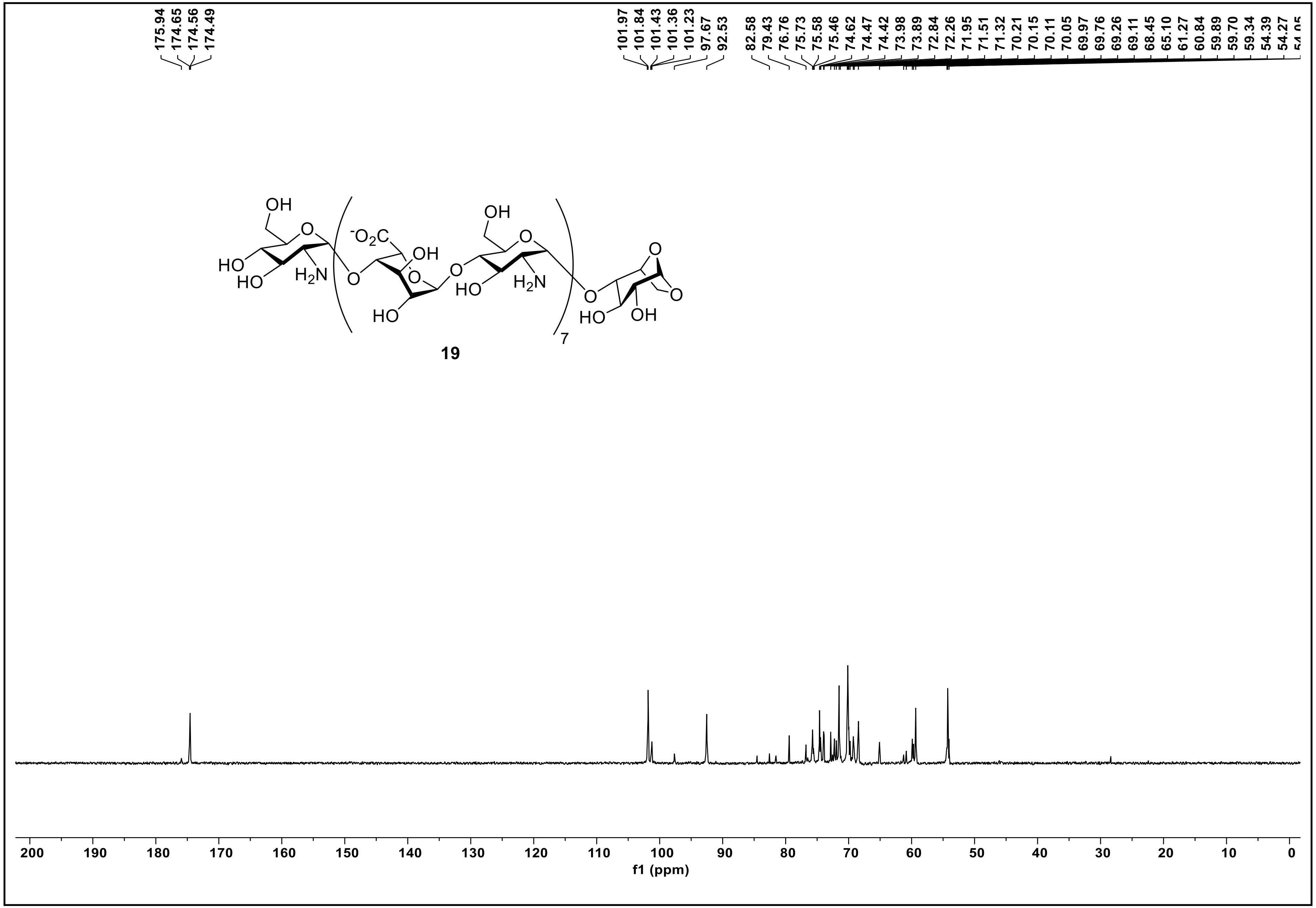
175.94
174.65
174.56
174.49
101.97
101.84
101.43
101.36
101.23
97.67
92.53
82.58
79.43
76.76
75.73
75.58
75.46
74.62
74.47
74.42
73.98
73.89
72.84
72.26
71.95
71.51
71.32
70.21
70.15
70.11
70.05
69.97
69.76
69.26
69.11
68.45
65.10
61.27
60.84
59.89
59.70
59.34
54.39
54.27
f1 (ppm)

19

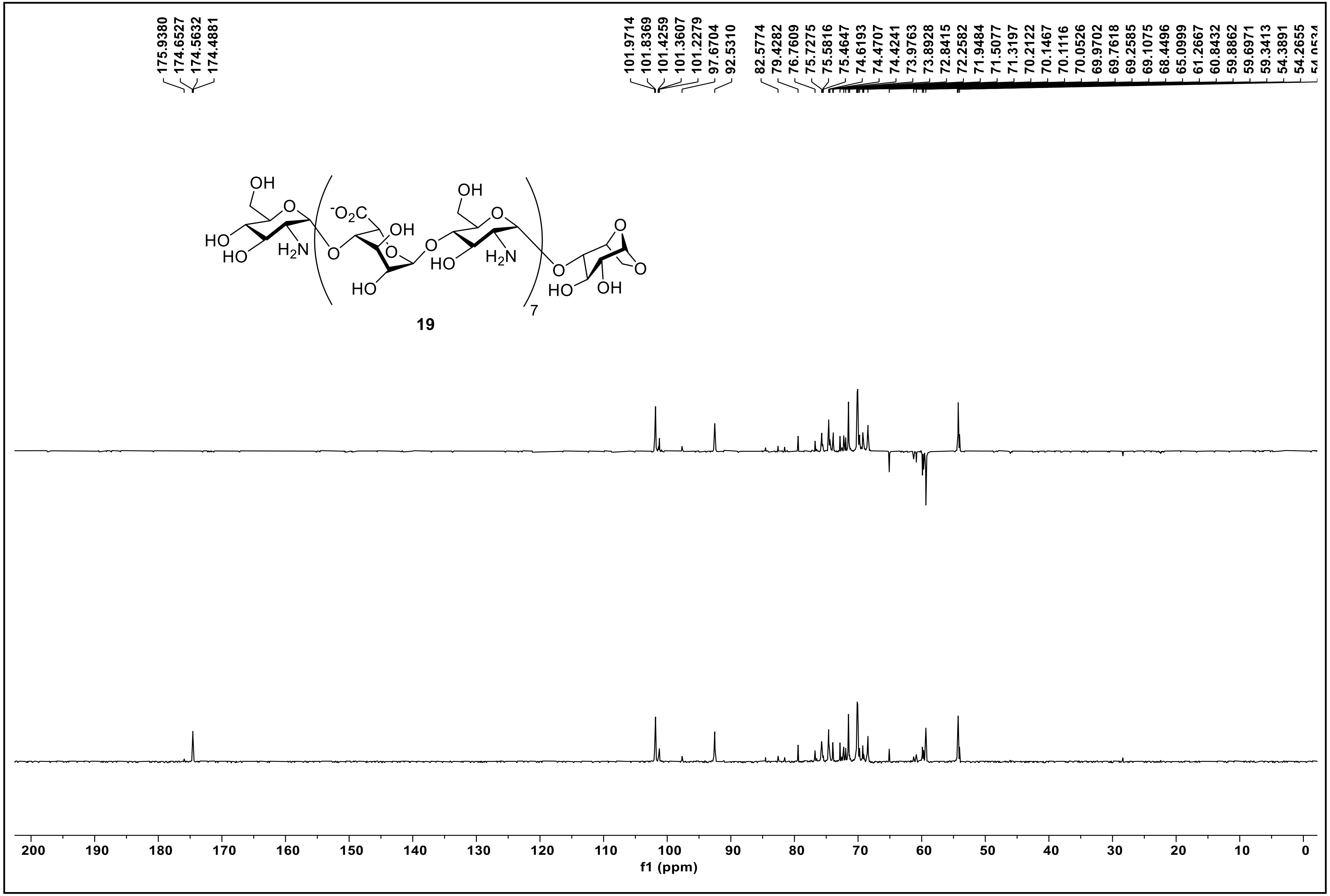
175.9380
174.6527
174.5632
174.4881
101.9714
101.8369
101.4259
101.3607
101.2279
97.6704
92.5310
82.5774
79.4282
76.7609
75.7275
75.5816
75.4647
74.6193
74.4707
74.4241
73.9763
73.8928
72.8415
72.2582
71.9484
71.5077
71.3197
70.2122
70.1467
70.1116
70.0526
69.9702
69.7618
69.2585
69.1075
68.4496
65.0999
61.2667
60.8432
59.8862
59.6971
59.3413
54.3891
54.2655
OH
O
HO
HO
H2N
O
-O2C
OH
O
HO
O
OH
O
HO
H2N
7
O
O
O
HO
OH
19
200
190
180
170
160
150
140
130
120
110
100
90
80
70
60
50
40
30
20
10
0
f1 (ppm)

HRMS-ESI

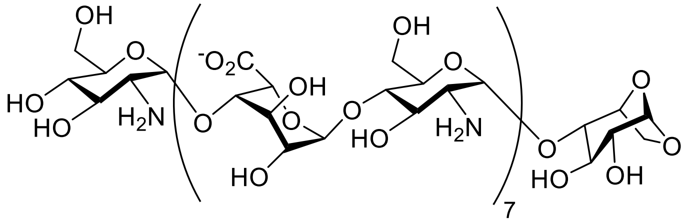

19

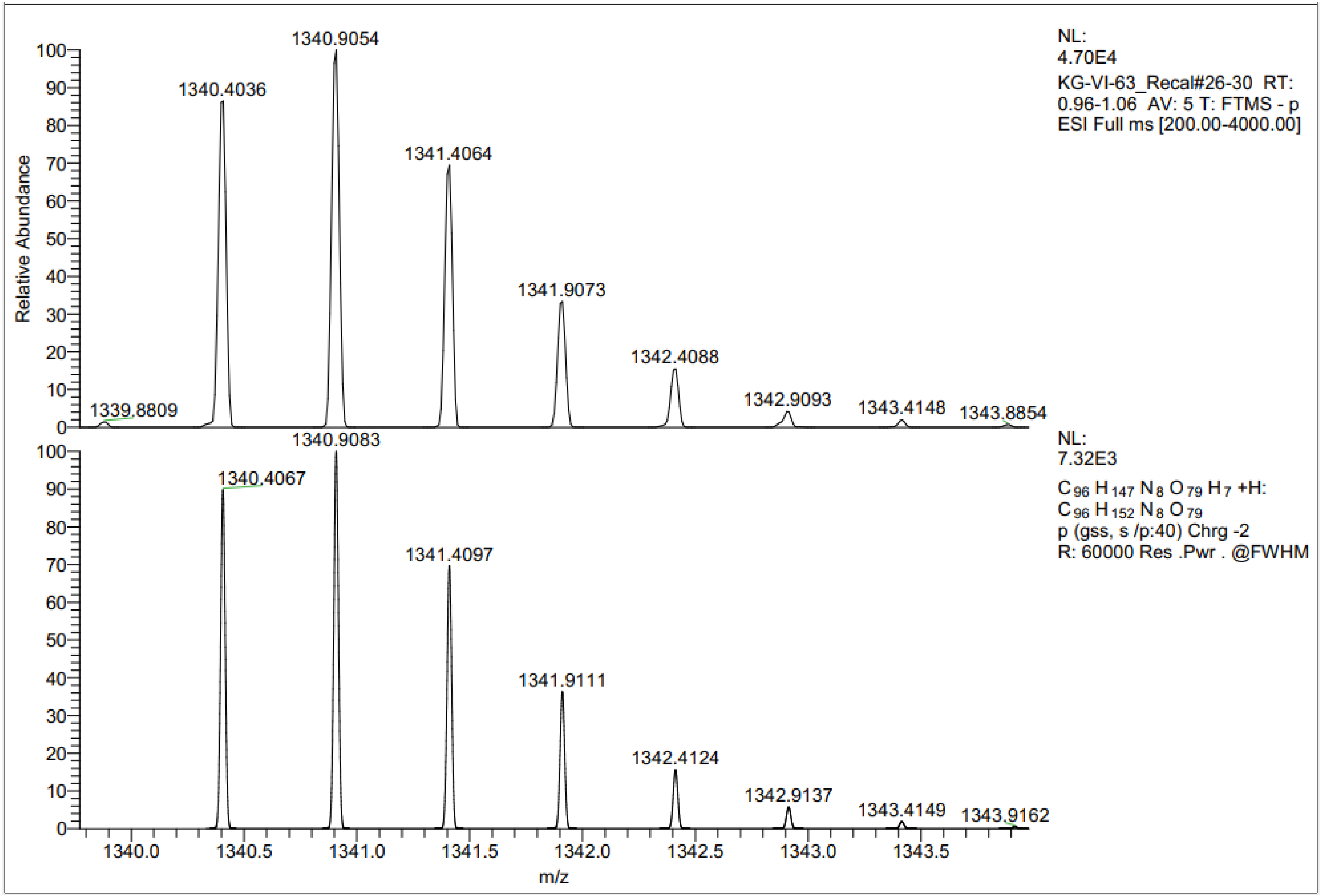

$(M + 5H^+)^{-2}$

Calculated : 1340.9083

Found : 1340.9054

Mass Error : 2.16 ppm

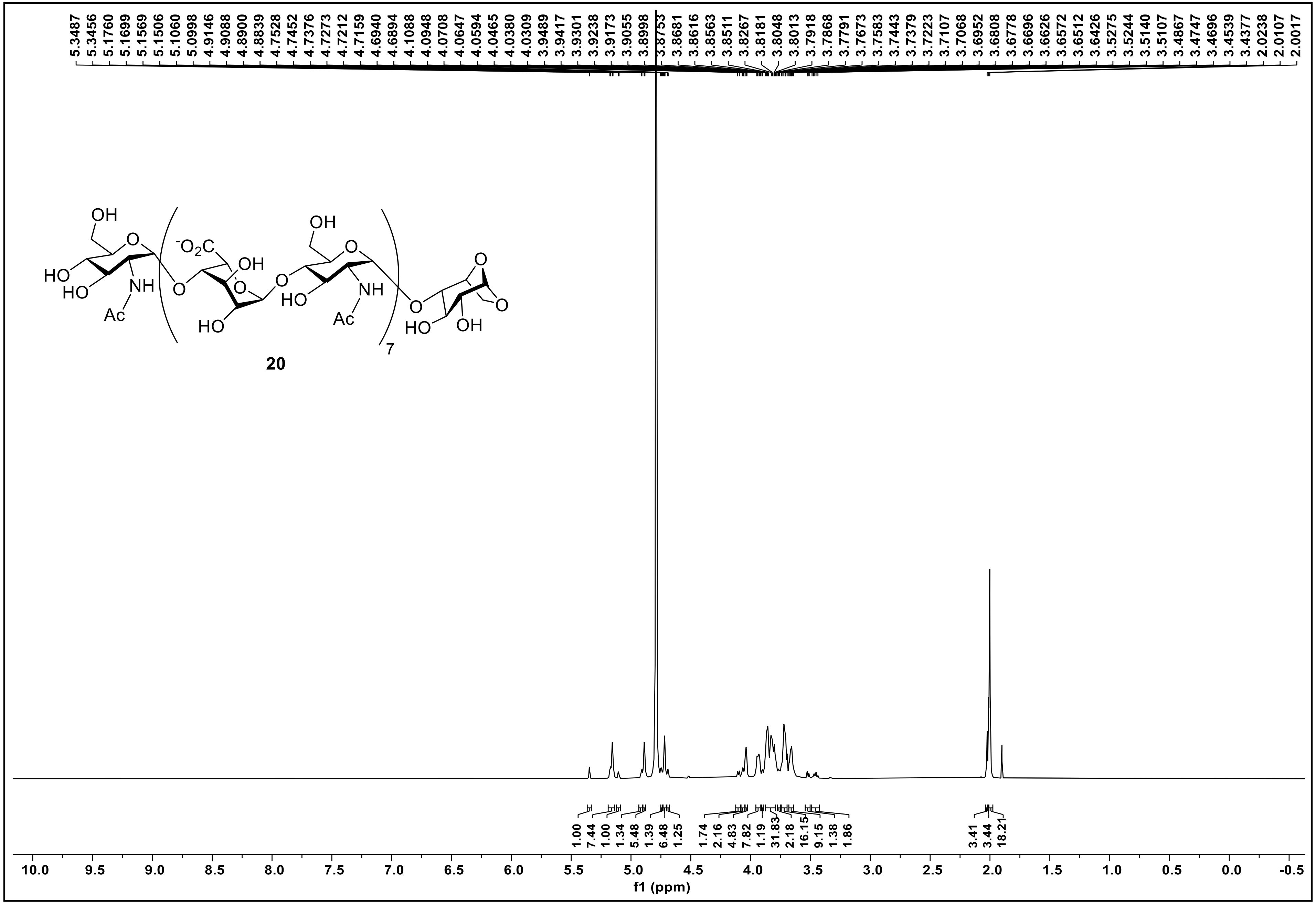
5.3487 5.3456 5.1760 5.1699 5.1569 5.1506 5.1060 5.0998 4.9146 4.9088 4.8900 4.8839 4.7528 4.7452 4.7376 4.7273 4.7212 4.7159 4.6940 4.6894 4.1088 4.0948 4.0708 4.0647 4.0594 4.0465 4.0380 4.0309 3.9489 3.9417 3.9301 3.9238 3.9173 3.9055 3.8998 3.8753 3.8681 3.8616 3.8563 3.8511 3.8267 3.8181 3.8048 3.8013 3.7918 3.7868 3.7791 3.7673 3.7583 3.7443 3.7379 3.7223 3.7107 3.7068 3.6952 3.6808 3.6778 3.6696 3.6626 3.6572 3.6512 3.6426 3.5275 3.5244 3.5140 3.5107 3.4867 3.4747 3.4696 3.4539 3.4377 2.0238 2.0107 2.0017
OH
O
HO
HO
NH
Ac
$^{-}O_2C$
OH
O
O
O
HO
OH
O
HO
NH
Ac
7
O
O
O
HO
OH
20
1.00 7.44 1.00 1.34 5.48 1.39 6.48 1.25 1.74 2.16 4.83 7.82 1.19 31.83 2.18 16.15 9.15 1.38 1.86 3.41 3.44 18.21
10.0 9.5 9.0 8.5 8.0 7.5 7.0 6.5 6.0 5.5 5.0 4.5 4.0 3.5 3.0 2.5 2.0 1.5 1.0 0.5 0.0 -0.5
f1 (ppm)

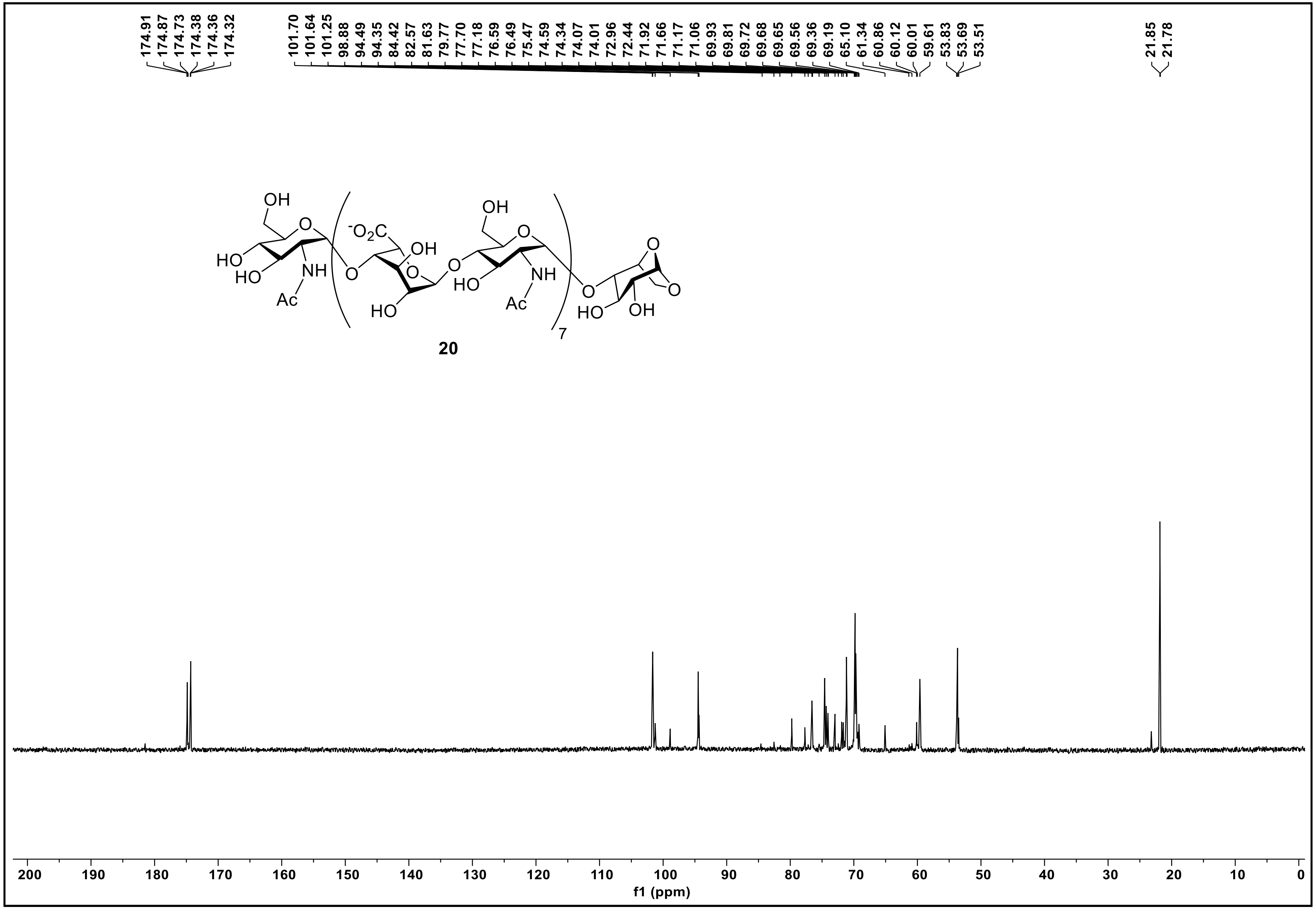
174.91
174.87
174.73
174.38
174.36
174.32
101.70
101.64
101.25
98.88
94.49
94.35
84.42
82.57
81.63
79.77
77.70
77.18
76.59
76.49
75.47
74.59
74.34
74.07
74.01
72.96
72.44
71.92
71.66
71.17
71.06
69.93
69.81
69.72
69.68
69.65
69.56
69.36
69.19
65.10
61.34
60.86
60.12
60.01
59.61
53.83
53.69
53.51
21.85
21.78
20
200 190 180 170 160 150 140 130 120 110 100 90 80 70 60 50 40 30 20 10 0
f1 (ppm)

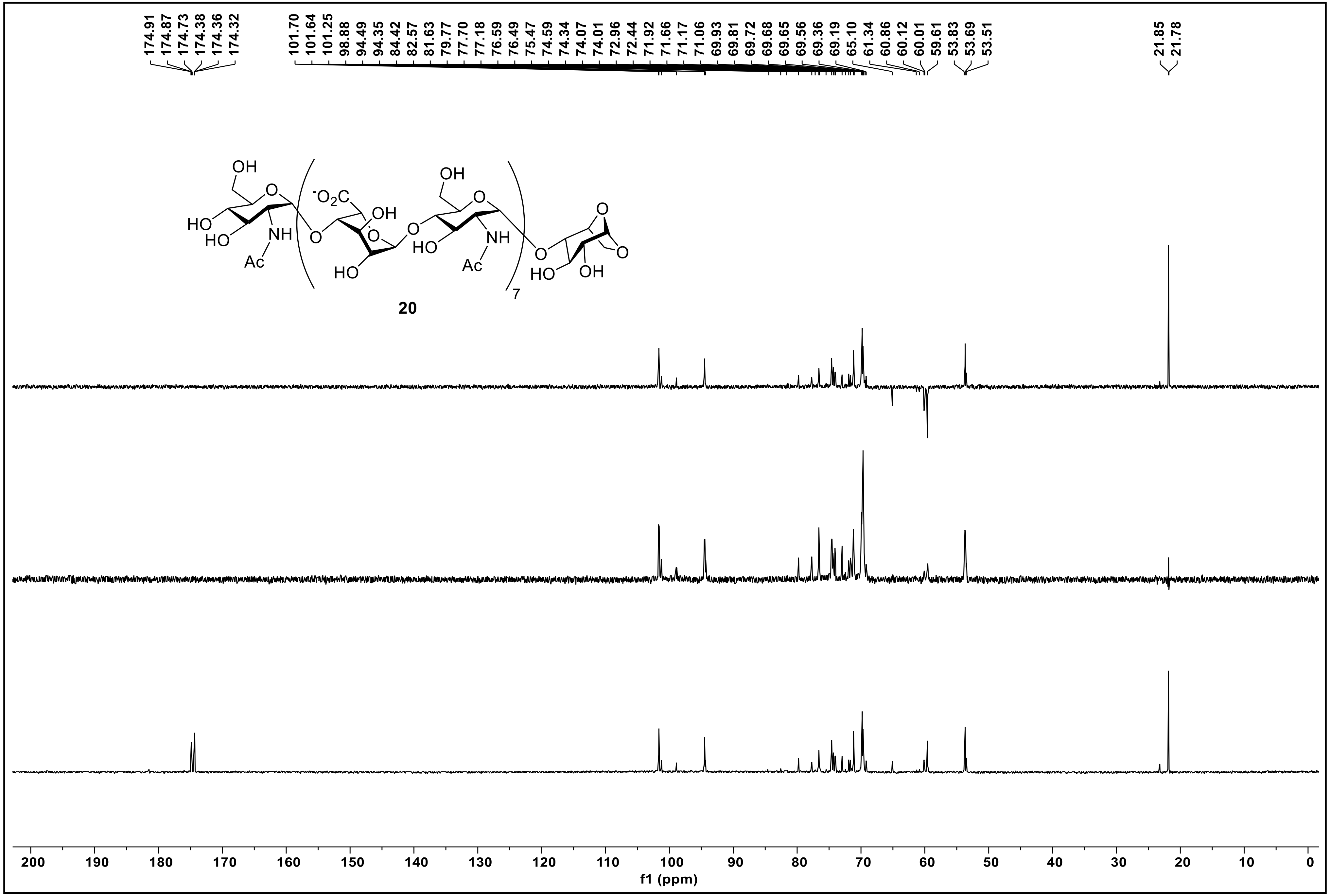

174.91
174.87
174.73
174.38
174.36
174.32
101.70
101.64
101.25
98.88
94.49
94.35
84.42
82.57
81.63
79.77
77.70
77.18
76.59
76.49
75.47
74.59
74.34
74.07
74.01
72.96
72.44
71.92
71.66
71.17
71.06
69.93
69.81
69.72
69.68
69.65
69.56
69.36
69.19
65.10
61.34
60.86
60.12
60.01
59.61
53.83
53.69
53.51
21.85
21.78
20
200 190 180 170 160 150 140 130 120 110 100 90 80 70 60 50 40 30 20 10 0
f1 (ppm)

HRMS-ESI

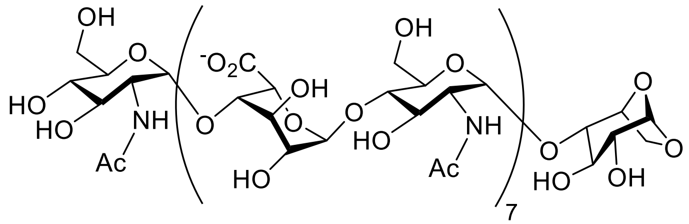

20

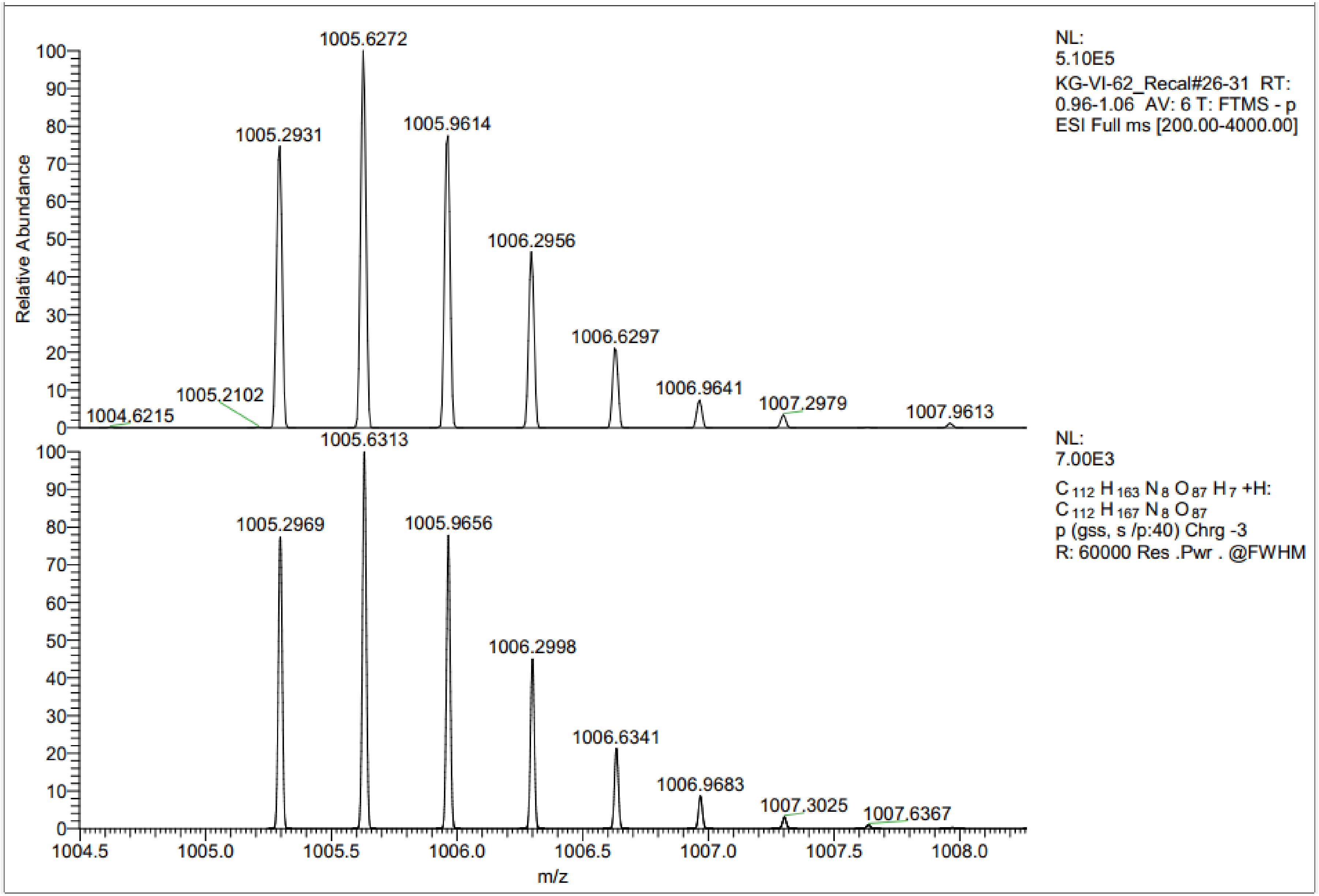

$(M + 4H^+)^{-3}$

Calculated : 1005.2969

Found : 1005.2931

Mass Error : 3.78 ppm

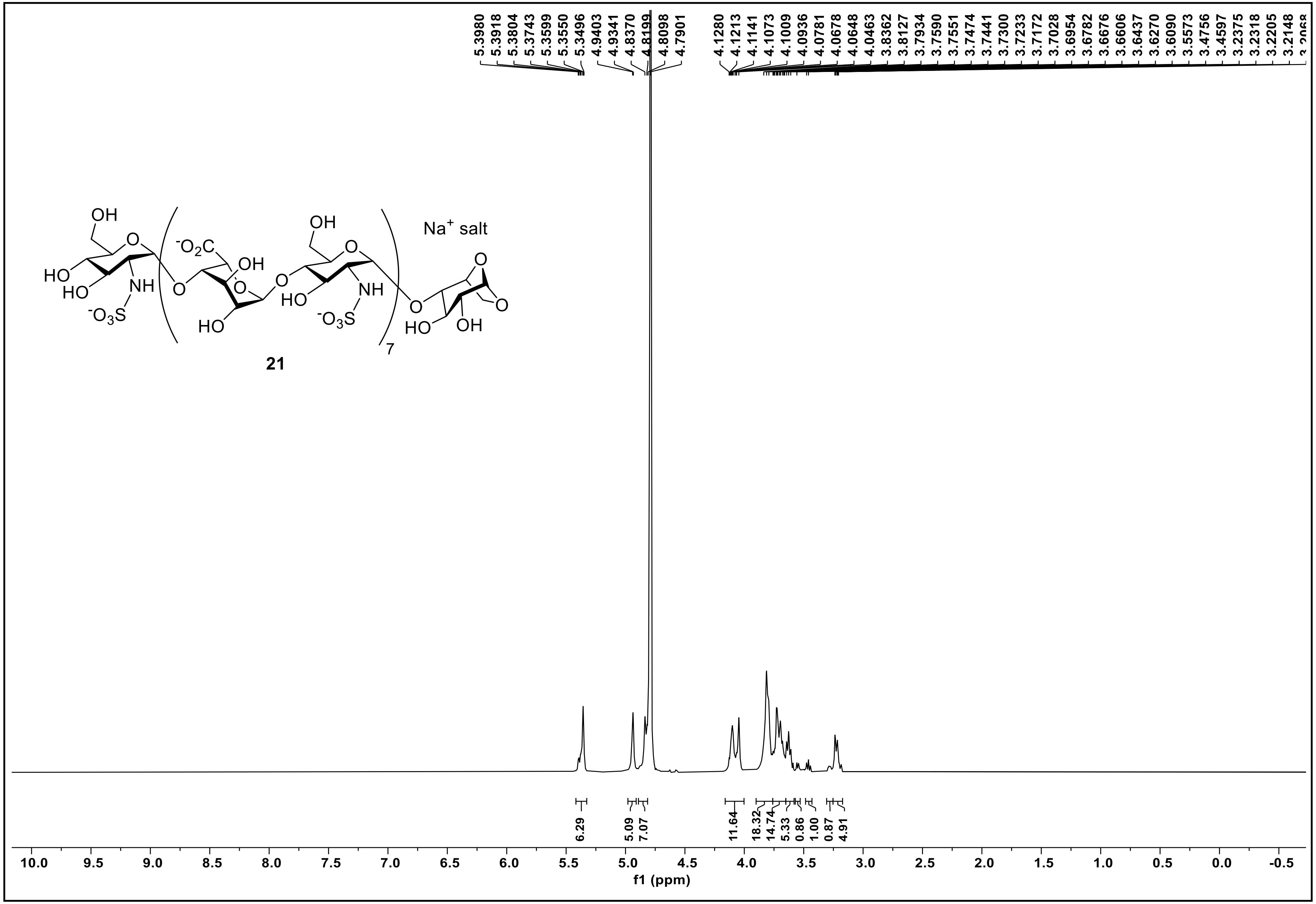
Na+ salt
21
f1 (ppm)

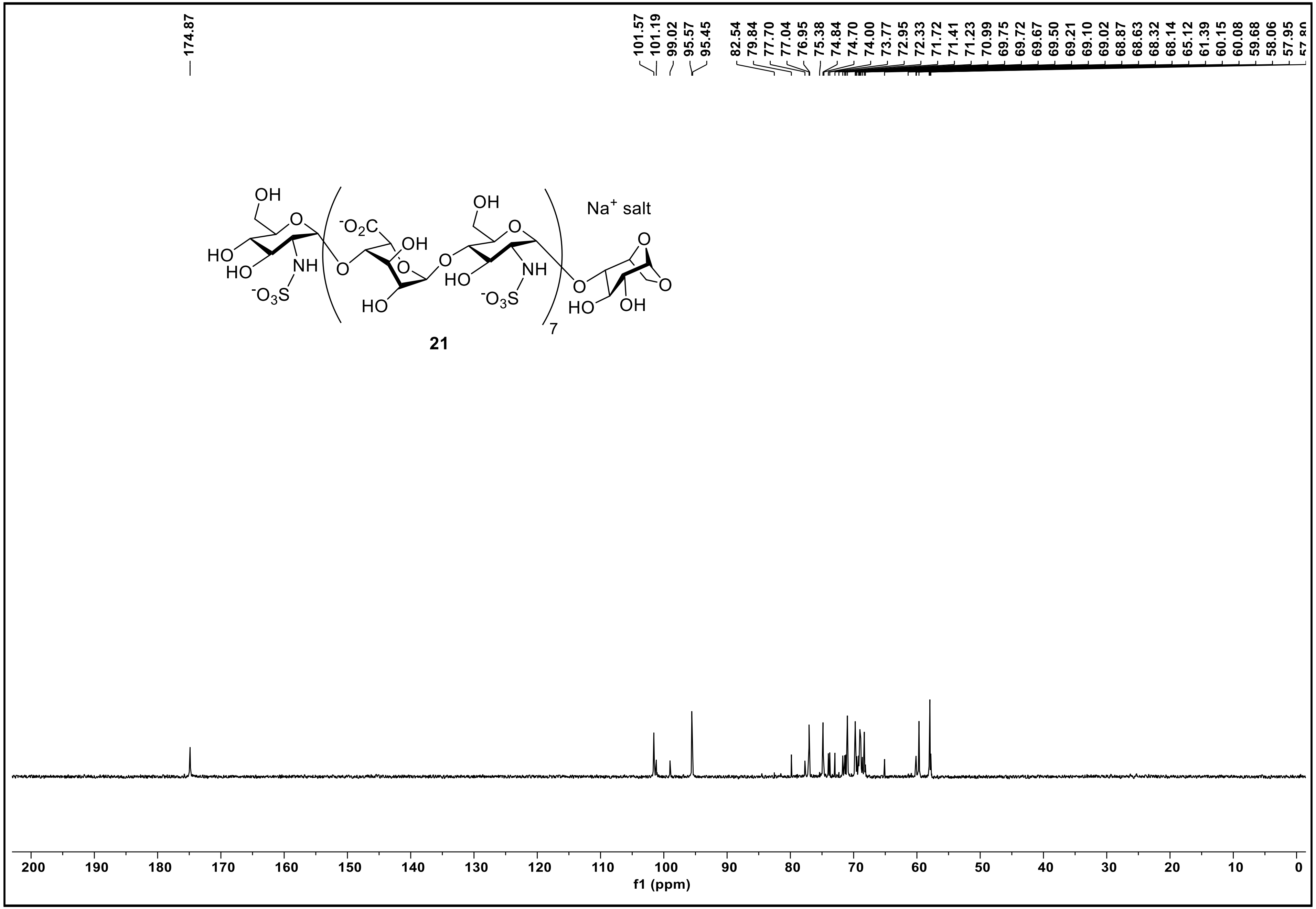
174.87
101.57
101.19
99.02
95.57
95.45
82.54
79.84
77.70
77.04
76.95
75.38
74.84
74.70
74.00
73.77
72.95
72.33
71.72
71.41
71.23
70.99
69.75
69.72
69.67
69.50
69.21
69.10
69.02
68.87
68.63
68.32
68.14
65.12
61.39
60.15
60.08
59.68
58.06
57.95
Na+ salt
21
200 190 180 170 160 150 140 130 120 110 100 90 80 70 60 50 40 30 20 10 0
f1 (ppm)

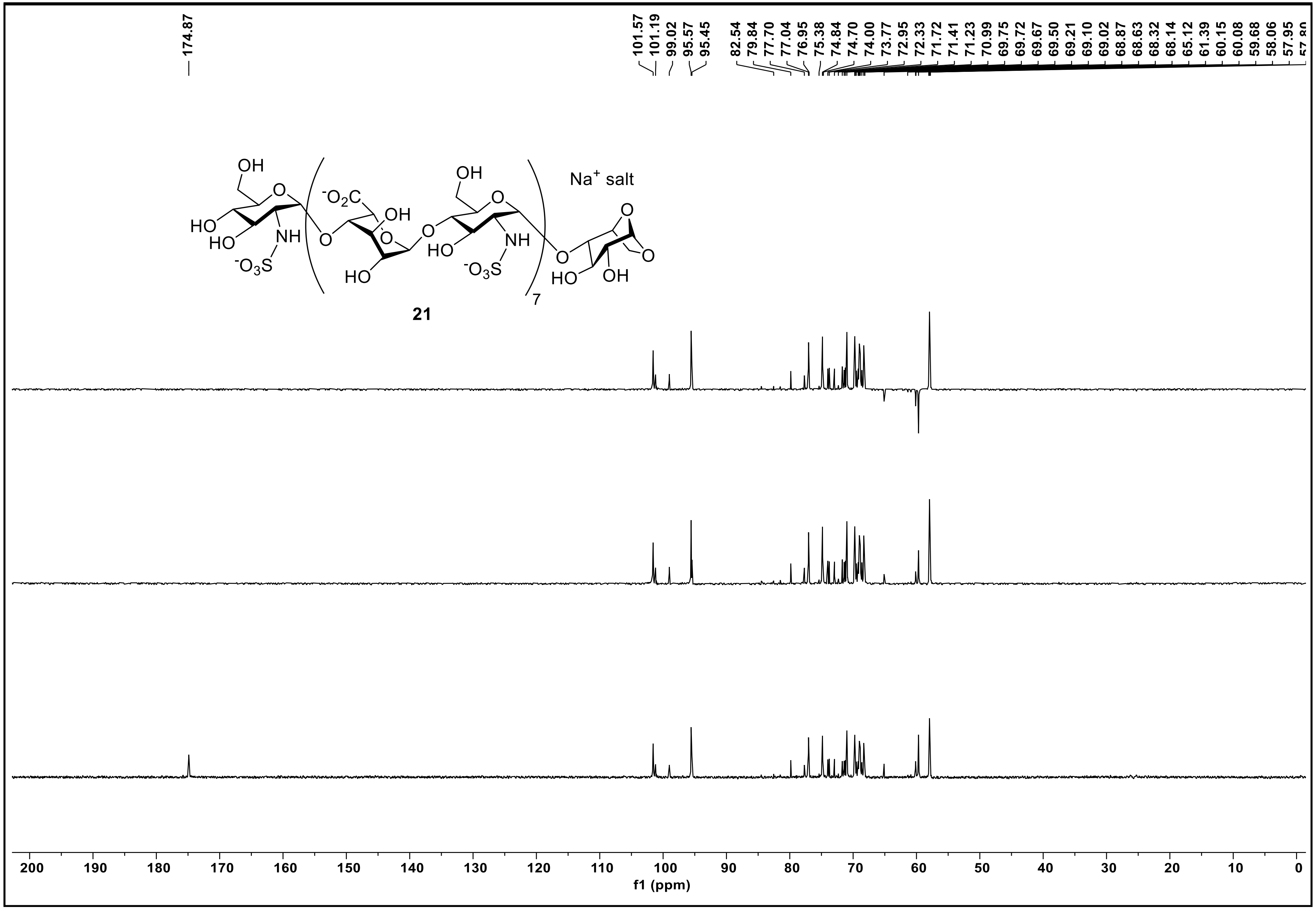
174.87
101.57
101.19
99.02
95.57
95.45
82.54
79.84
77.70
77.04
76.95
75.38
74.84
74.70
74.00
73.77
72.95
72.33
71.72
71.41
71.23
70.99
69.75
69.72
69.67
69.50
69.21
69.10
69.02
68.87
68.63
68.32
68.14
65.12
61.39
60.15
60.08
59.68
58.06
57.95
Na+ salt
21
f1 (ppm)

HRMS-ESI

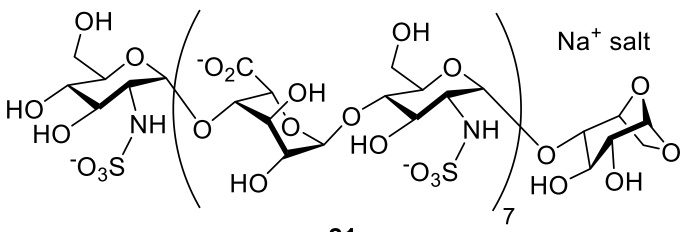

21

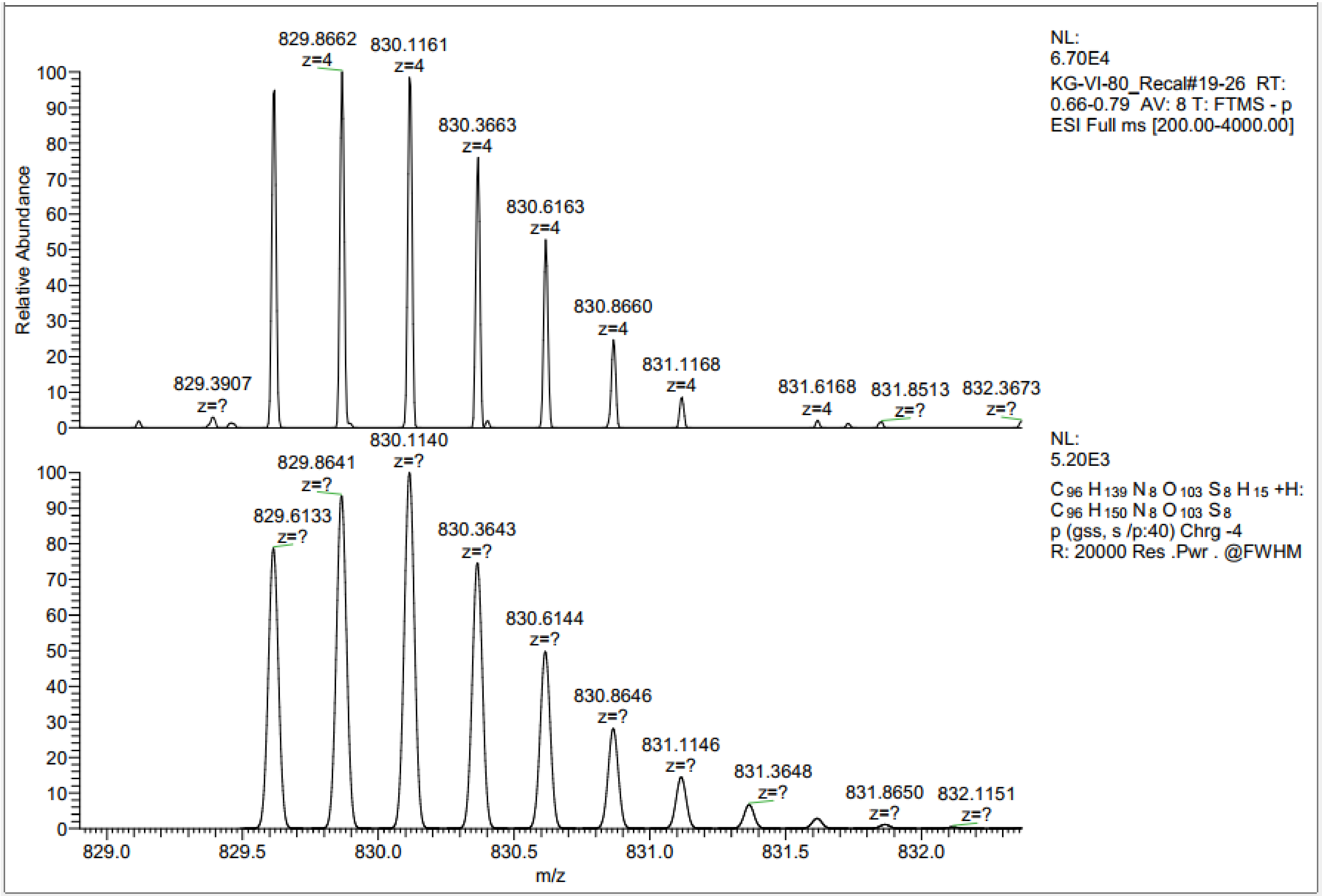

$(M + 11H^+)^{-4}$

Calculated : 830.1140

Found : 830.1161

Mass Error : 2.53 ppm

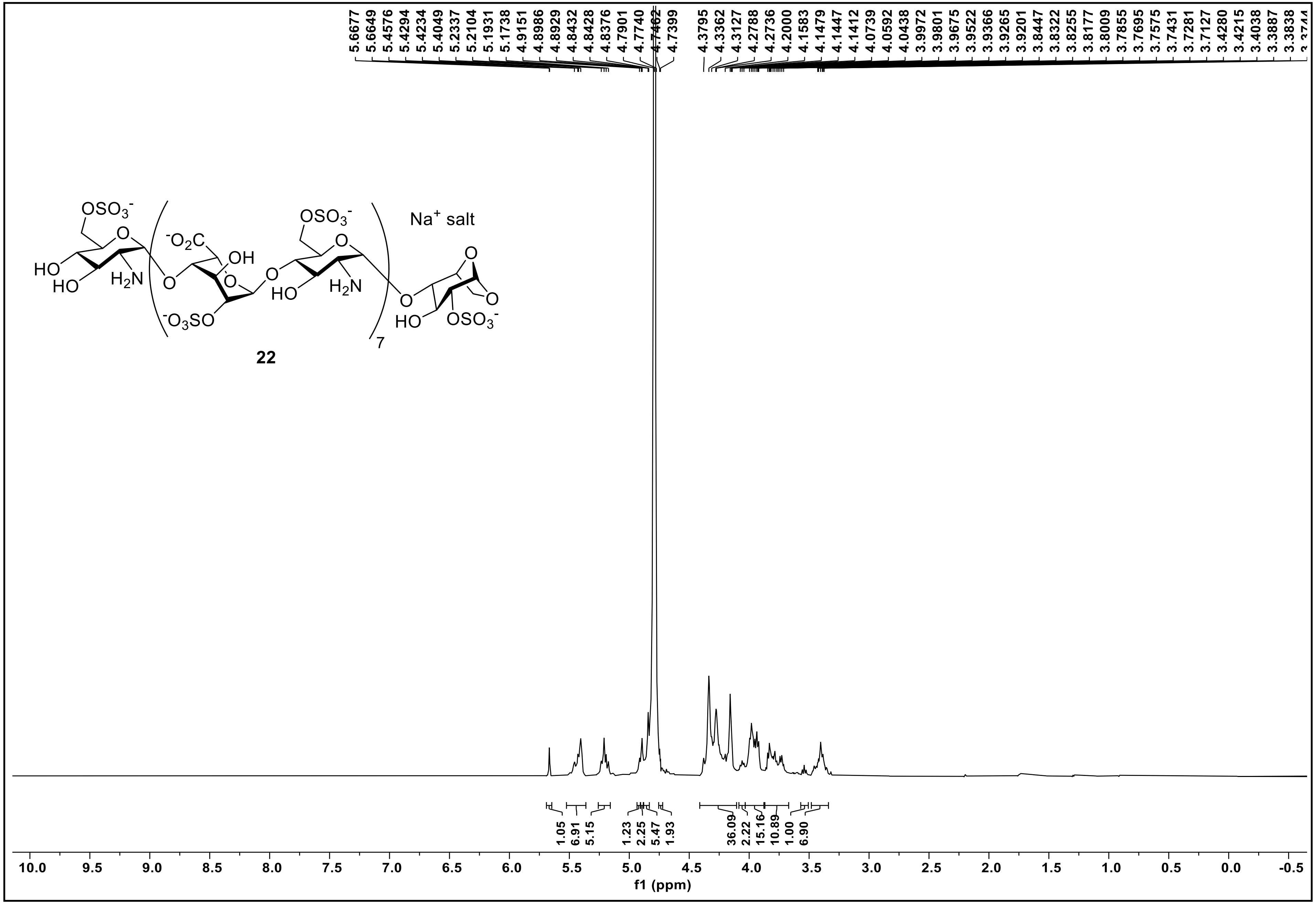
5.6677
5.6649
5.4576
5.4294
5.4234
5.4049
5.2337
5.2104
5.1931
5.1738
4.9151
4.8986
4.8929
4.8432
4.8428
4.8376
4.7901
4.7740
4.7462
4.7399
4.3795
4.3362
4.3127
4.2788
4.2736
4.2000
4.1583
4.1479
4.1447
4.1412
4.0739
4.0592
4.0438
3.9972
3.9801
3.9675
3.9522
3.9366
3.9265
3.9201
3.8447
3.8322
3.8255
3.8177
3.8009
3.7855
3.7695
3.7575
3.7431
3.7281
3.7127
3.4280
3.4215
3.4038
3.3887
3.3838
Na+ salt
22
1.05
6.91
5.15
1.23
2.25
5.47
1.93
36.09
2.22
15.16
10.89
1.00
6.90
10.0
9.5
9.0
8.5
8.0
7.5
7.0
6.5
6.0
5.5
5.0
4.5
4.0
3.5
3.0
2.5
2.0
1.5
1.0
0.5
0.0
-0.5
f1 (ppm)

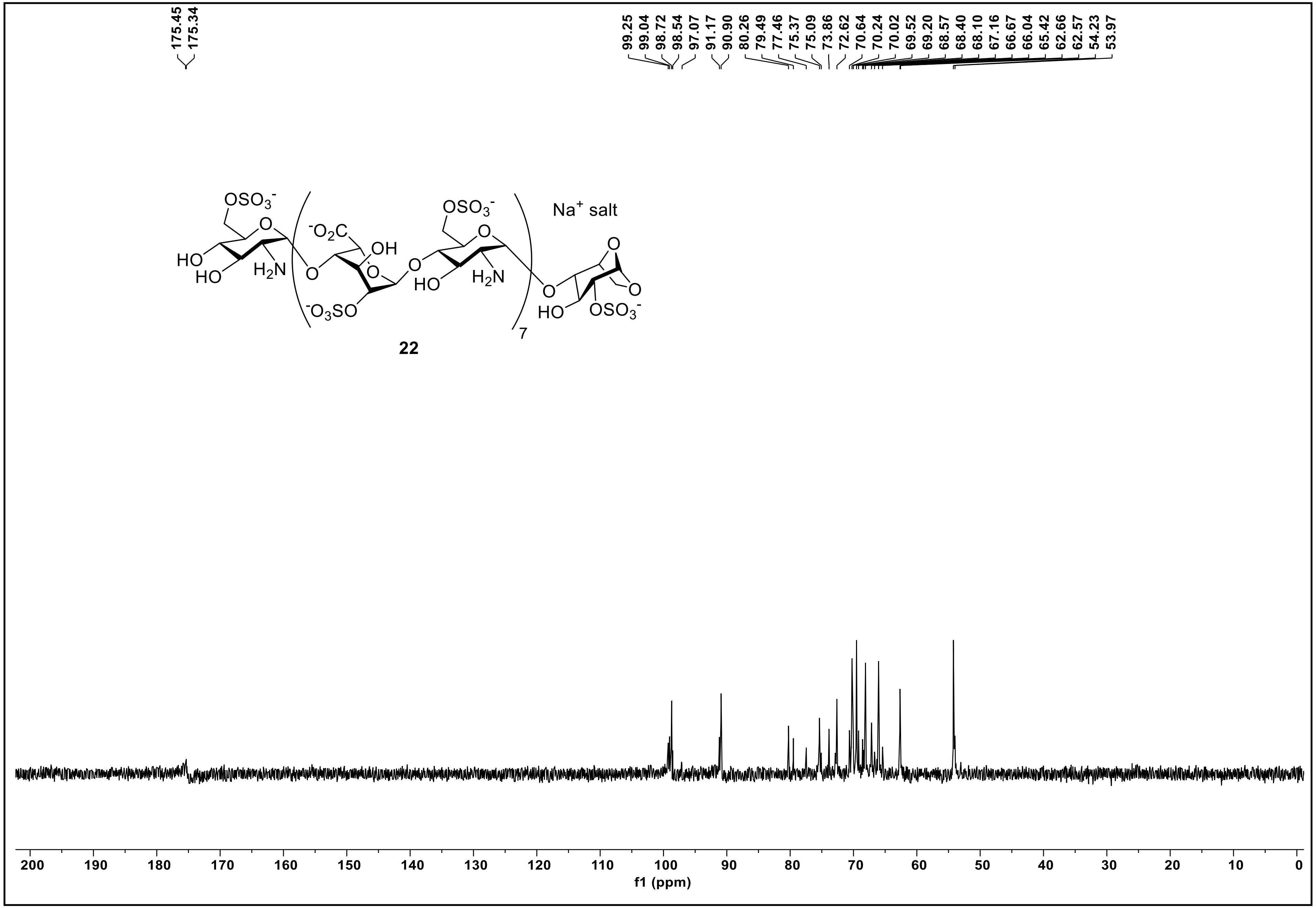
175.45
175.34
99.25
99.04
98.72
98.54
97.07
91.17
90.90
80.26
79.49
77.46
75.37
75.09
73.86
72.62
70.64
70.24
70.02
69.52
69.20
68.57
68.40
68.10
67.16
66.67
66.04
65.42
62.66
62.57
54.23
53.97
OSO3-
-O2C
OH
HO
HO
H2N
O
-O3SO
OSO3-
H2N
7
Na+ salt
HO
OSO3-
22
200 190 180 170 160 150 140 130 120 110 100 90 80 70 60 50 40 30 20 10 0
f1 (ppm)

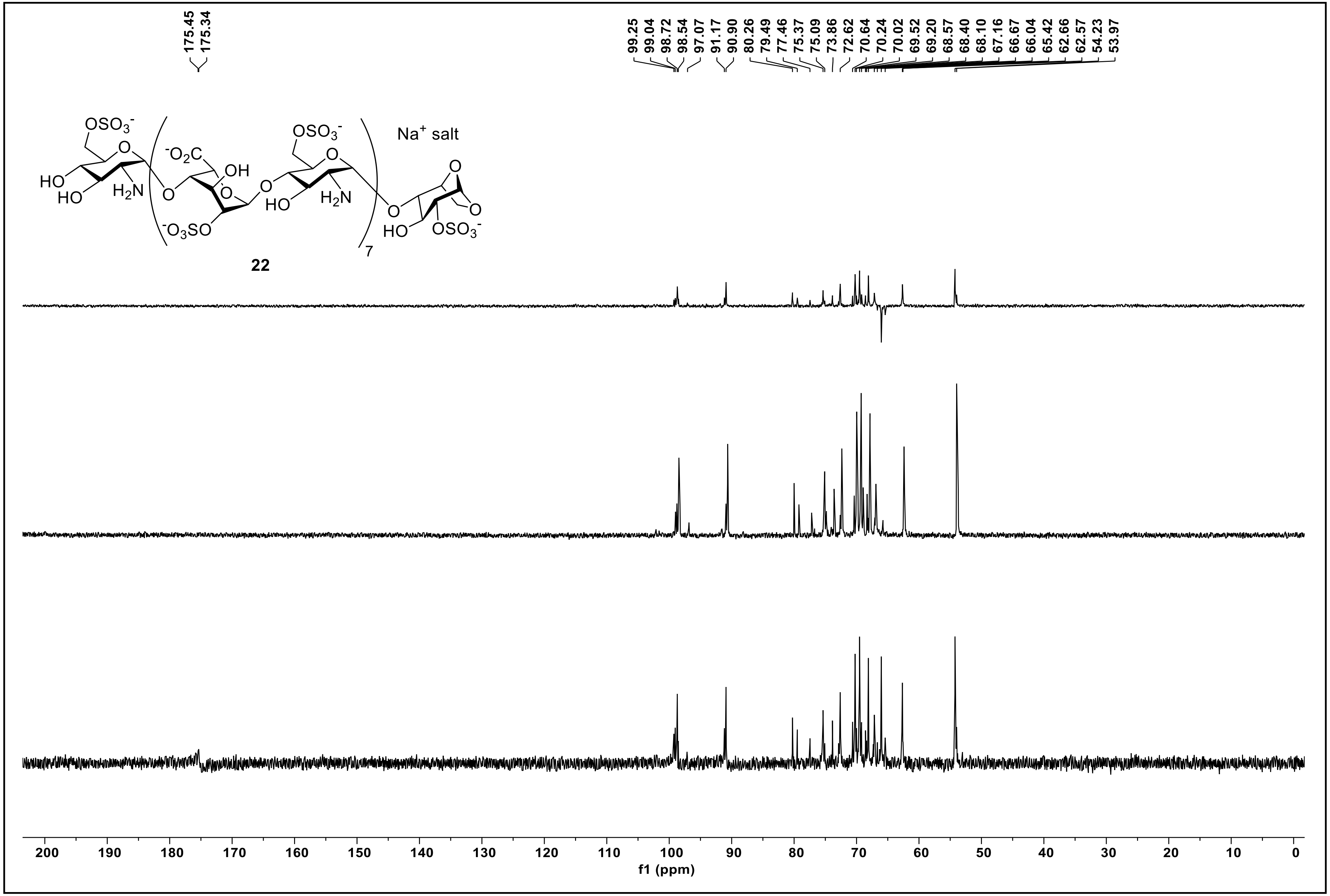

175.45
175.34
99.25
99.04
98.72
98.54
97.07
91.17
90.90
80.26
79.49
77.46
75.37
75.09
73.86
72.62
70.64
70.24
70.02
69.52
69.20
68.57
68.40
68.10
67.16
66.67
66.04
65.42
62.66
62.57
54.23
53.97
Na+ salt
22
200 190 180 170 160 150 140 130 120 110 100 90 80 70 60 50 40 30 20 10 0
f1 (ppm)

HRMS-ESI

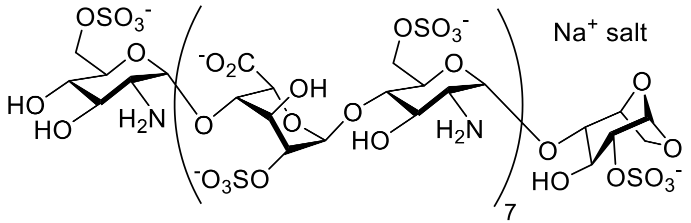

22

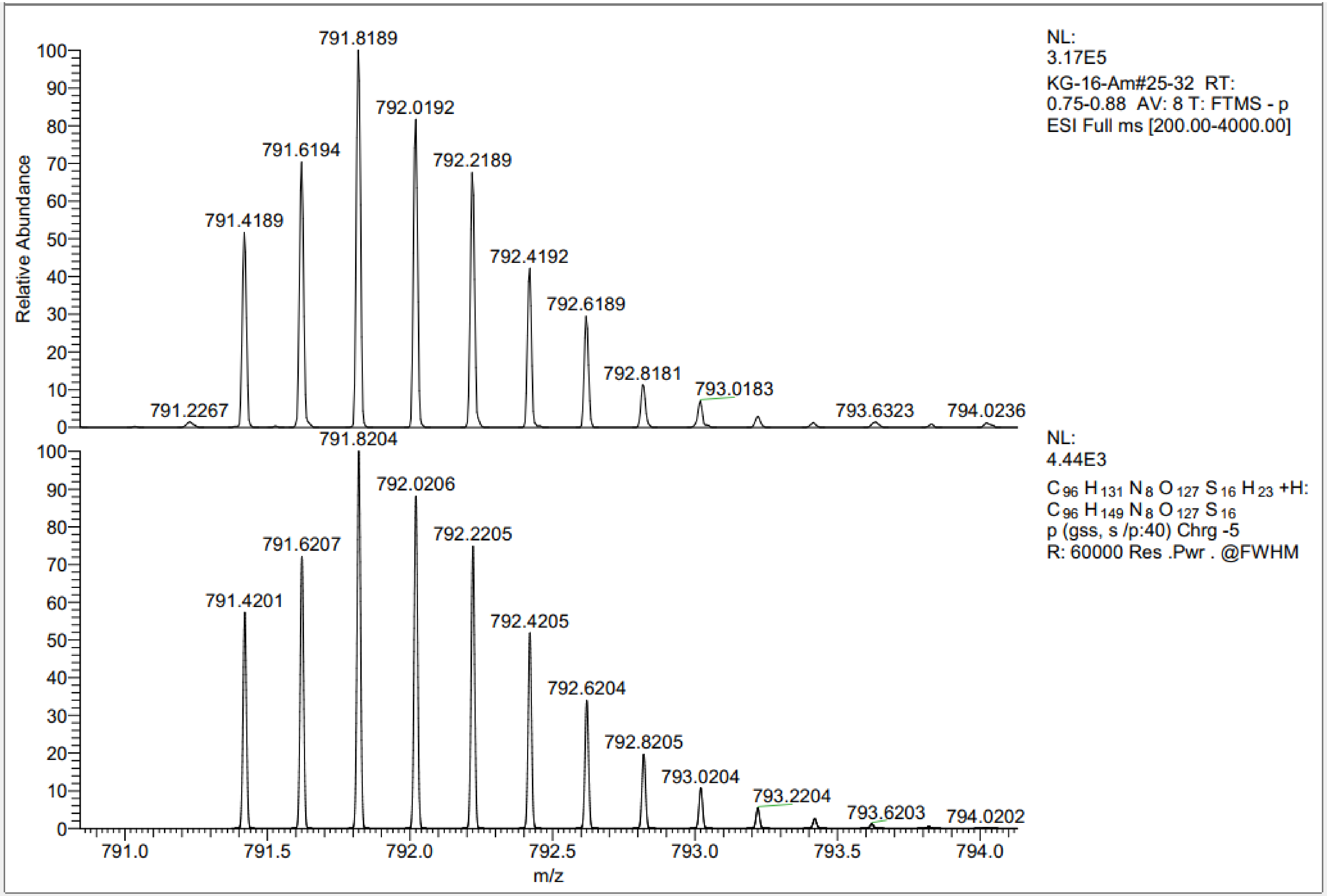

$(M + 18H^+)^{-5}$

Calculated : 791.8204

Found : 791.8189

Mass Error : 1.89 ppm

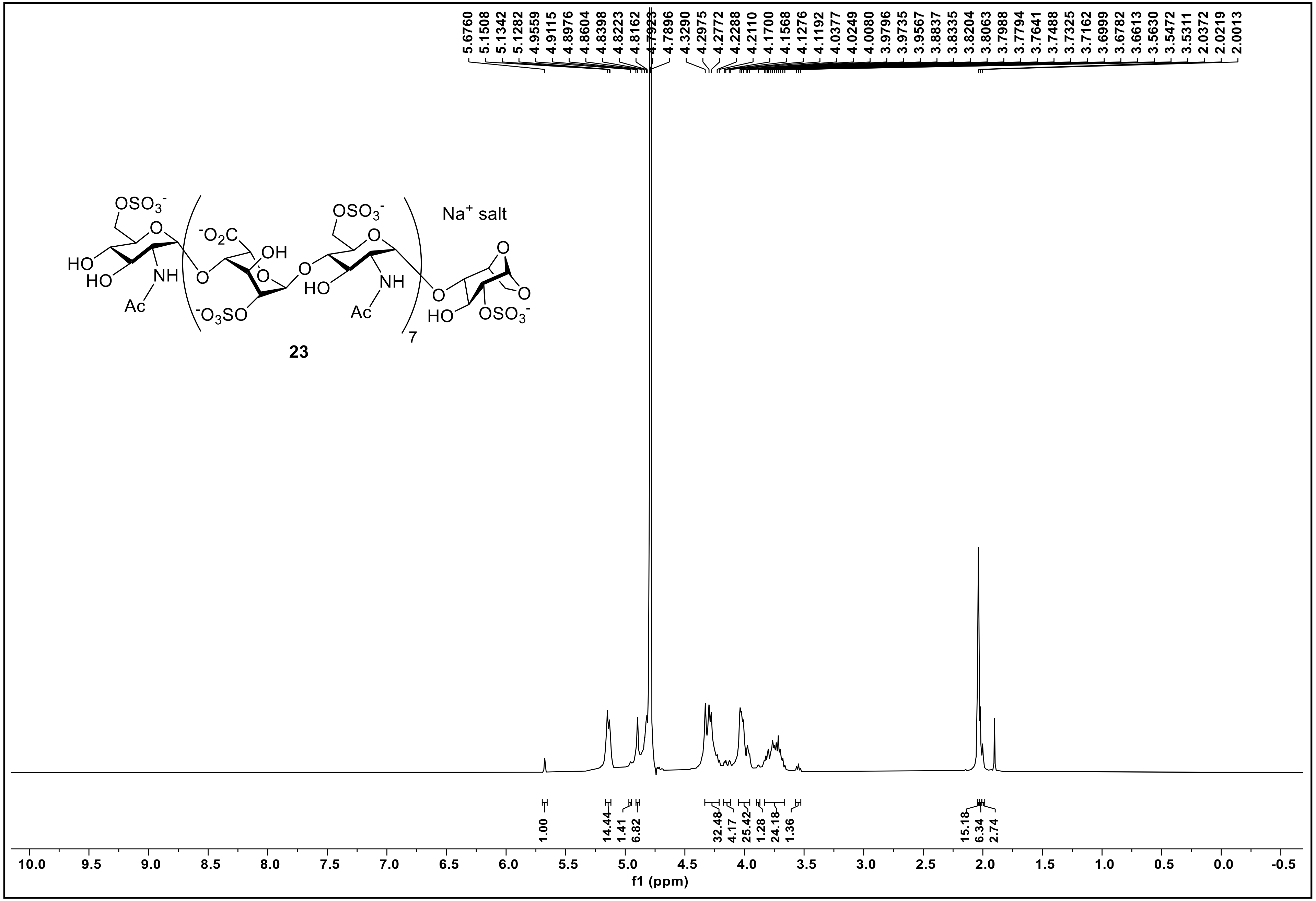
5.6760
5.1508
5.1342
5.1282
4.9559
4.9115
4.8976
4.8604
4.8398
4.8223
4.8162
4.7923
4.7896
4.3290
4.2975
4.2772
4.2288
4.2110
4.1700
4.1568
4.1276
4.1192
4.0377
4.0249
4.0080
3.9796
3.9735
3.9567
3.8837
3.8335
3.8204
3.8063
3.7988
3.7794
3.7641
3.7488
3.7325
3.7162
3.6999
3.6782
3.6613
3.5630
3.5472
3.5311
2.0372
2.0219
2.0013
OSO3-
-O2C
OH
OSO3-
Na+ salt
HO
HO
NH
O
O
O
HO
NH
O
O
O
Ac
-O3SO
Ac
HO
OSO3-
7
23
1.00
14.44
1.41
6.82
32.48
4.17
25.42
1.28
24.18
1.36
15.18
6.34
2.74
10.0 9.5 9.0 8.5 8.0 7.5 7.0 6.5 6.0 5.5 5.0 4.5 4.0 3.5 3.0 2.5 2.0 1.5 1.0 0.5 0.0 -0.5
f1 (ppm)

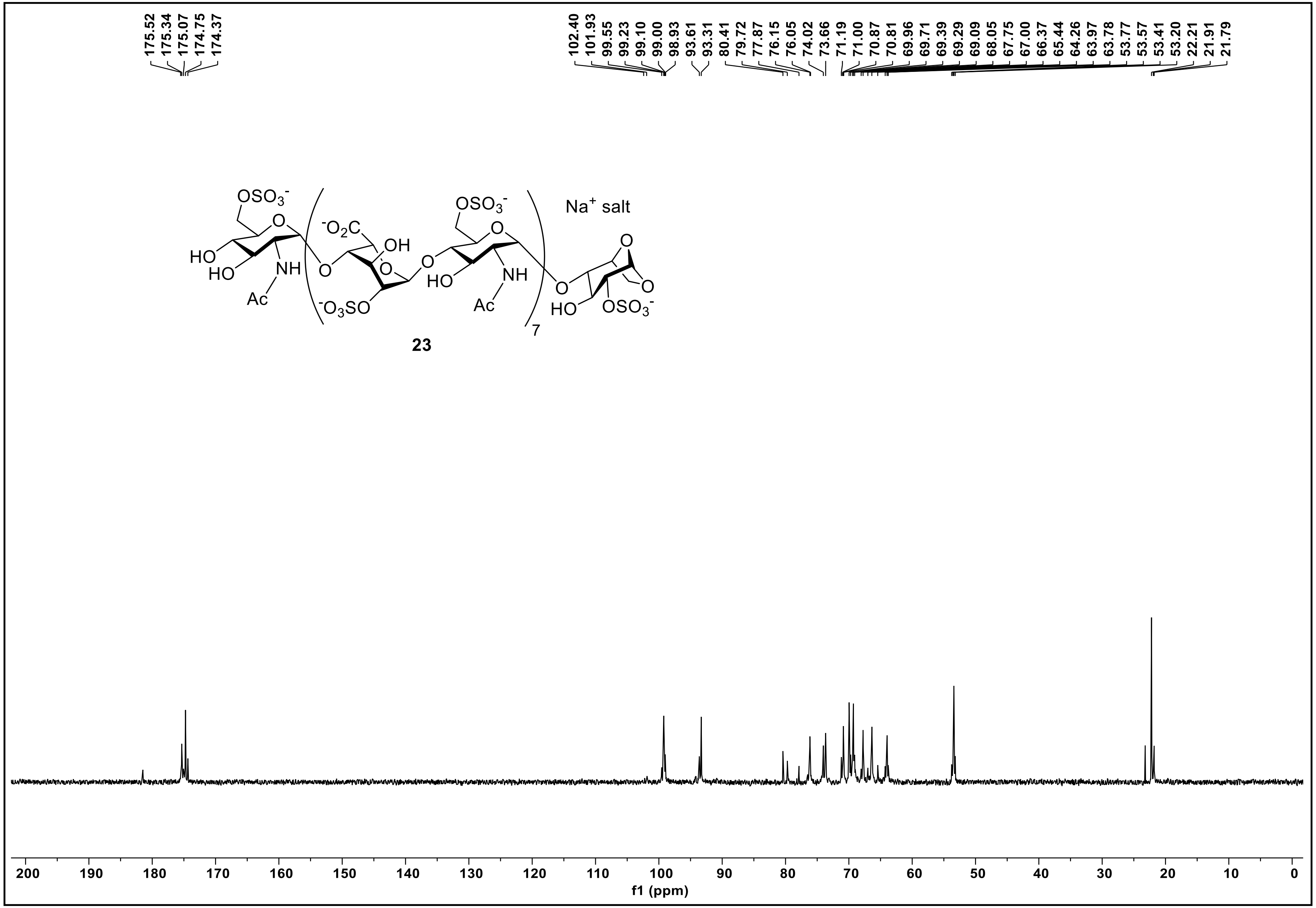
175.52
175.34
175.07
174.75
174.37
102.40
101.93
99.55
99.23
99.10
99.00
98.93
93.61
93.31
80.41
79.72
77.87
76.15
76.05
74.02
73.66
71.19
71.00
70.87
70.81
69.96
69.71
69.39
69.29
69.09
68.05
67.75
67.00
66.37
65.44
64.26
63.97
63.78
53.77
53.57
53.41
53.20
22.21
21.91
21.79
Na+ salt
23
f1 (ppm)

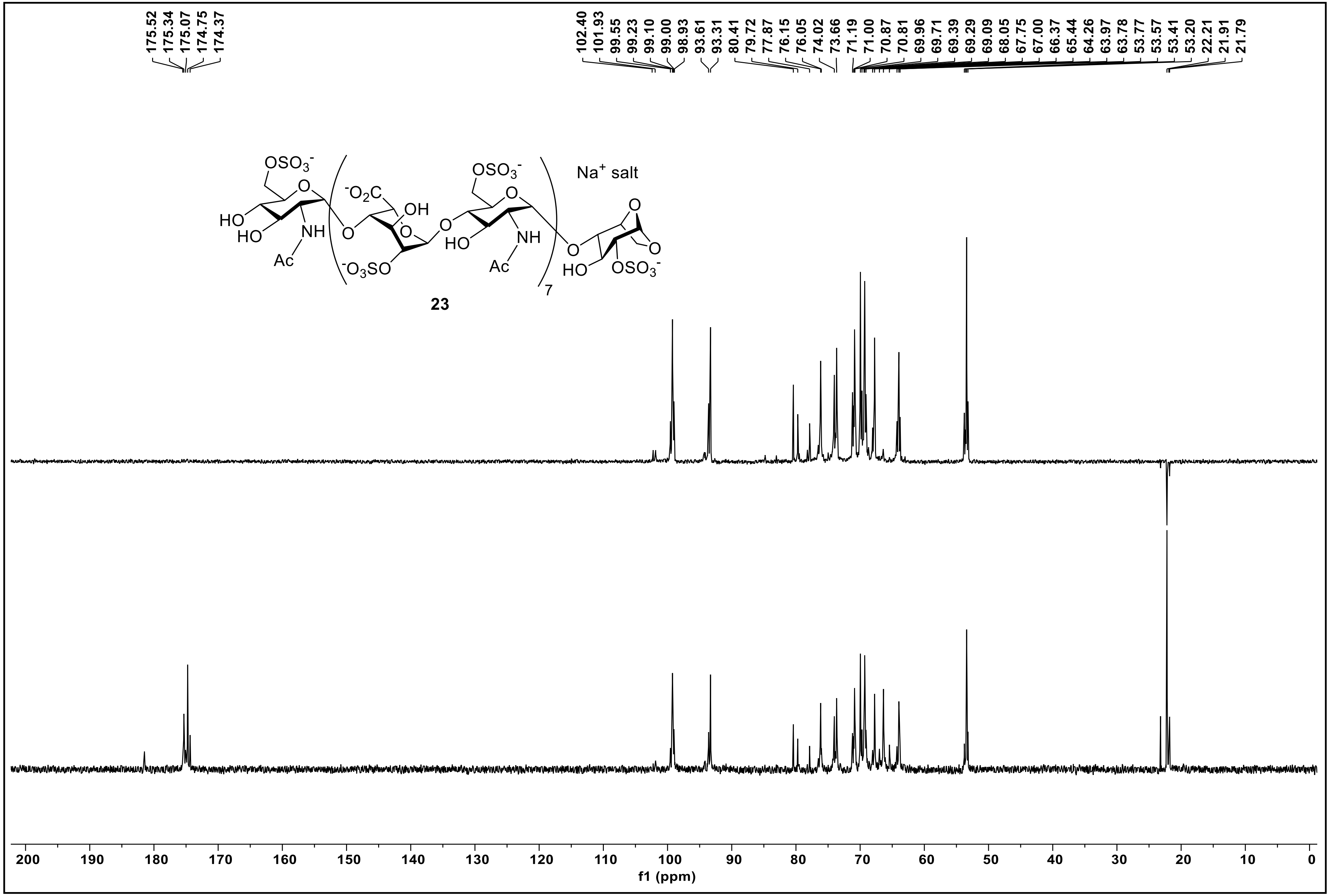
175.52
175.34
175.07
174.75
174.37
102.40
101.93
99.55
99.23
99.10
99.00
98.93
93.61
93.31
80.41
79.72
77.87
76.15
76.05
74.02
73.66
71.19
71.00
70.87
70.81
69.96
69.71
69.39
69.29
69.09
68.05
67.75
67.00
66.37
65.44
64.26
63.97
63.78
53.77
53.57
53.41
53.20
22.21
21.91
21.79
Na+ salt
23
f1 (ppm)

HRMS-ESI

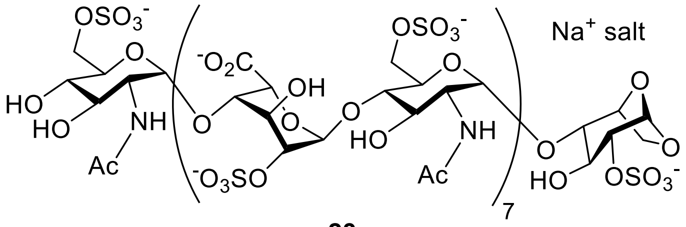

23

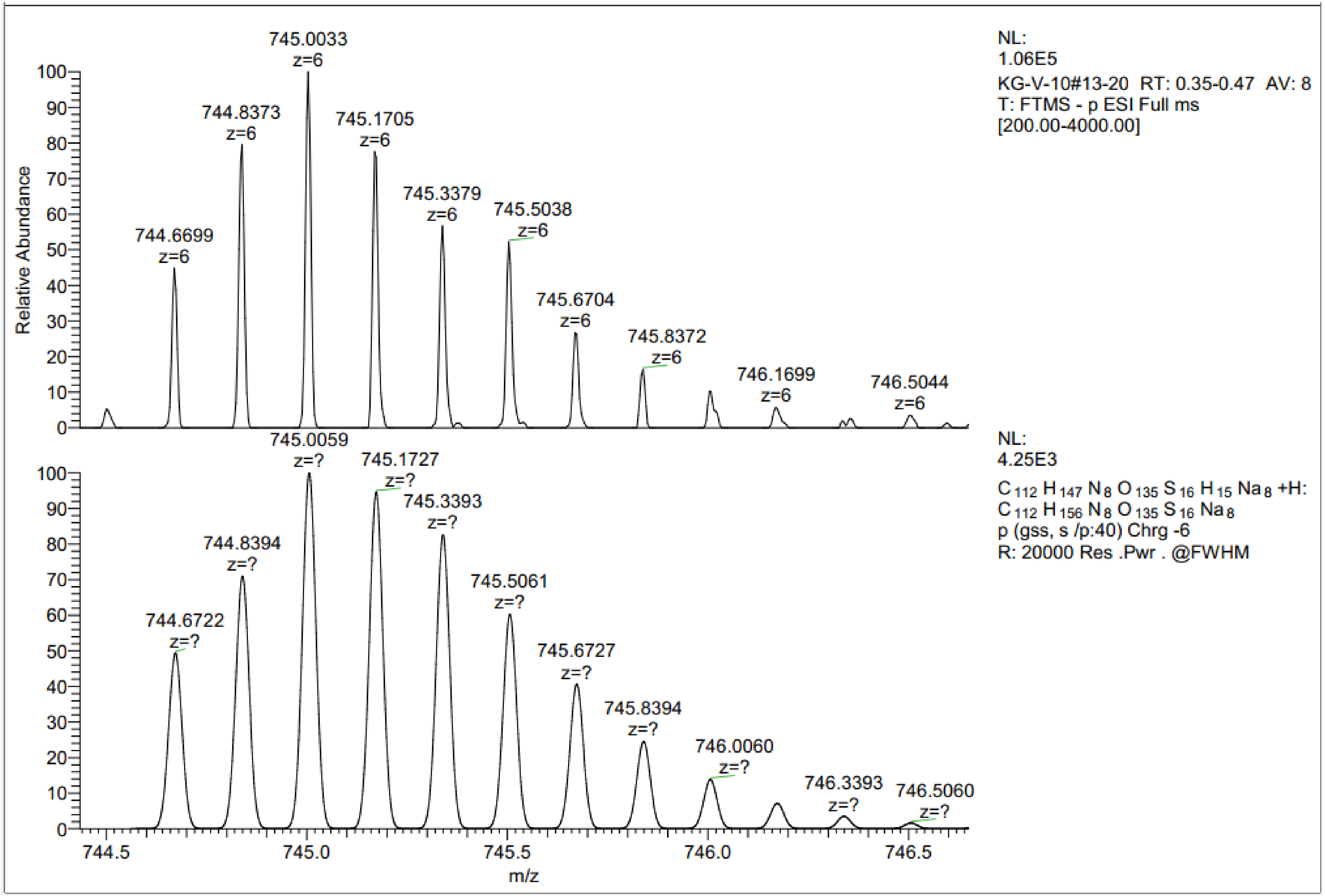

$(M + 9H^+ + 8Na^+)^{-6}$

Calculated : 745.0059

Found : 745.0033

Mass Error : 3.49 ppm

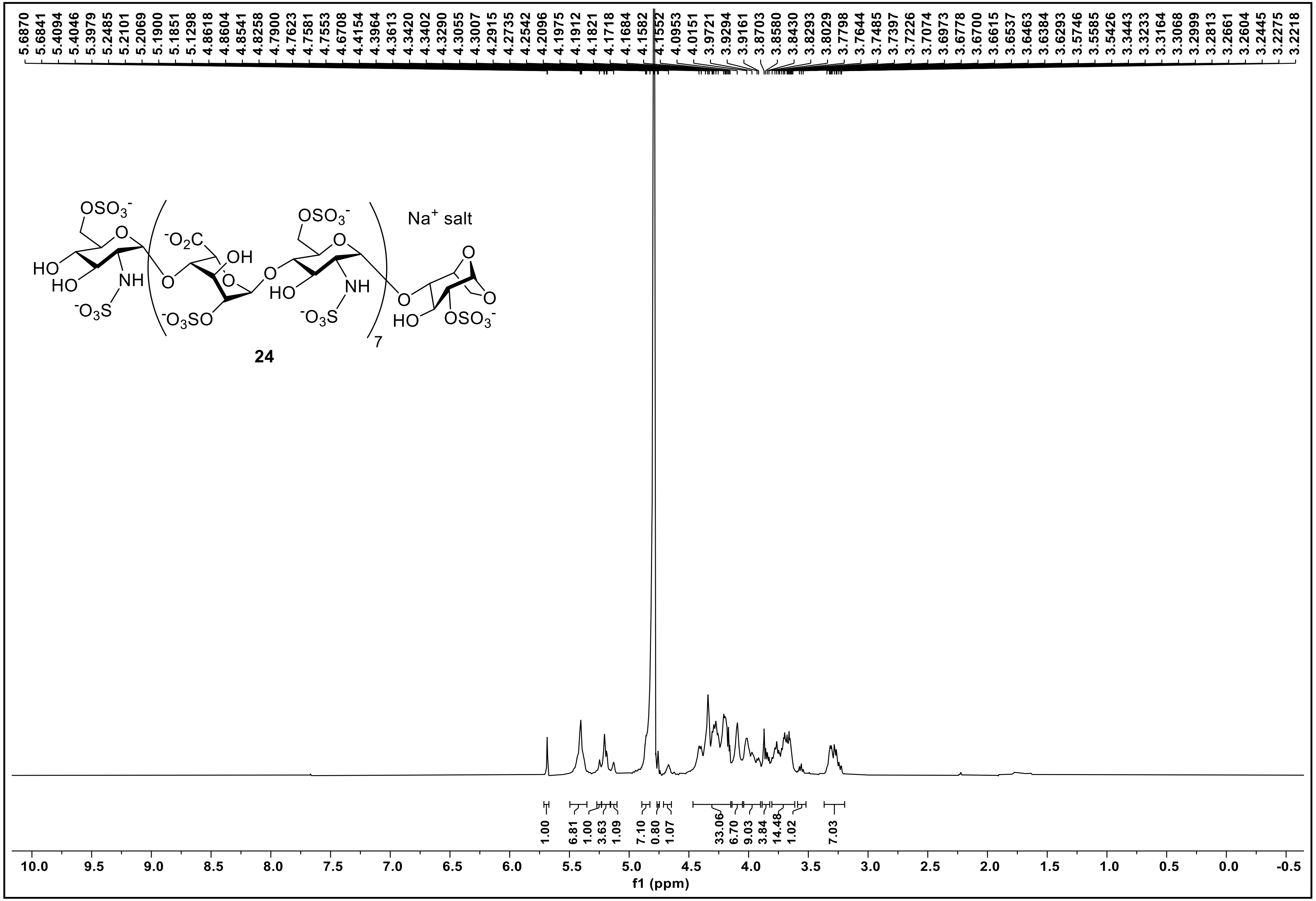
5.6870
5.6841
5.4094
5.4046
5.3979
5.2485
5.2101
5.2069
5.1900
5.1851
5.1298
4.8618
4.8604
4.8541
4.8258
4.7900
4.7623
4.7581
4.7553
4.6708
4.4154
4.3964
4.3613
4.3420
4.3402
4.3290
4.3055
4.3007
4.2915
4.2735
4.2542
4.2096
4.1975
4.1912
4.1821
4.1718
4.1684
4.1582
4.1552
4.0953
4.0151
3.9721
3.9294
3.9161
3.8703
3.8580
3.8430
3.8293
3.8029
3.7798
3.7644
3.7485
3.7397
3.7226
3.7074
3.6973
3.6778
3.6700
3.6615
3.6537
3.6463
3.6384
3.6293
3.5746
3.5585
3.5426
3.3443
3.3233
3.3164
3.3068
3.2999
3.2813
3.2661
3.2604
3.2445
3.2275
3.2218
Na+ salt
24
1.00
6.81
1.00
3.63
1.09
7.10
0.80
1.07
33.06
6.70
9.03
3.84
14.48
1.02
7.03
10.0 9.5 9.0 8.5 8.0 7.5 7.0 6.5 6.0 5.5 5.0 4.5 4.0 3.5 3.0 2.5 2.0 1.5 1.0 0.5 0.0 -0.5
f1 (ppm)

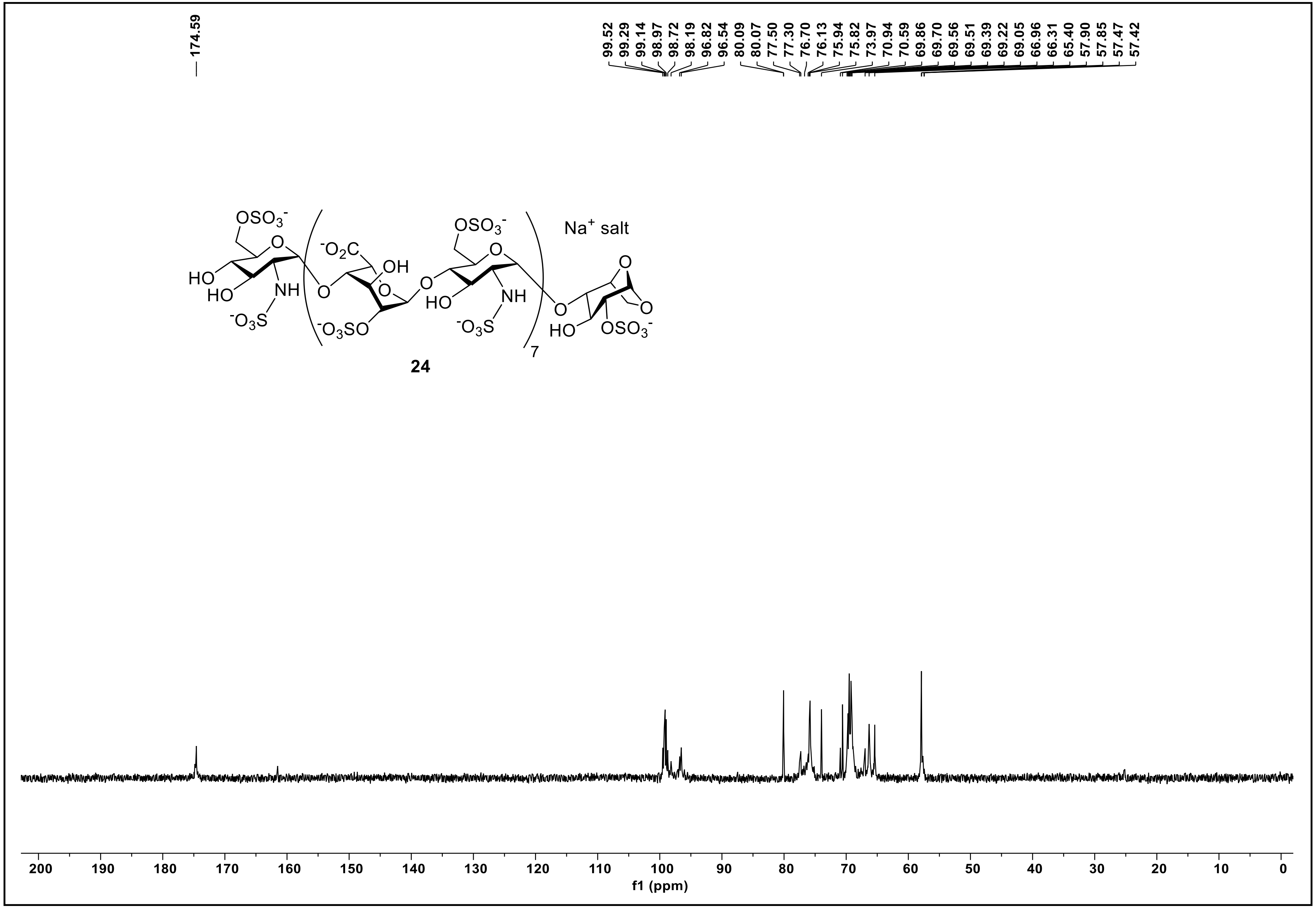
174.59
99.52
99.29
99.14
98.97
98.72
98.19
96.82
96.54
80.09
80.07
77.50
77.30
76.70
76.13
75.94
75.82
73.97
70.94
70.59
69.86
69.70
69.56
69.51
69.39
69.22
69.05
66.96
66.31
65.40
57.90
57.85
57.47
57.42
Na+ salt
24
f1 (ppm)

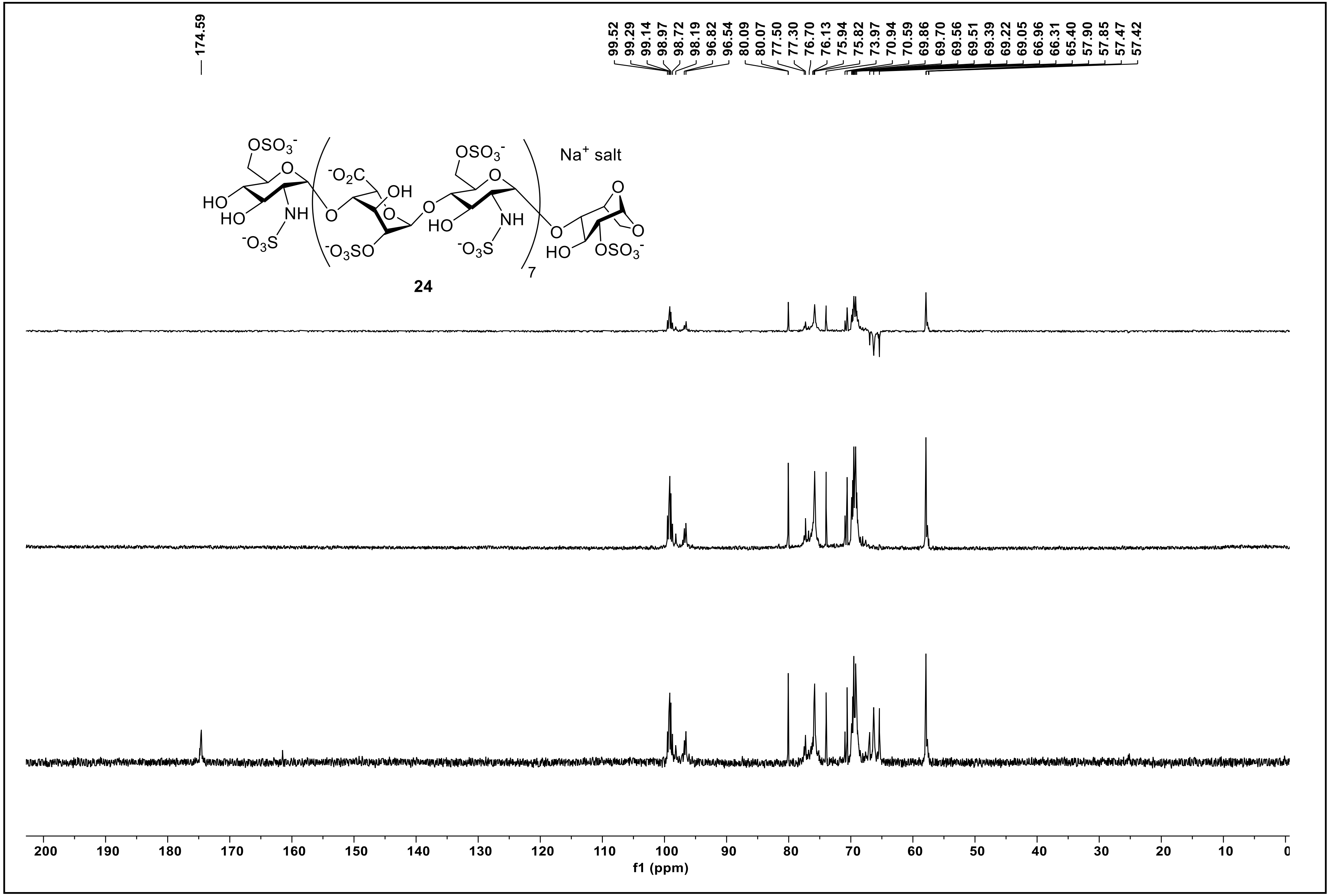
174.59
99.52
99.29
99.14
98.97
98.72
98.19
96.82
96.54
80.09
80.07
77.50
77.30
76.70
76.13
75.94
75.82
73.97
70.94
70.59
69.86
69.70
69.56
69.51
69.39
69.22
69.05
66.96
66.31
65.40
57.90
57.85
57.47
57.42
Na+ salt
OSO3-
-O2C
OH
HO
NH
O
-O3S
-O3SO
HO
OSO3-
7
24
200 190 180 170 160 150 140 130 120 110 100 90 80 70 60 50 40 30 20 10 0
f1 (ppm)

HRMS-ESI

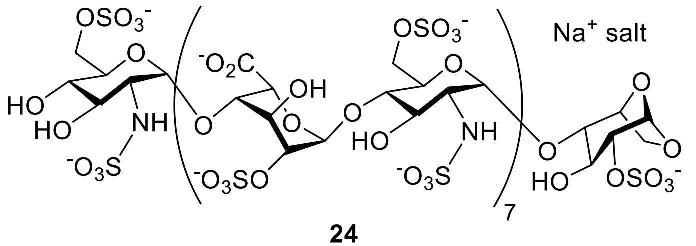

24

$(M + 12H^{+} + 13Na^{+})^{-6}$

Calculated : 813.9190

Found : 813.9227

Mass Error : 4.55 ppm

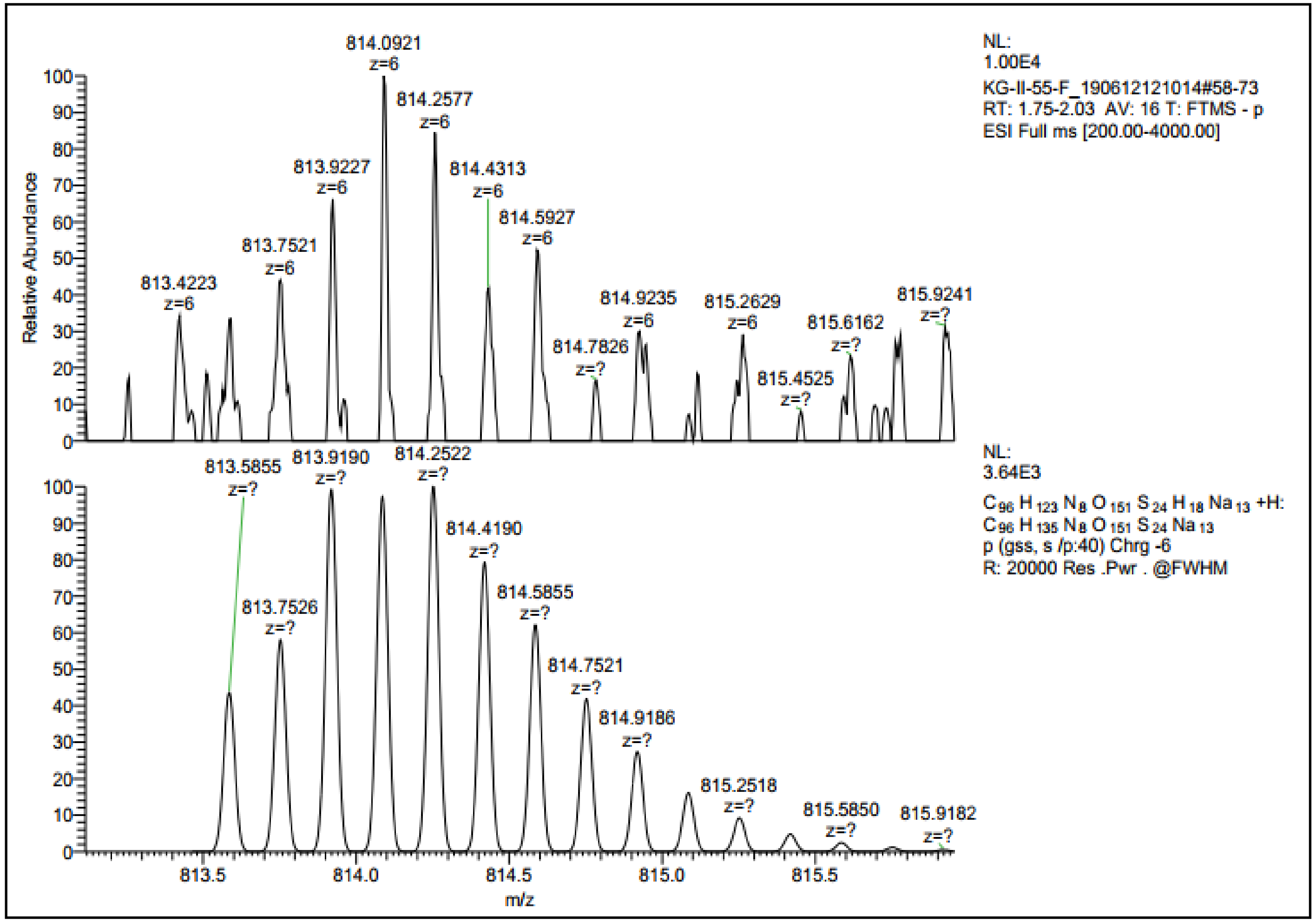

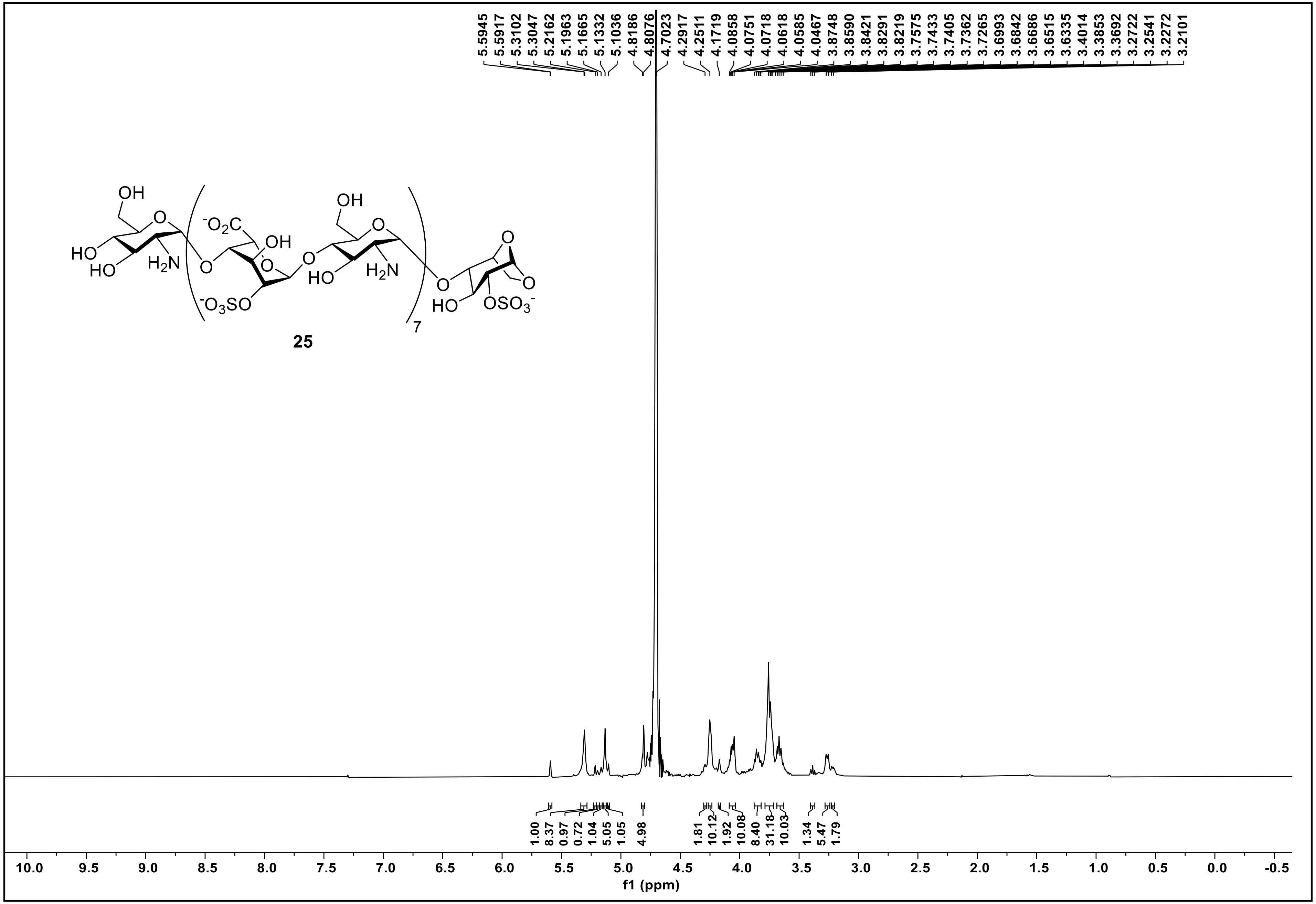
5.5945
5.5917
5.3102
5.3047
5.2162
5.1963
5.1665
5.1332
5.1036
4.8186
4.8076
4.7023
4.2917
4.2511
4.1719
4.0858
4.0751
4.0718
4.0618
4.0585
4.0467
3.8748
3.8590
3.8421
3.8291
3.8219
3.7575
3.7433
3.7405
3.7362
3.7265
3.6993
3.6842
3.6686
3.6515
3.6335
3.4014
3.3853
3.3692
3.2722
3.2541
3.2272
3.2101
25
1.00
8.37
0.97
0.72
1.04
5.05
1.05
4.98
1.81
10.12
1.92
10.08
8.40
31.18
10.03
1.34
5.47
1.79
f1 (ppm)

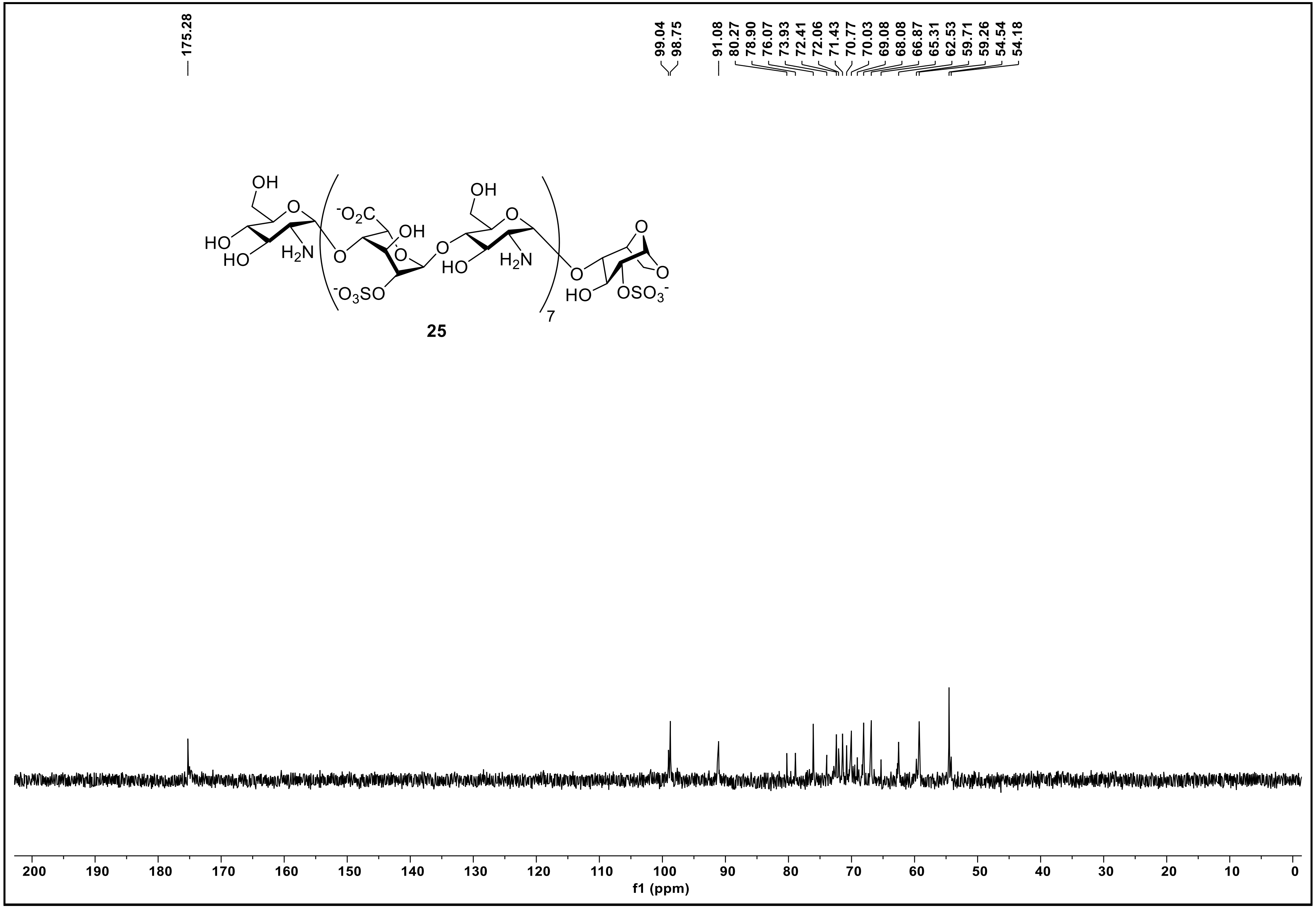
175.28
99.04
98.75
91.08
80.27
78.90
76.07
73.93
72.41
72.06
71.43
70.77
70.03
69.08
68.08
66.87
65.31
62.53
59.71
59.26
54.54
54.18
25
f1 (ppm)

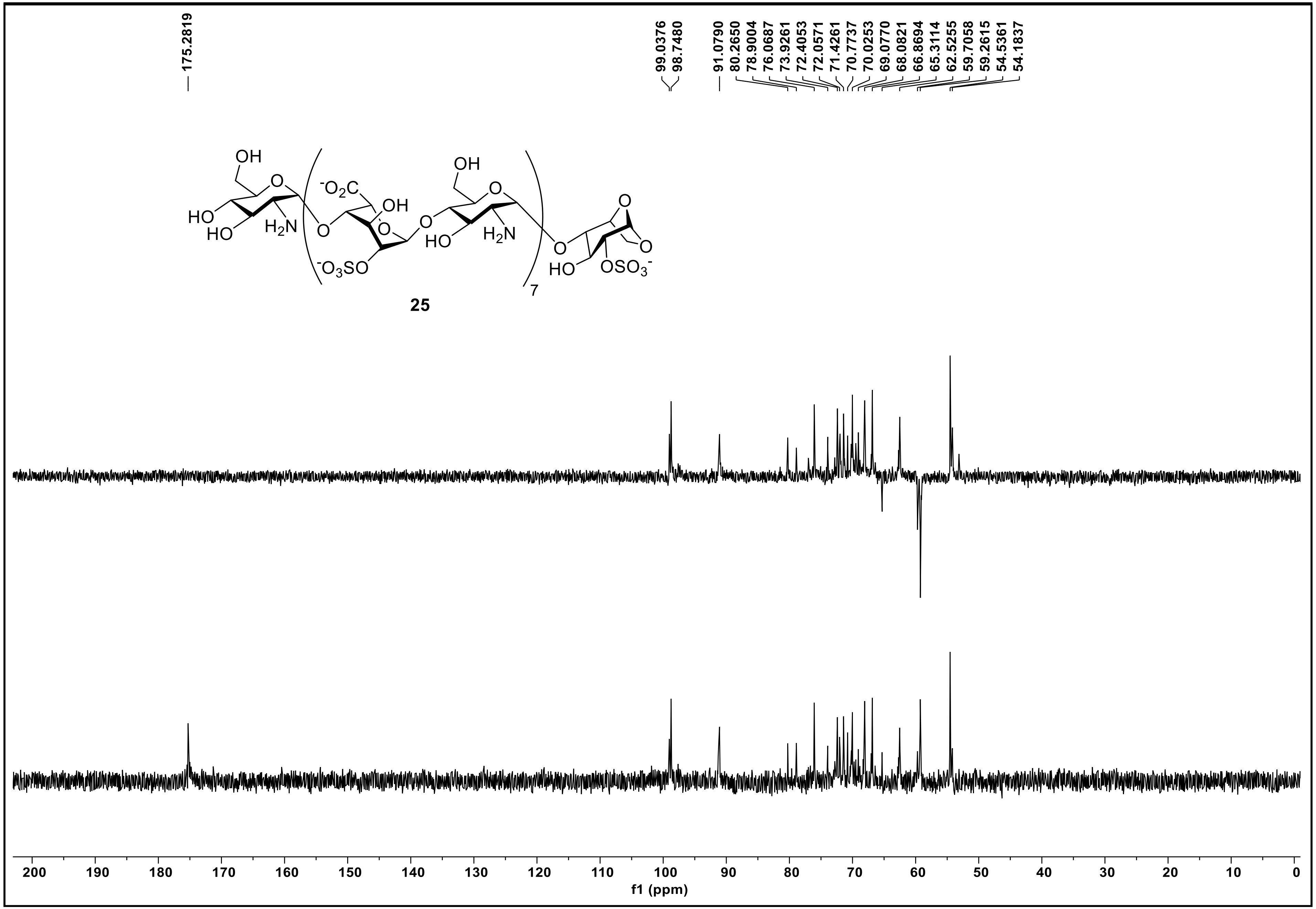
175.2819
99.0376
98.7480
91.0790
80.2650
78.9004
76.0687
73.9261
72.4053
72.0571
71.4261
70.7737
70.0253
69.0770
68.0821
66.8694
65.3114
62.5255
59.7058
59.2615
54.5361
54.1837
25
200 190 180 170 160 150 140 130 120 110 100 90 80 70 60 50 40 30 20 10 0
f1 (ppm)

HRMS-ESI

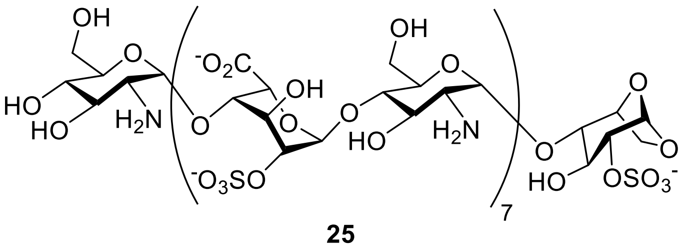

25

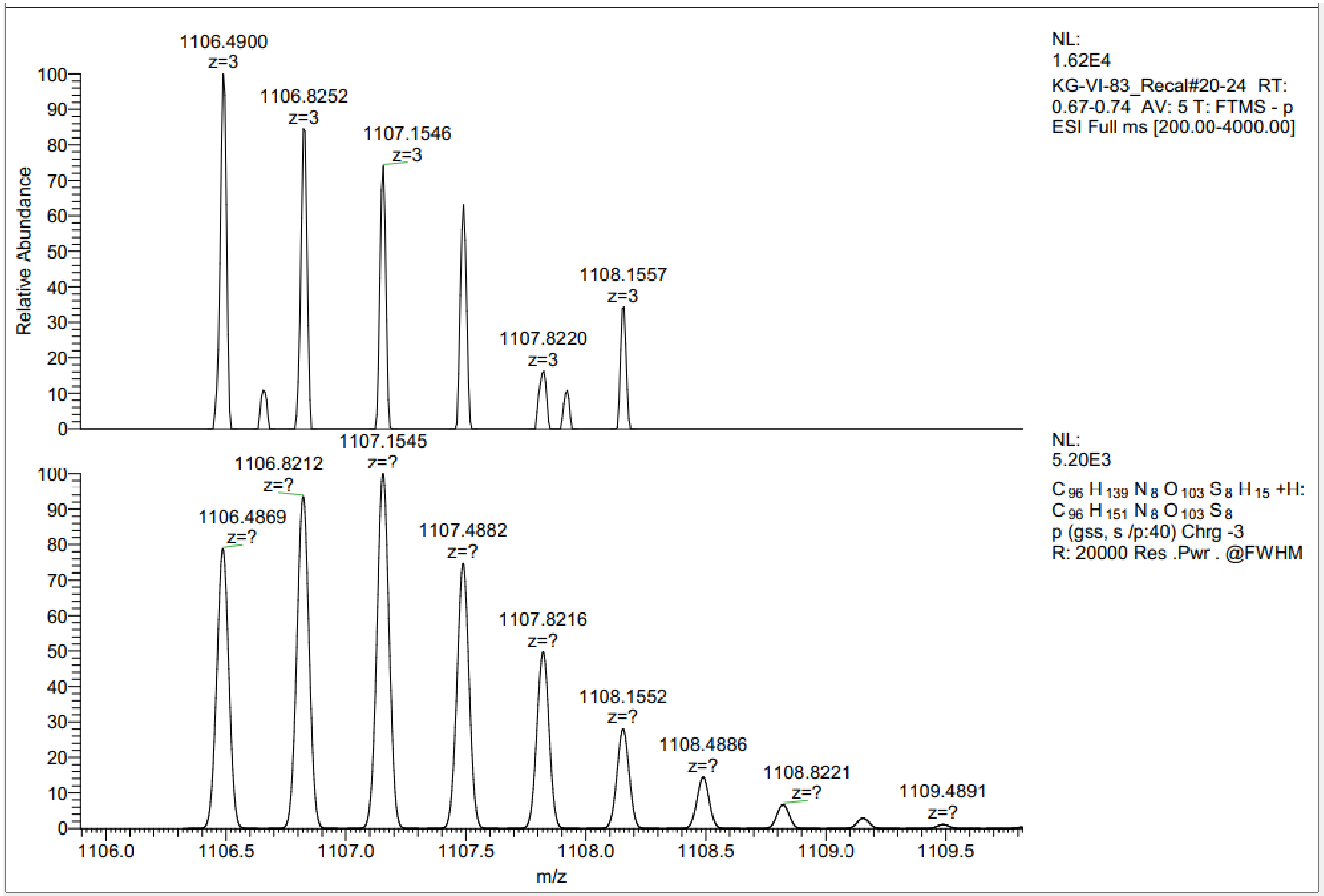

$(M + 12H^+)^{-3}$

Calculated : 1106.8212

Found : 1106.8252

Mass Error : 3.61 ppm

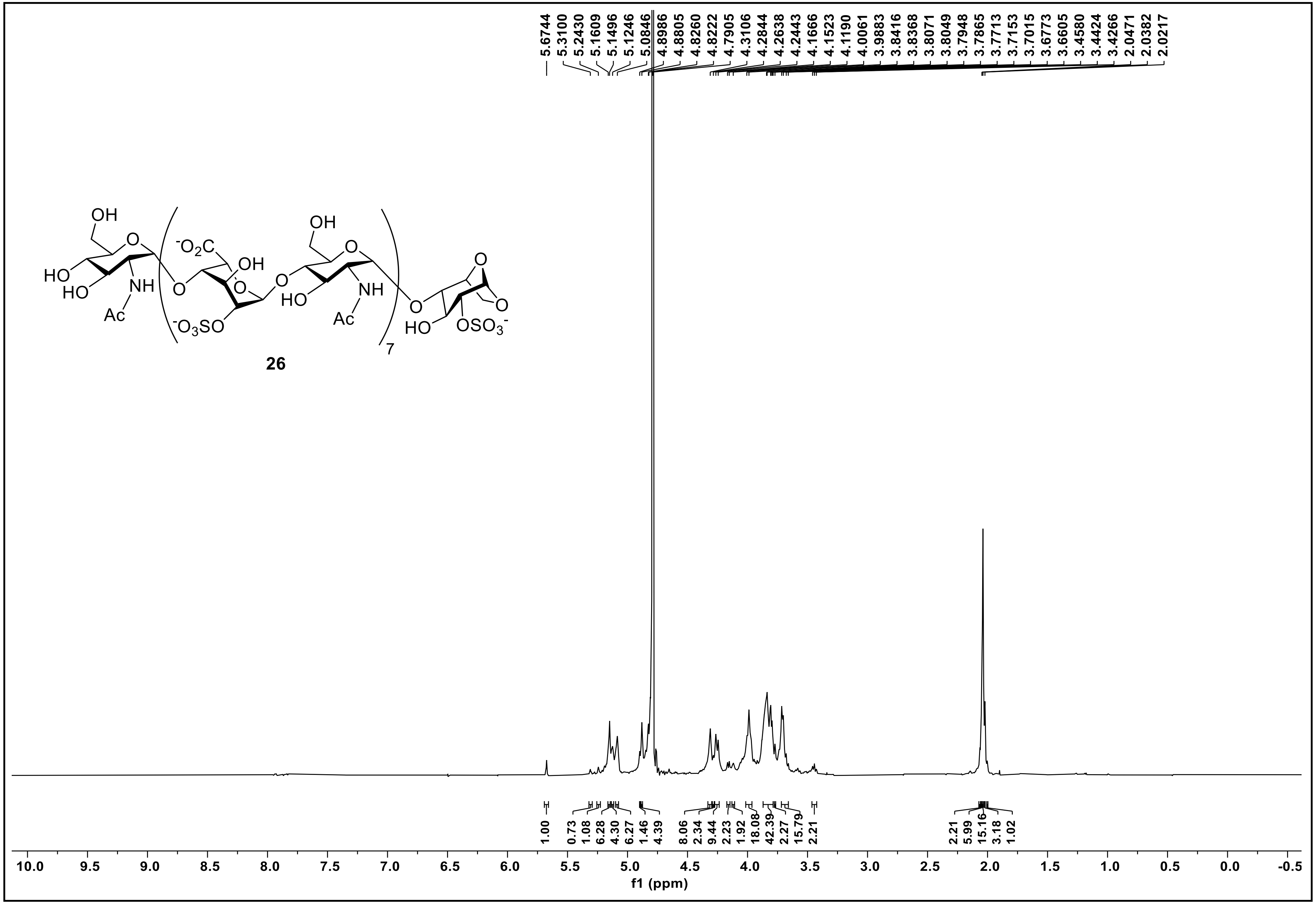
5.6744
5.3100
5.2430
5.1609
5.1496
5.1246
5.0846
4.8986
4.8805
4.8260
4.8222
4.7905
4.3106
4.2844
4.2638
4.2443
4.1666
4.1523
4.1190
4.0061
3.9883
3.8416
3.8368
3.8071
3.8049
3.7948
3.7865
3.7713
3.7153
3.7015
3.6773
3.6605
3.4580
3.4424
3.4266
2.0471
2.0382
2.0217
OH
O
HO
HO
NH
Ac
O
-O2C
OH
O
-O3SO
O
OH
O
HO
NH
Ac
7
O
O
O
HO
OSO3-
26
1.00
0.73
1.08
6.28
4.30
6.27
1.46
4.39
8.06
2.34
9.44
2.23
1.92
18.08
42.39
2.27
15.79
2.21
2.21
5.99
15.16
3.18
1.02
10.0 9.5 9.0 8.5 8.0 7.5 7.0 6.5 6.0 5.5 5.0 4.5 4.0 3.5 3.0 2.5 2.0 1.5 1.0 0.5 0.0 -0.5
f1 (ppm)

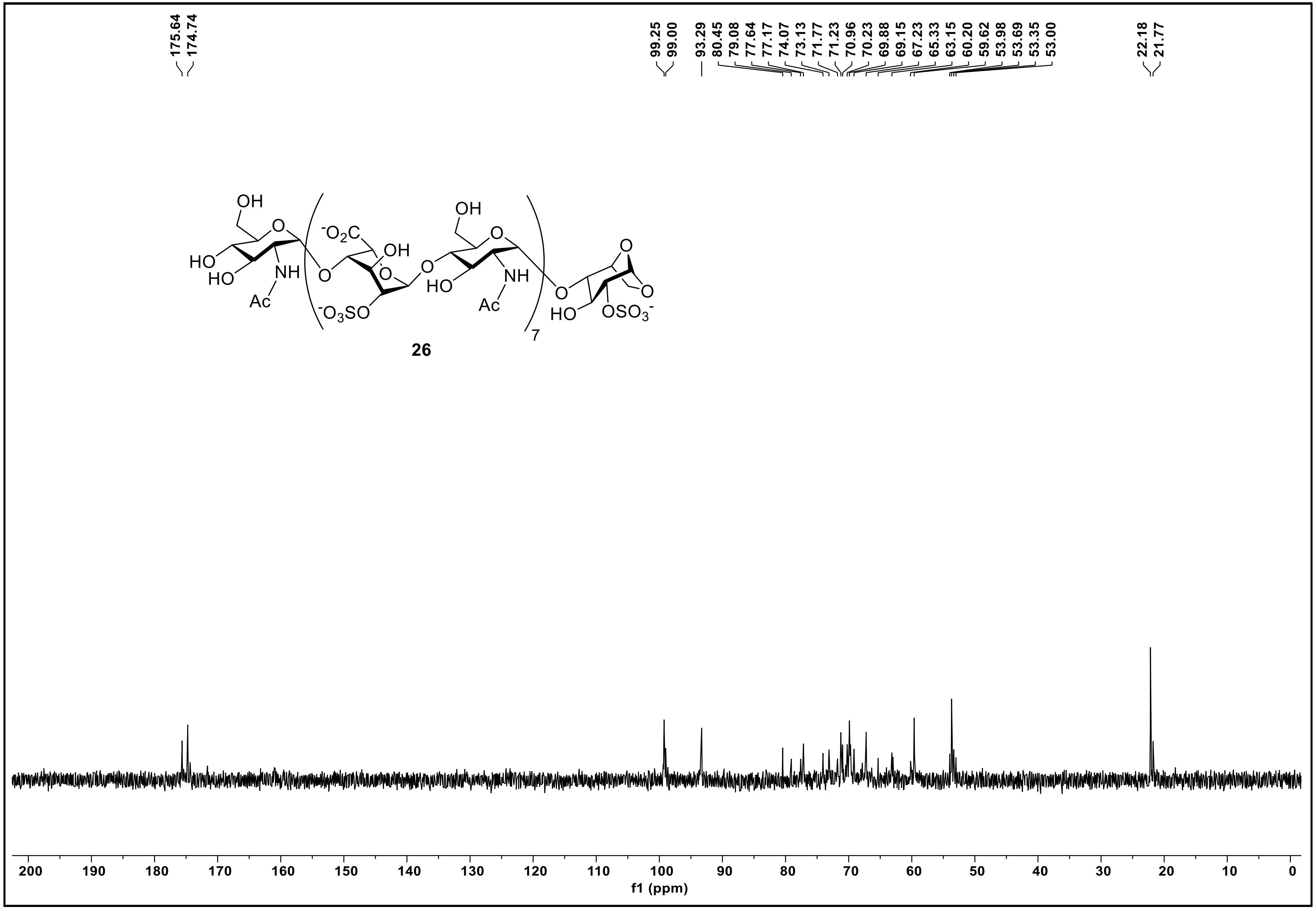
175.64
174.74
99.25
99.00
93.29
80.45
79.08
77.64
77.17
74.07
73.13
71.77
71.23
70.96
70.23
69.88
69.15
67.23
65.33
63.15
60.20
59.62
53.98
53.69
53.35
53.00
22.18
21.77
26
f1 (ppm)

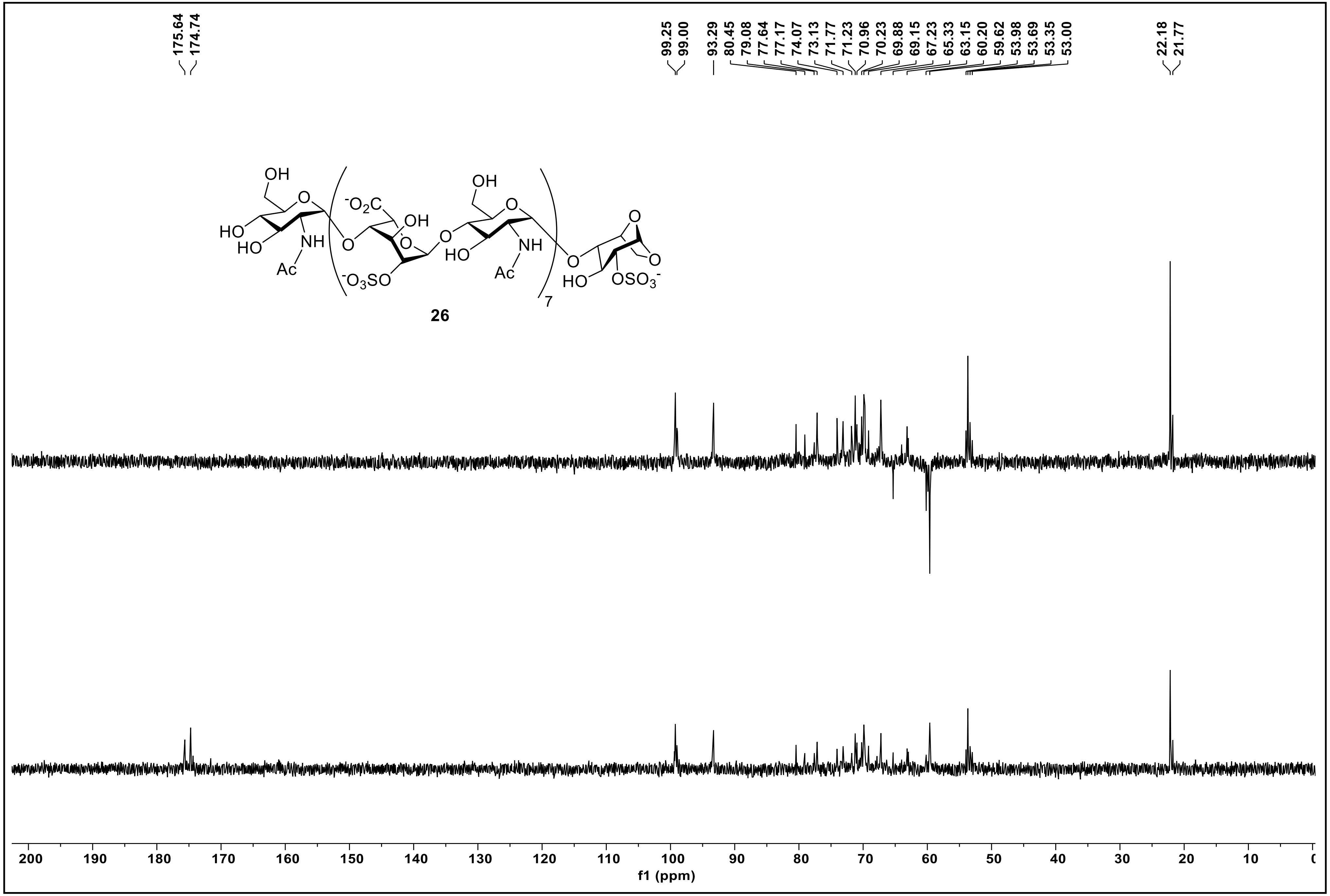
175.64
174.74
99.25
99.00
93.29
80.45
79.08
77.64
77.17
74.07
73.13
71.77
71.23
70.96
70.23
69.88
69.15
67.23
65.33
63.15
60.20
59.62
53.98
53.69
53.35
53.00
22.18
21.77
26
f1 (ppm)

HRMS-ESI

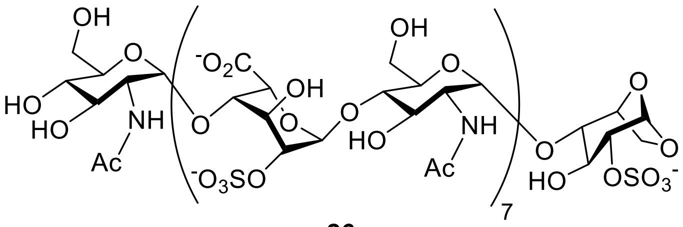

26

$(M + 10H^+)^{-5}$

Calculated : 730.9067

Found : 730.9020

Mass Error : 6.43 ppm

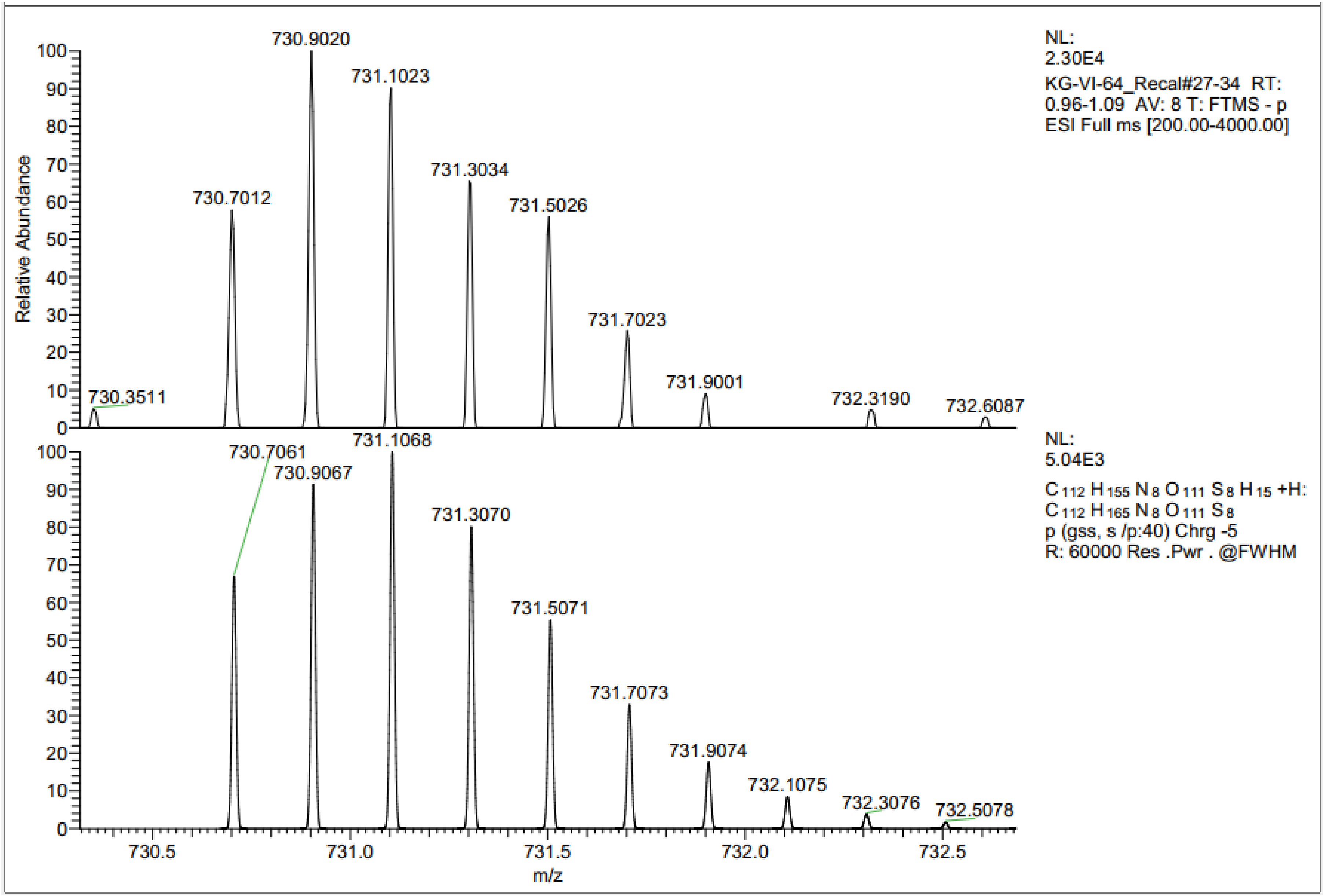

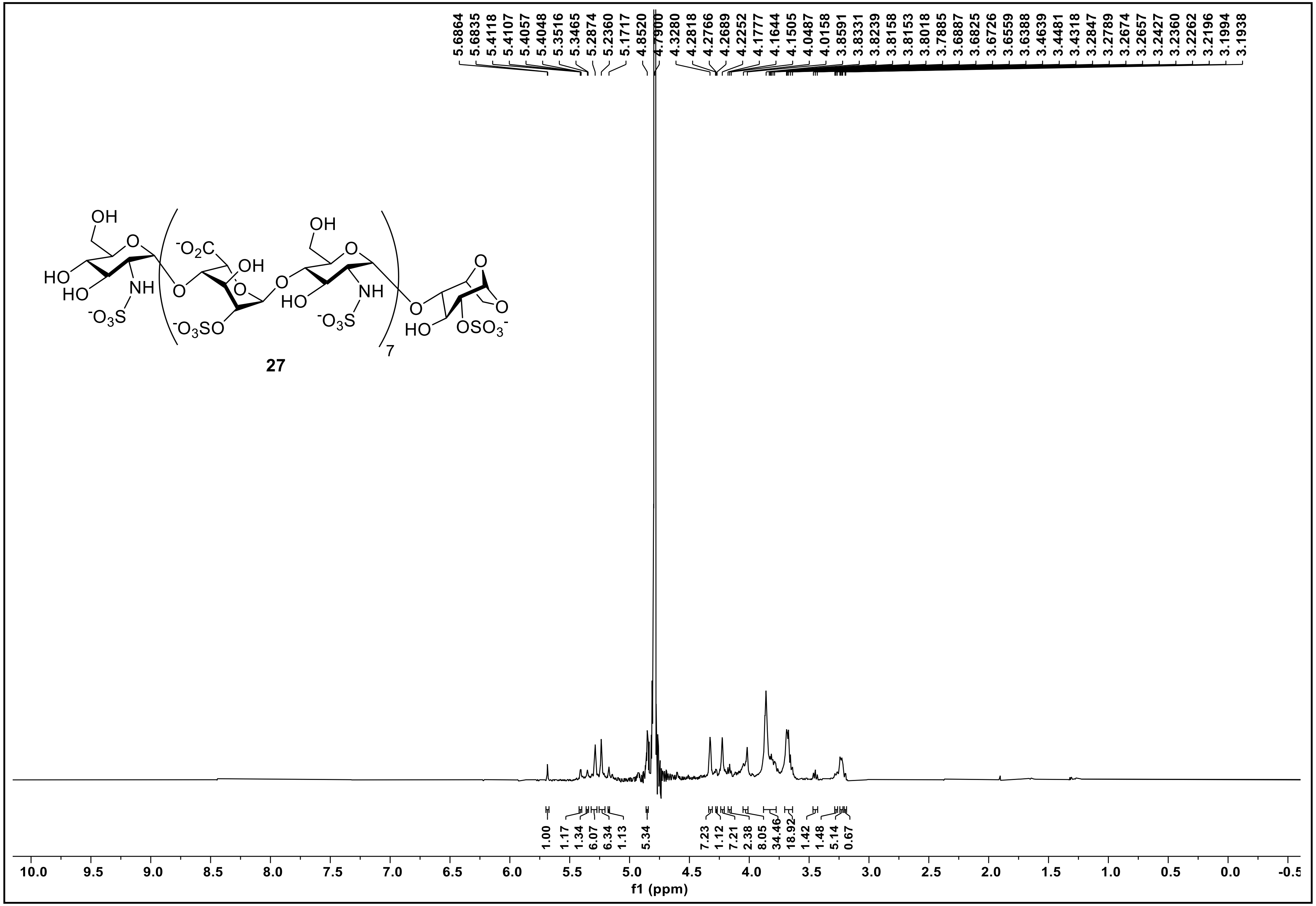
5.6864
5.6835
5.4118
5.4107
5.4057
5.4048
5.3516
5.3465
5.2874
5.2360
5.1717
4.8520
4.7900
4.3280
4.2818
4.2766
4.2689
4.2252
4.1777
4.1644
4.1505
4.0487
4.0158
3.8591
3.8331
3.8239
3.8158
3.8153
3.8018
3.7885
3.6887
3.6825
3.6726
3.6559
3.6388
3.4639
3.4481
3.4318
3.2847
3.2789
3.2674
3.2657
3.2427
3.2360
3.2262
3.2196
3.1994
3.1938
27
1.00
1.17
1.34
6.07
6.34
1.13
5.34
7.23
1.12
7.21
2.38
8.05
34.46
18.92
1.42
1.48
5.14
0.67
10.0 9.5 9.0 8.5 8.0 7.5 7.0 6.5 6.0 5.5 5.0 4.5 4.0 3.5 3.0 2.5 2.0 1.5 1.0 0.5 0.0 -0.5
f1 (ppm)

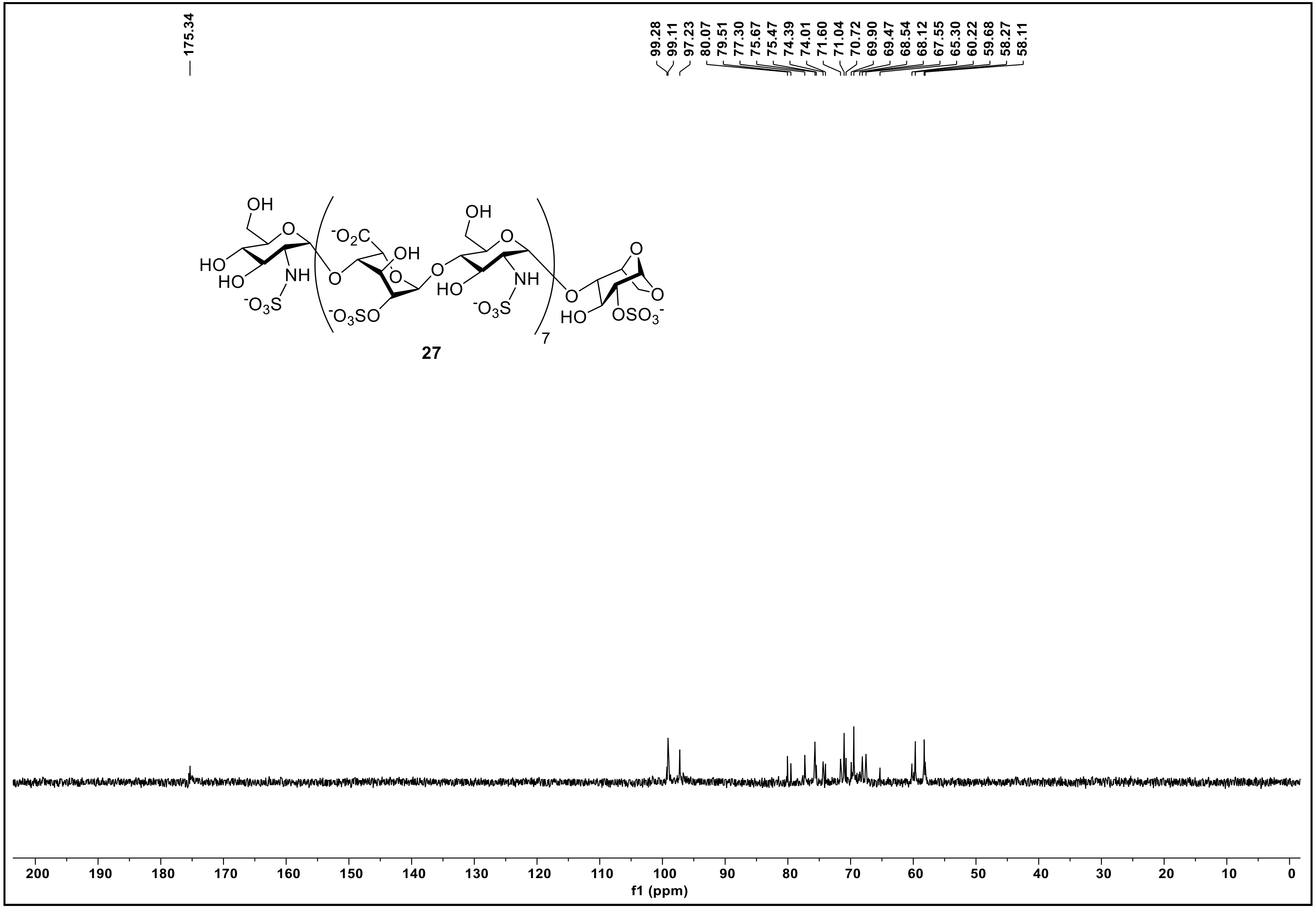
175.34
99.28
99.11
97.23
80.07
79.51
77.30
75.67
75.47
74.39
74.01
71.60
71.04
70.72
69.90
69.47
68.54
68.12
67.55
65.30
60.22
59.68
58.27
58.11
27
f1 (ppm)

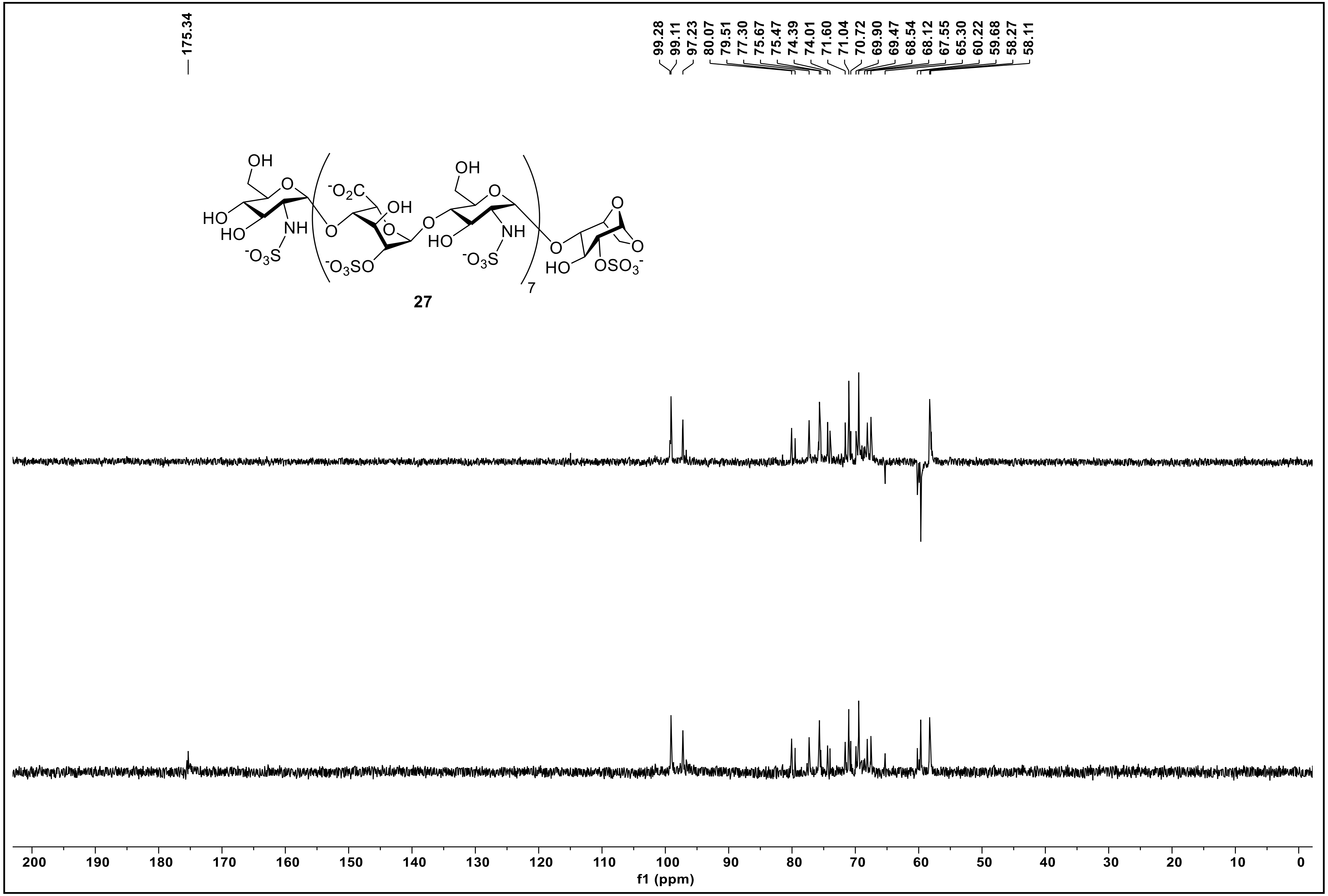
175.34
99.28
99.11
97.23
80.07
79.51
77.30
75.67
75.47
74.39
74.01
71.60
71.04
70.72
69.90
69.47
68.54
68.12
67.55
65.30
60.22
59.68
58.27
58.11
27
200 190 180 170 160 150 140 130 120 110 100 90 80 70 60 50 40 30 20 10 0
f1 (ppm)

HRMS-ESI

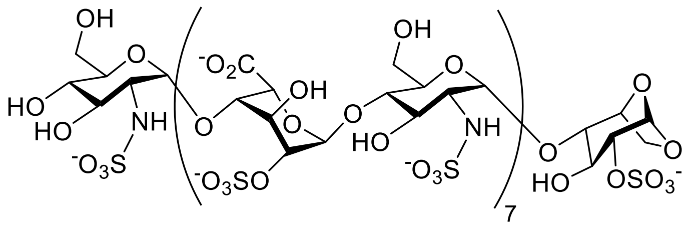

27

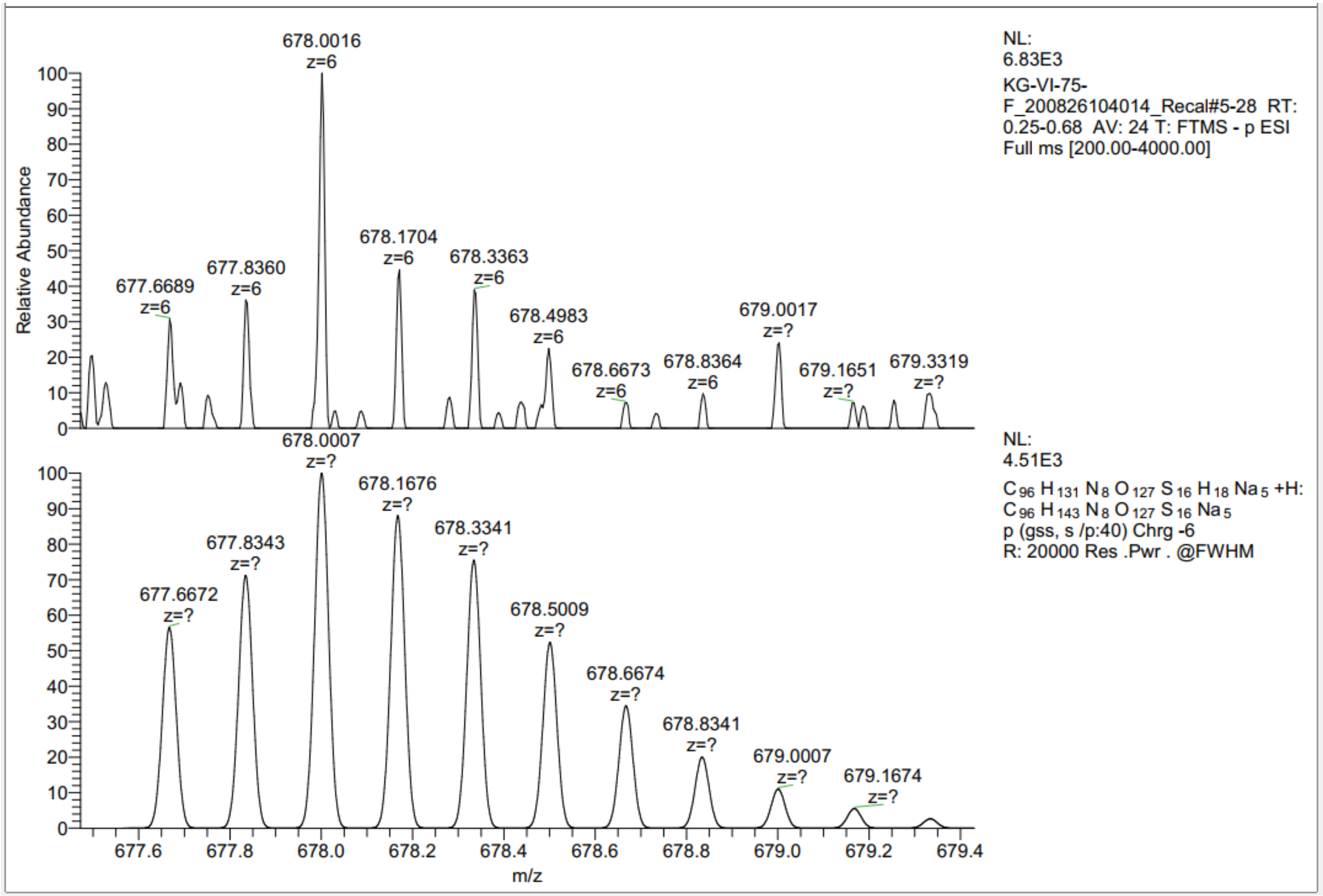

$(M + 12H^{+} + 5Na^{+})^{-6}$

Calculated : 678.0007

Found : 678.0016

Mass Error : 1.33 ppm

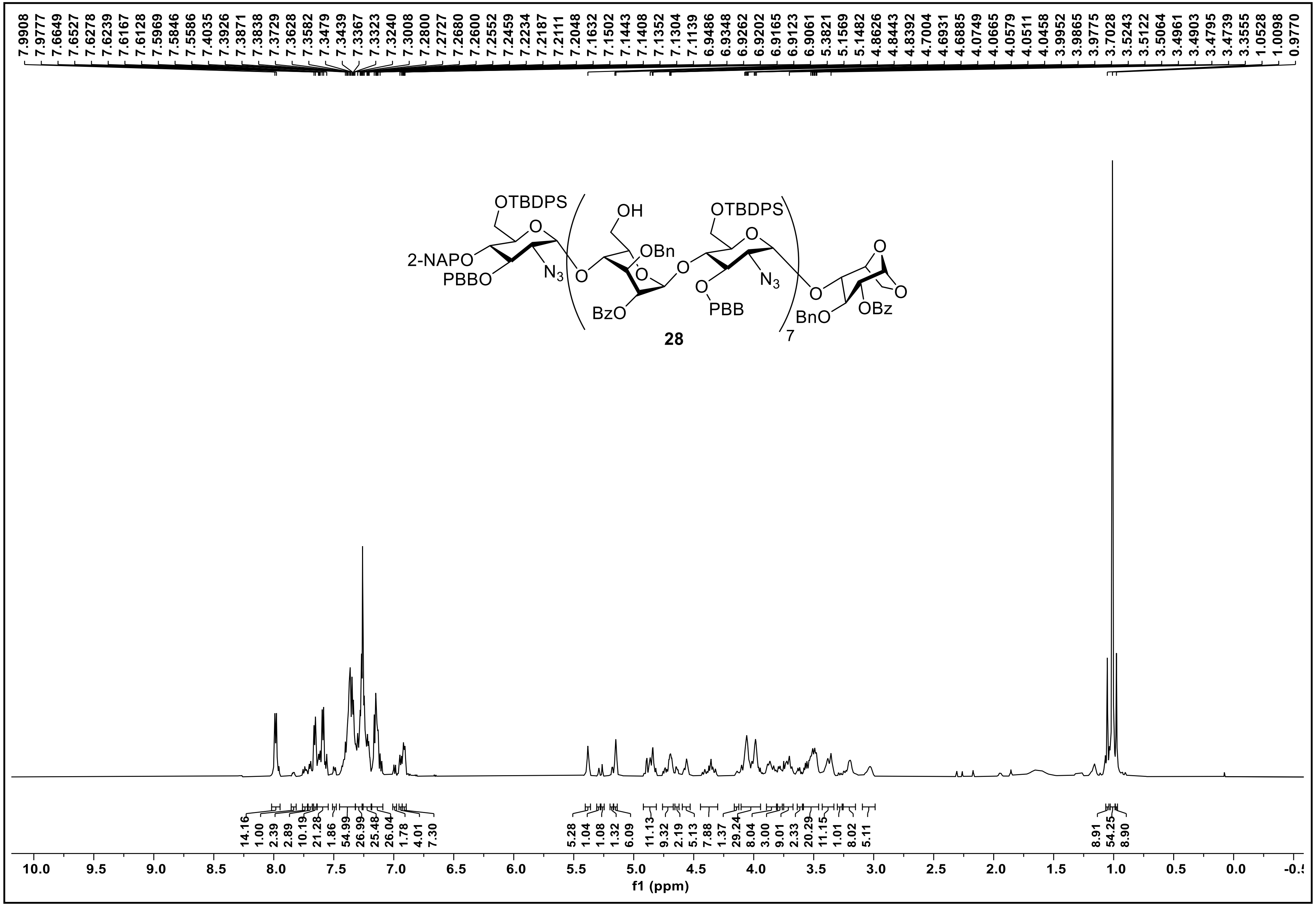

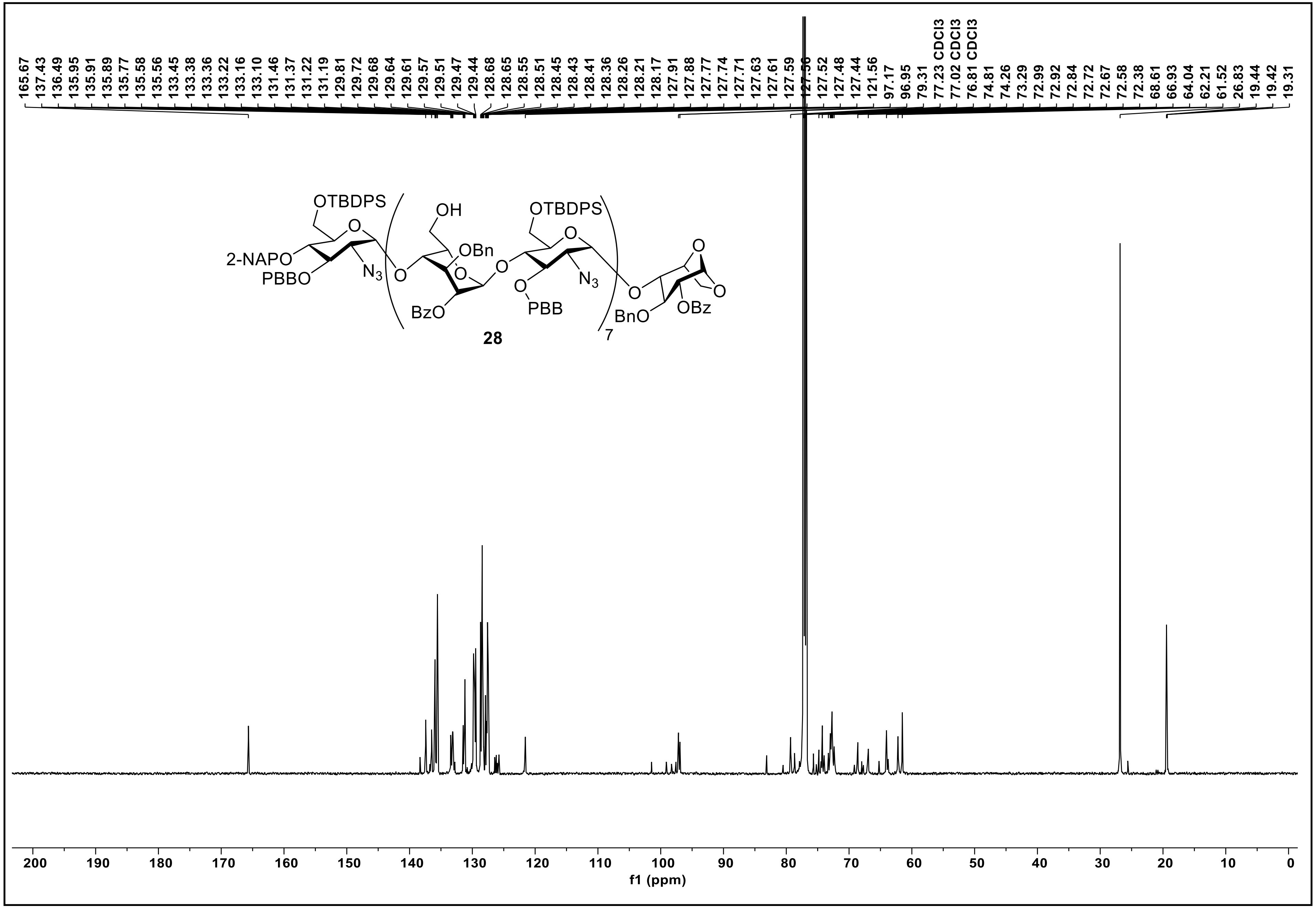
165.67
137.43
136.49
135.95
135.91
135.89
135.77
135.58
135.56
133.45
133.38
133.36
133.22
133.16
133.10
131.46
131.37
131.22
131.19
129.81
129.72
129.68
129.64
129.61
129.57
129.51
129.47
129.44
128.68
128.65
128.55
128.51
128.45
128.43
128.41
128.36
128.26
128.21
128.17
127.91
127.88
127.77
127.74
127.71
127.63
127.61
127.59
127.52
127.48
127.44
121.56
97.17
96.95
79.31
77.23 CDCl3
77.02 CDCl3
76.81 CDCl3
74.81
74.26
73.29
72.99
72.92
72.84
72.72
72.67
72.58
72.38
68.61
66.93
64.04
62.21
61.52
26.83
19.44
19.42
19.31
OTBDPS
OH
OTBDPS
2-NAPO
PBBO
N_3
O
OBn
O
O
N_3
O
BzO
PBB
7
BnO
OBz
O
O
28
200 190 180 170 160 150 140 130 120 110 100 90 80 70 60 50 40 30 20 10 0
f1 (ppm)

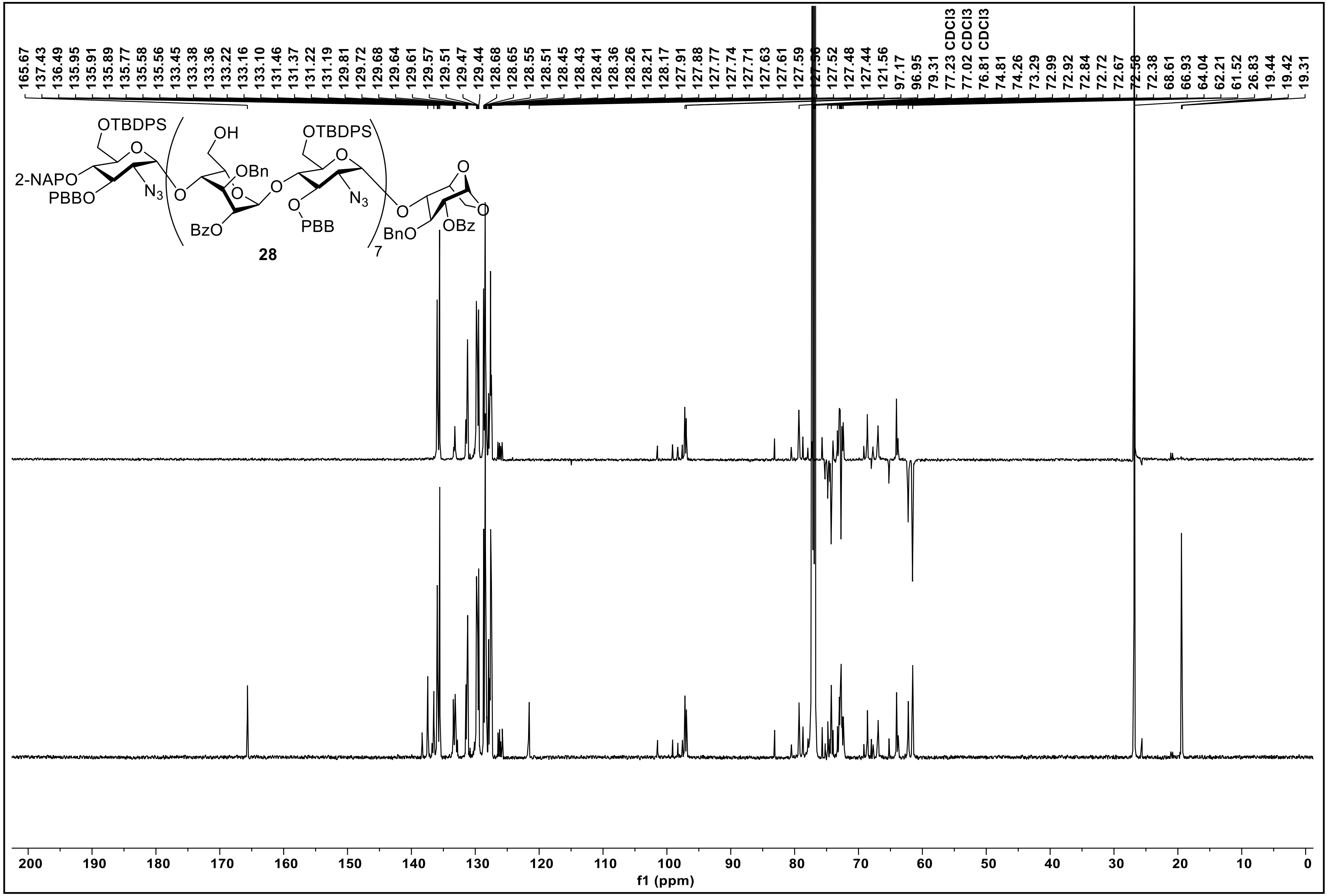
165.67
137.43
136.49
135.95
135.91
135.89
135.77
135.58
135.56
133.45
133.38
133.36
133.22
133.16
133.10
131.46
131.37
131.22
131.19
129.81
129.72
129.68
129.64
129.61
129.57
129.51
129.47
129.44
128.68
128.65
128.55
128.51
128.45
128.43
128.41
128.36
128.26
128.21
128.17
127.91
127.88
127.77
127.74
127.71
127.63
127.61
127.59
127.52
127.48
127.44
121.56
97.17
96.95
79.31
77.23 CDCl3
77.02 CDCl3
76.81 CDCl3
74.81
74.26
73.29
72.99
72.92
72.84
72.72
72.67
72.58
72.38
68.61
66.93
64.04
62.21
61.52
26.83
19.44
19.42
19.31
OTBDPS
2-NAPO
PBBO
N3
OH
OBn
BzO
PBB
OTBDPS
N3
7
BnO
OBz
28
200
190
180
170
160
150
140
130
120
110
100
90
80
70
60
50
40
30
20
10
0
f1 (ppm)

HRMS-MALDI

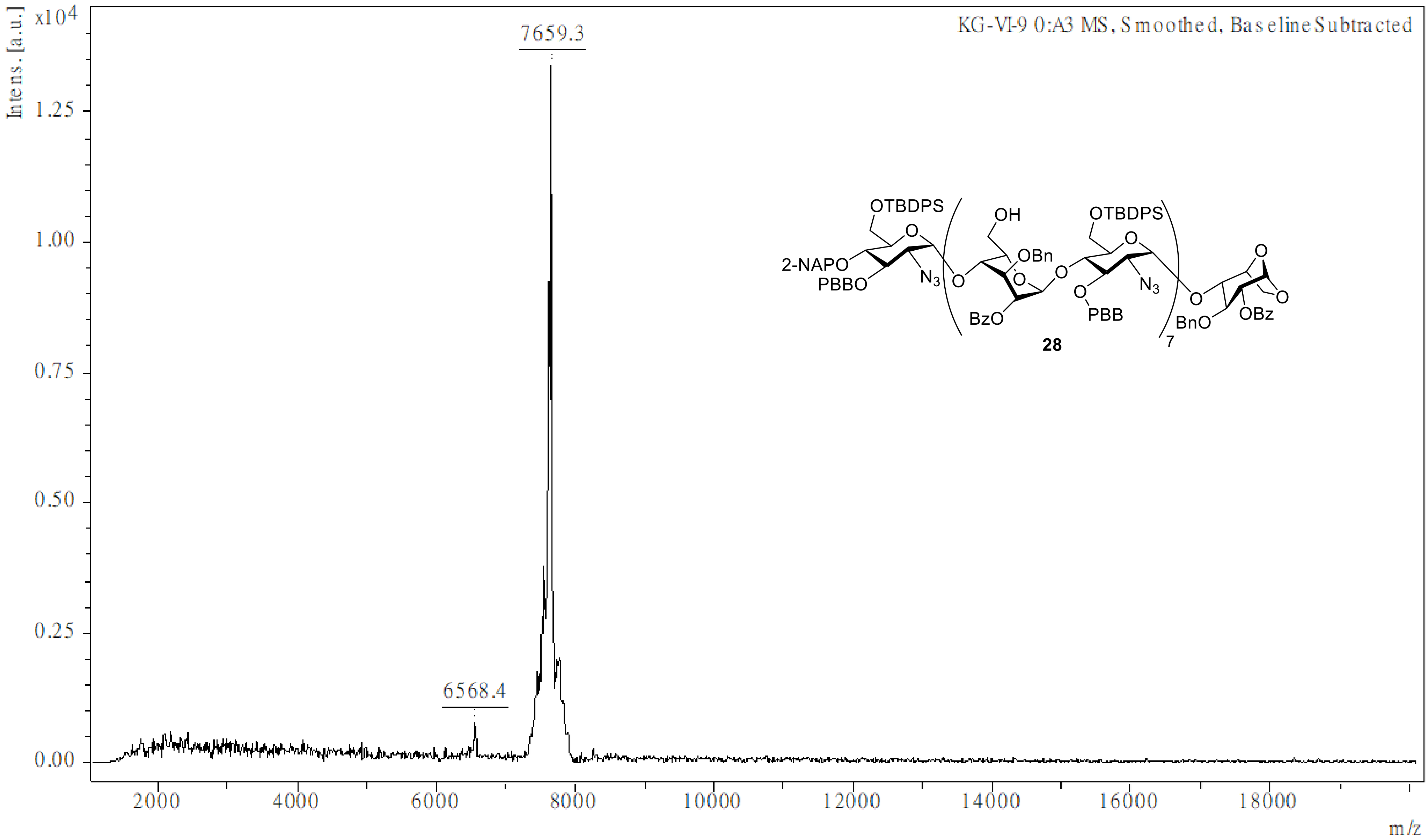

Intens. [a.u.]
x10^4
KG-VI-9 0:A3 MS, Smoothed, Baseline Subtracted
7659.3
6568.4
1.25
1.00
0.75
0.50
0.25
0.00
2000
4000
6000
8000
10000
12000
14000
16000
18000
m/z
OTBDPS
OH
OTBDPS
2-NAPO
PBBO
N3
OBn
O
BzO
PBB
N3
BnO
OBz
7
28

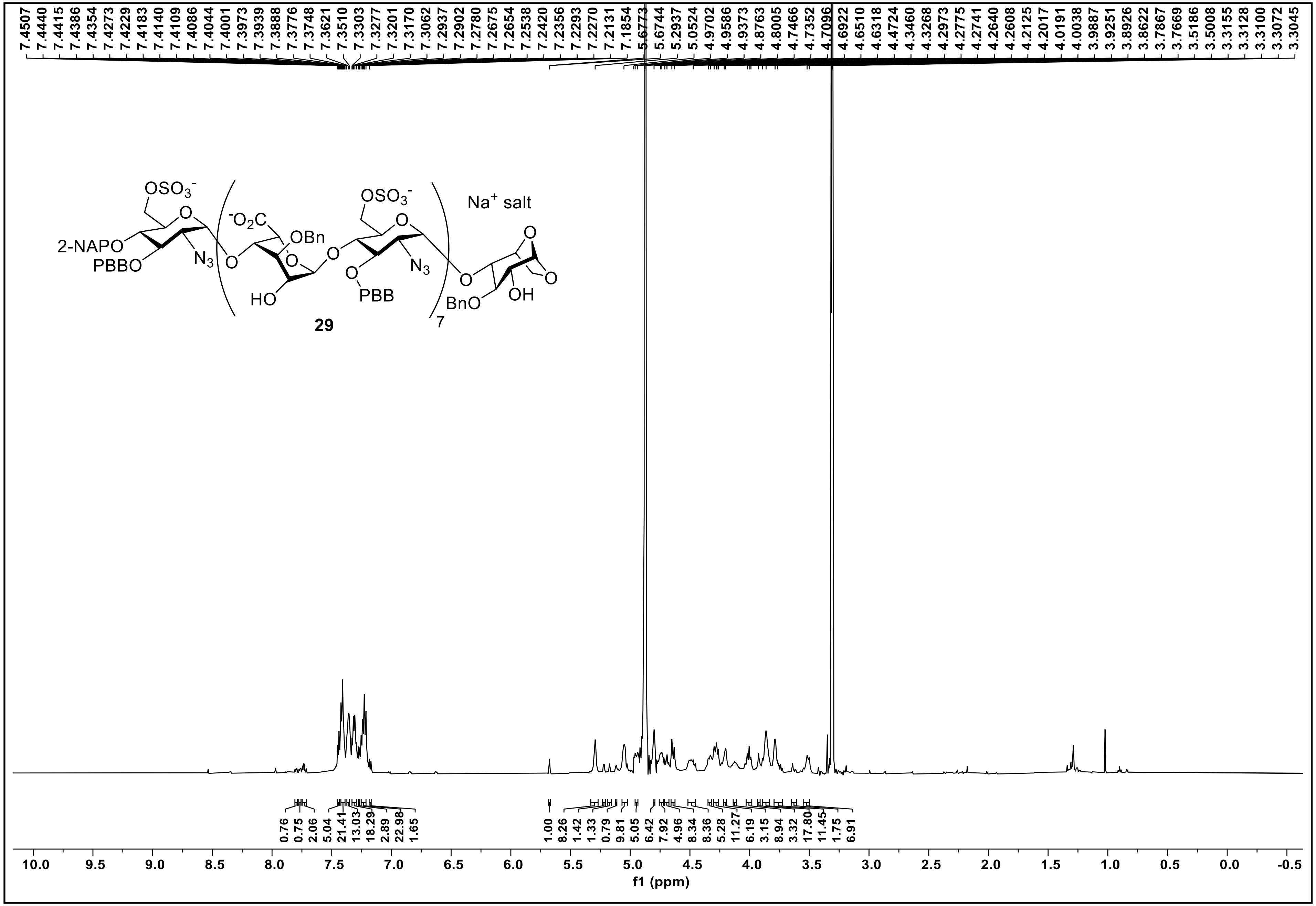

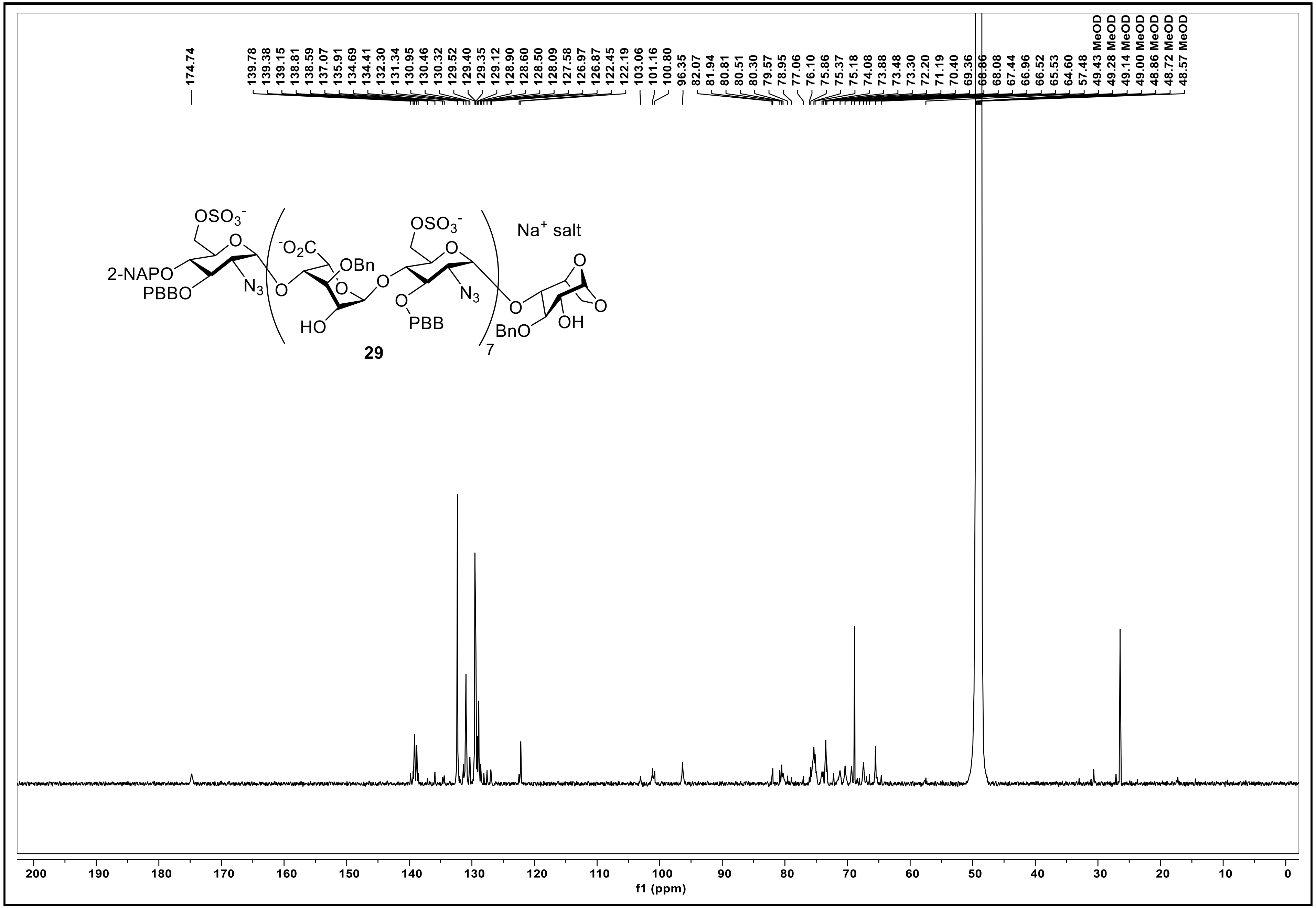
174.74
139.78
139.38
139.15
138.81
138.59
137.07
135.91
134.69
134.41
132.30
131.34
130.95
130.46
130.32
129.52
129.40
129.35
129.12
128.90
128.60
128.50
128.09
127.58
126.97
126.87
122.45
122.19
103.06
101.16
100.80
96.35
82.07
81.94
80.81
80.51
80.30
79.57
78.95
77.06
76.10
75.86
75.37
75.18
74.08
73.88
73.48
73.30
72.20
71.19
70.40
69.36
68.86
68.08
67.44
66.96
66.52
65.53
64.60
57.48
49.43 MeOD
49.28 MeOD
49.14 MeOD
49.00 MeOD
48.86 MeOD
48.72 MeOD
48.57 MeOD
OSO3-
2-NAPO
PBBO
N3
-O2C
OBn
HO
OSO3-
PBB
N3
7
Na+ salt
BnO
OH
29
200 190 180 170 160 150 140 130 120 110 100 90 80 70 60 50 40 30 20 10 0
f1 (ppm)

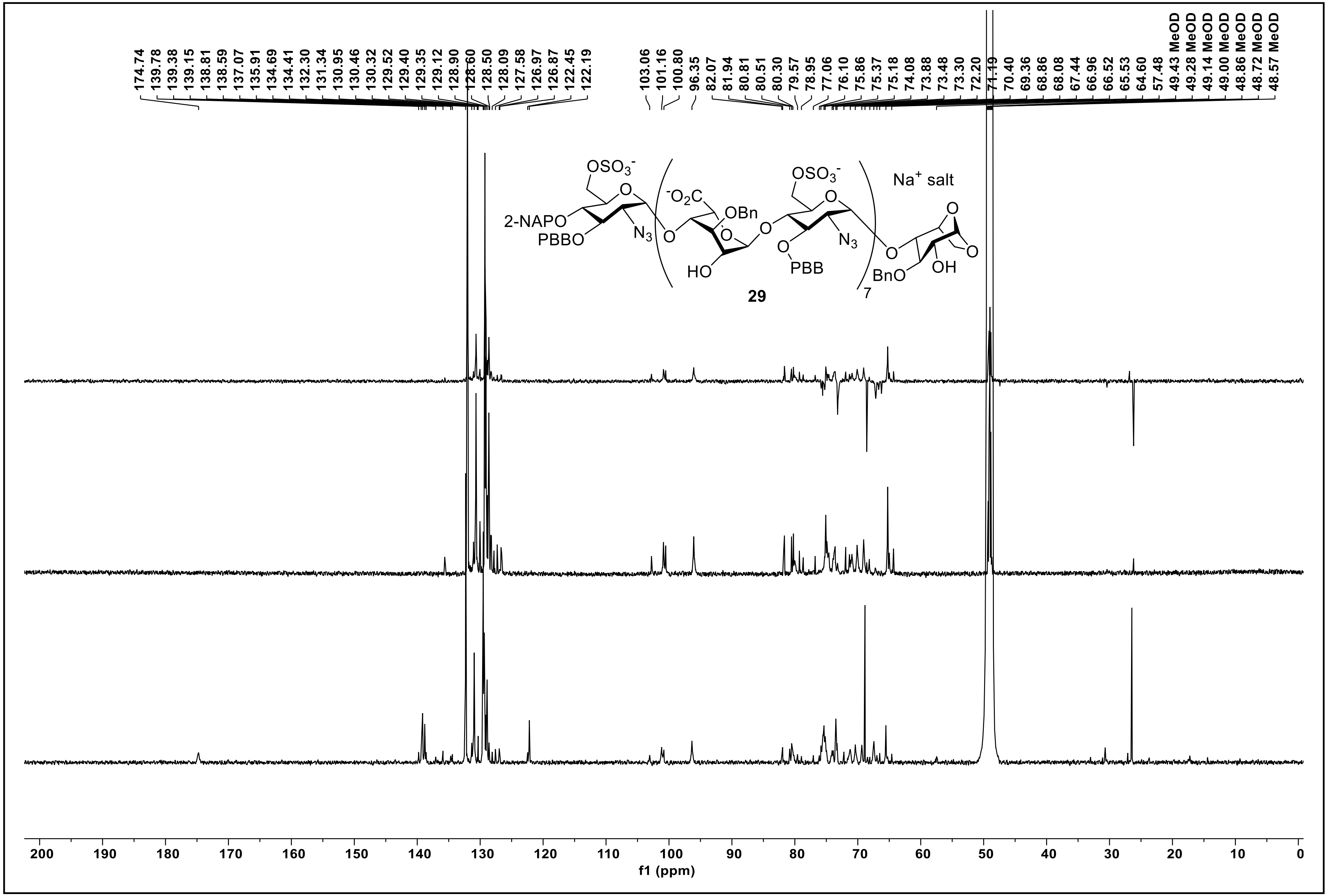

174.74
139.78
139.38
139.15
138.81
138.59
137.07
135.91
134.69
134.41
132.30
131.34
130.95
130.46
130.32
129.52
129.40
129.35
129.12
128.90
128.60
128.50
128.09
127.58
126.97
126.87
122.45
122.19
103.06
101.16
100.80
96.35
82.07
81.94
80.81
80.51
80.30
79.57
78.95
77.06
76.10
75.86
75.37
75.18
74.08
73.88
73.48
73.30
72.20
71.19
70.40
69.36
68.86
68.08
67.44
66.96
66.52
65.53
64.60
57.48
49.43 MeOD
49.28 MeOD
49.14 MeOD
49.00 MeOD
48.86 MeOD
48.72 MeOD
48.57 MeOD
Na+ salt
29
f1 (ppm)

HRMS-ESI

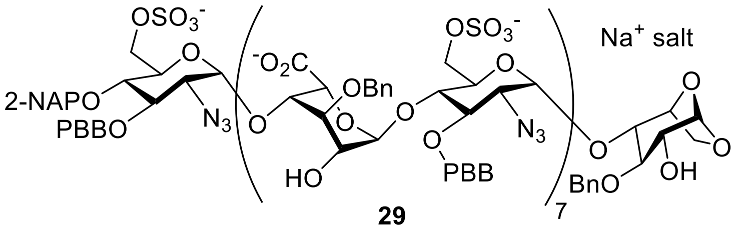

29

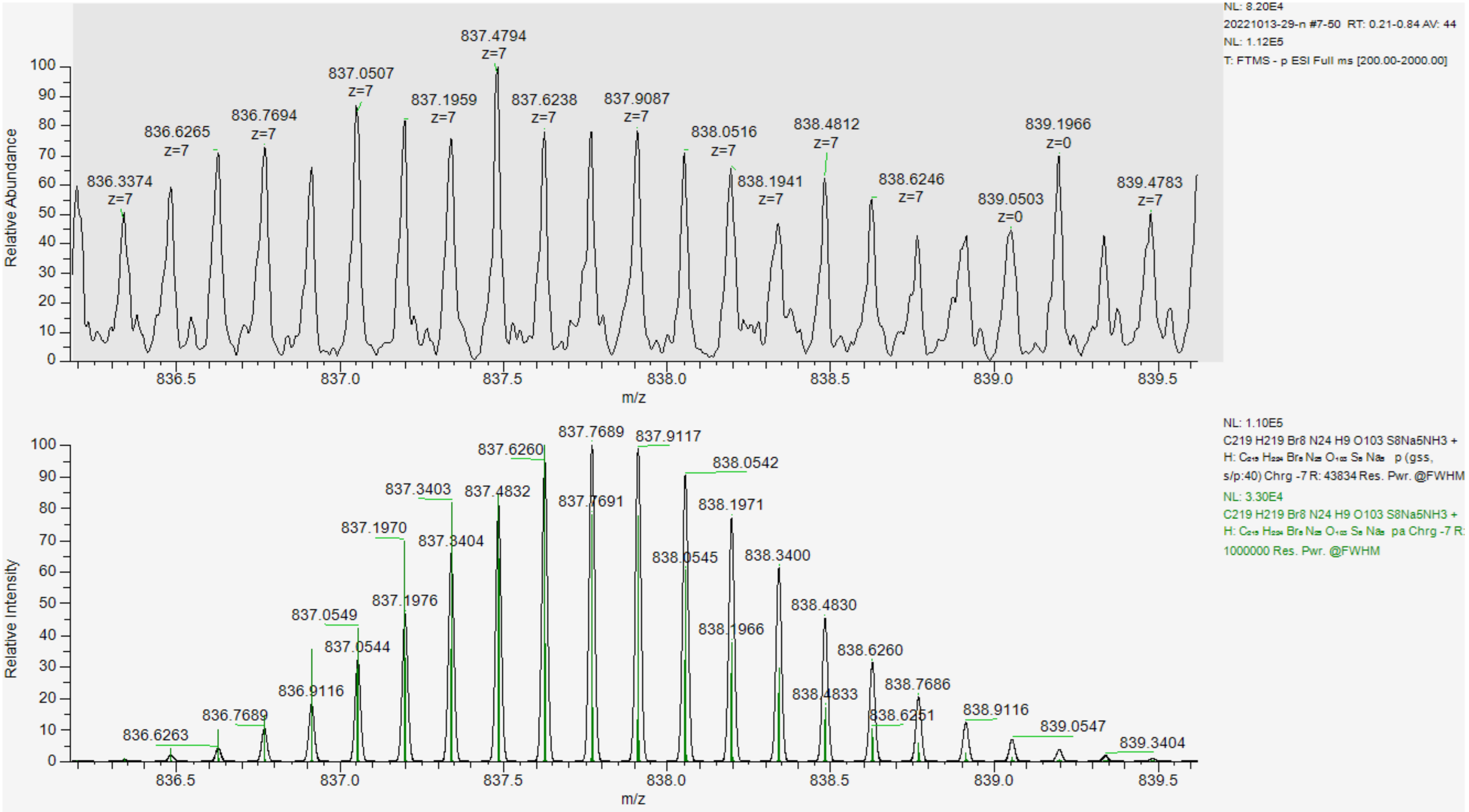

$(M + 3H^+ + 5Na^+)^{-7}$

Calculated : 837.6260

Found : 837.6238

Mass Error : 2.63 ppm

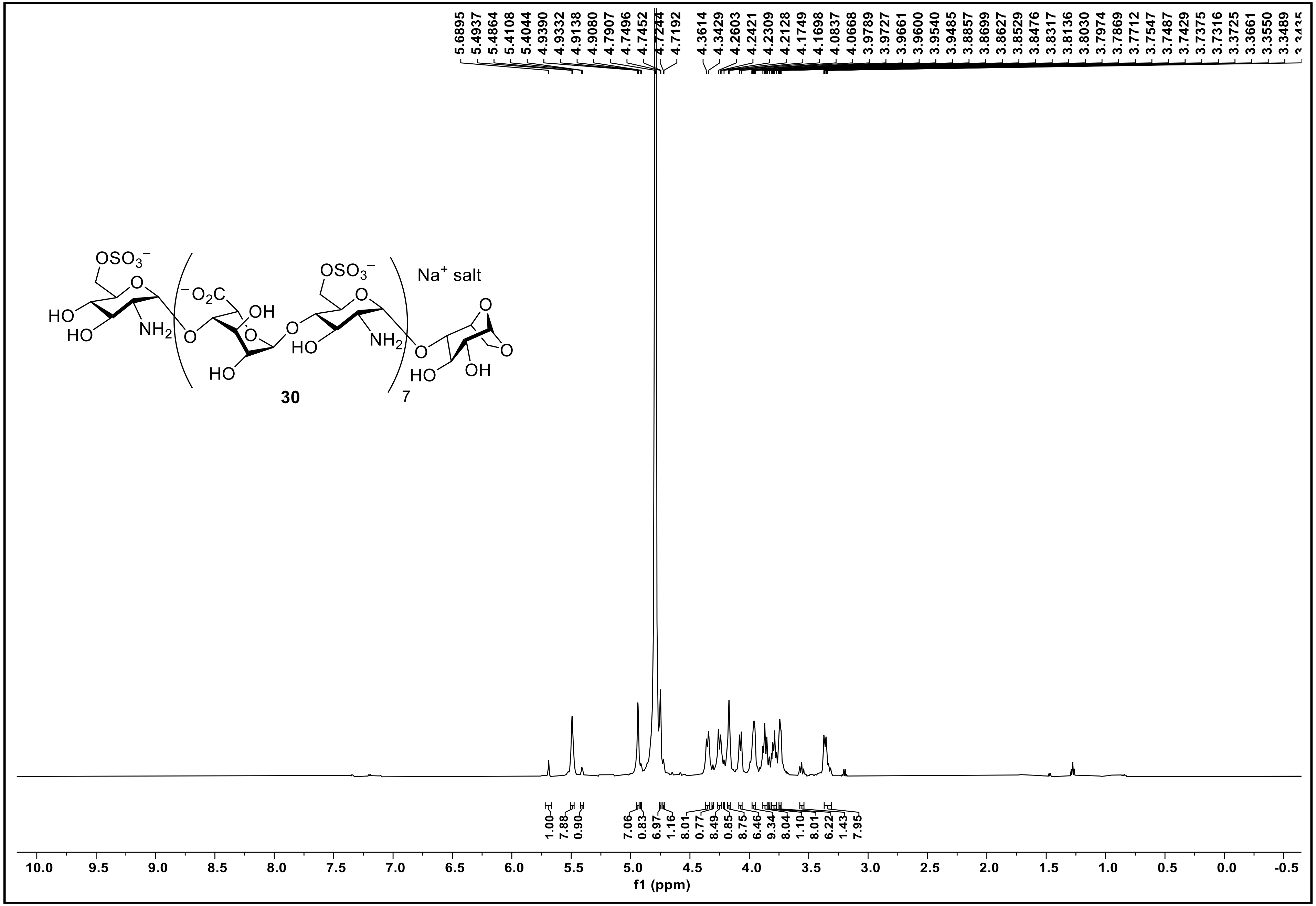

5.6895
5.4937
5.4864
5.4108
5.4044
4.9390
4.9332
4.9138
4.9080
4.7907
4.7496
4.7452
4.7244
4.7192
4.3614
4.3429
4.2603
4.2421
4.2309
4.2128
4.1749
4.1698
4.0837
4.0668
3.9789
3.9727
3.9661
3.9600
3.9540
3.9485
3.8857
3.8699
3.8627
3.8529
3.8476
3.8317
3.8136
3.8030
3.7974
3.7869
3.7712
3.7547
3.7487
3.7429
3.7375
3.7316
3.3725
3.3661
3.3550
3.3489
Na+ salt
30
7
1.00
7.88
0.90
7.06
0.83
6.97
1.16
8.01
0.77
8.49
0.85
8.75
6.46
9.34
8.04
1.10
8.01
6.22
1.43
7.95
10.0
9.5
9.0
8.5
8.0
7.5
7.0
6.5
6.0
5.5
5.0
4.5
4.0
3.5
3.0
2.5
2.0
1.5
1.0
0.5
0.0
-0.5
f1 (ppm)

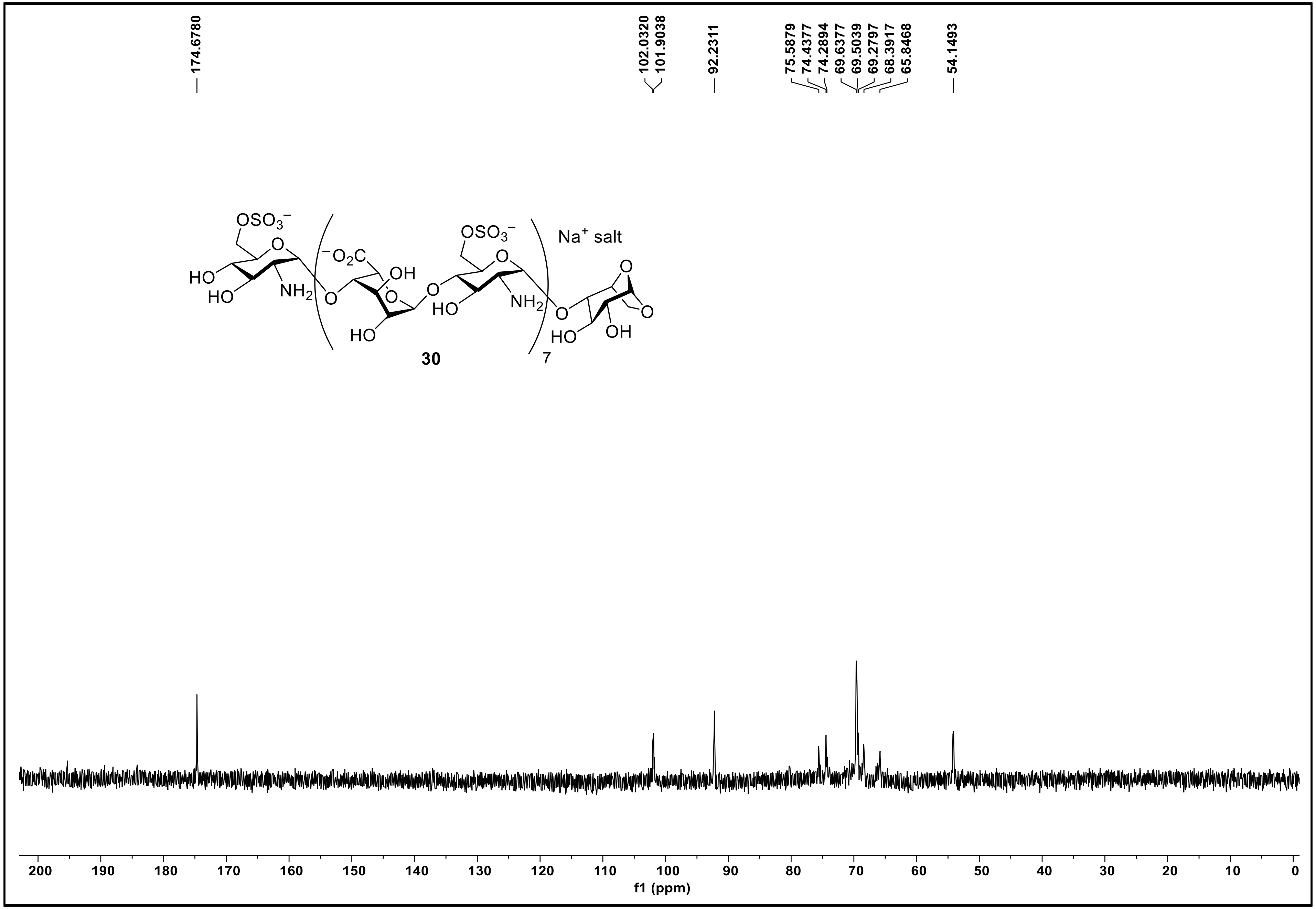
174.6780
102.0320
101.9038
92.2311
75.5879
74.4377
74.2894
69.6377
69.5039
69.2797
68.3917
65.8468
54.1493
Na+ salt
30
7
200 190 180 170 160 150 140 130 120 110 100 90 80 70 60 50 40 30 20 10 0
f1 (ppm)

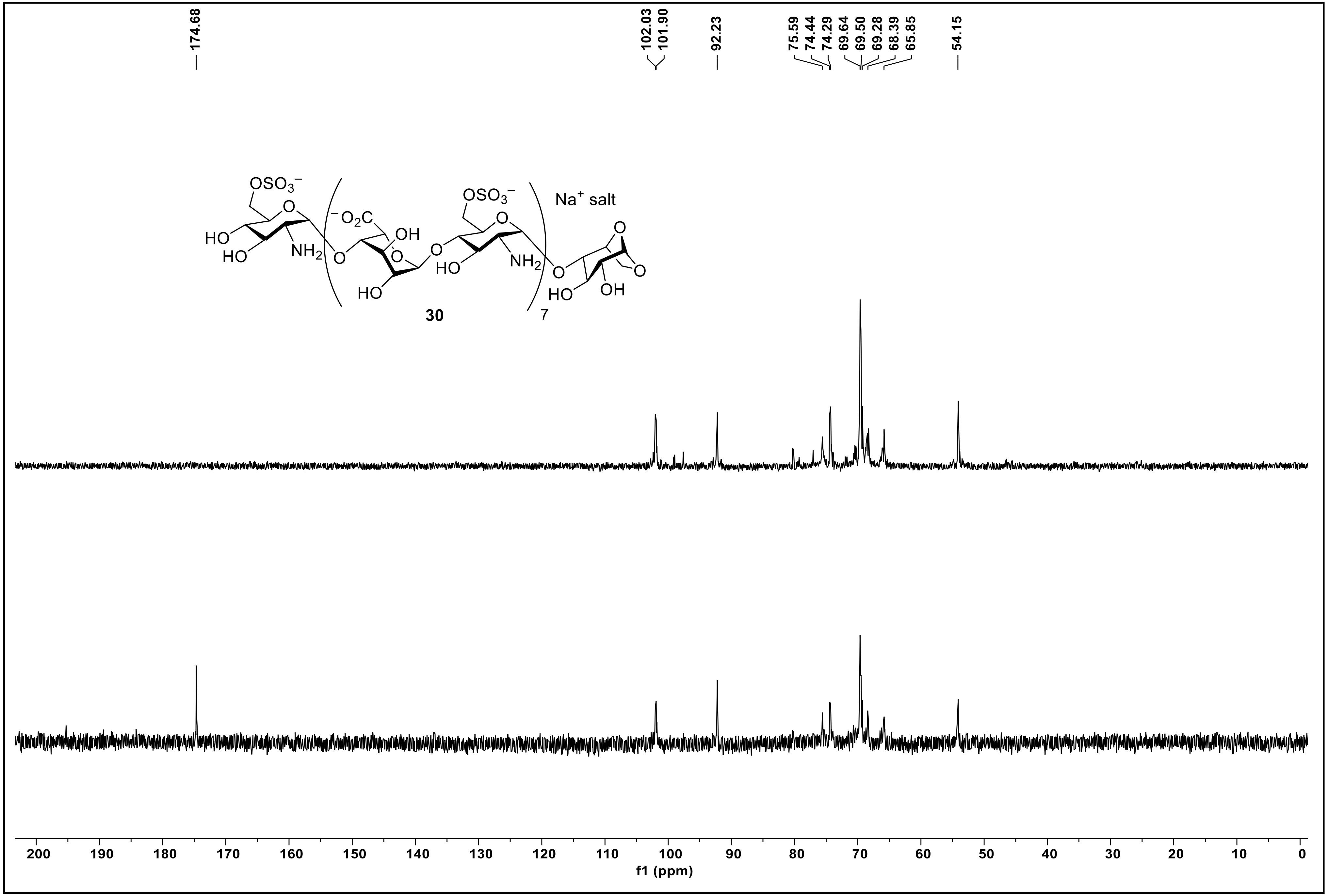
174.68
102.03
101.90
92.23
75.59
74.44
74.29
69.64
69.50
69.28
68.39
65.85
54.15
OSO3−
Na+ salt
HO
NH2
OH
O
30
7
f1 (ppm)

HRMS-ESI

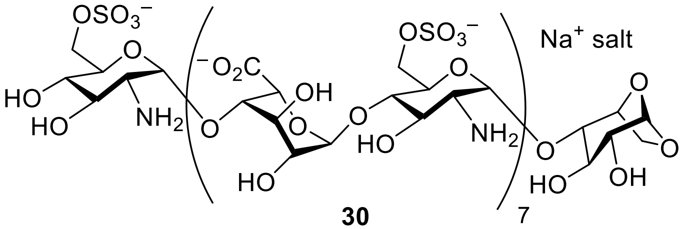

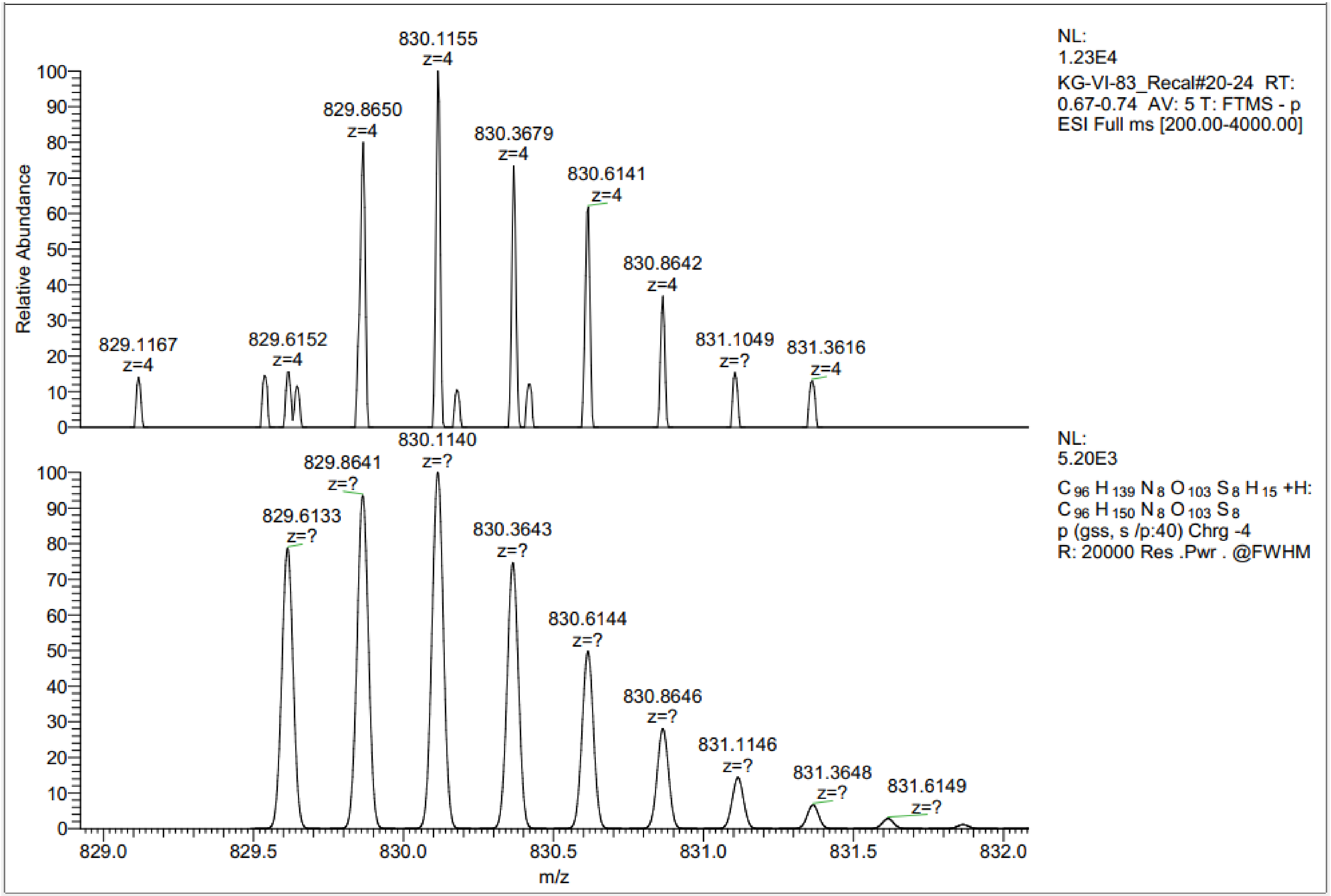

$(M + 11H^+)^{-4}$

Calculated : 830.1140

Found : 830.1155

Mass Error : 1.81 ppm

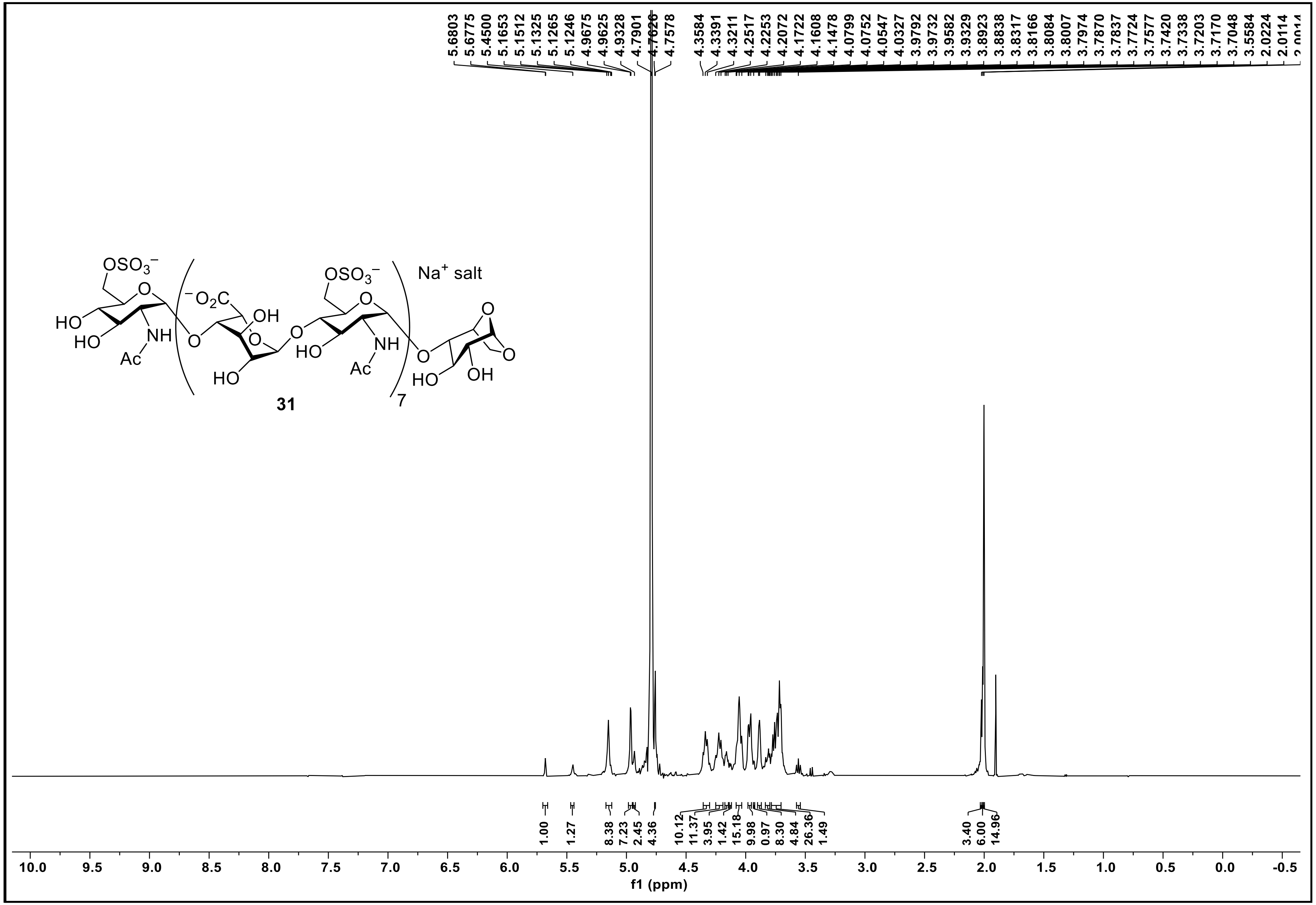
5.6803
5.6775
5.4500
5.1653
5.1512
5.1325
5.1265
5.1246
4.9675
4.9625
4.9328
4.7901
4.7620
4.7578
4.3584
4.3391
4.3211
4.2517
4.2253
4.2072
4.1722
4.1608
4.1478
4.0799
4.0752
4.0547
4.0327
3.9792
3.9732
3.9582
3.9329
3.8923
3.8838
3.8317
3.8166
3.8084
3.8007
3.7974
3.7870
3.7837
3.7724
3.7577
3.7420
3.7338
3.7203
3.7170
3.7048
3.5584
2.0224
2.0114
OSO3−
Na+ salt
−O2C
OH
HO
NH
Ac
O
7
31
1.00
1.27
8.38
7.23
2.45
4.36
10.12
11.37
3.95
1.42
15.18
9.98
0.97
8.30
4.84
26.36
1.49
3.40
6.00
14.96
10.0 9.5 9.0 8.5 8.0 7.5 7.0 6.5 6.0 5.5 5.0 4.5 4.0 3.5 3.0 2.5 2.0 1.5 1.0 0.5 0.0 -0.5
f1 (ppm)

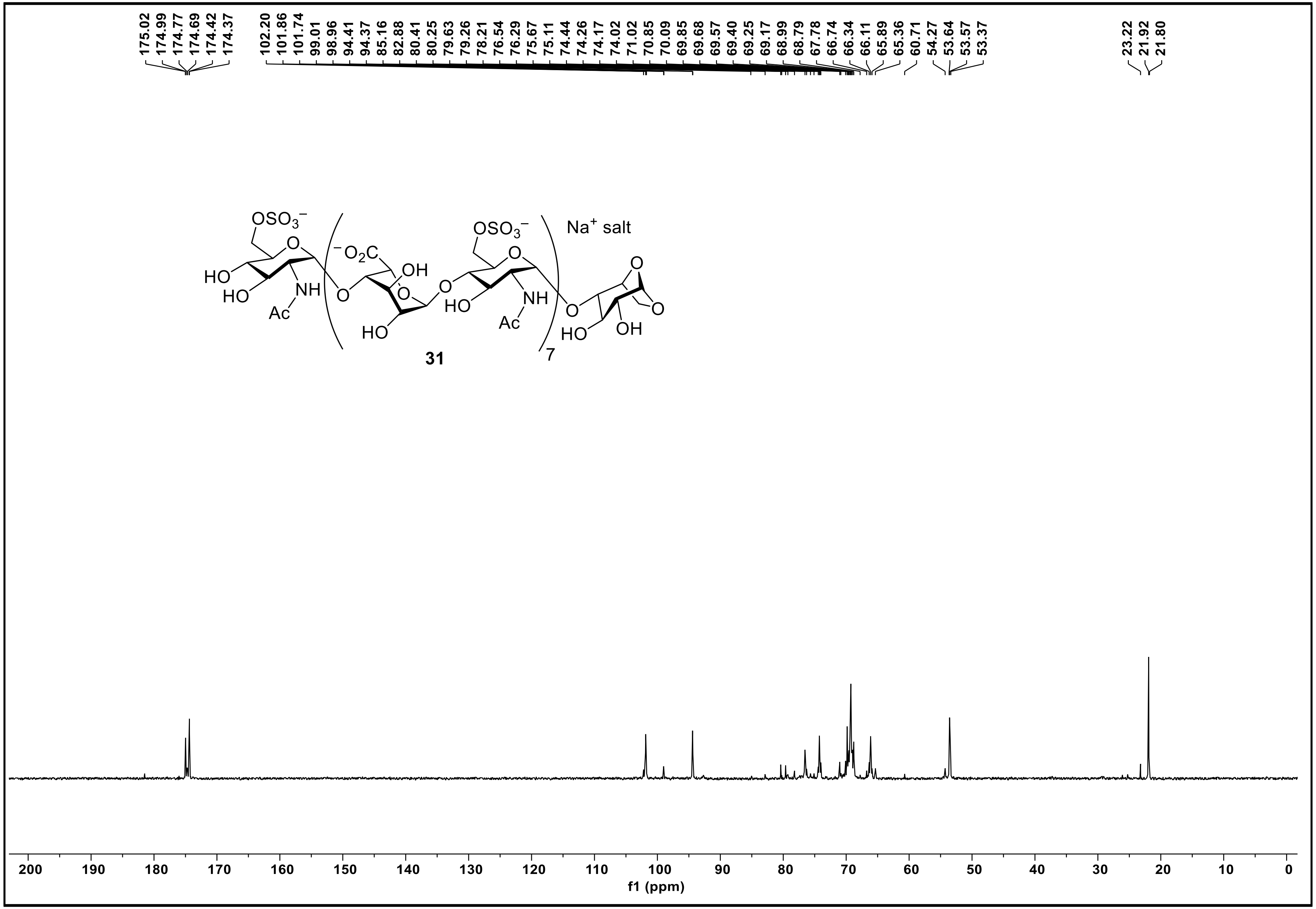
175.02
174.99
174.77
174.69
174.42
174.37
102.20
101.86
101.74
99.01
98.96
94.41
94.37
85.16
82.88
80.41
80.25
79.63
79.26
78.21
76.54
76.29
75.67
75.11
74.44
74.26
74.17
74.02
71.02
70.85
70.09
69.85
69.68
69.57
69.40
69.25
69.17
68.99
68.79
67.78
66.74
66.34
66.11
65.89
65.36
60.71
54.27
53.64
53.57
53.37
23.22
21.92
21.80
OSO3−
−O2C
OH
HO
HO
NH
Ac
O
HO
HO
Na+ salt
OH
31
7
200 190 180 170 160 150 140 130 120 110 100 90 80 70 60 50 40 30 20 10 0
f1 (ppm)

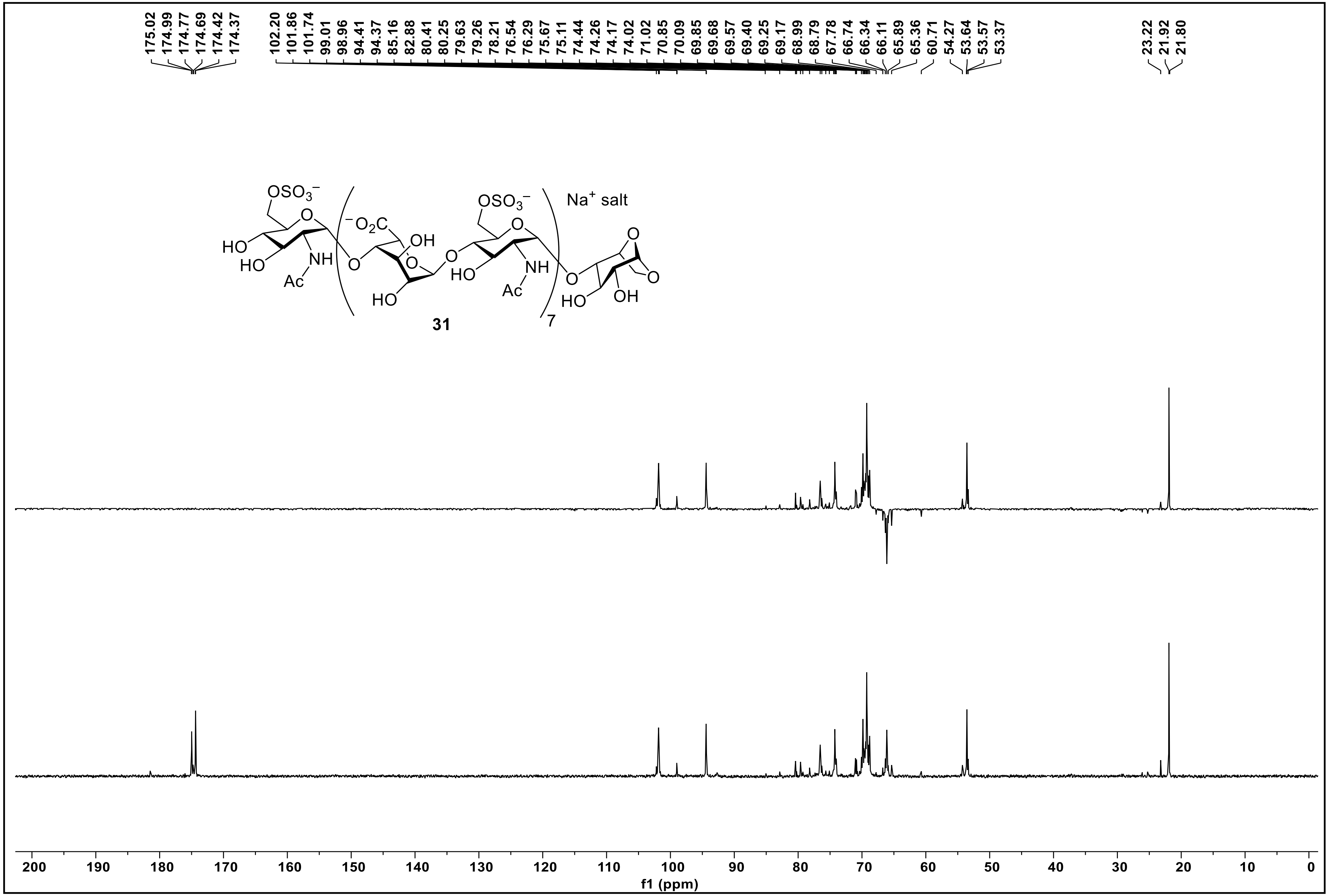
175.02
174.99
174.77
174.69
174.42
174.37
102.20
101.86
101.74
99.01
98.96
94.41
94.37
85.16
82.88
80.41
80.25
79.63
79.26
78.21
76.54
76.29
75.67
75.11
74.44
74.26
74.17
74.02
71.02
70.85
70.09
69.85
69.68
69.57
69.40
69.25
69.17
68.99
68.79
67.78
66.74
66.34
66.11
65.89
65.36
60.71
54.27
53.64
53.57
53.37
23.22
21.92
21.80
Na+ salt
31
7
f1 (ppm)

HRMS-ESI

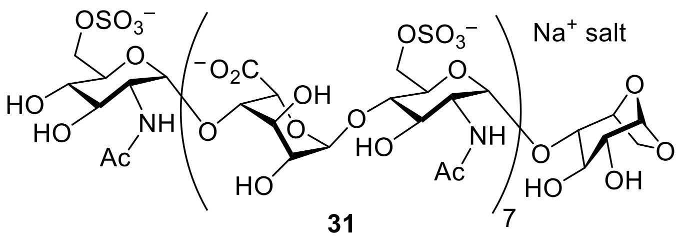

31

(M + 10H⁺)⁻⁵

Calculated : 730.9067

Found : 730.9037

Mass Error : 4.10 ppm

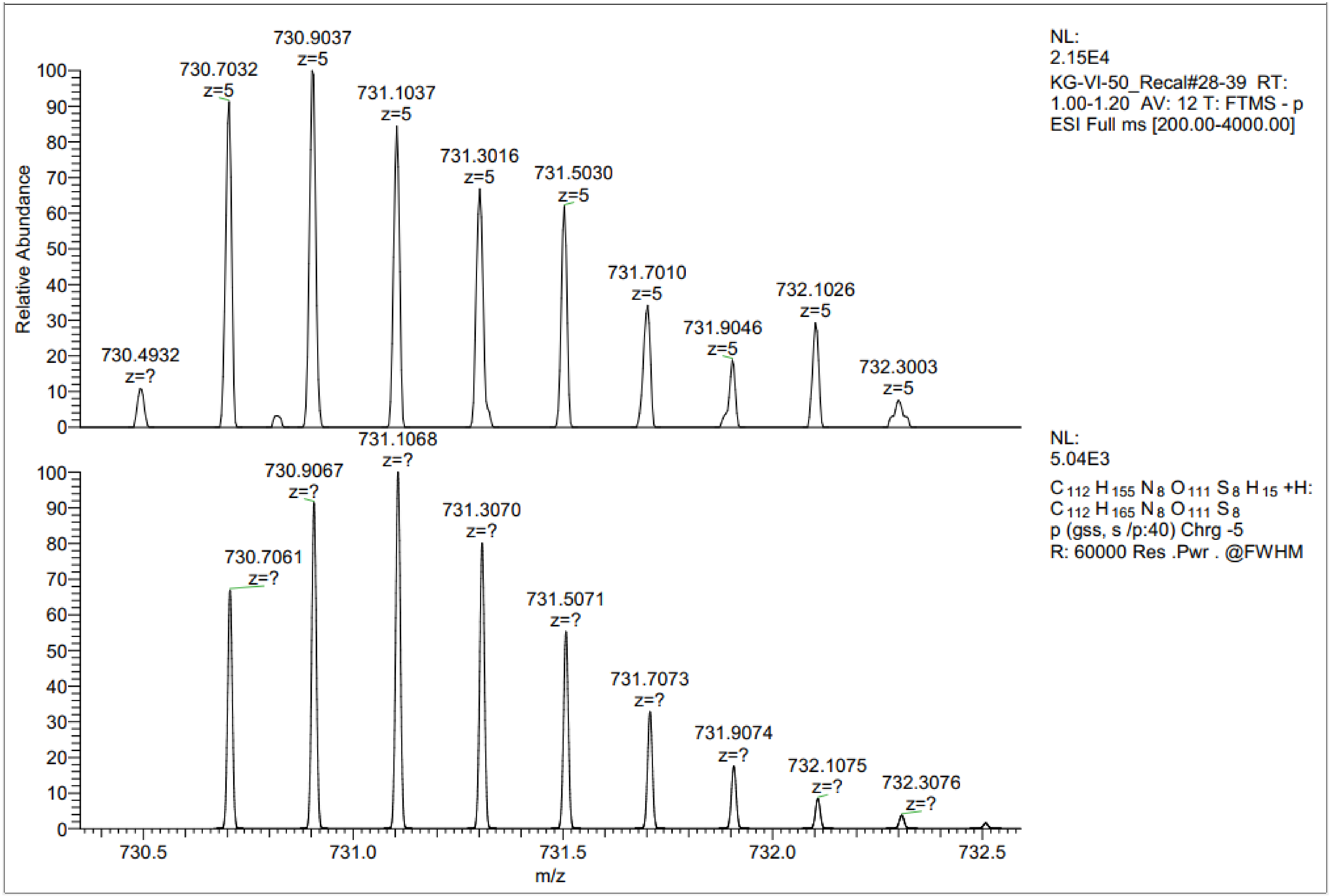

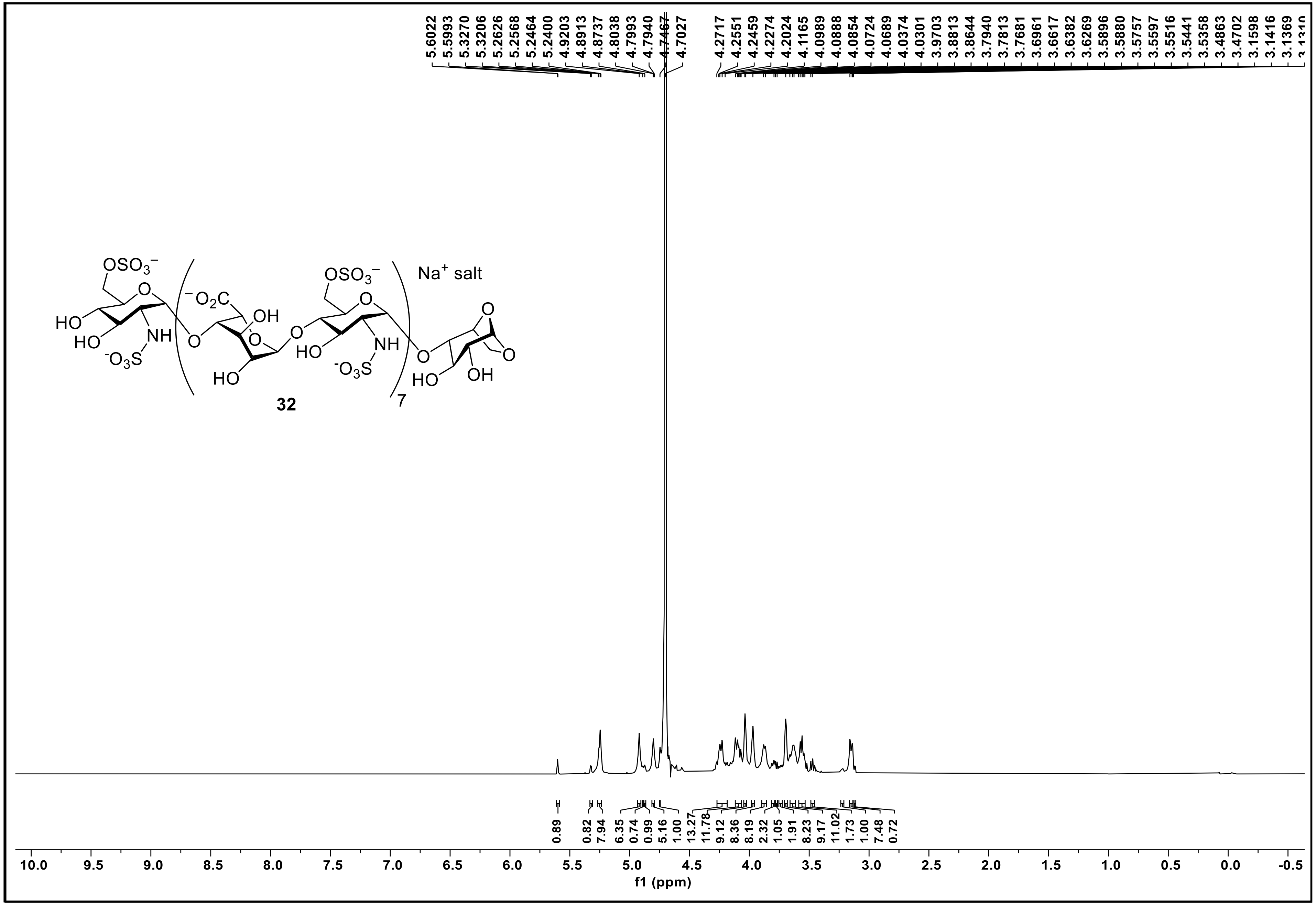
Na+ salt
32
f1 (ppm)

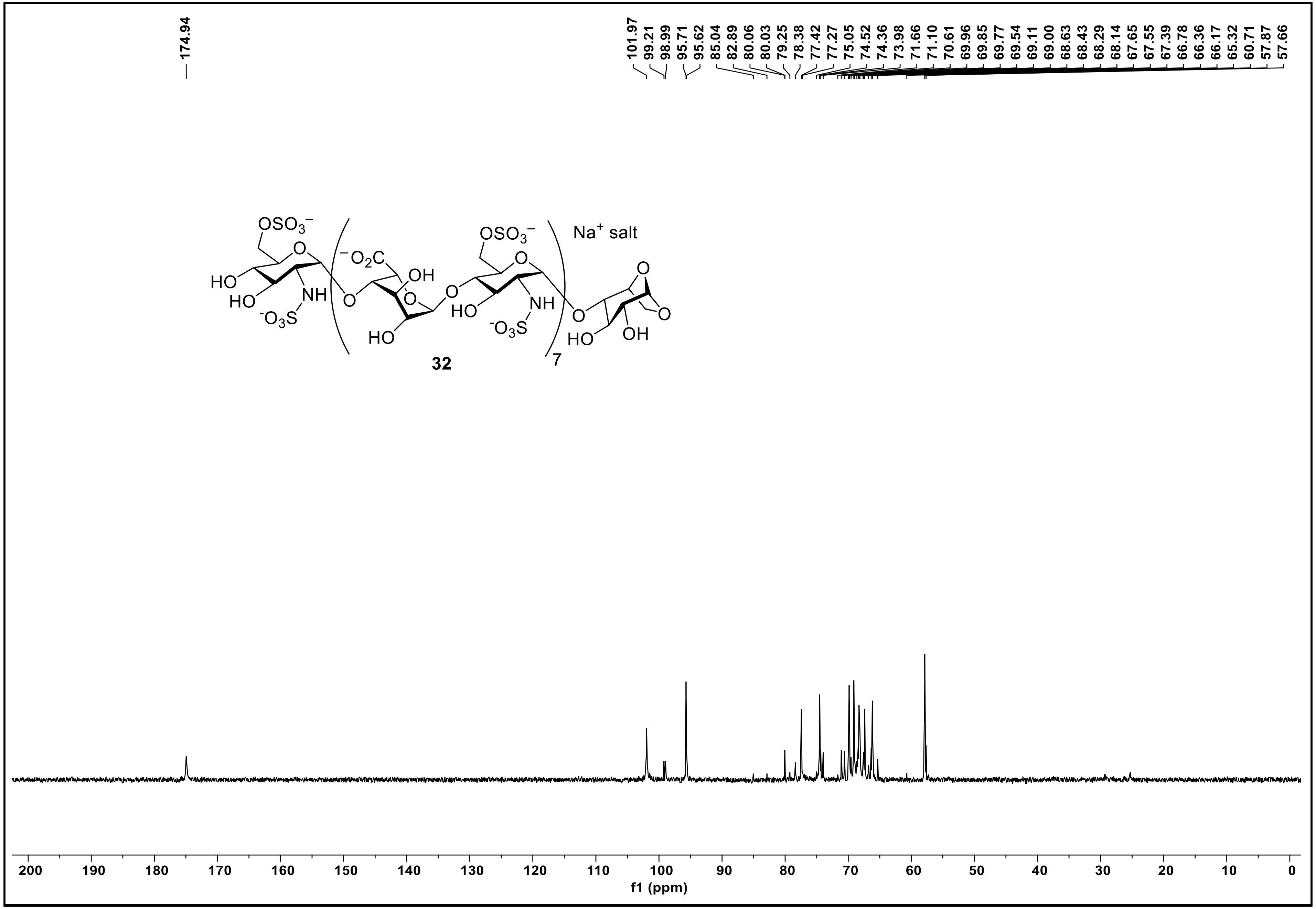
174.94
101.97
99.21
98.99
95.71
95.62
85.04
82.89
80.06
80.03
79.25
78.38
77.42
77.27
75.05
74.52
74.36
73.98
71.66
71.10
70.61
69.96
69.85
69.77
69.54
69.11
69.00
68.63
68.43
68.29
68.14
67.65
67.55
67.39
66.78
66.36
66.17
65.32
60.71
57.87
57.66
Na+ salt
32
f1 (ppm)

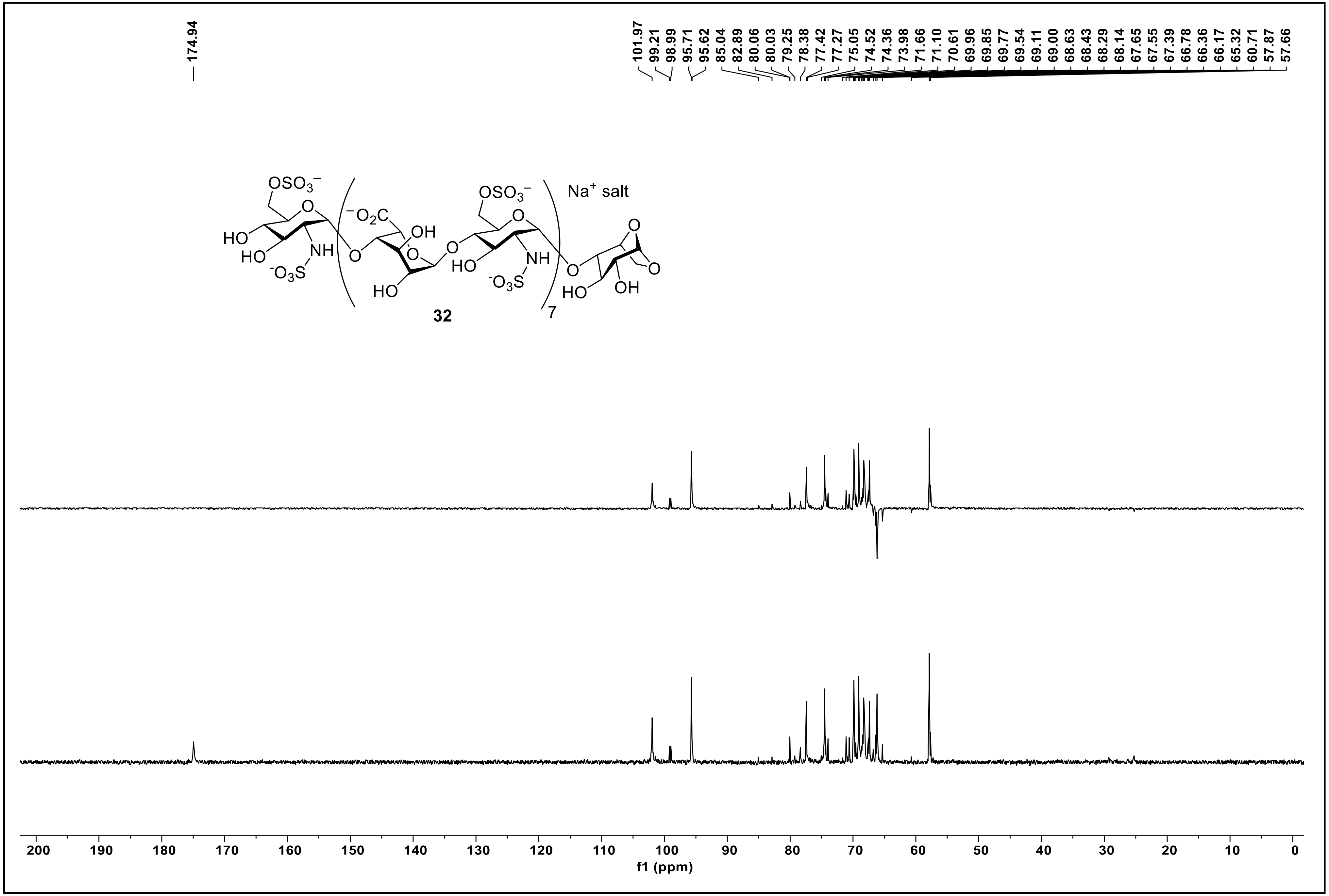
174.94
101.97
99.21
98.99
95.71
95.62
85.04
82.89
80.06
80.03
79.25
78.38
77.42
77.27
75.05
74.52
74.36
73.98
71.66
71.10
70.61
69.96
69.85
69.77
69.54
69.11
69.00
68.63
68.43
68.29
68.14
67.65
67.55
67.39
66.78
66.36
66.17
65.32
60.71
57.87
57.66
Na+ salt
32
7
f1 (ppm)

HRMS-ESI

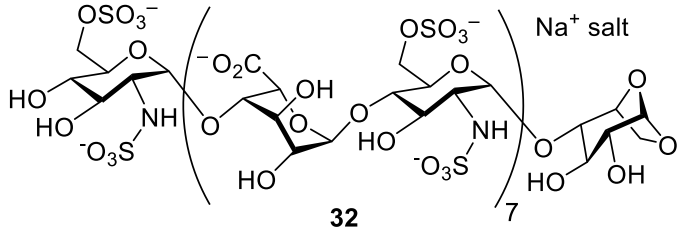

32

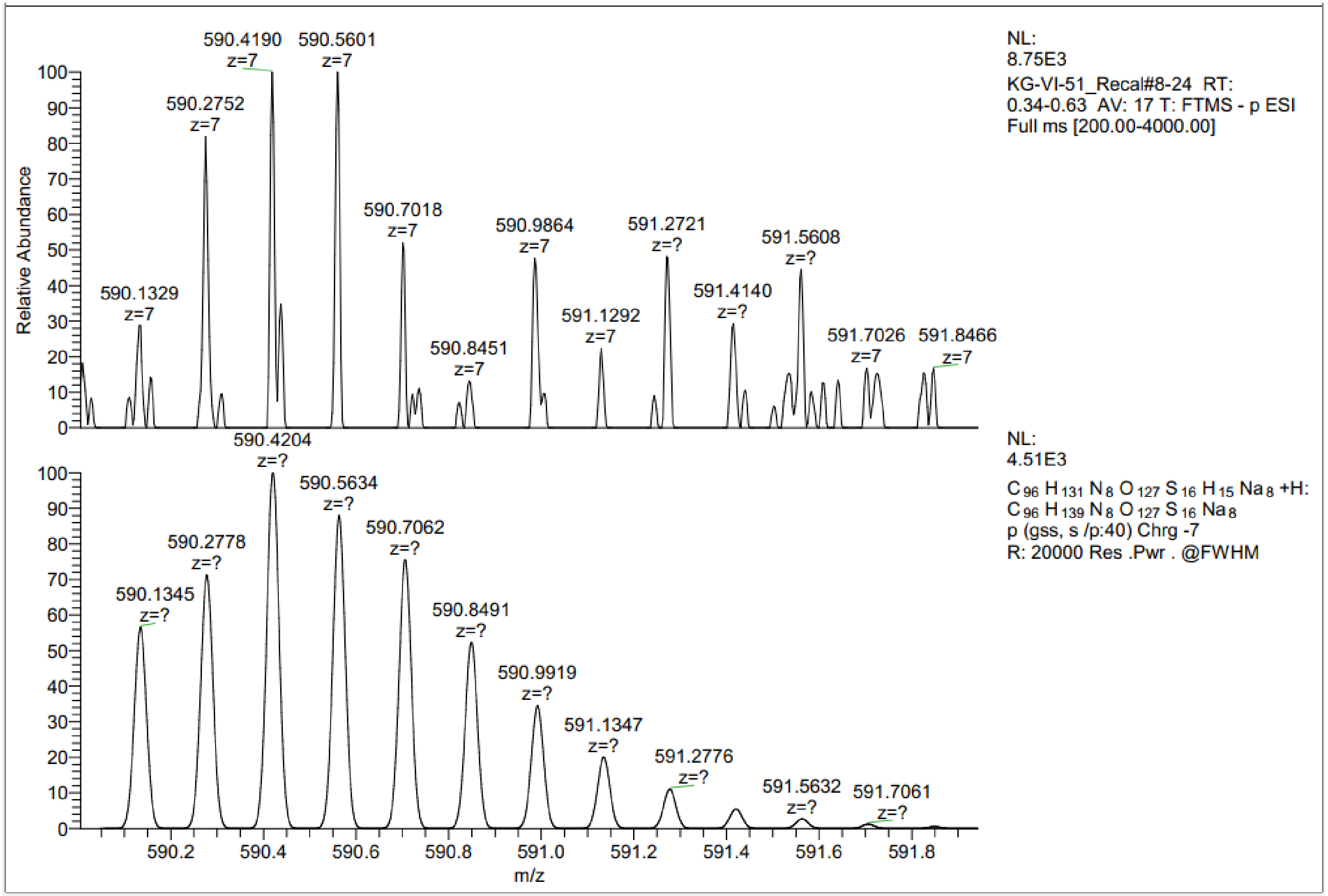

$(M + 8H^{+} + 8Na^{+})^{-7}$

Calculated : 590.1345

Found : 590.1329

Mass Error : 2.71 ppm

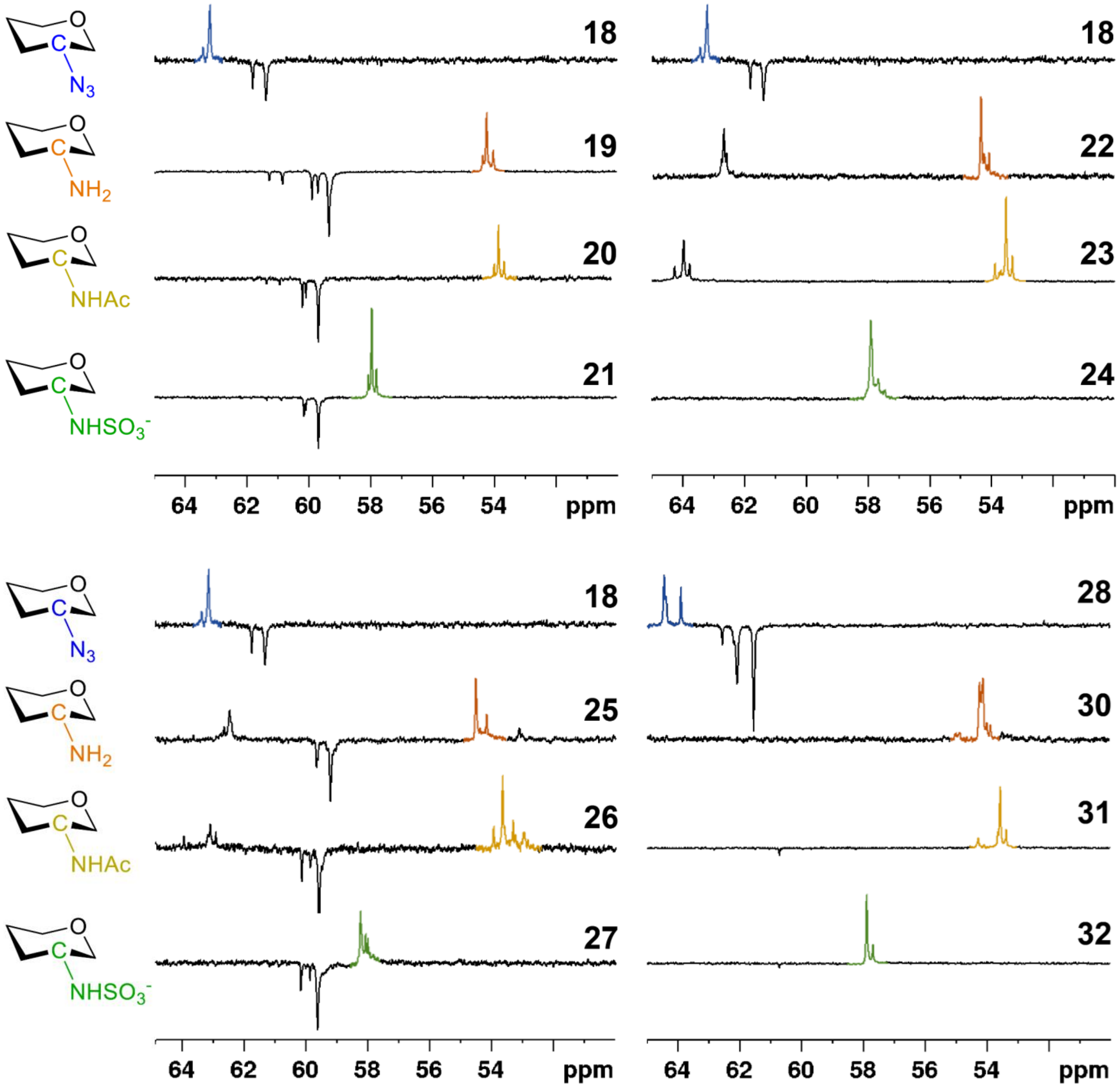
O
C
N3
NH2
NHAc
NHSO3-
18
19
20
21
22
23
24
25
26
27
28
30
31
32
64
62
60
58
56
54
ppm

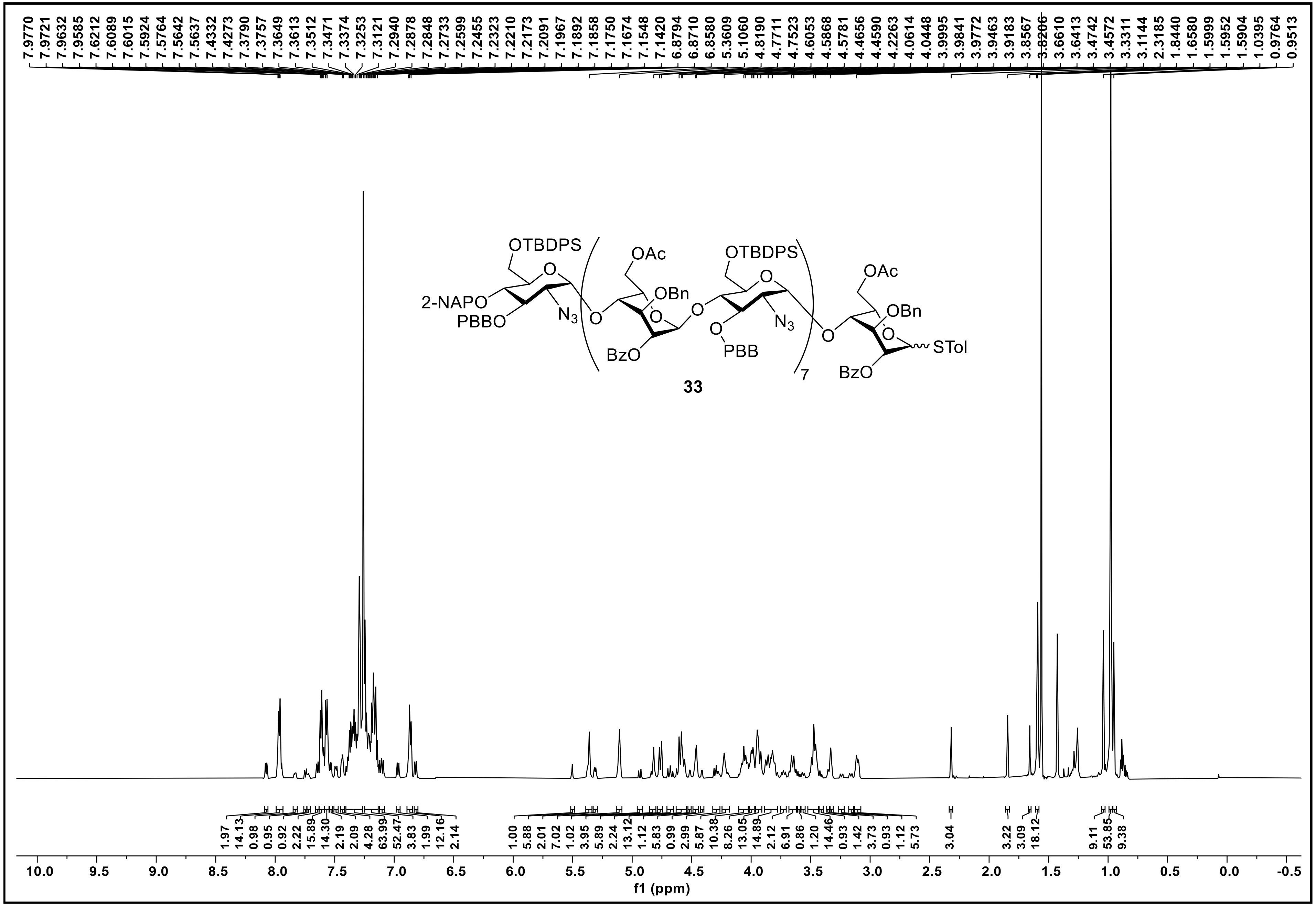
7.9770 7.9721 7.9632 7.9585 7.6212 7.6089 7.6015 7.5924 7.5764 7.5642 7.5637 7.4332 7.4273 7.3790 7.3757 7.3649 7.3613 7.3512 7.3471 7.3374 7.3253 7.3121 7.2940 7.2878 7.2848 7.2733 7.2599 7.2455 7.2323 7.2210 7.2173 7.2091 7.1967 7.1892 7.1858 7.1750 7.1674 7.1548 7.1420 6.8794 6.8710 6.8580 5.3609 5.1060 4.8190 4.7711 4.7523 4.6053 4.5868 4.5781 4.4656 4.4590 4.2263 4.0614 4.0448 3.9995 3.9841 3.9772 3.9463 3.9183 3.8567 3.8206 3.6610 3.6413 3.4742 3.4572 3.3311 3.1144 2.3185 1.8440 1.6580 1.5999 1.5952 1.5904 1.0395 0.9764 0.9513
OTBDPS
2-NAPO
PBBO
N3
OAc
OBn
BzO
O
PBB
7
STol
33
1.97 14.13 0.95 0.92 2.22 15.89 14.30 2.19 2.09 4.28 63.99 52.47 3.83 1.99 12.16 2.14
1.00 5.88 2.01 7.02 1.02 3.95 5.89 2.24 13.12 1.12 5.83 0.99 2.99 5.87 10.38 8.26 13.05 14.89 2.12 6.91 0.86 1.20 14.46 0.93 1.42 3.73 0.93 1.12 5.73
3.04
3.22 3.09 18.12
9.11 53.85 9.38
10.0 9.5 9.0 8.5 8.0 7.5 7.0 6.5 6.0 5.5 5.0 4.5 4.0 3.5 3.0 2.5 2.0 1.5 1.0 0.5 0.0 -0.5
f1 (ppm)

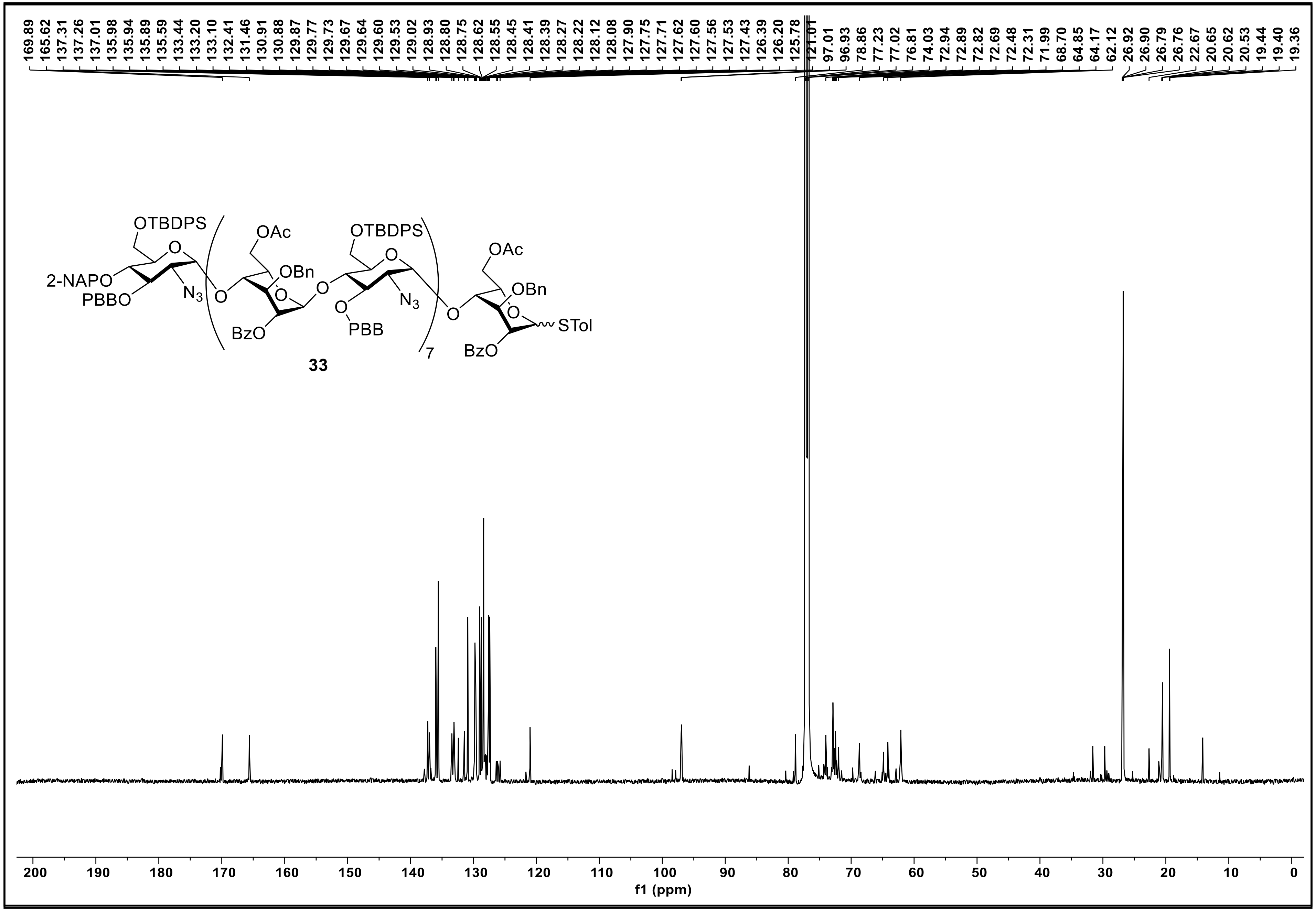

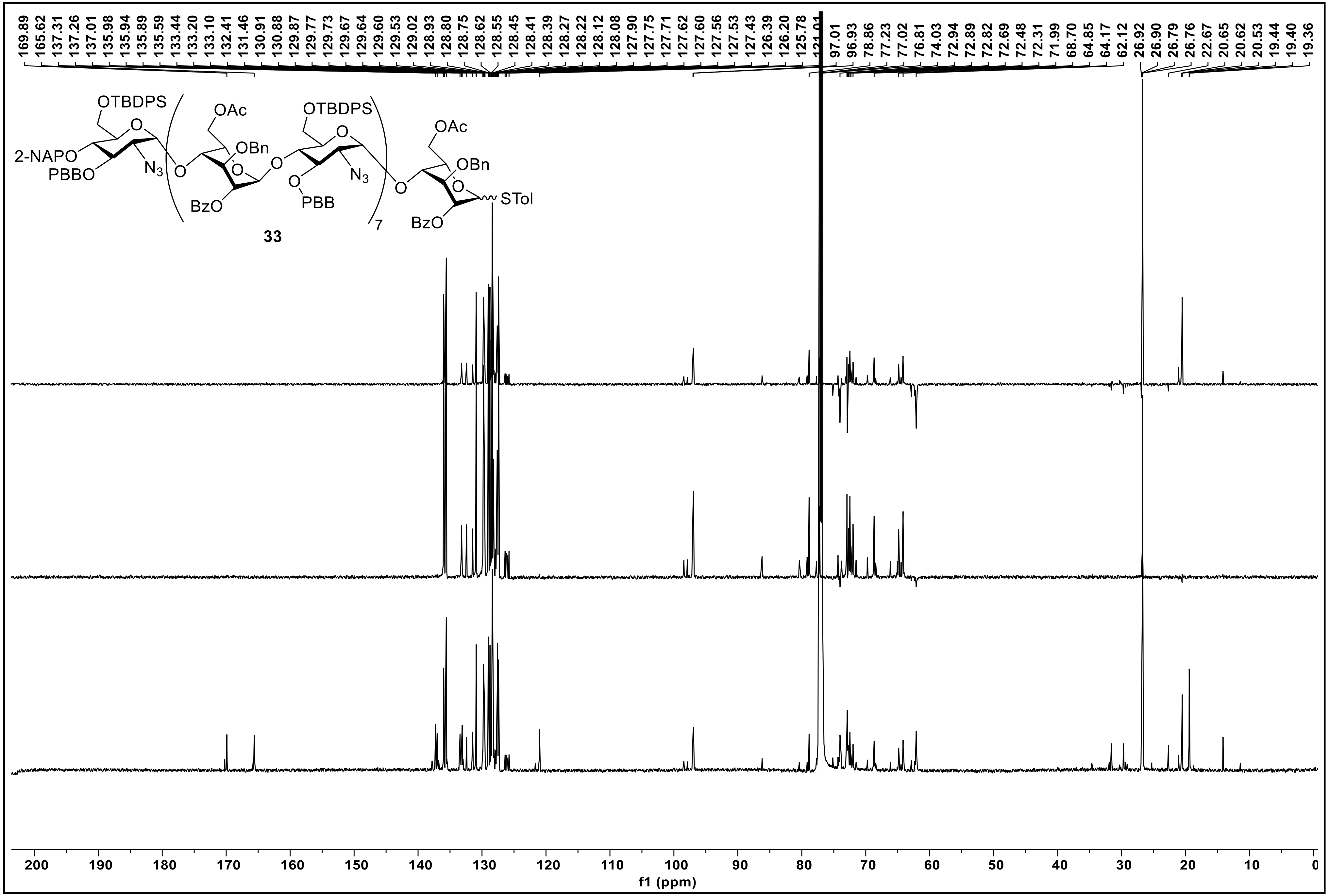
169.89
165.62
137.31
137.26
137.01
135.98
135.94
135.89
135.59
133.44
133.20
133.10
132.41
131.46
130.91
130.88
129.87
129.77
129.73
129.67
129.64
129.60
129.53
129.02
128.93
128.80
128.75
128.62
128.55
128.45
128.41
128.39
128.27
128.22
128.12
128.08
127.90
127.75
127.71
127.62
127.60
127.56
127.53
127.43
126.39
126.20
125.78
121.01
97.01
96.93
78.86
77.23
77.02
76.81
74.03
72.94
72.89
72.82
72.69
72.48
72.31
71.99
68.70
64.85
64.17
62.12
26.92
26.90
26.79
26.76
22.67
20.65
20.62
20.53
19.44
19.40
19.36
OTBDPS
2-NAPO
PBBO
N3
O
OAc
OBn
BzO
PBB
OTBDPS
7
OAc
OBn
STol
BzO
33
f1 (ppm)

HRMS-MALDI

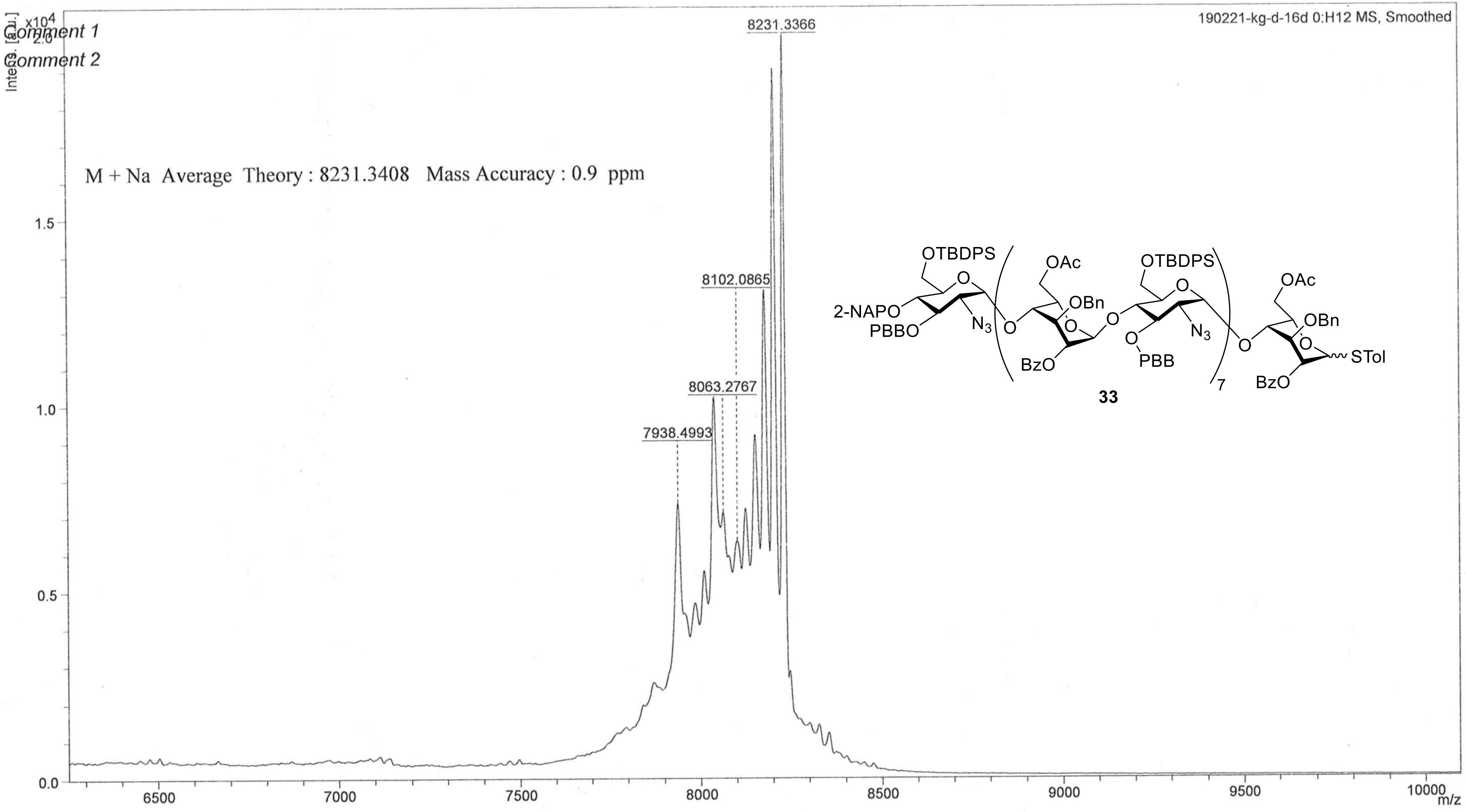

Comment 1
Comment 2
Intens. [a.u.] x10^4
190221-kg-d-16d 0:H12 MS, Smoothed
8231.3366
8102.0865
8063.2767
7938.4993
M + Na Average Theory : 8231.3408 Mass Accuracy : 0.9 ppm
OTBDPS
2-NAPO
PBBO
N3
OAc
OBn
BzO
OTBDPS
PBB
7
OAc
OBn
STol
BzO
33
m/z

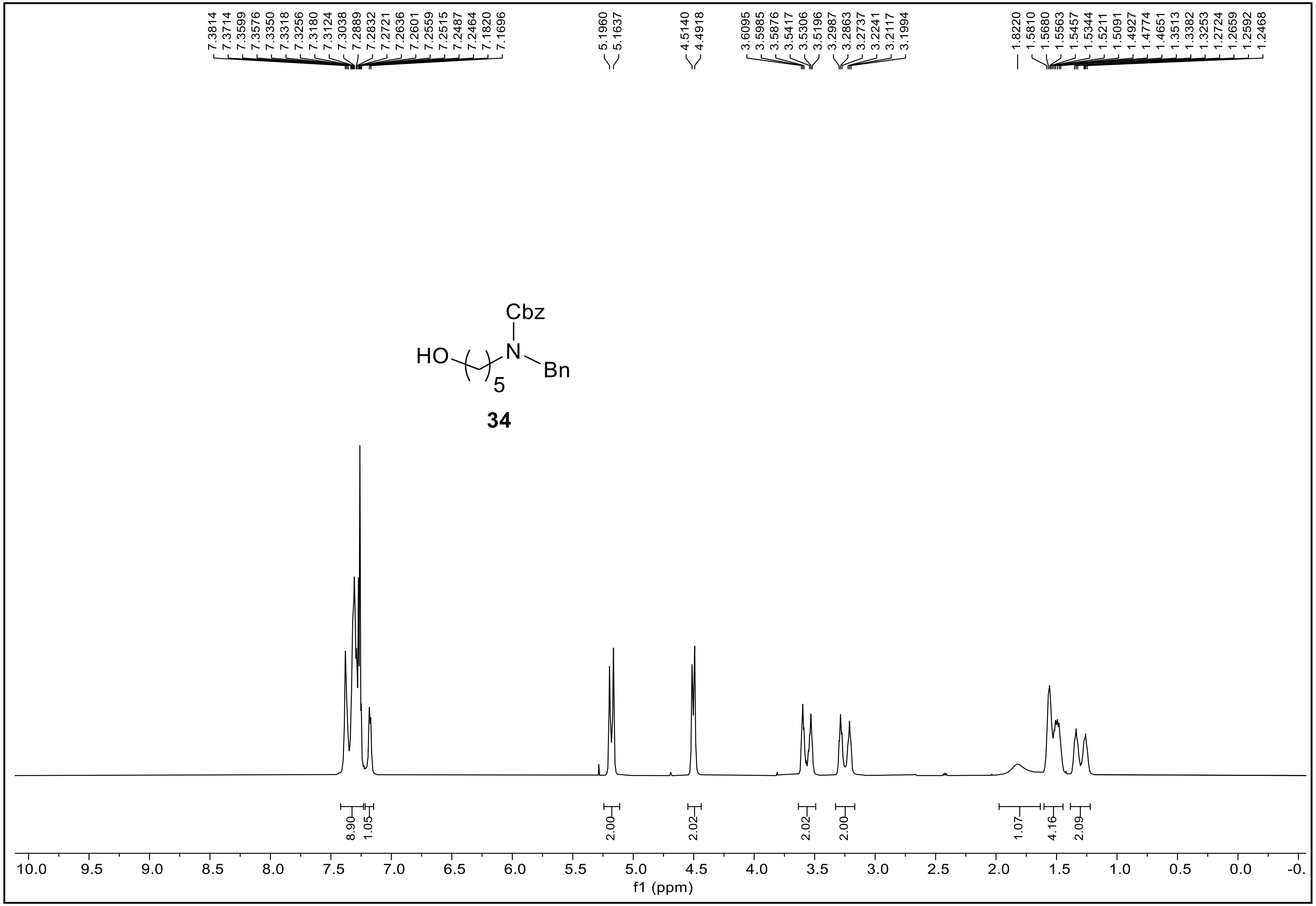

7.3814
7.3714
7.3599
7.3576
7.3350
7.3318
7.3256
7.3180
7.3124
7.3038
7.2889
7.2832
7.2721
7.2636
7.2601
7.2559
7.2515
7.2487
7.2464
7.1820
7.1696
5.1960
5.1637
4.5140
4.4918
3.6095
3.5985
3.5876
3.5417
3.5306
3.5196
3.2987
3.2863
3.2737
3.2241
3.2117
3.1994
1.8220
1.5810
1.5680
1.5563
1.5457
1.5344
1.5211
1.5091
1.4927
1.4774
1.4651
1.3513
1.3382
1.3253
1.2724
1.2659
1.2592
1.2468
Cbz
N
HO
5
Bn
34
8.90
1.05
2.00
2.02
2.02
2.00
1.07
4.16
2.09
f1 (ppm)

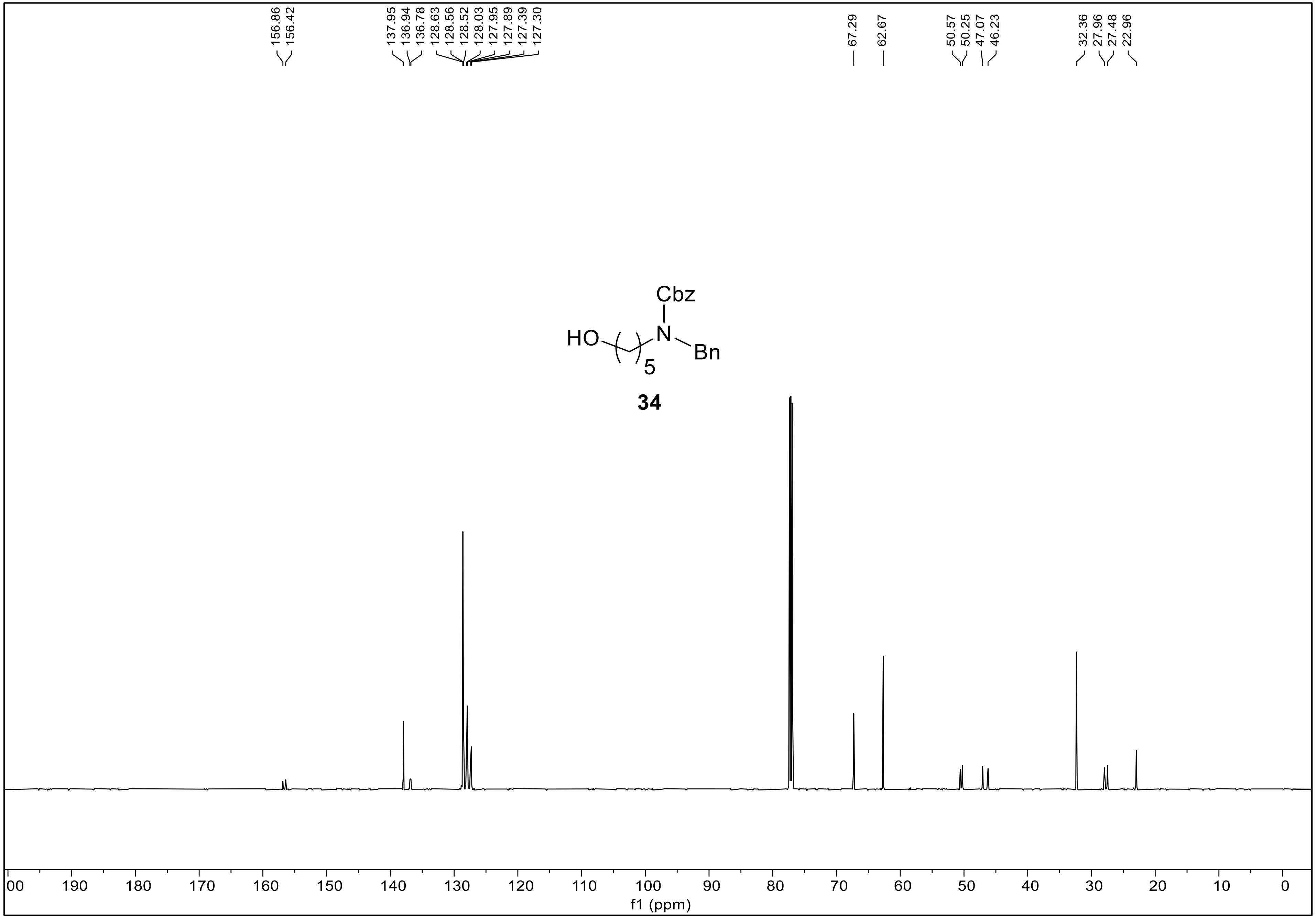
156.86
156.42
137.95
136.94
136.78
128.63
128.56
128.52
128.03
127.95
127.89
127.39
127.30
67.29
62.67
50.57
50.25
47.07
46.23
32.36
27.96
27.48
22.96
Cbz
HO
N
Bn
5
34
f1 (ppm)

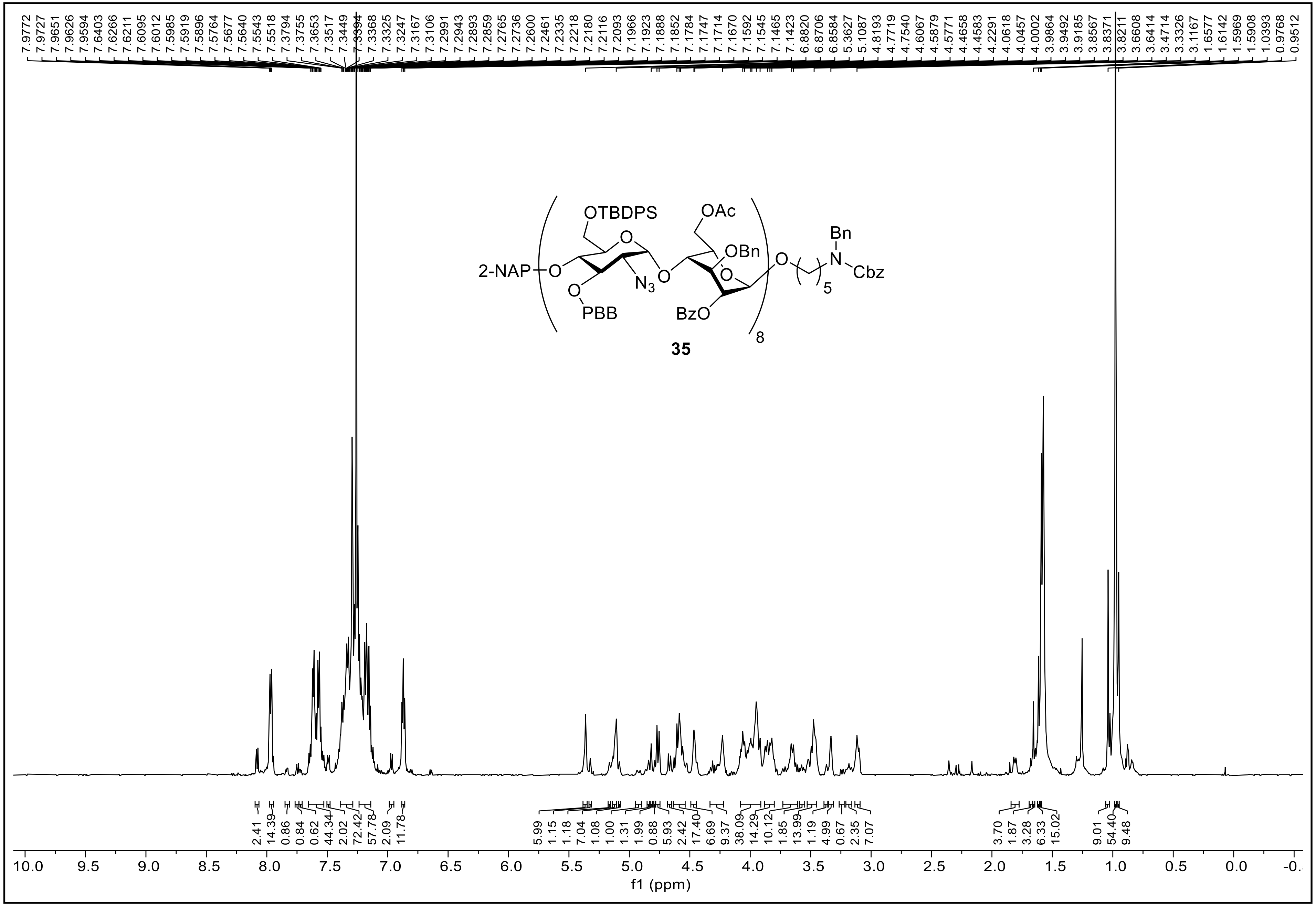
2-NAP
OTBDPS
N3
PBB
OAc
OBn
BzO
Bn
N
Cbz
5
8
35
f1 (ppm)

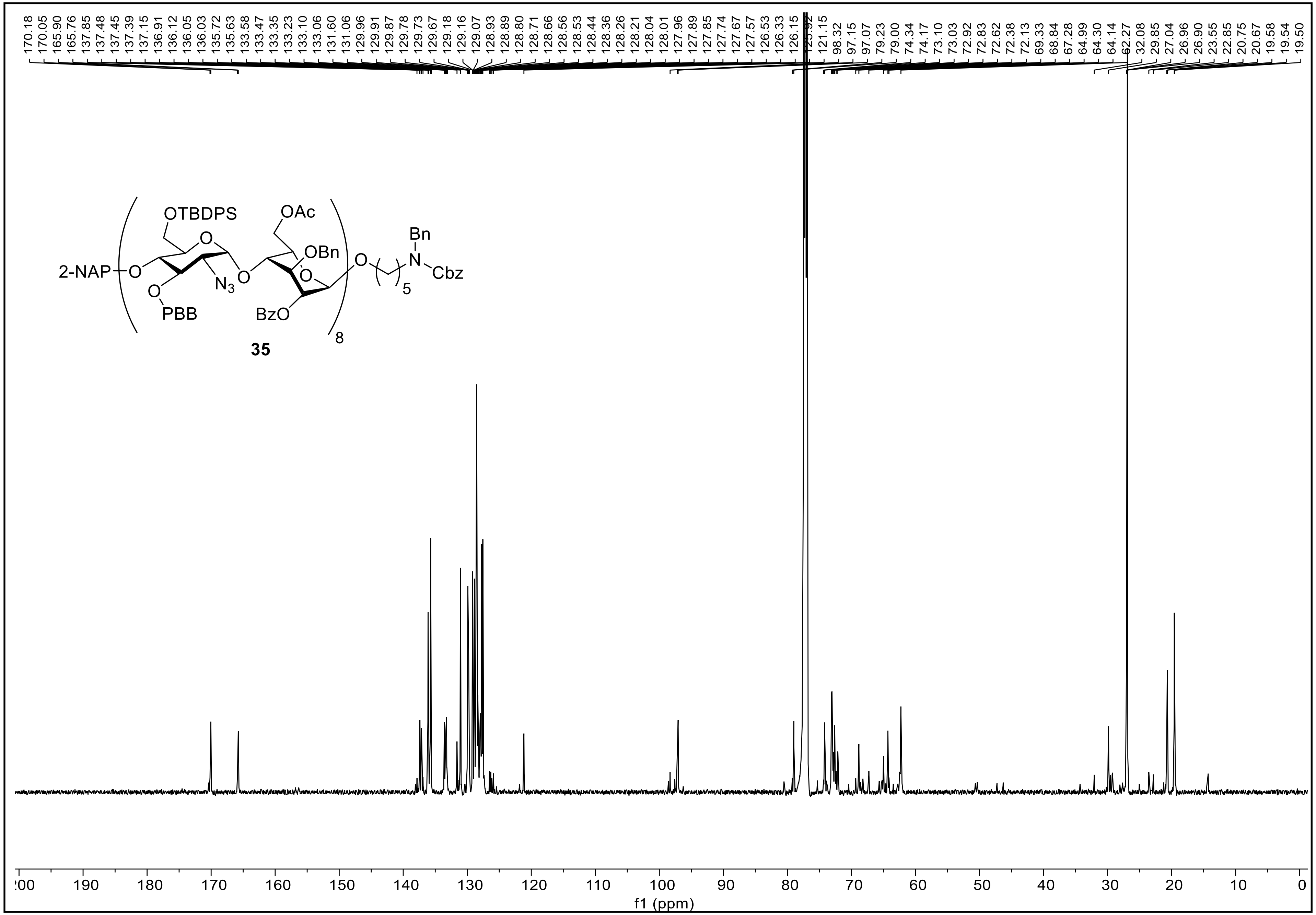
170.18
170.05
165.90
165.76
137.85
137.48
137.45
137.39
137.15
136.91
136.12
136.05
136.03
135.72
135.63
133.58
133.47
133.35
133.23
133.10
133.06
131.60
131.06
129.96
129.91
129.87
129.78
129.73
129.67
129.18
129.16
129.07
128.93
128.89
128.80
128.71
128.66
128.56
128.53
128.44
128.36
128.26
128.21
128.04
128.01
127.96
127.89
127.85
127.74
127.67
127.57
126.53
126.33
126.15
121.15
98.32
97.15
97.07
79.23
79.00
74.34
74.17
73.10
73.03
72.92
72.83
72.62
72.38
72.13
69.33
68.84
67.28
64.99
64.30
64.14
62.27
32.08
29.85
27.04
26.96
26.90
23.55
22.85
20.75
20.67
19.58
19.54
19.50
OTBDPS
OAc
OBn
Bn
2-NAP
O
N
Cbz
N_3
PBB
BzO
5
8
35
f1 (ppm)

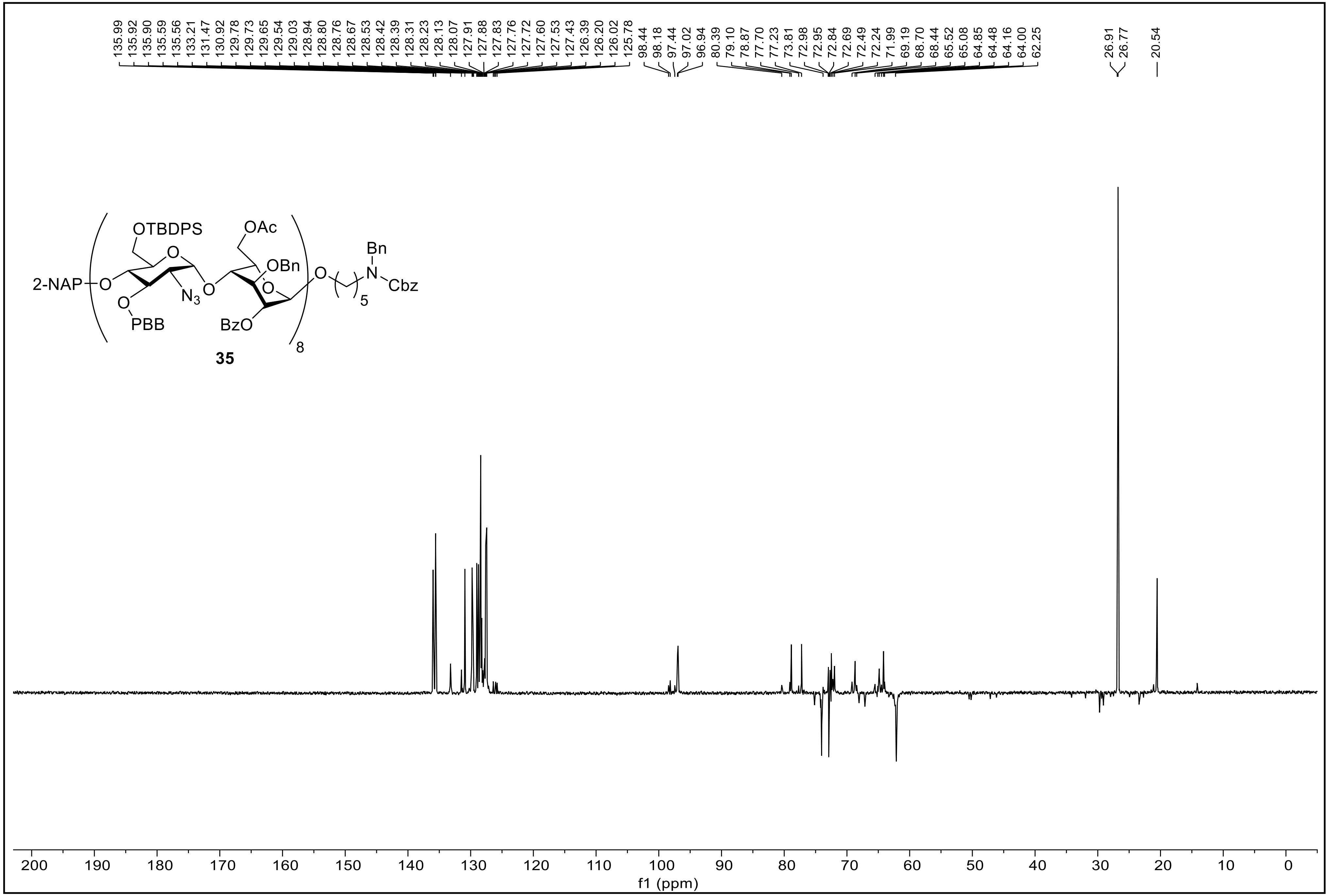
135.99
135.92
135.90
135.59
135.56
133.21
131.47
130.92
129.78
129.73
129.65
129.54
129.03
128.94
128.80
128.76
128.67
128.53
128.42
128.39
128.31
128.23
128.13
128.07
127.91
127.88
127.83
127.76
127.72
127.60
127.53
127.43
126.39
126.20
126.02
125.78
98.44
98.18
97.44
97.02
96.94
80.39
79.10
78.87
77.70
77.23
73.81
72.98
72.95
72.84
72.69
72.49
72.24
71.99
69.19
68.70
68.44
65.52
65.08
64.85
64.48
64.16
64.00
62.25
26.91
26.77
20.54
OTBDPS
OAc
OBn
Bn
2-NAP
O
N
Cbz
N_3
PBB
BzO
8
5
35
f1 (ppm)

HRMS-MALDI

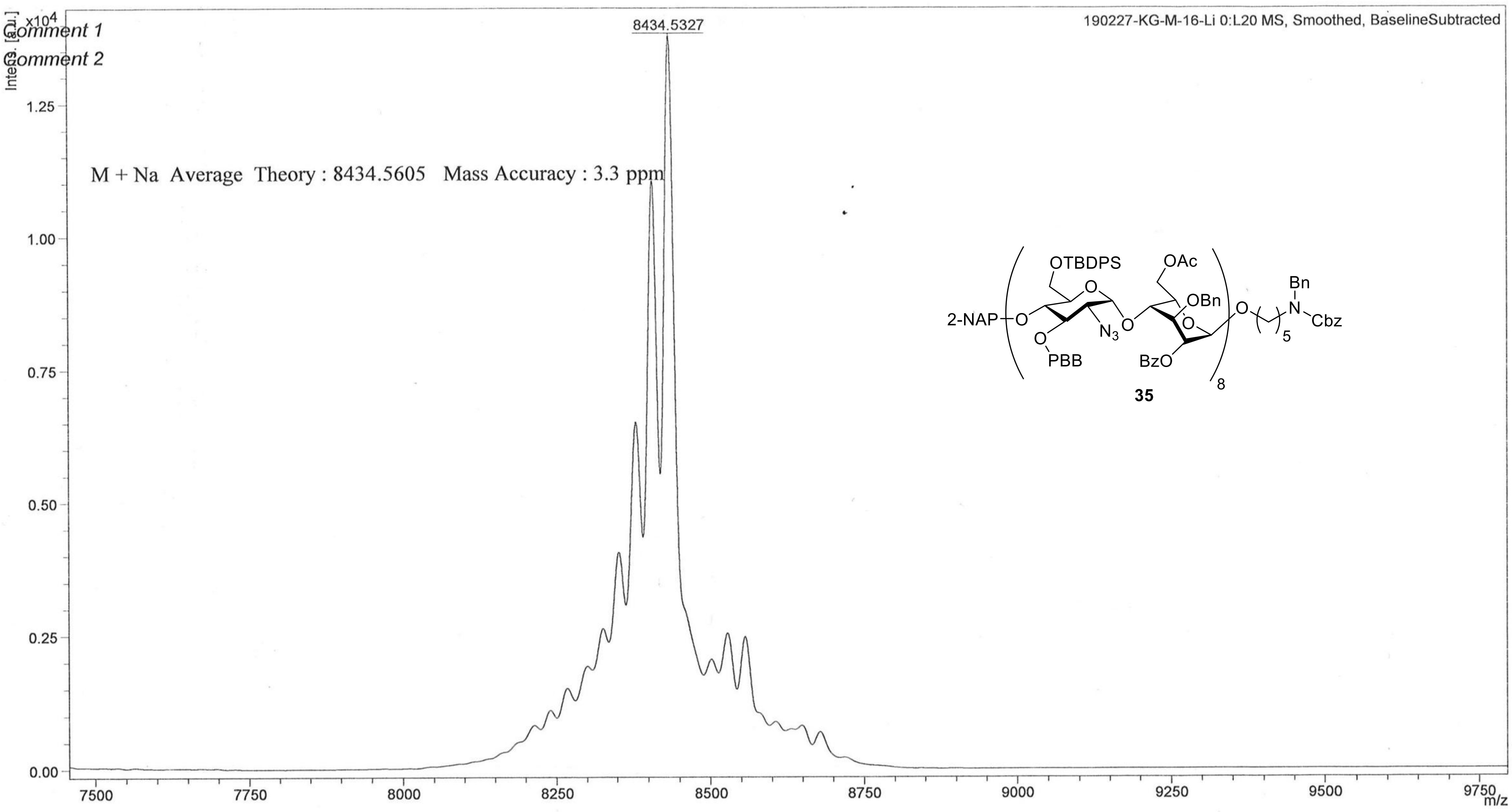

35

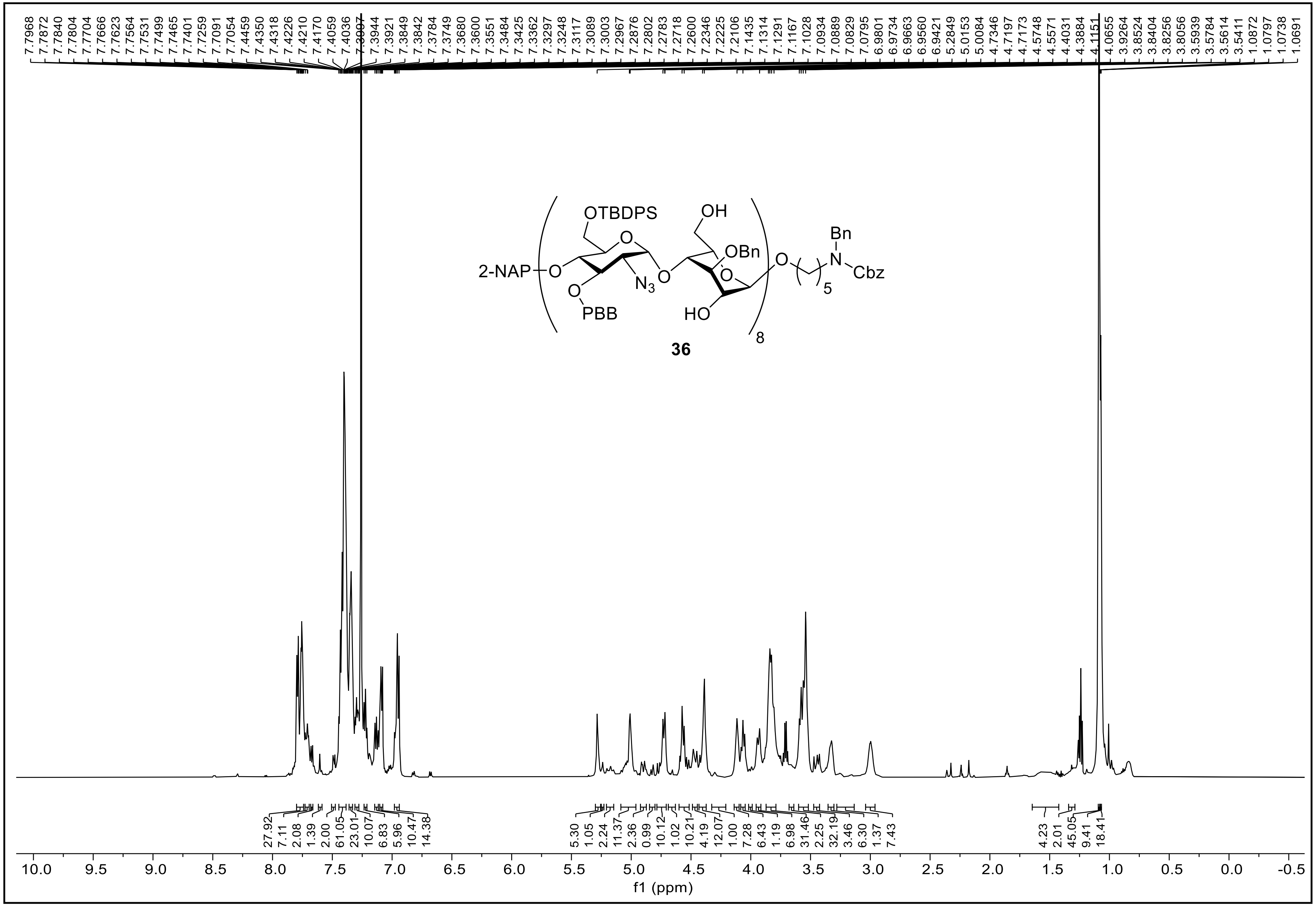

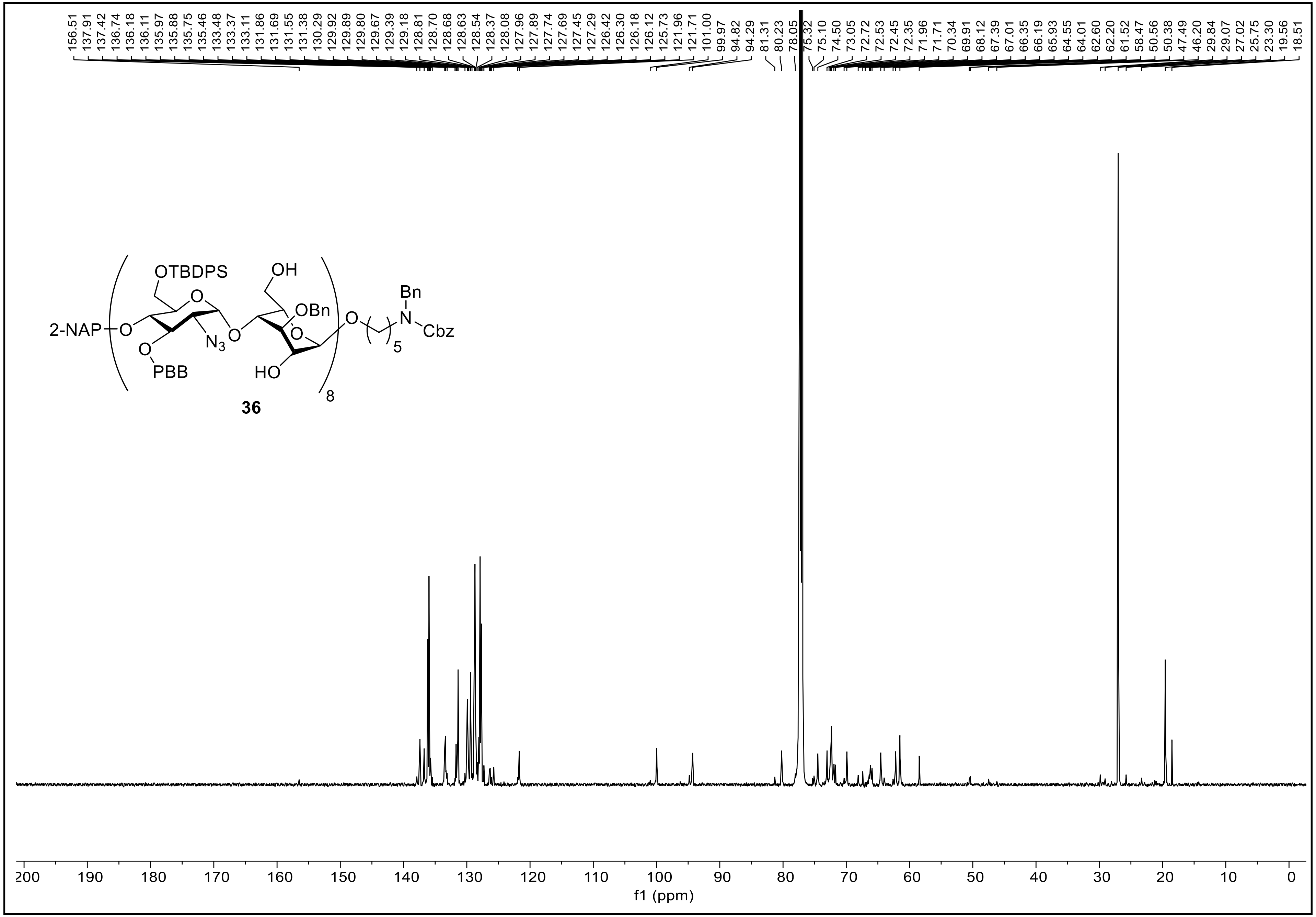
OTBDPS
OH
OBn
2-NAP
O
N3
PBB
HO
Bn
N
Cbz
5
8
36
f1 (ppm)

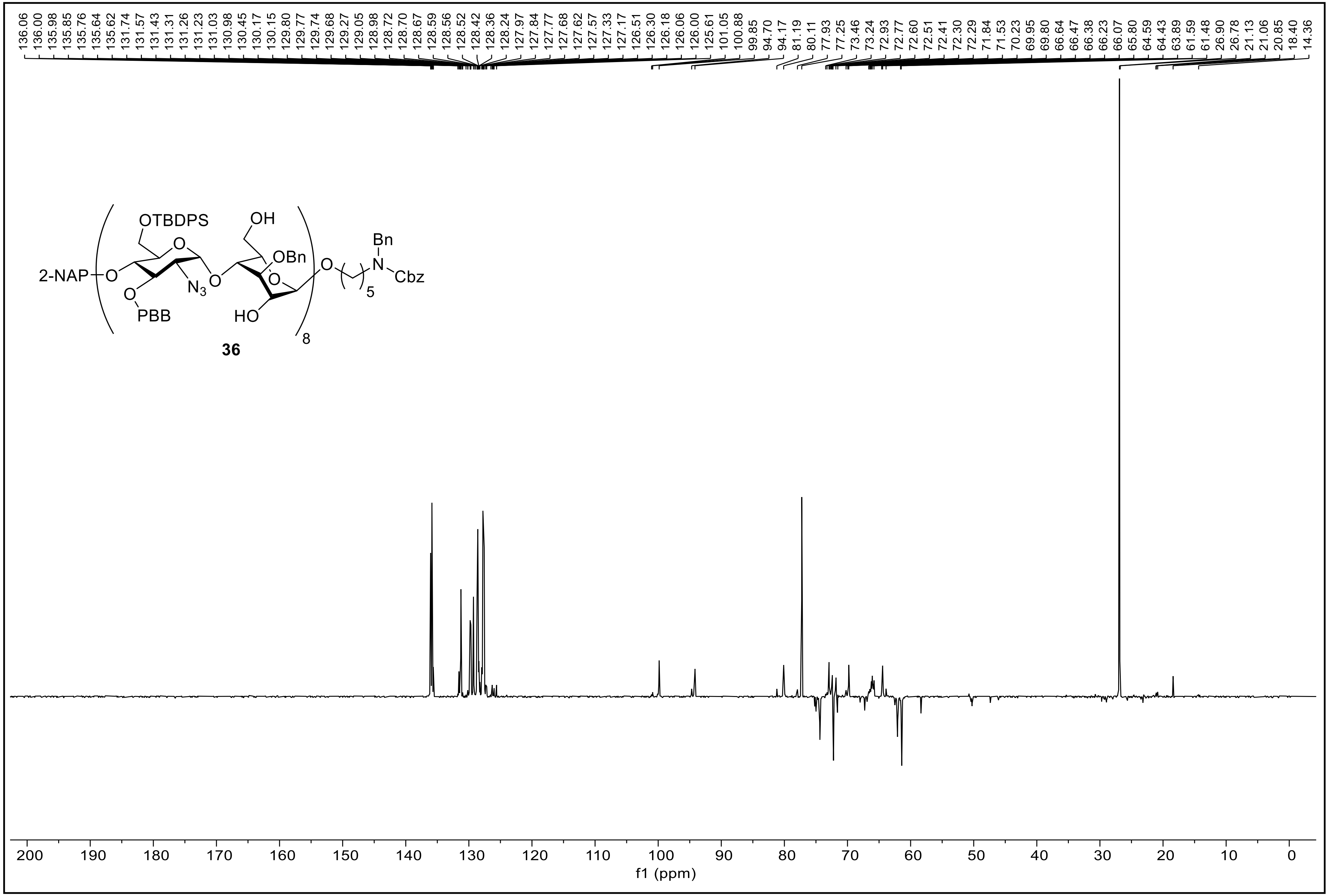
136.06
136.00
135.98
135.85
135.76
135.64
135.62
131.74
131.57
131.43
131.31
131.26
131.23
131.03
130.98
130.45
130.17
130.15
129.80
129.77
129.74
129.68
129.27
129.05
128.98
128.72
128.70
128.67
128.59
128.56
128.52
128.42
128.36
128.24
127.97
127.84
127.77
127.68
127.62
127.57
127.33
127.17
126.51
126.30
126.18
126.06
126.00
125.61
101.05
100.88
99.85
94.70
94.17
81.19
80.11
77.93
77.25
73.46
73.24
72.93
72.77
72.60
72.51
72.41
72.30
72.29
71.84
71.53
70.23
69.95
69.80
66.64
66.47
66.38
66.23
66.07
65.80
64.59
64.43
63.89
61.59
61.48
26.90
26.78
21.13
21.06
20.85
18.40
14.36
OTBDPS
OH
OBn
Bn
2-NAP
O
N
Cbz
N3
5
PBB
HO
8
36
200 190 180 170 160 150 140 130 120 110 100 90 80 70 60 50 40 30 20 10 0
f1 (ppm)

HRMS-MALDI

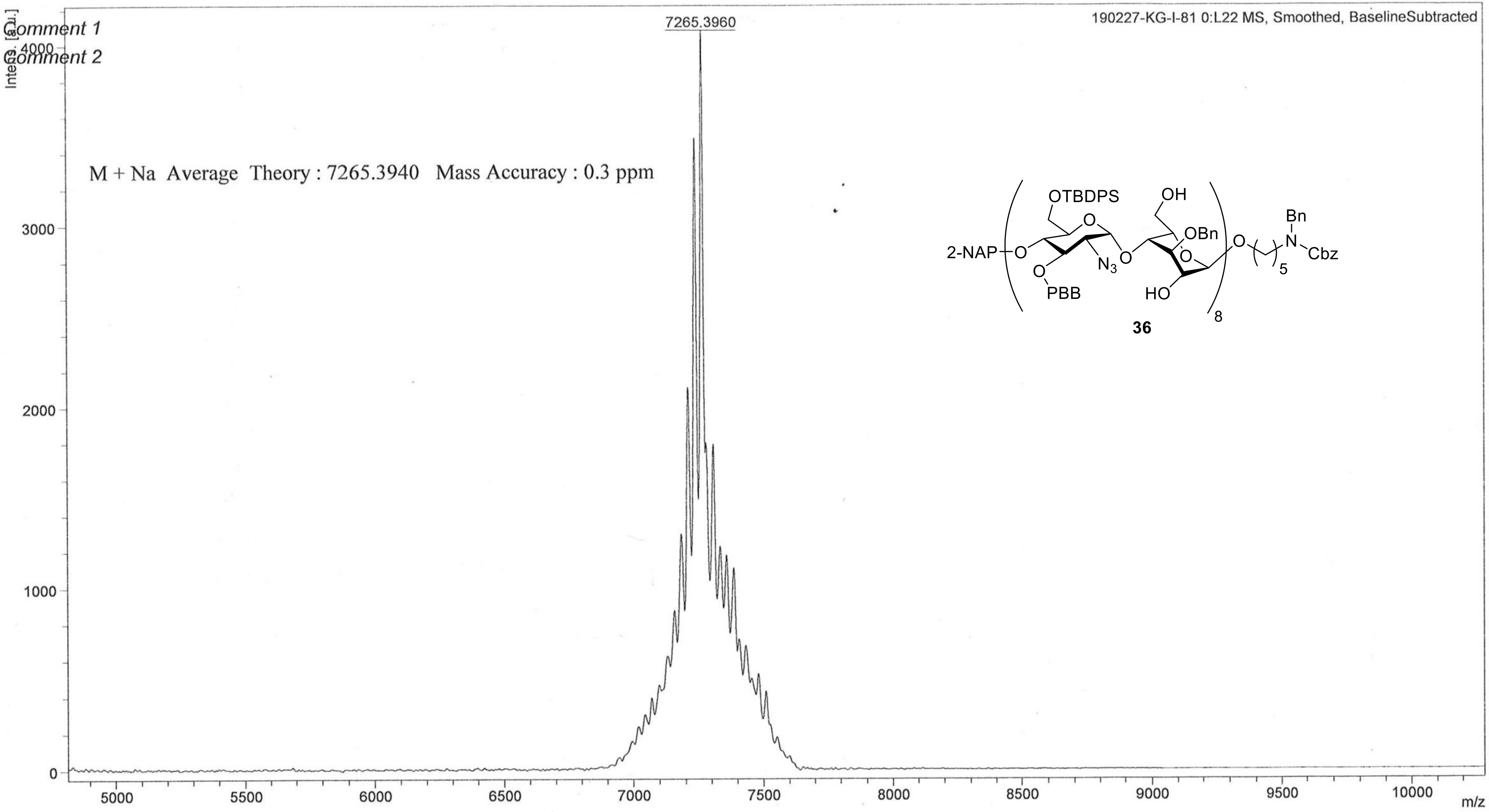
190227-KG-I-81 0:L22 MS, Smoothed, BaselineSubtracted
Comment 1
Comment 2
Intens. [a.u.]
7265.3960
M + Na Average Theory : 7265.3940 Mass Accuracy : 0.3 ppm
OTBDPS
OH
OBn
2-NAP
N3
PBB
HO
Bn
N
Cbz
5
8
36
m/z

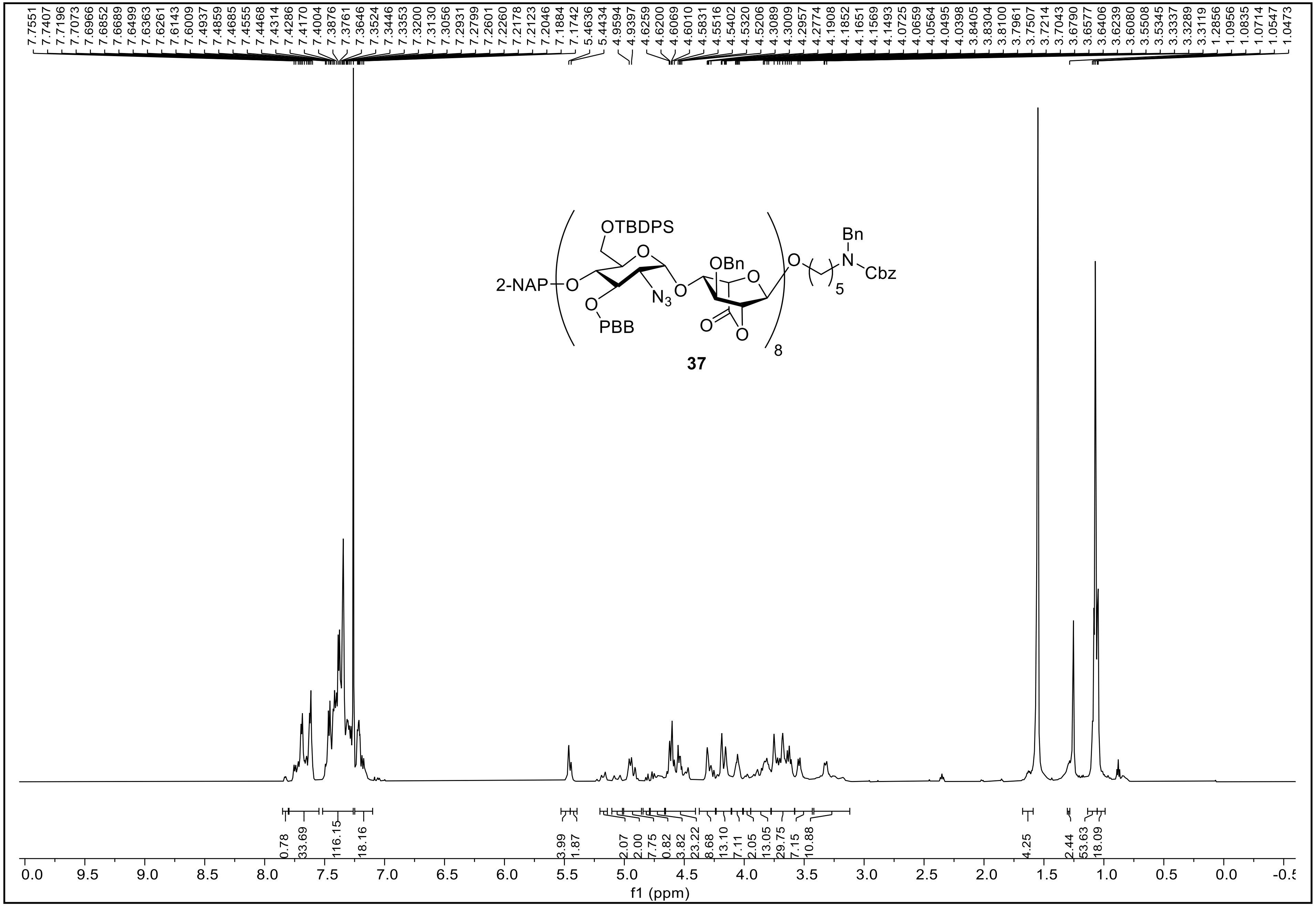
OTBDPS
2-NAP
O
N3
PBB
OBn
Bn
N
Cbz
5
8
37
f1 (ppm)

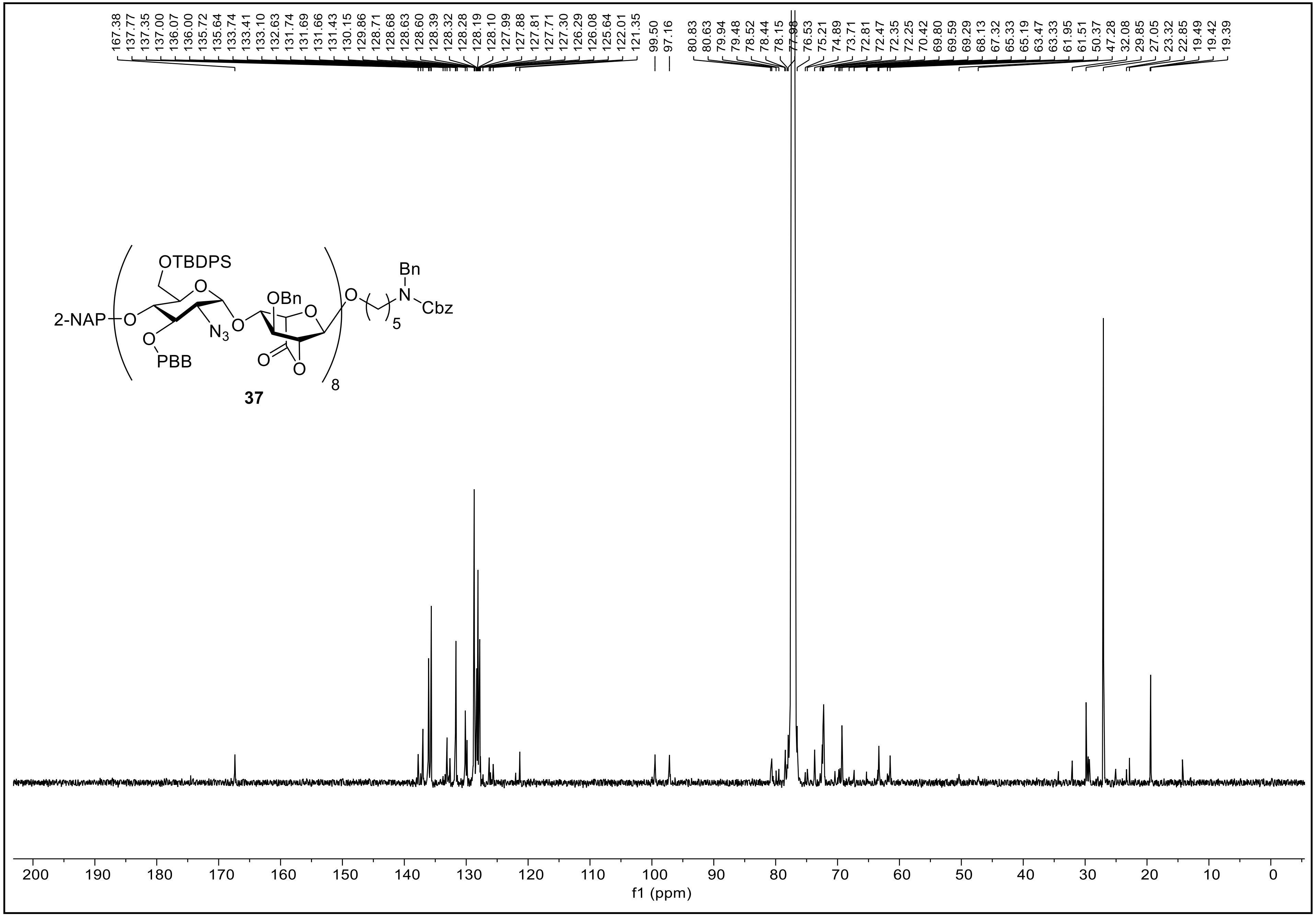

167.38
137.77
137.35
137.00
136.07
136.00
135.72
135.64
133.74
133.41
133.10
132.63
131.74
131.69
131.66
131.43
130.15
129.86
128.71
128.68
128.63
128.60
128.39
128.32
128.28
128.19
128.10
127.99
127.88
127.81
127.71
127.30
126.29
126.08
125.64
122.01
121.35
99.50
97.16
80.83
80.63
79.94
79.48
78.52
78.44
78.15
77.98
76.53
75.21
74.89
73.71
72.81
72.47
72.35
72.25
70.42
69.80
69.59
69.29
68.13
67.32
65.33
65.19
63.47
63.33
61.95
61.51
50.37
47.28
32.08
29.85
27.05
23.32
22.85
19.49
19.42
19.39
OTBDPS
O
OBn
O
Bn
N
Cbz
2-NAP
O
O
N3
O
5
O
PBB
O
O
8
200
190
180
170
160
150
140
130
120
110
100
90
80
70
60
50
40
30
20
10
0
f1 (ppm)

37

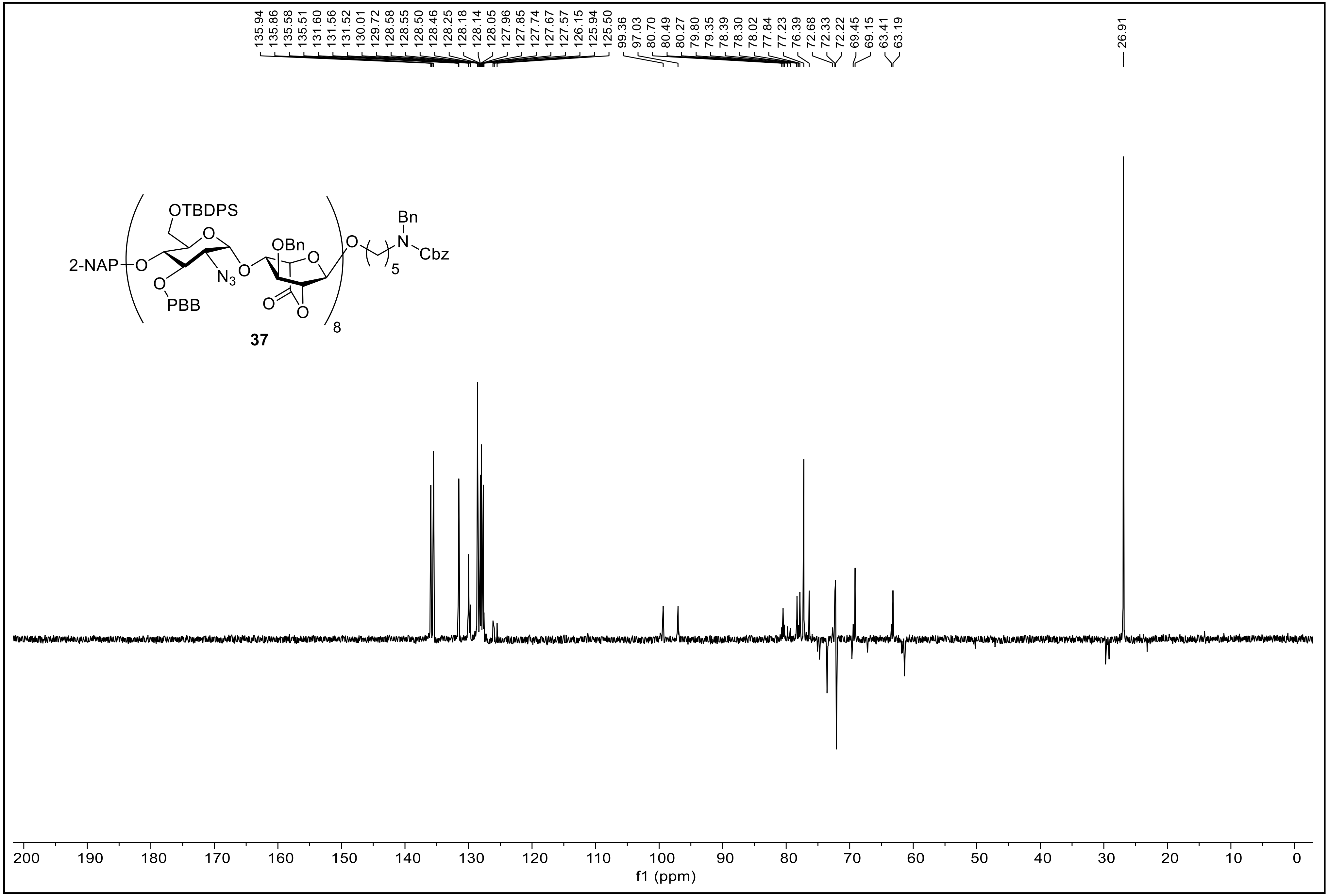

135.94
135.86
135.58
135.51
131.60
131.56
131.52
130.01
129.72
128.58
128.55
128.50
128.46
128.25
128.18
128.14
128.05
127.96
127.85
127.74
127.67
127.57
126.15
125.94
125.50
99.36
97.03
80.70
80.49
80.27
79.80
79.35
78.39
78.30
78.02
77.84
77.23
76.39
72.68
72.33
72.22
69.45
69.15
63.41
63.19
26.91
OTBDPS
2-NAP
PBB
N3
OBn
Bn
N
Cbz
5
8
37
f1 (ppm)

HRMS-MALDI

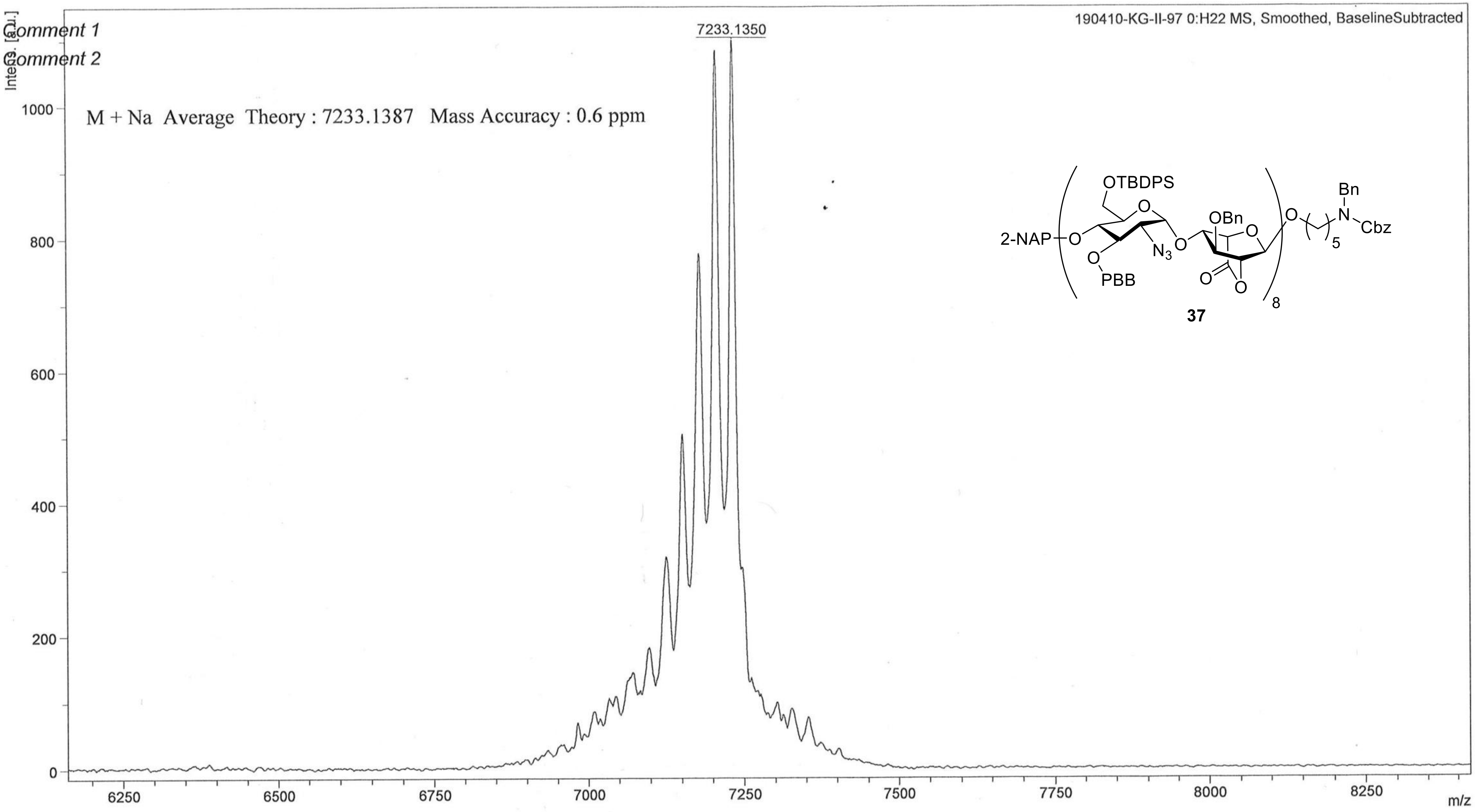

Comment 1
Comment 2
Intens. [a.u.]
7233.1350
190410-KG-II-97 0:H22 MS, Smoothed, BaselineSubtracted
M + Na Average Theory : 7233.1387 Mass Accuracy : 0.6 ppm
OTBDPS
OBn
Bn
2-NAP
N
Cbz
N_3
PBB
5
8
37
m/z

FTIR

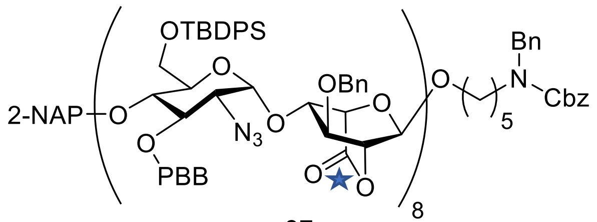
OTBDPS
2-NAP
O
PBB
N3
OBn
Bn
N
Cbz
5
8

37

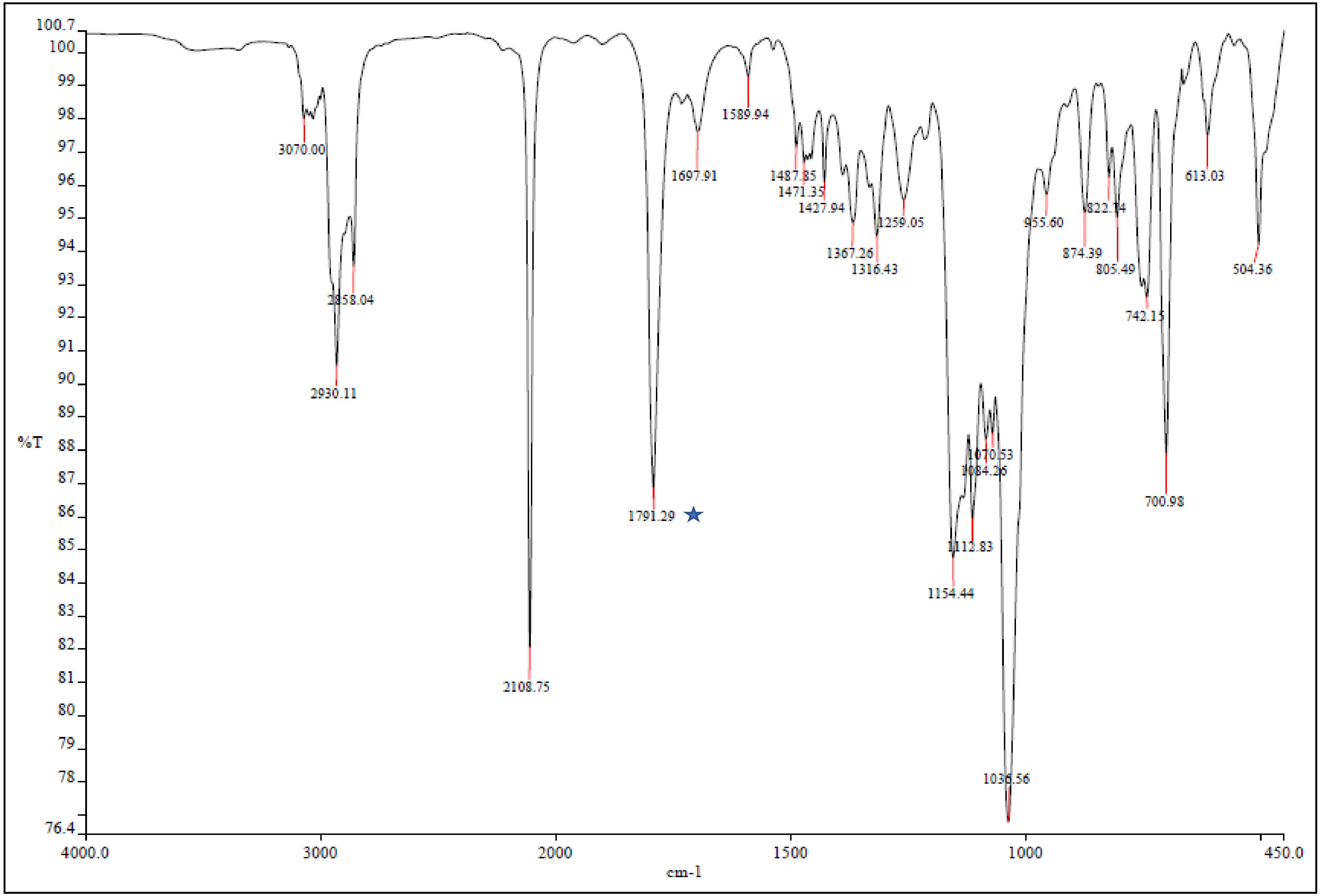
%T
cm-1
3070.00
2930.11
2858.04
2108.75
1791.29
1697.91
1589.94
1487.85
1471.35
1427.94
1367.26
1316.43
1259.05
1154.44
1112.83
1084.26
1070.53
1036.56
955.60
874.39
822.74
805.49
742.15
700.98
613.03
504.36
4000.0
3000
2000
1500
1000
450.0

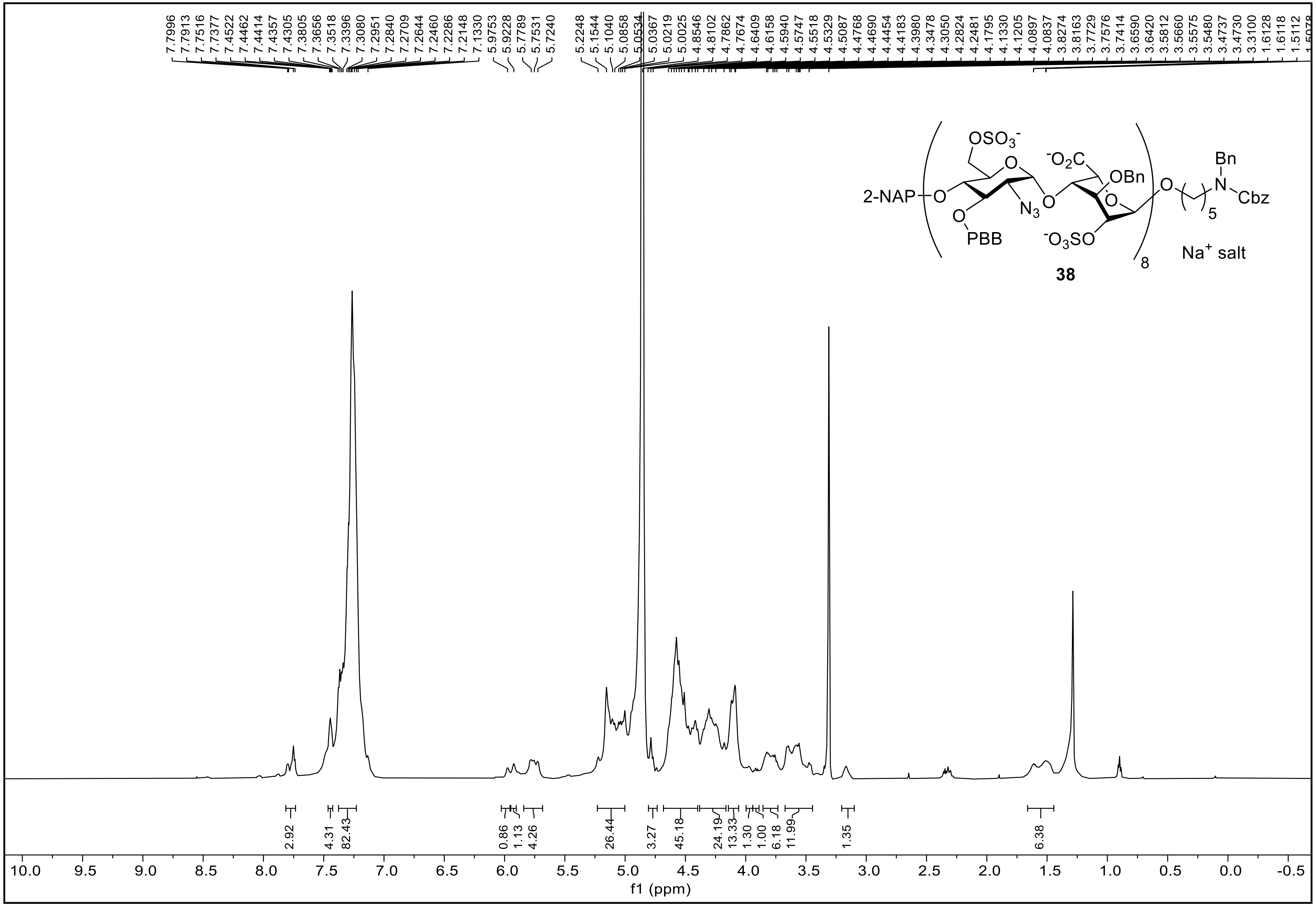

2-NAP
OSO3-
-O2C
OBn
N3
PBB
-O3SO
Bn
N
Cbz
5
8
Na+ salt
38
f1 (ppm)

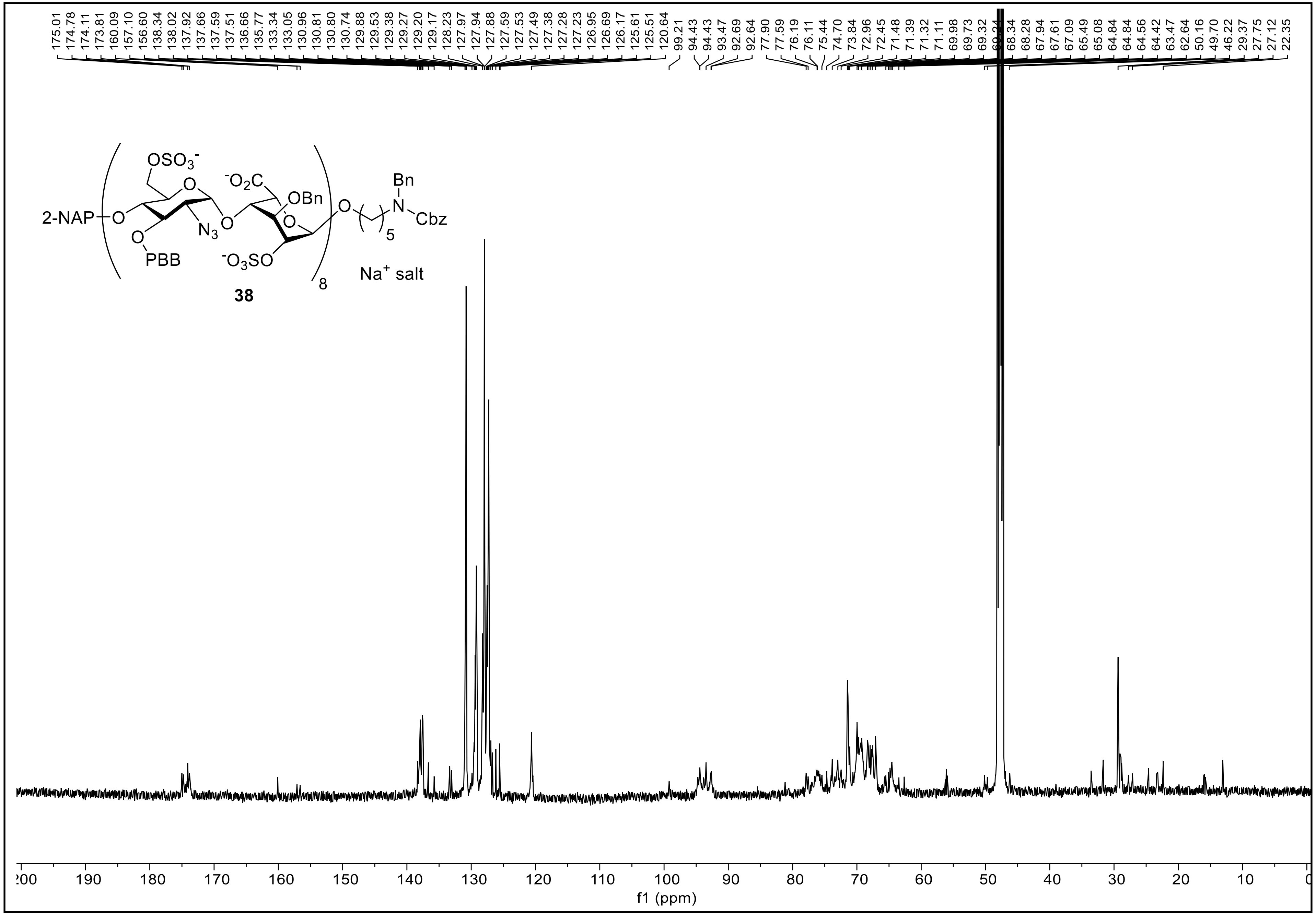
175.01
174.78
174.11
173.81
160.09
157.10
156.60
138.34
138.02
137.92
137.66
137.59
137.51
136.66
135.77
133.34
133.05
130.96
130.81
130.80
130.74
129.88
129.53
129.38
129.27
129.20
129.17
128.23
127.97
127.94
127.88
127.59
127.53
127.49
127.38
127.28
127.23
126.95
126.69
126.17
125.61
125.51
120.64
99.21
94.43
94.43
93.47
92.69
92.64
77.90
77.59
76.19
76.11
75.44
74.70
73.84
72.96
72.45
71.48
71.39
71.32
71.11
69.98
69.73
69.32
68.34
68.28
67.94
67.61
67.09
65.49
65.08
64.84
64.84
64.56
64.42
63.47
62.64
50.16
49.70
46.22
29.37
27.75
27.12
22.35
OSO3-
-O2C
OBn
Bn
N
Cbz
2-NAP
O
N3
PBB
-O3SO
8
5
Na+ salt
38
200
190
180
170
160
150
140
130
120
110
100
90
80
70
60
50
40
30
20
10
0
f1 (ppm)

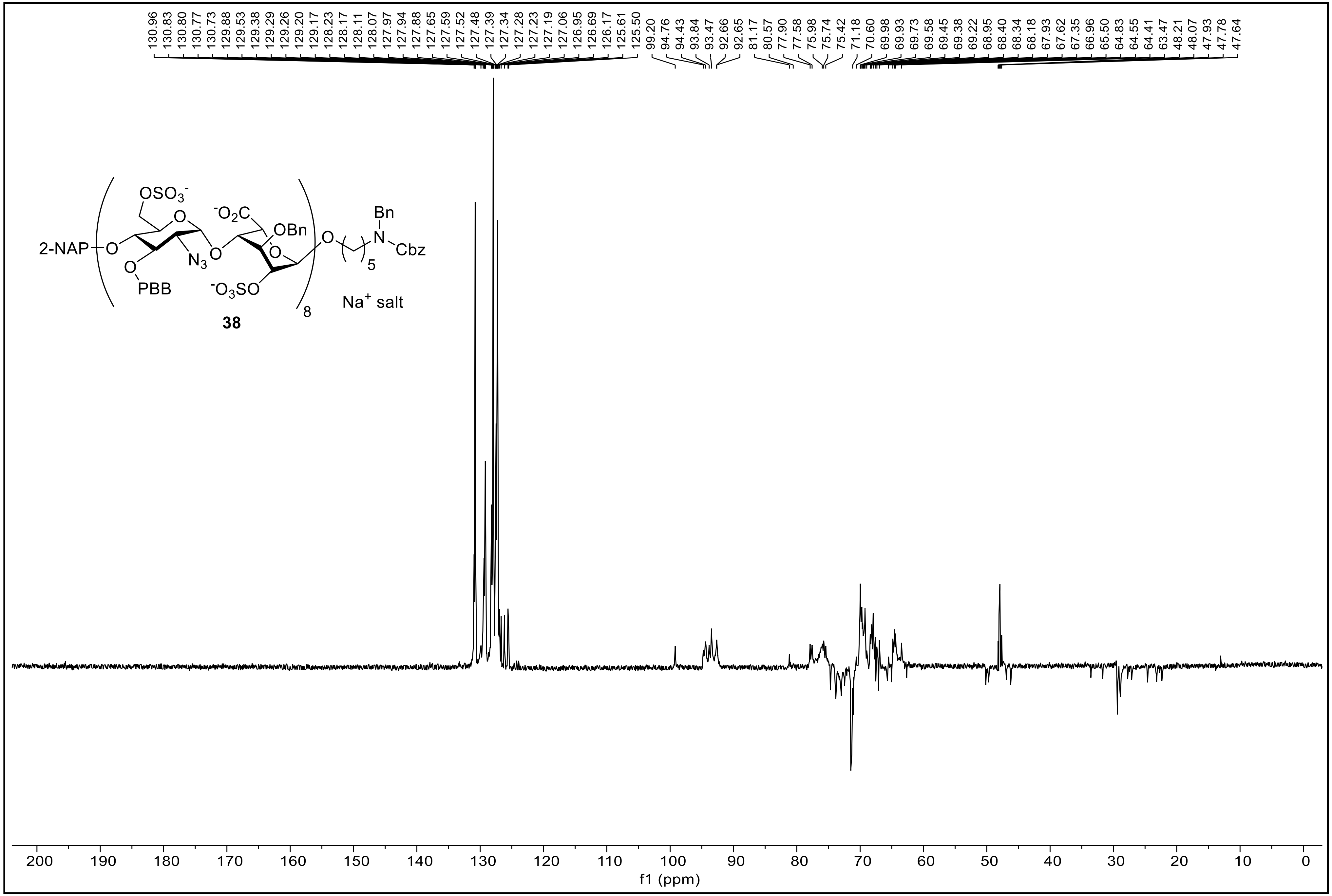

2-NAP
OSO3-
-O2C
OBn
N3
PBB
-O3SO
8
5
Bn
N
Cbz
Na+ salt
38
f1 (ppm)

HRMS-ESI

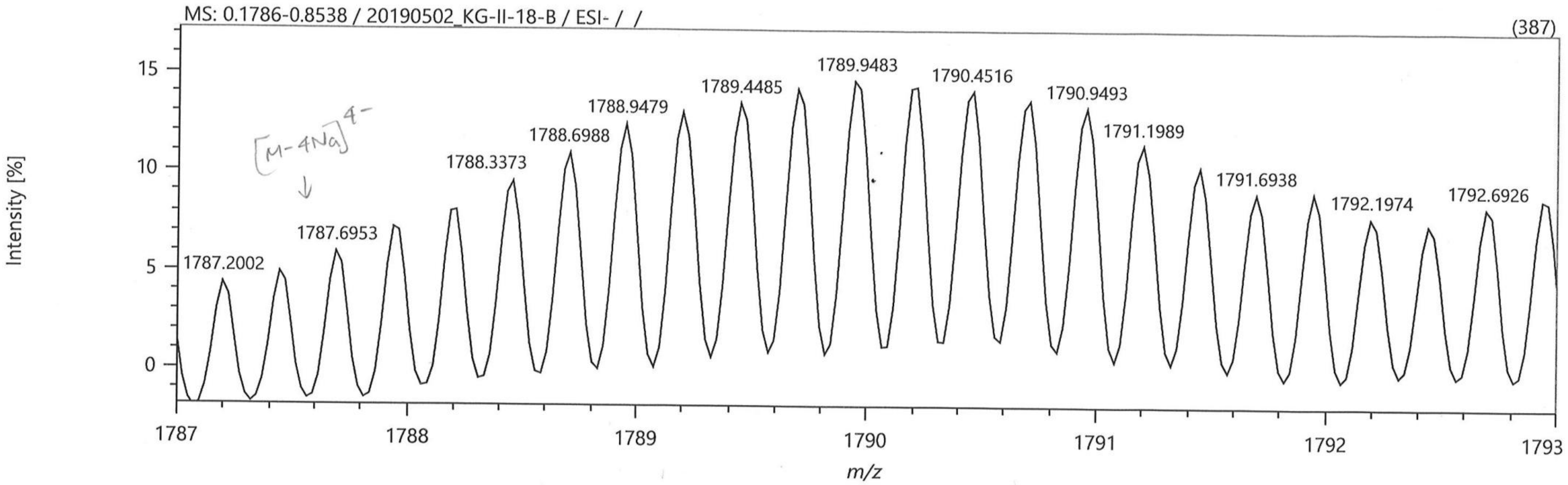

Elemental Composition

Parameters

Tolerance:	±100.00 ppm
Electron:	Odd/Even
Charge:	-4
DBE:	-1.5 - 999.0

Elements Set 1:

Symbol	C	H	O	Na	Br	S	N
Min	239	230	131	20	8	16	25
Max	239	400	131	24	8	16	25

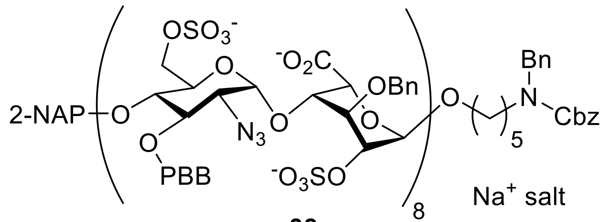

38

Results

Mass	Formula	Calculated Mass	Mass Difference [mDa]	Mass Difference [ppm]	DBE
1787.69534	C239 H233 N25 O131 Na20 S16 Br8	1787.73283	-37.49	-20.97	122.0

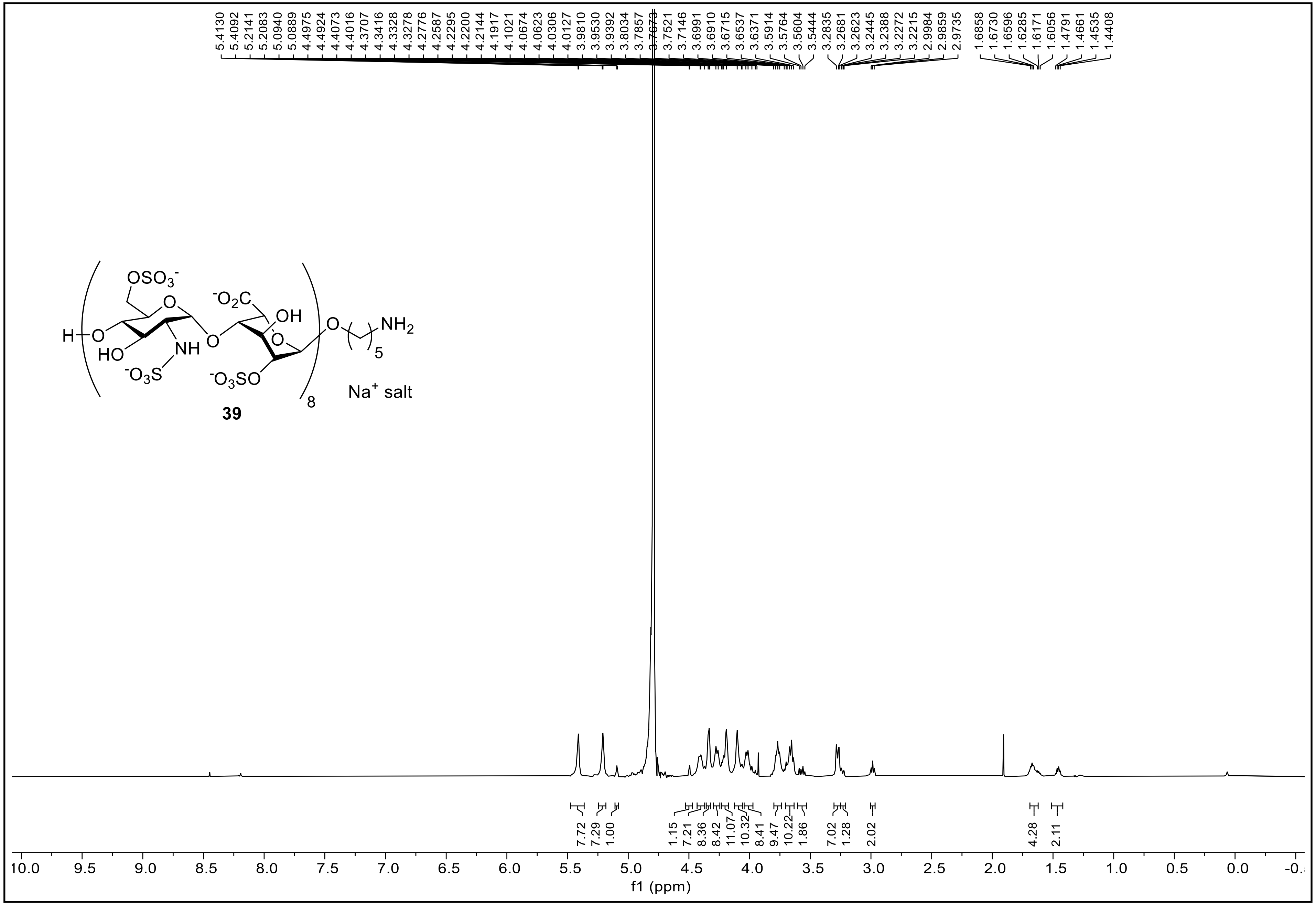
5.4130
5.4092
5.2141
5.2083
5.0940
5.0889
4.4975
4.4924
4.4073
4.4016
4.3707
4.3416
4.3328
4.3278
4.2776
4.2587
4.2295
4.2200
4.2144
4.1917
4.1021
4.0674
4.0623
4.0306
4.0127
3.9810
3.9530
3.9392
3.8034
3.7857
3.7673
3.7521
3.7146
3.6991
3.6910
3.6715
3.6537
3.6371
3.5914
3.5764
3.5604
3.5444
3.2835
3.2681
3.2623
3.2445
3.2388
3.2272
3.2215
2.9984
2.9859
2.9735
1.6858
1.6730
1.6596
1.6285
1.6171
1.6056
1.4791
1.4661
1.4535
1.4408
OSO3-
-O2C
OH
H
O
O
O
O
NH2
HO
NH
5
-O3S
-O3SO
8
Na+ salt
39
7.72
7.29
1.00
1.15
7.21
8.36
8.42
11.07
10.32
8.41
9.47
10.22
1.86
7.02
1.28
2.02
4.28
2.11
10.0
9.5
9.0
8.5
8.0
7.5
7.0
6.5
6.0
5.5
5.0
4.5
4.0
3.5
3.0
2.5
2.0
1.5
1.0
0.5
0.0
f1 (ppm)

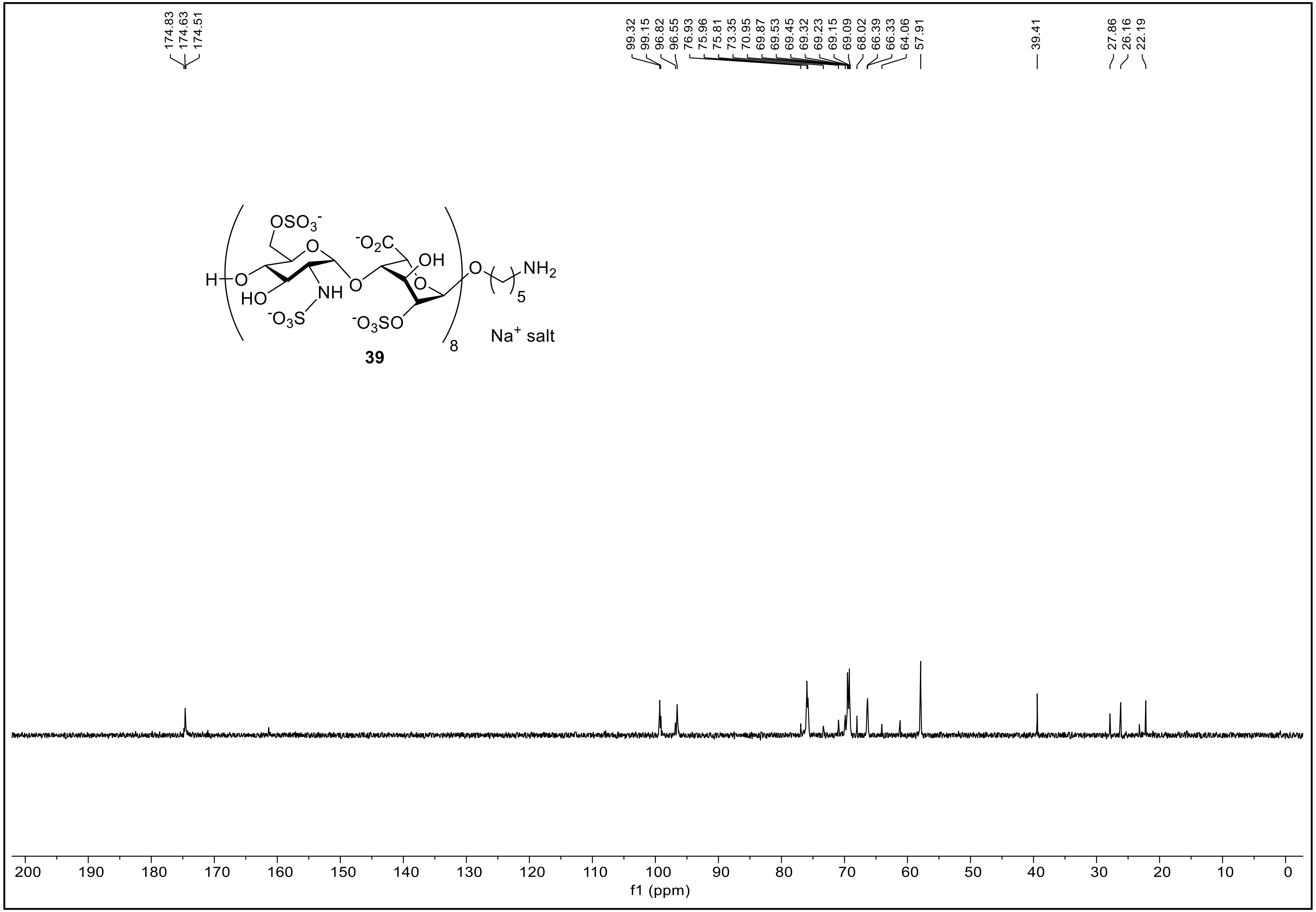
174.83
174.63
174.51
99.32
99.15
96.82
96.55
76.93
75.96
75.81
73.35
70.95
69.87
69.53
69.45
69.32
69.23
69.15
69.09
68.02
66.39
66.33
64.06
57.91
39.41
27.86
26.16
22.19
Na+ salt
39
200
190
180
170
160
150
140
130
120
110
100
90
80
70
60
50
40
30
20
10
0
f1 (ppm)

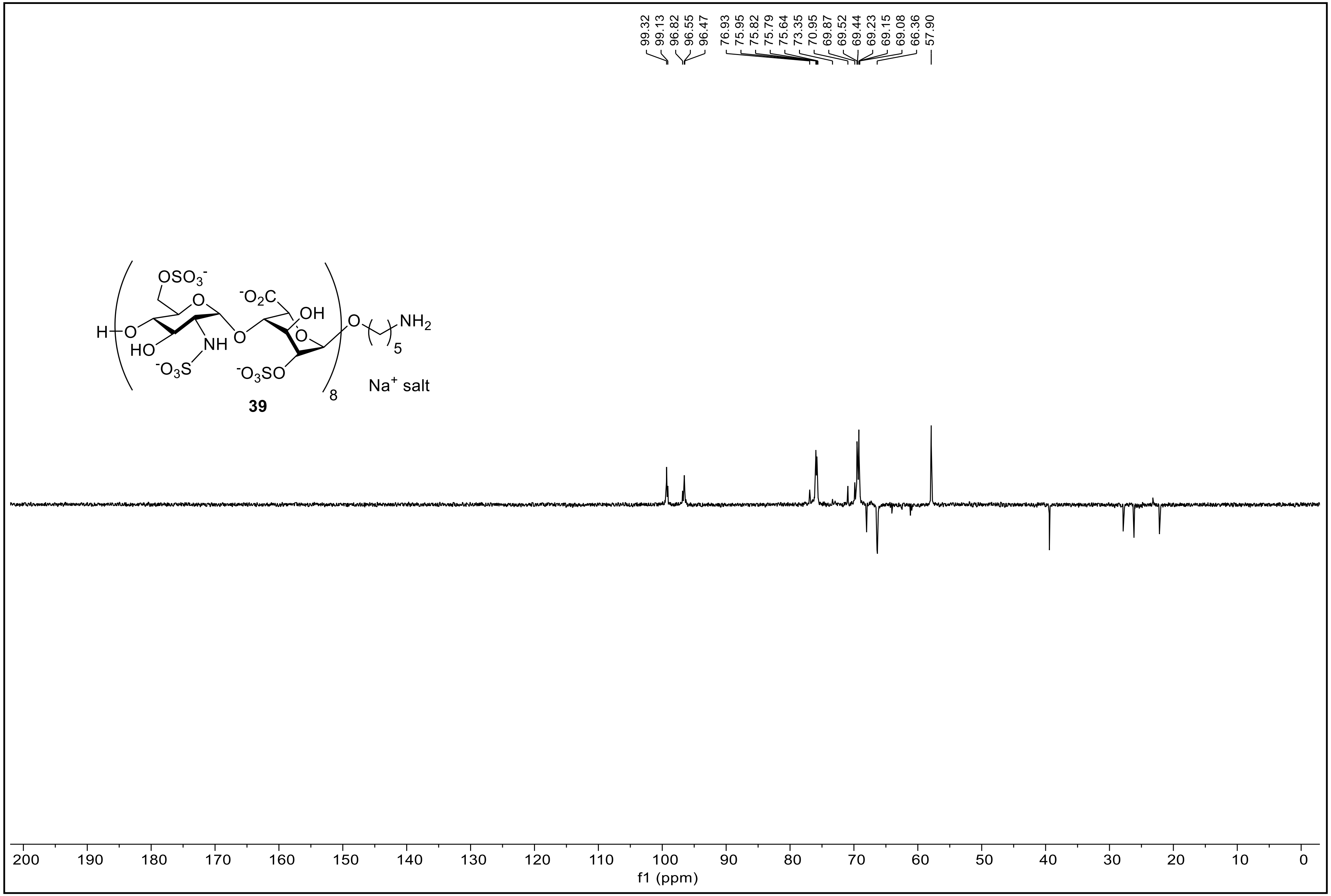
99.32
99.13
96.82
96.55
96.47
76.93
75.95
75.82
75.79
75.64
73.35
70.95
69.87
69.52
69.44
69.23
69.15
69.08
66.36
57.90
OSO3-
-O2C
OH
H
O
HO
NH
-O3S
-O3SO
NH2
5
8
Na+ salt
39
200 190 180 170 160 150 140 130 120 110 100 90 80 70 60 50 40 30 20 10 0
f1 (ppm)

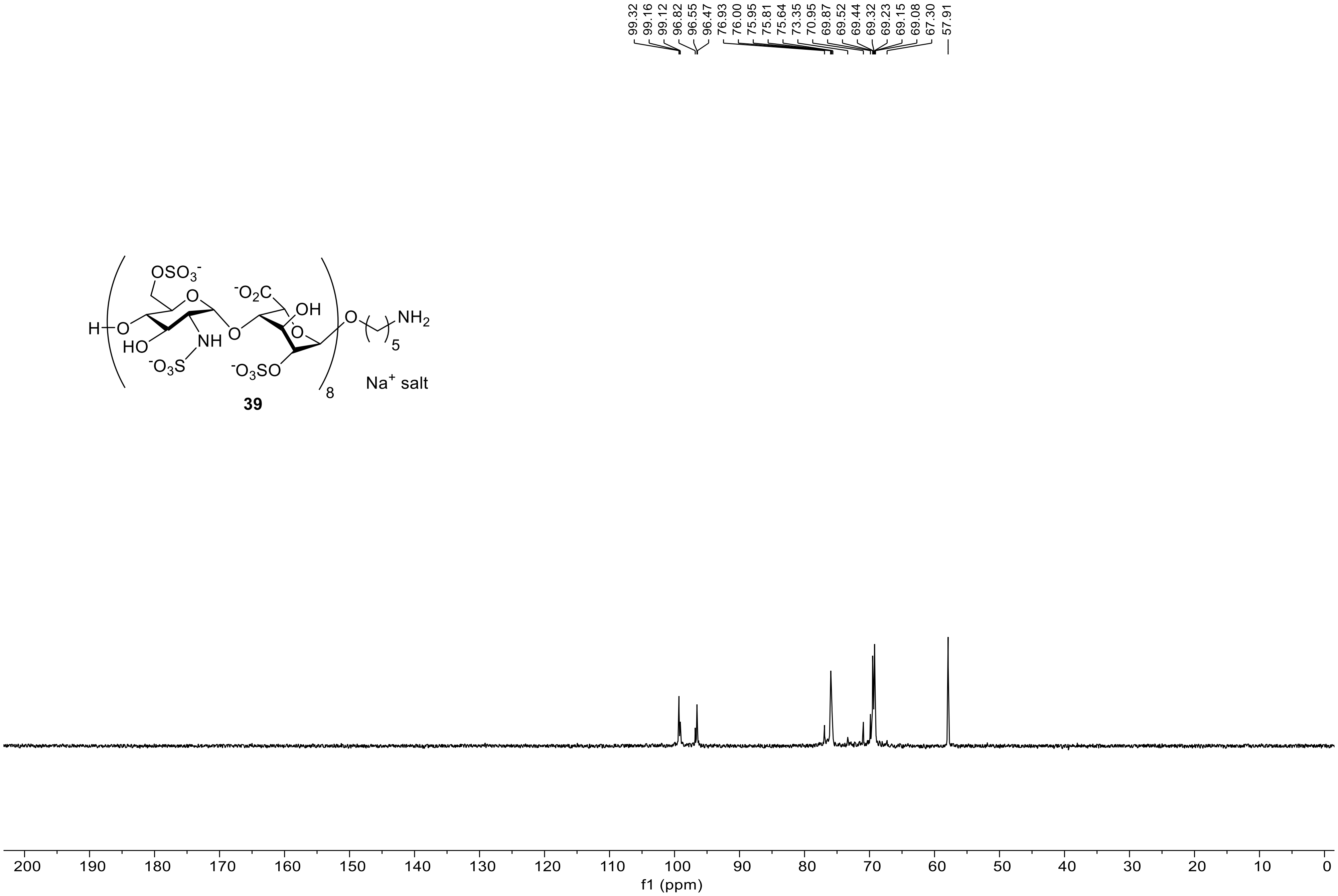
99.32
99.16
99.12
96.82
96.55
96.47
76.93
76.00
75.95
75.81
75.64
73.35
70.95
69.87
69.52
69.44
69.32
69.23
69.15
69.08
67.30
57.91
Na+ salt
39
f1 (ppm)

HRMS-ESI

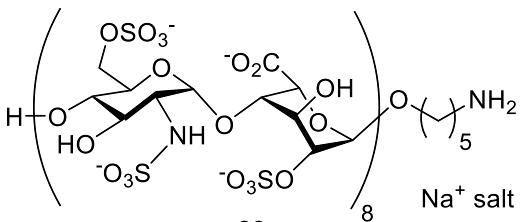

39

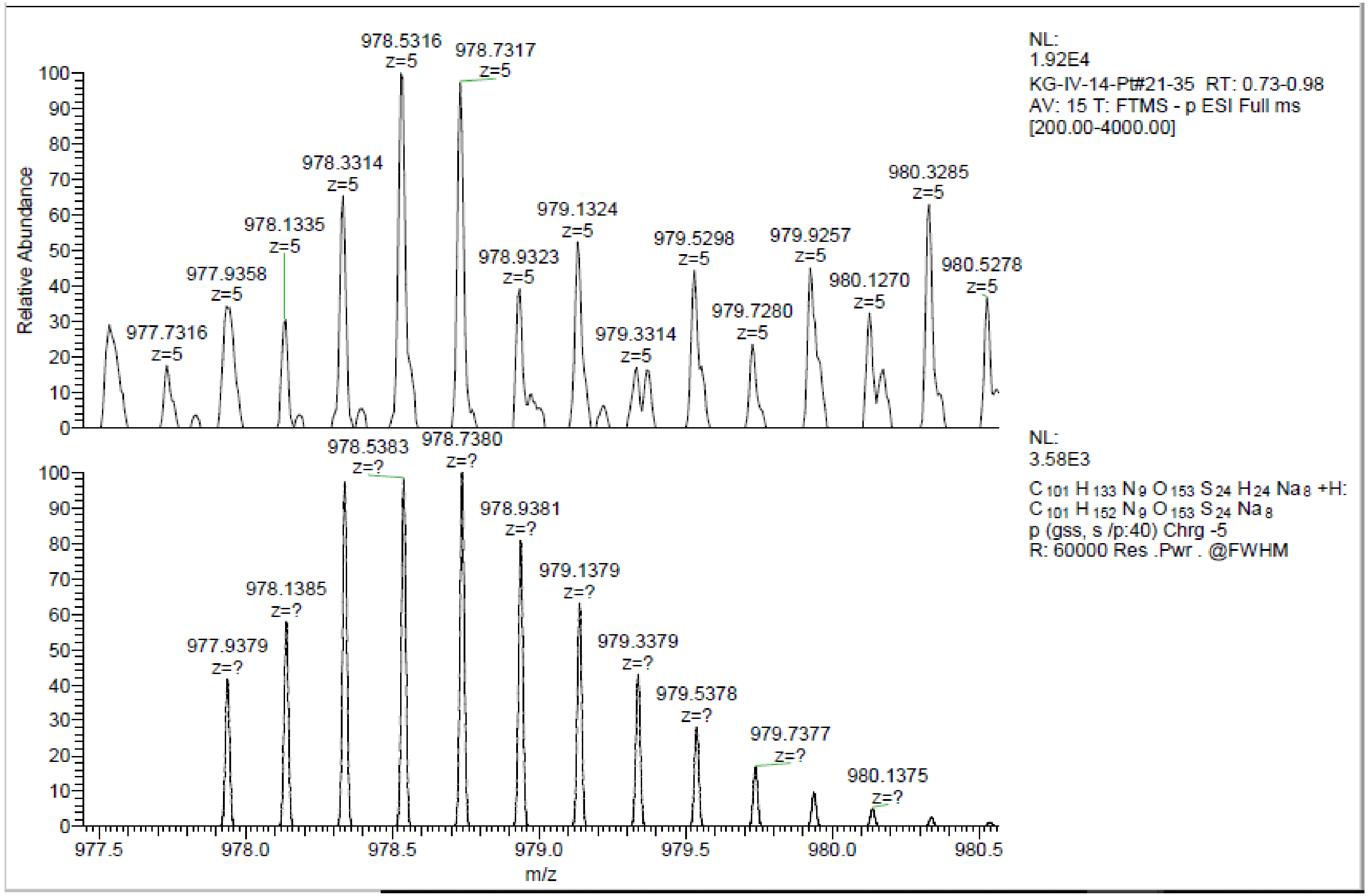

$(M + 19H^{+} - 24Na^{+})^{-5}$

Calculated : 977.9379

Found : 977.9358

Mass Error : 2.15 ppm

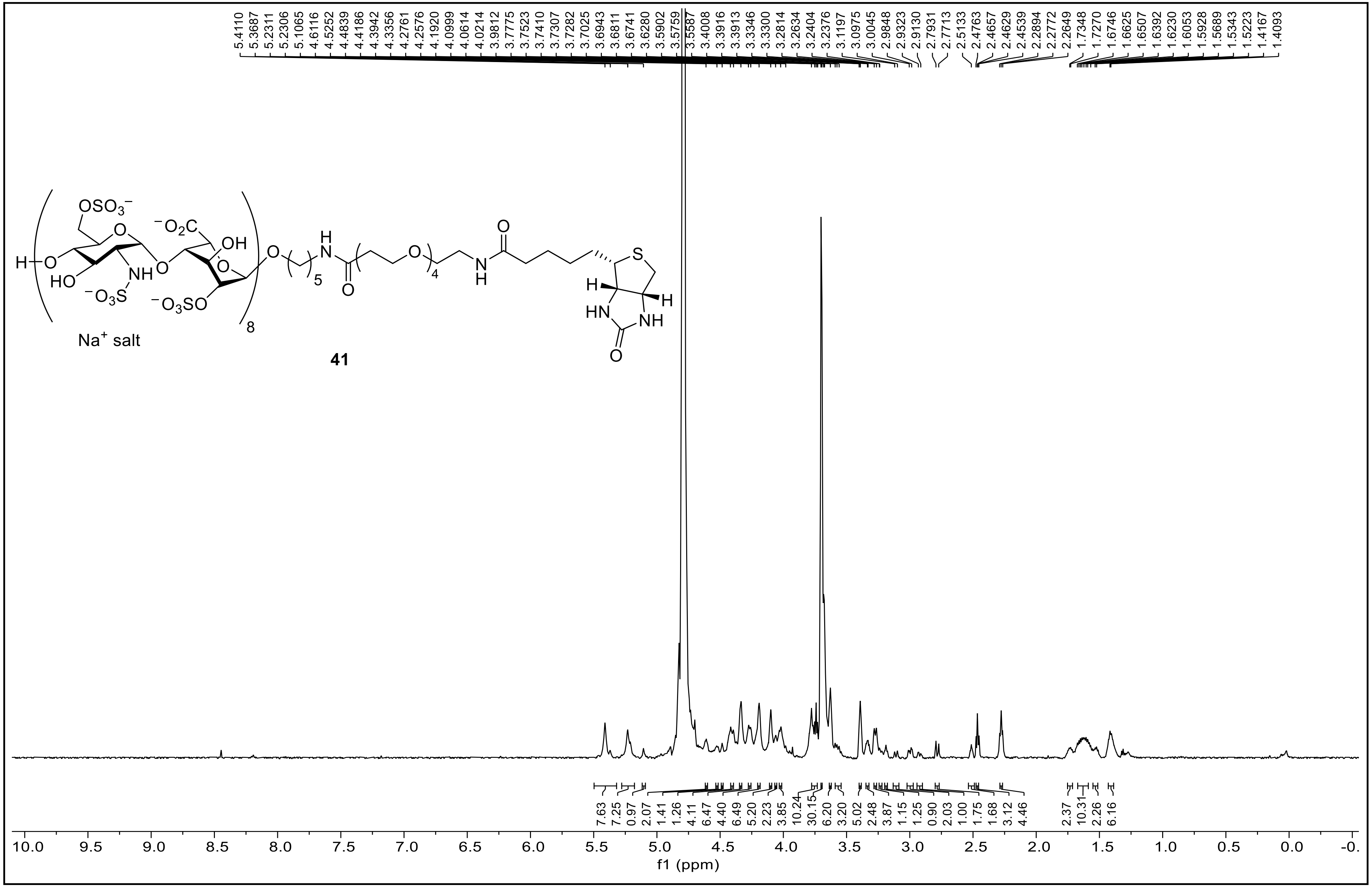
Na+ salt
41
f1 (ppm)

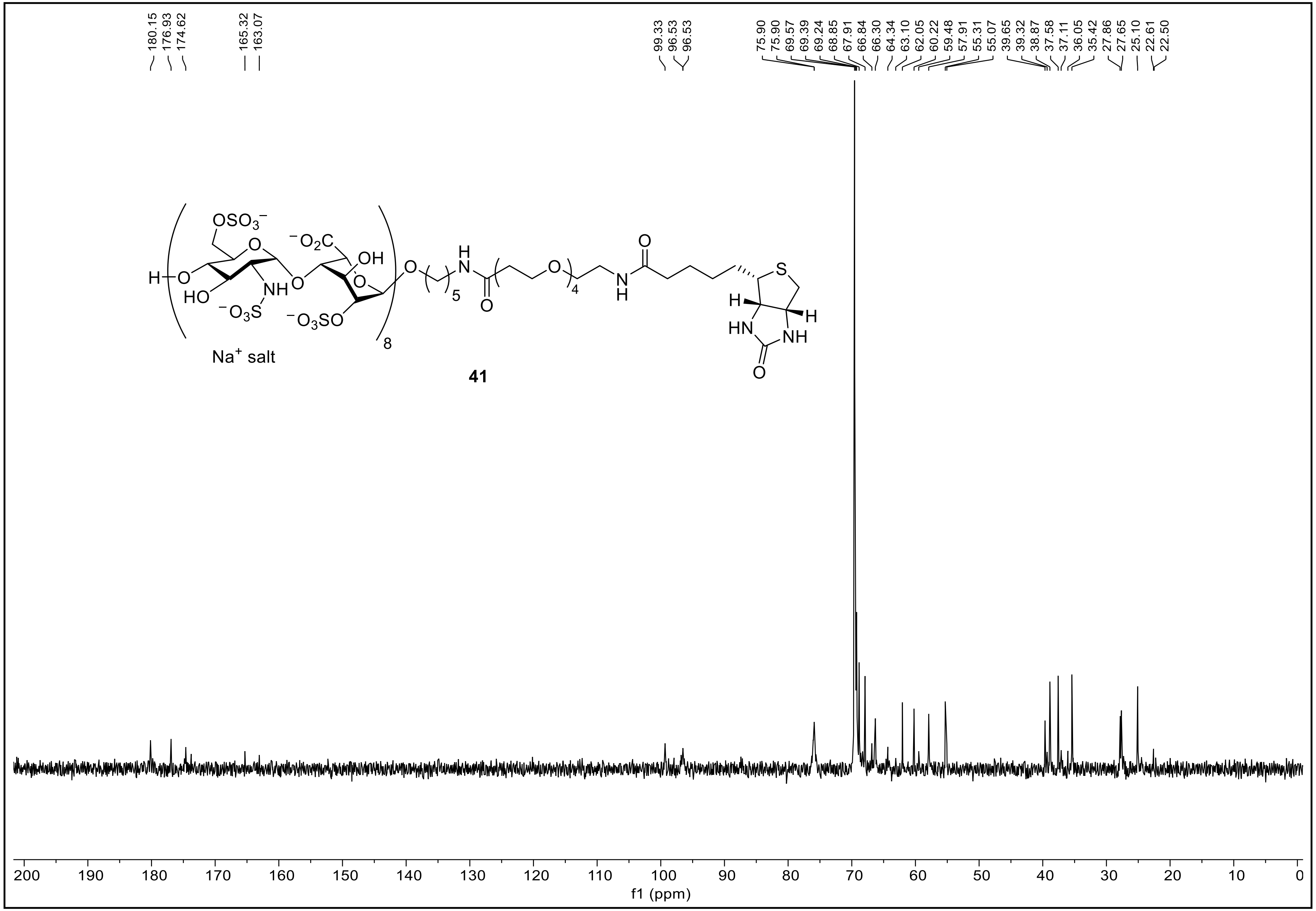
180.15
176.93
174.62
165.32
163.07
99.33
96.53
96.53
75.90
75.90
69.57
69.39
69.24
68.85
67.91
66.84
66.30
64.34
63.10
62.05
60.22
59.48
57.91
55.31
55.07
39.65
39.32
38.87
37.58
37.11
36.05
35.42
27.86
27.65
25.10
22.61
22.50
Na+ salt
41
f1 (ppm)

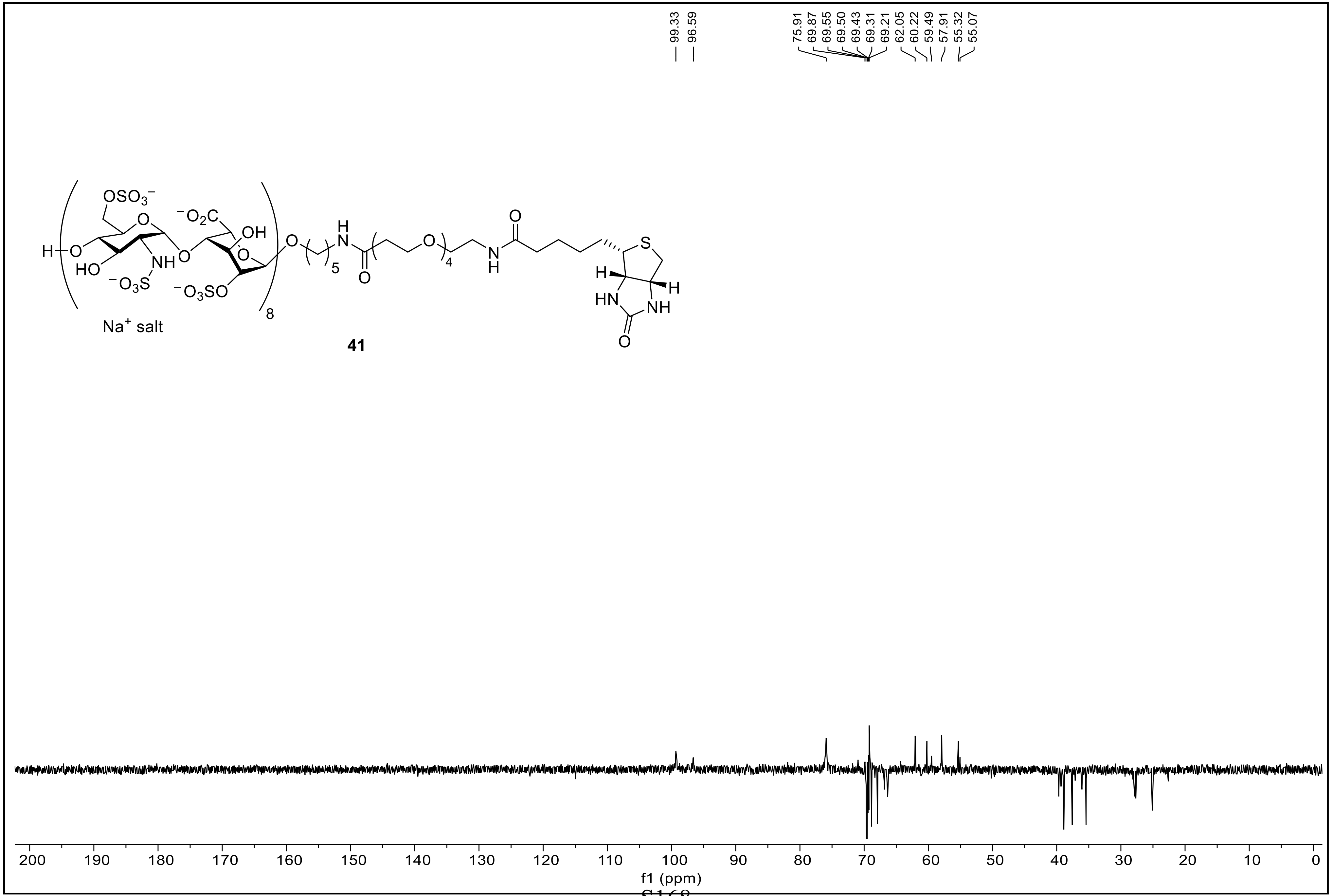
99.33
96.59
75.91
69.87
69.55
69.50
69.43
69.31
69.21
62.05
60.22
59.49
57.91
55.32
55.07
Na+ salt
41
f1 (ppm)

HRMS-ESI

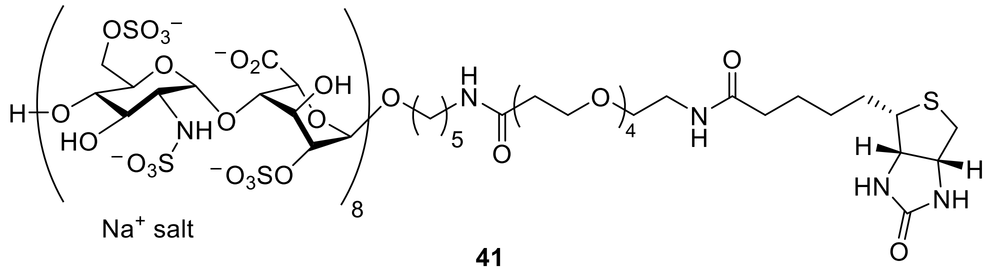

41

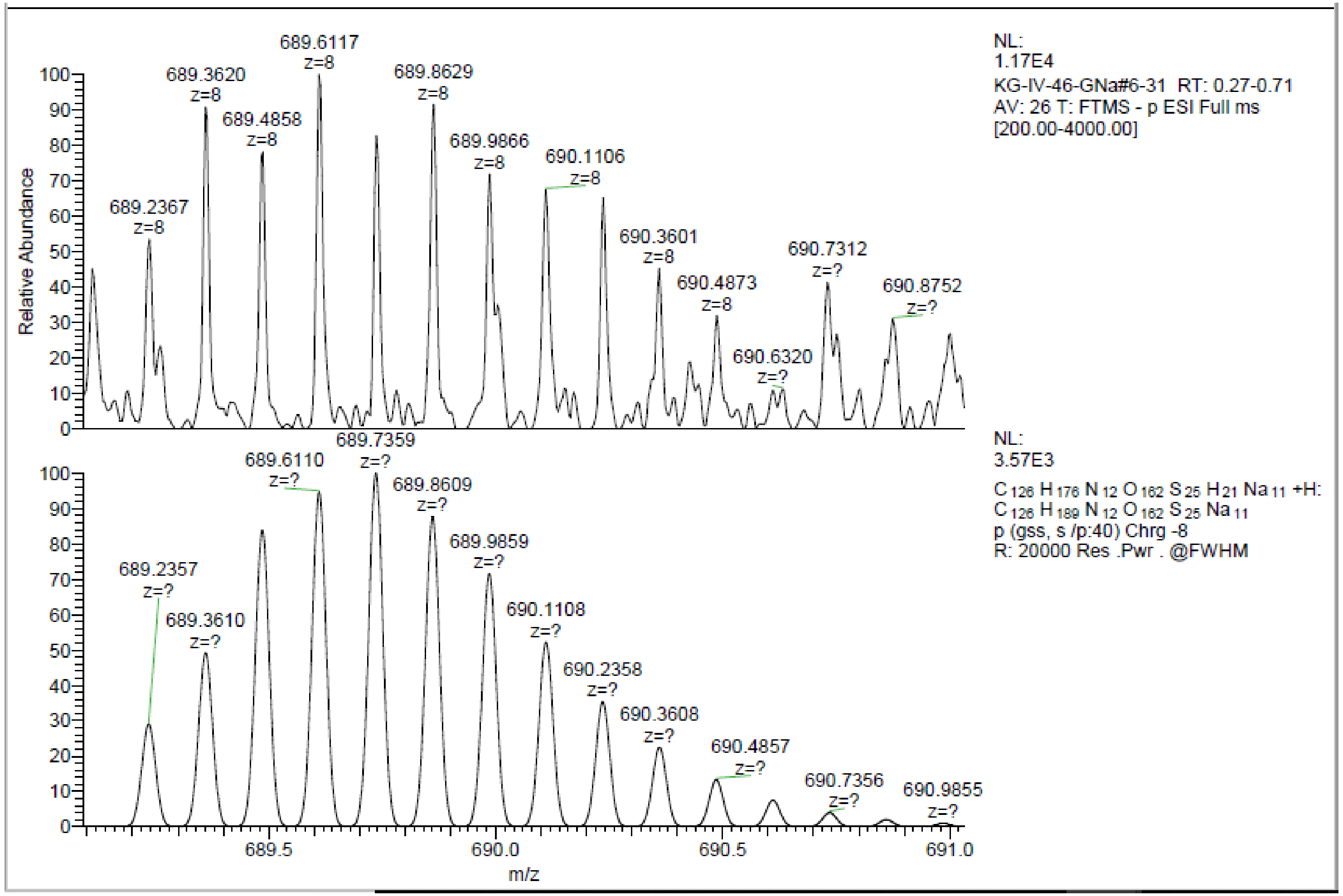

$(M + 13H^{+} - 21Na^{+})^{-8}$

Calculated : 689.2357

Found : 689.2367

Mass Error : 1.45 ppm

References

1. Huang, T.-Y.; Irene, D.; Zulueta, M. M. L.; Tai, T.-J.; Lain, S.-H.; Cheng, C.-P.; Tsai, P.-X.; Lin, S.-Y.; Chen, Z.-G.; Ku, C.-C.; Hsiao, C.-D.; Chyan, C.-L.; Hung, S.-C. Structure of the complex between a heparan sulfate octasaccharide and mycobacterial heparin-binding hemagglutinin. *Angew. Chem., Int. Ed.* **2017**, *56*, 4192–4196.
2. Hricovíni, M.; Bízik, F. Relationship between structure and three-bond proton–proton coupling constants in glycosaminoglycans. *Carbohydr. Res.* **2007**, *342*, 779–783.
3. Hricovíni, M.; Driguez, P.-A.; Malkina, O. L. NMR and DFT analysis of trisaccharide from heparin repeating sequence. *J. Phys. Chem. B* **2014**, *118*, 11931–11942.
4. Guerrini, M.; Guglieri, S.; Beccati, D.; Torri, G.; Viskov, C.; Mourier, P. Conformational transitions induced in heparin octasaccharides by binding with antithrombin III. *Biochem. J.* **2006**, *399*, 191–198.
5. Zhang, Z.; McCallum, S. A.; Xie, J.; Nieto, L.; Corzana, F.; Jiménez-Barbero, J.; Chen, M.; Liu, J.; Linhardt, R. J. Solution structures of chemoenzymatically synthesized heparin and its precursors. *J. Am. Chem. Soc.* **2008**, *130*, 12998–13007.
6. Hung, S.-C., Lu, X.-A., Lee, J.-C., Chang, M. D.-T., Fang, S.-L., Fan, T.-C., Zulueta, M. M. L., Zhong, Y.-Q. Synthesis of heparin oligosaccharides and their interaction with eosinophil-derived neurotoxin. *Org. Biomol. Chem.* **2012**, *10*, 760–772.
7. Zulueta, M. M. L.; Lin, S.-Y.; Lin, Y.-T.; Huang, C.-J.; Wang, C.-C.; Ku, C.-C.; Shi, Z.; Chyan, C.-L.; Irene, D.; Lim, L.-H.; Tsai, T.-I.; Hu, Y.-P.; Arco, S. D.; Wong, C.-H.; Hung, S.-C. α-Glycosylation by D-glucosamine-derived donors: synthesis of heparosan and heparin analogues that interact with mycobacterial heparin-binding hemagglutinin. *J. Am. Chem. Soc.* **2012**, *134*, 8988–8995.
8. Whitmore, L.; Wallace, B. A. Protein secondary structure analyses from circular dichroism spectroscopy: methods and reference databases. *Biopolymers* **2008**, *89*, 392–400.
9. Whitmore, L.; Wallace, B. A. DICHROWEB, an online server for protein secondary structure analyses from circular dichroism spectroscopic data. *Nucleic Acids Res.* **2004**, *32*, W668–W673.
10. Coxon, B. Developments in the Karplus equation as they relate to the NMR coupling constants of carbohydrates. *Adv. Carbohydr. Chem. Biochem.* **2009**, *62*, 17–82.
11. Kudoh, S.; Kudoh, T. A simple technique for culturing *tubercle bacilli*. *Bull. World Health Organ.* **1974**, *51*, 71–82.

12. Chang, W.-H.; Wang, C.-H.; Yang, S.-Y.; Lin, Y.-C.; Wu, J.-J.; Lee, M. S.; Lee, G.-B. Rapid isolation and diagnosis of live bacteria from human joint fluids by using an integrated microfluidic system. *Lab Chip* **2014**, *14*, 3376–3384.
13. Wang, C.-H.; Chang, J.-R.; Hung, S.-C.; Dou, H.-Y.; Lee, G.-B. Rapid molecular diagnosis of live *Mycobacterium tuberculosis* on an integrated micro-fluidic system. *Sens. Actuators B: Chem.* **2022**, *365*, 131968.
